# Carbene-Catalyzed Atroposelective Annulation for Quick Access to Axially Chiral Thiazine Derivatives

**DOI:** 10.3390/molecules28104052

**Published:** 2023-05-12

**Authors:** Xiaoqun Yang, Tingting Li, Jinli Chen, Yixian Huang, Tingwei Shen, Shiguang Li, Zhichao Jin, Shi-Chao Ren

**Affiliations:** National Key Laboratory of Green Pesticides, Key Laboratory of Green Pesticide and Agricultural Bioengineering, Ministry of Education, Guizhou University, Huaxi District, Guiyang 550025, China; yangxiaoqun95@163.com (X.Y.); litt8293@163.com (T.L.); m15287839083@163.com (J.C.); hyx13608506867@163.com (Y.H.); m15723160631@163.com (T.S.); 18711336599@163.com (S.L.)

**Keywords:** *N*-heterocyclic carbene, annulation reaction, thiazine, organocatalysis

## Abstract

An *N*-heterocyclic carbene (NHC)-catalyzed atroposelective annulation reaction is disclosed for quick and efficient access to thiazine derivatives. A series of axially chiral thiazine derivatives bearing various substituents and substitution patterns were produced in moderate to high yields with moderate to excellent optical purities. Preliminary studies revealed that some of our products exhibit promising antibacterial activities against *Xanthomonas oryzae* pv. oryzae (*Xoo*) that causes rice bacterial blight.

## 1. Introduction

**Figure 1:**
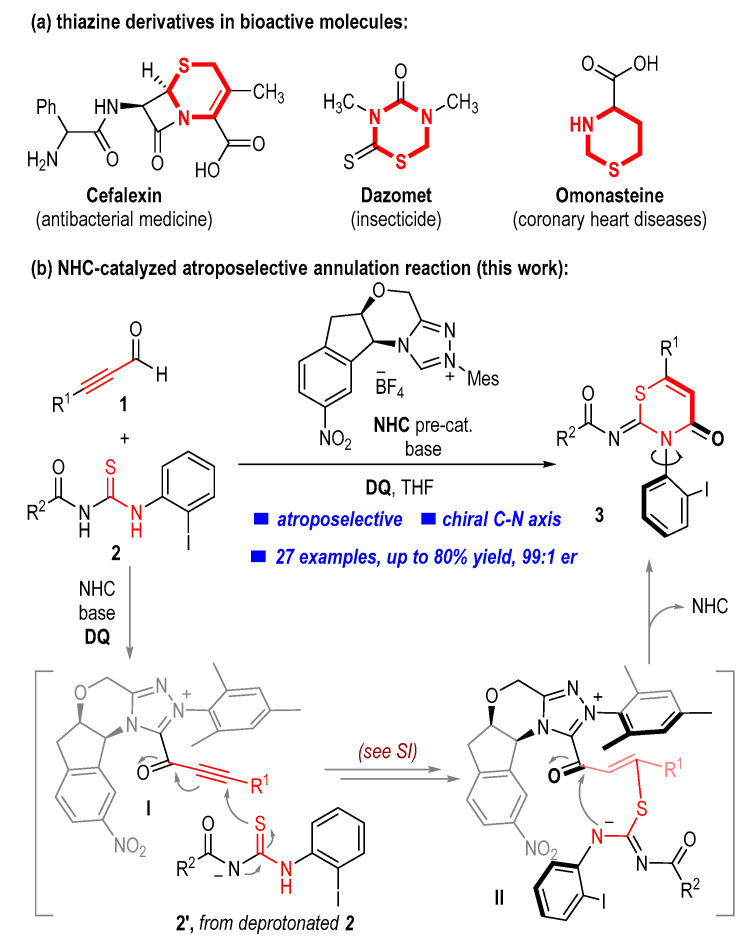
Bioactive molecules containing thiazine rings and our project design.

Thiazine is a privileged structure that exists in a good number of natural products, medicines, and agrochemicals with proven biological activities (Figure 1a) [1,2,3,4,5]. For example, thiazine-containing cephalosporins such as cefalexin [6] have been used to cure or prevent bacterial infections. Dazomet [7] is a commercially available insecticide used to protect crops from various pests. Omonasteine [8] bearing a hydrothiazine fragment can be used in the curing of coronary heart diseases. Some molecules containing thiazine moiety have also shown potential antifungal or antibacterial activities in the field of pesticides [9,10,11]. Therefore, the development of quick and efficient strategies to construct thiazine scaffolds from readily available chemicals is of particular interest and has certainly attracted considerable attention in organic synthesis [12,13,14,15,16,17,18,19]. On the other hand, axial chirality is a common phenomenon in modern organic synthesis and living systems. Axially chiral molecules have found wieldy applications in catalysts [20,21], ligands [22,23], and medicines [24,25]. The incorporation of thiazine moiety into the axial skeletons would result in unique physical and chemical properties and will certainly bring wide potential applications.

*N*-heterocyclic carbenes (NHCs) have emerged as powerful organocatalysts for the asymmetric synthesis of structurally diverse heterocyclic molecules due to their unique Lewis basicity and nucleophilicity [26,27,28,29,30,31,32,33,34,35,36,37,38,39,40,41,42,43,44,45,46,47,48,49]. However, most of the chiral compounds are provided by the introduction of point-chiral centers. On the contrary, the chiral molecules with axial chirality obtained from asymmetric synthesis enabled by NHC organocatalysis are still relatively scarce and challenging [50,51,52,53]. Based on the continuous interest in harnessing NHC catalysis for asymmetric construction of bioactive chiral molecules, we herein report a carbene-catalyzed atroposelective annulation of alkynyl aldehyde and benzoylthiourea to produce axially chiral thiazine derivatives (Figure 1b). Key steps of our protocol include the nucleophilic addition of the deprotonated benzoylthiourea **2** to the acetylenic acylazolium intermediate **I** (generated from alkynyl aldehyde **1** and NHC catalyst) to forge the C-S bond. Subsequent proton transfer and tautomerization of the intermediate **I** afford an NHC-bound acyl azolium intermediate **II**, which then undergoes intramolecular nucleophilic addition and elimination steps to deliver the desired axially chiral product and regenerate the NHC catalyst (see SI for detailed proposed reaction mechanism). A series of axially chiral thiazine derivatives bearing various substituents and substitution patterns were produced by using this method in moderate to high yields with moderate to excellent optical purities. Compared to our previously reported atroposelective annulation between alkyl-substituted thioureas and ynals [54], the incorporation of iodine atoms into the axially chiral thiazine derivatives results in better antibacterial activities. For example, some of such axially chiral products exhibit promising antibacterial activities against *Xanthomonas oryzae* pv. oryzae (*Xoo*) that causes rice bacterial blight.

## 2. Results and Discussion

The alkynyl aldehyde **1a** and benzoylthiourea **2a** were selected as model substrates to evaluate the feasibility of the axially selective cyclization. A typical reaction mixture contained **1a** (2 equivalents), **2a** (1 equivalent), an NHC (20 mol%) as a catalyst, tetra-tert- butyldiphenylquinone (**DQ**, 2 equivalents) as the oxidant, and 4-dimethylaminopyridine (DMAP, 1.0 equivalent) as a base in furan as the solvent. Various aminoindanol-derived NHC precatalysts were evaluated (e.g., Table 1, entries 1–5). It was found that the NHC precatalysts bearing electron-rich N-aryl groups [55,56,57] could deliver the thiazine derivative **3a** in lower yields with moderate optical purities (entries 1–2). On the contrary, the NHC precatalyst **C** [58] bearing an electron-poor N-C_6_F_5_ group gave only a trace amount of the desired product **3a** (entry 3). Installation of a NO_2_ group (**D**) [59] or a Br group (**E**) [60] resulted in similar yields but with slightly improved enantioselectivity (entries 4–5). Then, the effects of base and solvent were explored by using **D** as the optimal NHC precatalyst. Replacing the DMAP with various organic or inorganic bases resulted in decreased yields, albeit with similar or slightly increased enantioselectivities (entries 6–8). The solvent was proved to have a significant impact on both the reaction outcome and enantioselectivity (entries 9–11). An acceptable yield and high enantioselectivity were obtained when tetrahydrofuran was used as a solvent (entry 9).

**Scheme 1:**
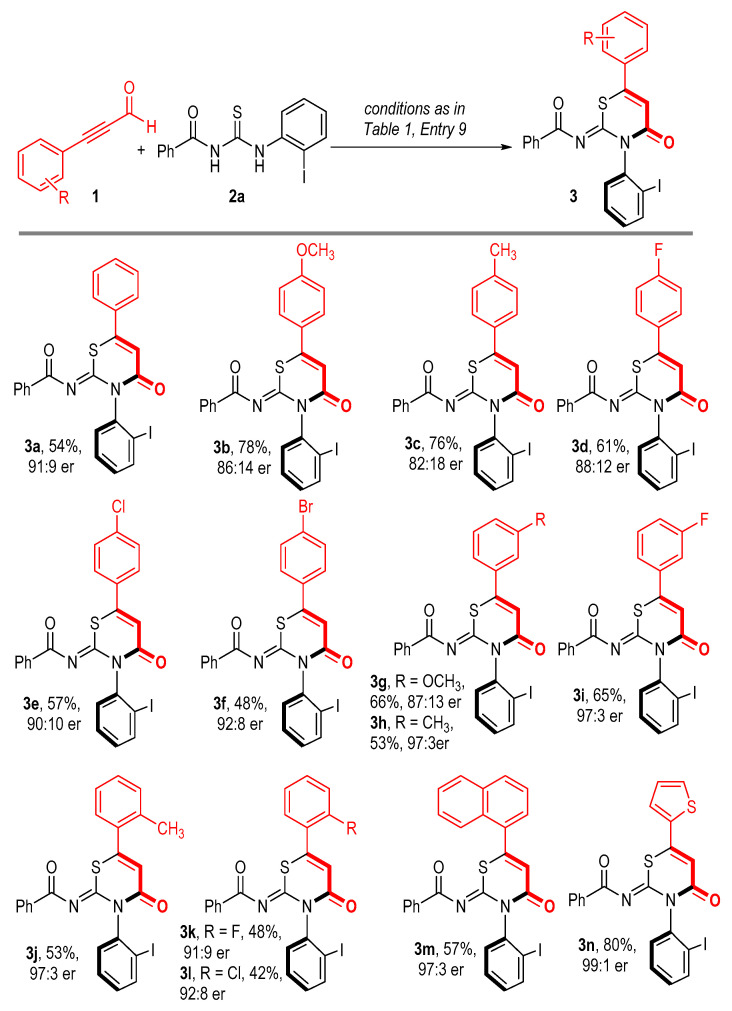
Scope of ynals **1** [a]. [a] All reactions were carried out at 30 °C using 1 (0.2 mmol), **2a** (0.1 mmol), pre-NHC **D** (20 mol%), DMAP (1.0 equiv), **DQ** (0.2 mmol), and 2.0 mL THF for 12 h.

**Figure 2:**
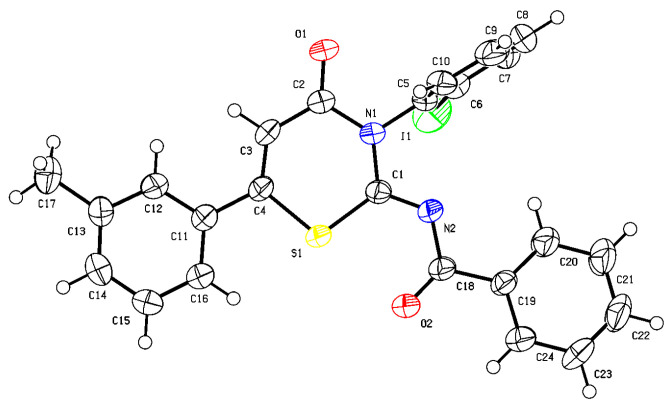
X-ray crystallography of **3h** (black for carbon atoms, green for iodine atom, yellow for sulfur atom, dark blue for nitrogen atoms, and red for oxygen atoms), CCDC number: 2255058.

With the optimized reaction conditions in hand, we turned to evaluate the scope of our axially selective annulation reaction. The scope of alkynyl aldehyde (**1**) was first evaluated by using benzoylthiourea **2a** as the model reaction partner (Scheme 1). Various functional groups on the 4-position of the phenyl group of **1a**, such as methoxyl (**3b**), methyl (**3c**), and halides (**3d**–**3f**), were tolerated, giving the corresponding axially chiral products in moderate to good yields with maintained or slightly decreased enantioselectivities, regardless of their electronic nature. Substrates bearing a substituent on the 3-position of the benzene ring proceeded to smoothly produce the target products in moderate yields with good to excellent enantioselectivities. For example, **3h**–**3i** were obtained in 53% and 65% yields, respectively, and a 97:3 enantiomeric ratio. The structure of **3h** was further characterized by X-ray single crystal diffraction analysis (Figure 2). The steric effect on the benzene ring has little influence on our reaction since alkynyl aldehydes bearing methyl (**3j**) or halides (**3k**–**3l**) on the 2-position of the benzene ring were all converted to the corresponding products in acceptable yields and with good optical purities. Other aromatic systems such as naphthalene and thiophene were also tolerated under the standard conditions affording **3m**–**3n** in good yields with excellent optical purities.

**Scheme 2:**
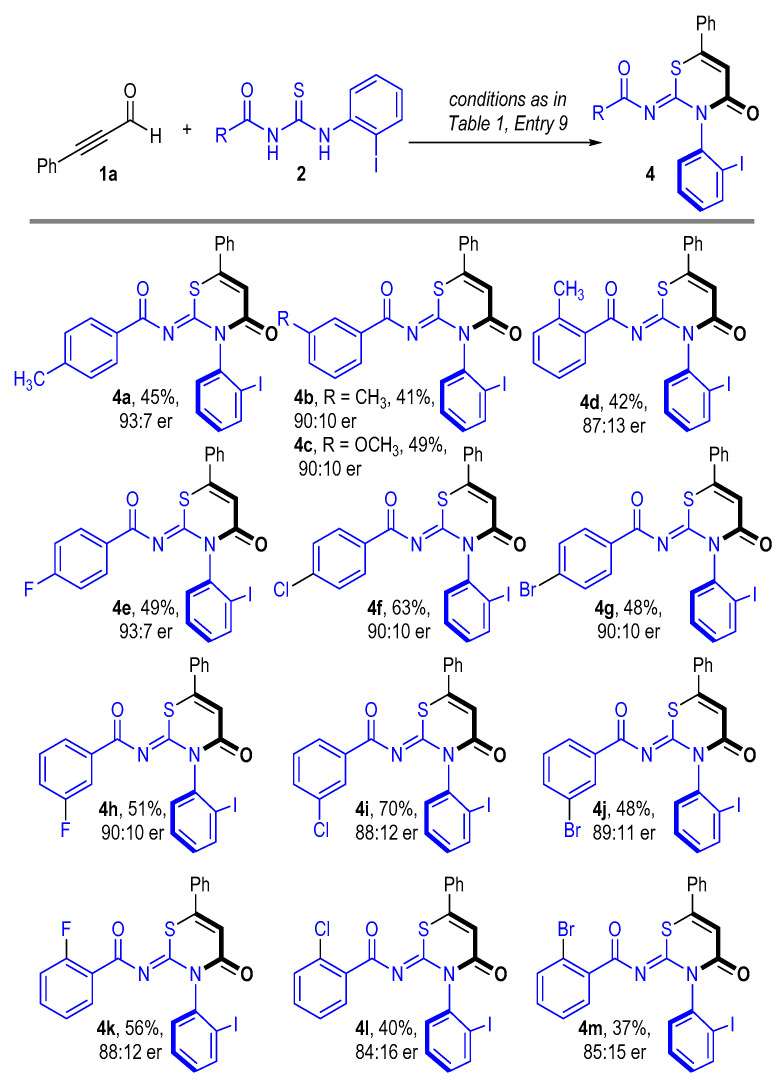
Scope of thioureas **2** [a]. [a] All reactions were carried out at 30 °C using 1a (0.2 mmol), **2** (0.1 mmol), pre-NHC **D** (20 mol%), DMAP (1.0 equiv), **DQ** (0.2 mmol), and 2.0 mL THF for 12 h.

We then examined the scope of benzoylthiourea **2** using **1a** as the model substrate. As disclosed in Scheme 2, both electron-donating (**4a**–**4d**) and electron-withdrawing (**4e**–**4m**) functional groups can be decorated on the benzene ring of benzoylthiourea **2** to produce the corresponding axially chiral thiazine-containing products with acceptable to good yields and enantioselectivities. Halide atoms including fluorine, chlorine, and bromine were all tolerated to afford halide atom-containing products, regardless of their substituted position (**4e**–**4m**). Such halide atom-substituted products provide opportunities for further transformations.

**Figure 3:**
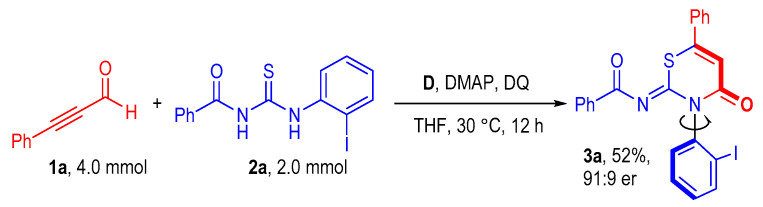
Up-scaled reaction.

To test the potential utility of this carbene-catalyzed atroposelective annulation reaction, the model reaction was conducted in 2.0 mmol scale. To our delight, the desired axially chiral **3a** was isolated in a maintained yield (52%) without any loss of the enantioselectivity (Figure 3).

Based on the fantastic bioactivities of the thiazine derivatives and our continuous interest in searching for unique small molecules bearing antiviral and antibacterial activities in agricultural applications, the antibacterial activities of our axially chiral thiazine derivatives against *Xanthomonas oryzae* pv. oryzae (*Xoo*) that cause rice bacterial blight [61] were evaluated and are summarized in Table 2. To our delight, many of our chiral products exhibited superior bioactivities to the commercial thiodiazole copper (TC) and bismerthiazol (BT).

## 3. Materials and Methods

### 3.1. General Information

Commercially available materials and dry solvents purchased from Energy Chemical and J&K were used as received. Unless otherwise specified, all reactions were prepared using 4 mL vials. NMR spectra were measured on a Bruker ASCEND (AVANCE III HD 400 MHz) spectrometer. The chemical shift values were corrected to 7.26 ppm (^1^H NMR) and 77.23 ppm (^13^C NMR) for CDCl_3_ or 3.33 ppm (^1^H NMR) and 39.51 ppm (^13^C NMR) for DMSO-*d*_6_. ^1^H NMR splitting patterns were designated as singlet (s), double (d), triplet (t), quartet (q), doublet of doublets (dd), multiplets (m), etc. All first-order splitting patterns were assigned on the basis of the appearance of the multiplet. Splitting patterns that could not be easily interpreted are designated as multiplet (m) or broad (br). High-resolution mass spectrometer analysis (HRMS) was performed on a Thermo Fisher Q Exactive mass spectrometer (QTOF mass analyzer). HPLC analyses were measured on Shimadzu Model SIL-20AC220V instruments. Chiralcel brand chiral columns from Daicel Chemical Industries were used with models IA, IB, ODH in 4.6 × 250 mm size. UPLC analyses were measured on Waters systems with an Empower3 system controller, Waters UPLC H-Class, and Waters ACQUITY UPLC PDA detector. Chiralcel brand chiral columns from Daicel Chemical Industries were used with models AD-3, OD-3 in 3.0 × 100 mm size. The racemic products used to determine the er values were synthesized using a racemic catalyst. Optical rotations were measured on an Insmark IP-digi Polarimeter in a 1 dm cuvette. The concentration (*c*) is given in g/100 mL. Melting points were measured on an uncorrected Beijing Tech Instrument X-4 digital display micro melting point apparatus. Single-crystal X-ray diffraction was recorded at Xcalibur, Eos, Gemini. Analytical thin-layer chromatography (TLC) was carried out on precoated silica gel plates (0.2 mm thickness). Visualization was performed using a UV lamp.

### 3.2. General Procedure for the Synthesis of Target Compounds ***3*** or ***4***

To a 4 mL vial equipped with a magnetic stir bar, chiral NHC precatalyst **D** (0.02 mmol, 20 mol%, 9.28 mg), DMAP (0.1 mmol, 100 mol%, 12.22 mg), **DQ** (0.2 mmol, 200 mol%, 81.73 mg), and substituted thiourea **2** (0.1 mmol) were added. After that, THF (2.0 mL) and ynal **1** (0.2 mmol) were added and the reaction mixture was allowed to stir for 12 h at 30 °C. The solution was then concentrated under reduced pressure, and the residue was subjected to column chromatography (petroleum ether/ethyl acetate = 50/1 to 20/1) directly to obtain the products **3** or **4**.

*(Z)-N-(3-(2-iodophenyl)-4-oxo-6-phenyl-3,4-dihydro-2H-1,3-thiazin-2-ylidene)benzamide* (**3a**), white solid, 54% yield, 27.5 mg, m.p. 134–136 °C; ${\left[\alpha \right]}_{D}^{23}$ = +54.3 (*c* = 0.5 in CHCl_3_); ^1^H NMR (400 MHz, CDCl_3_) δ 8.04 (dd, *J* = 8.0, 1.4 Hz, 1H), 7.81–7.78 (m, 2H), 7.72–7.69 (m, 2H), 7.58–7.45 (m, 5H), 7.35–7.31 (m, 3H), 7.22 (td, *J* = 7.6, 1.6 Hz, 1H), 6.94 (s, 1H); ^13^C NMR (101 MHz, CDCl_3_) δ 175.6, 161.8, 160.4, 151.6, 141.4, 140.0, 135.0, 134.4, 133.1, 131.8, 130.13, 130.11, 129.7, 129.4, 129.1, 128.3, 126.8, 115.8, 98.0; HRMS (ESI-TOF) *m*/*z*: [M + Na]^+^ calcd for C_23_H_15_IN_2_O_2_SNa 532.9791, found 532.9786; UPLC analysis: 91:9 er (OD-3 column, 25 °C, hexane/iPrOH = 80/20, 0.5 mL/min, *λ* = 254 nm), Rt (major) = 25.9 min, Rt (minor) = 33.1 min.
*(Z)-N-(3-(2-iodophenyl)-6-(4-methoxyphenyl)-4-oxo-3,4-dihydro-2H-1,3-thiazin-2-ylidene)benzamide* (**3b**), white solid, 78% yield, 42.3 mg, m.p. 118–120 °C; ${\left[\alpha \right]}_{D}^{27}$ = +52.5 (*c* = 1.0 in CHCl_3_); ^1^H NMR (400 MHz, CDCl_3_) δ 8.03 (dd, *J* = 8.0, 1.4 Hz, 1H), 7.82–7.79 (m, 2H), 7.69–7.65 (m, 2H), 7.55 (td, *J* = 7.6, 1.4 Hz, 1H), 7.49–7.45 (m, 1H), 7.35–7.31 (m, 3H), 7.21 (td, *J* = 7.6, 1.6 Hz, 1H), 7.03–7.0 (m, 2H), 6.88 (s, 1H), 3.88 (s, 3H); ^13^C NMR (101 MHz, CDCl_3_) δ 175.6, 162.6, 162.0, 160.4, 150.9, 141.5, 139.8, 135.1, 133.0, 130.11, 130.05, 129.6, 129.2, 128.4, 128.3, 126.5, 114.8, 113.9, 98.1, 55.6; HRMS (ESI-TOF) *m*/*z*: [M + Na]^+^ calcd for C_24_H_17_IN_2_O_3_SNa 562.9897, found 562.9892; HPLC analysis: 86:14 er (AD-3 column, 25 °C, hexane/iPrOH = 80/20, 0.5 mL/min, *λ* = 254 nm), Rt (major) = 35.2 min, Rt (minor) = 42.3 min.
*(Z)-N-(3-(2-iodophenyl)-4-oxo-6-(p-tolyl)-3,4-dihydro-2H-1,3-thiazin-2-ylidene)benzamide* (**3c**), white solid, 76% yield, 40.0 mg, m.p. 156–158 °C; ${\left[\alpha \right]}_{D}^{25}$ = +17.3 (*c* = 1.0 in CHCl_3_); ^1^H NMR (400 MHz, CDCl_3_) δ 8.04 (dd, *J* = 8.0, 1.4 Hz, 1H), 7.78–7.76 (m, 2H), 7.57 (td, *J* = 7.6, 1.4 Hz, 1H), 7.49–7.44 (m, 1H), 7.39–7.29 (m, 7H), 7.22 (td, *J* = 7.6, 1.6 Hz, 1H), 6.64 (s, 1H), 2.46 (s, 3H); ^13^C NMR (101 MHz, CDCl_3_) δ 175.6, 161.5, 153.0, 141.5, 139.9, 135.6, 135.0, 134.1, 133.1, 131.1, 130.6, 130.1, 129.7, 129.2, 128.6, 128.3, 126.4, 119.2, 98.0, 19.9; HRMS (ESI-TOF) *m*/*z*: [M + Na]^+^ calcd for C_24_H_17_IN_2_O_2_SNa 546.9948, found 546.9941; HPLC analysis: 82:18 er (IB column, 25 °C, hexane/iPrOH = 80/20, 0.5 mL/min, *λ* = 254 nm), Rt (major) = 24.4 min, Rt (minor) = 25.9 min.
*(Z)-N-(6-(4-fluorophenyl)-3-(2-iodophenyl)-4-oxo-3,4-dihydro-2H-1,3-thiazin-2-ylidene)benzamide* (**3d**), white solid, 61% yield, 32.2 mg, m.p. 216–218 °C; ${\left[\alpha \right]}_{D}^{24}$ = +24.0 (*c* = 1.0 in CHCl_3_); ^1^H NMR (400 MHz, CDCl_3_) δ 8.04 (dd, *J* = 8.0, 1.4 Hz, 1H), 7.81–7.78 (m, 2H), 7.73–7.68 (m, 2H), 7.56 (td, *J* = 7.6, 1.4 Hz, 1H), 7.50–7.46 (m, 1H), 7.35–7.31 (m, 3H), 7.24–7.19 (m, 3H), 6.89 (s, 1H); ^13^C NMR (101 MHz, CDCl_3_) δ 175.6, 164.8 (d, *J* = 255.3 Hz), 161.7, 160.0 150.4, 141.3, 139.9, 134.9, 133.2, 130.58, 130.6 (d, *J* = 3.1 Hz), 130.1, 129.7, 129.1 (d, *J* = 4.2 Hz), 129.0, 128.3, 116.7 (d, *J* = 22.4 Hz), 115.8, 98.0; ^19^F NMR (377 MHz, CDCl_3_) δ -107.0; HRMS (ESI-TOF) *m*/*z*: [M + Na]^+^ calcd for C_23_H_14_FIN_2_O_2_SNa 550.9697, found 550.9698; HPLC analysis: 88:12 er (IA column, 25 °C, hexane/iPrOH = 80/20, 0.5 mL/min, *λ* = 254 nm), Rt (major) = 45.6 min, Rt (minor) = 48.8 m.
*(Z)-N-(6-(4-chlorophenyl)-3-(2-iodophenyl)-4-oxo-3,4-dihydro-2H-1,3-thiazin-2-ylidene)benzamide* (**3e**), white solid, 57% yield, 31.2 mg, m.p. 180–182 °C; ${\left[\alpha \right]}_{D}^{25}$ = +22.6 (*c* = 1.0 in CHCl_3_); ^1^H NMR (400 MHz, CDCl_3_) δ 8.04 (dd, *J* = 8.0, 1.4 Hz, 1H), 7.80–7.78 (m, 2H), 7.66–7.63 (m, 2H), 7.56 (td, *J* = 7.8, 1.4 Hz, 1H), 7.51–7.46 (m, 3H), 7.35–7.31 (m, 3H), 7.22 (td, *J* = 7.8, 1.6 Hz, 1H), 6.91 (s, 1H); ^13^C NMR (101 MHz, CDCl_3_) δ 175.6, 161.6, 160.0, 150.3, 141.3, 139.9, 138.2, 134.9, 133.2, 132.8, 130.2, 130.1, 129.8, 129.7, 129.1, 128.3, 128.1, 116.0, 98.0; HRMS (ESI-TOF) *m*/*z*: [M + Na]^+^ calcd for C_23_H_14_ClIN_2_O_2_SNa 566.9401, found 566.9391; HPLC analysis: 90:10 er (IB column, 25 °C, hexane/iPrOH = 80/20, 0.5 mL/min, *λ* = 254 nm), Rt (major) = 36.7 min, Rt (minor) = 40.1 min.
*(Z)-N-(6-(4-bromophenyl)-3-(2-iodophenyl)-4-oxo-3,4-dihydro-2H-1,3-thiazin-2-ylidene)benzamide* (**3f**), white solid, 48% yield, 28.2 mg, m.p. 94–96 °C; ${\left[\alpha \right]}_{D}^{22}$ = +10.0 (*c* =1.0 in CHCl_3_); ^1^H NMR (400 MHz, CDCl_3_) δ 8.04 (dd, *J* = 8.0, 1.4 Hz, 1H), 7.80–7.78 (m, 2H), 7.67–7.64 (m, 2H), 7.59–7.54 (m, 3H), 7.50–7.46 (m, 1H), 7.35–7.31 (m, 3H), 7.22 (td, *J* = 7.6, 1.6 Hz, 1H), 6.91 (s, 1H); ^13^C NMR (101 MHz, CDCl_3_) δ 175.6, 161.6, 160.0, 150.4, 141.3, 139.9, 134.9, 133.3, 133.2, 132.7, 130.2, 130.1, 129.7, 129.1, 128.3, 128.2, 126.6, 116.0, 98.0; HRMS (ESI-TOF) *m*/*z*: [M + Na]^+^ calcd for C_23_H_14_BrIN_2_O_2_SNa 610.8896, found 610.8893; UPLC analysis: 92:8 er (AD-3 column, 25 °C, hexane/iPrOH = 80/20, 0.5 mL/min, *λ* = 254 nm), Rt (major) = 46.2 min, Rt (minor) = 49.9 min.
*(Z)-N-(3-(2-iodophenyl)-6-(3-methoxyphenyl)-4-oxo-3,4-dihydro-2H-1,3-thiazin-2-ylidene)benzamide* (**3g**), white solid, 66% yield, 35.8 mg, m.p. 118–120 °C; ${\left[\alpha \right]}_{D}^{26}$ = +15.5 (*c* = 1.0 in CHCl_3_); ^1^H NMR (400 MHz, CDCl3) δ 8.04 (dd, *J* = 8.0, 1.4 Hz, 1H), 7.81–7.78 (m, 2H), 7.55 (dd, *J* = 7.8, 1.4 Hz, 1H), 7.50–7.45 (m, 1H), 7.42 (t, *J* = 8.0 Hz, 1H), 7.37–7.27 (m, 4H), 7.24–7.18 (m, 2H), 7.10–7.07 (m, 1H), 6.93 (s, 1H), 3.88 (s, 3H); ^13^C NMR (101 MHz, CDCl_3_) δ 175.5, 161.8, 160.34, 160.25, 151.6, 141.4, 139.9 (2C), 135.7, 135.0, 133.1, 130.5, 130.1, 129.7, 129.1, 128.3, 119.2, 117.7, 115.9, 111.9, 98.0, 55.6; HRMS (ESI-TOF) *m*/*z*: [M + Na]^+^ calcd for C_24_H_17_IN_2_O_3_SNa 562.9897, found 562.9886; HPLC analysis: 87:13 er (IB column, 25 °C, hexane/iPrOH = 80/20, 0.5 mL/min, *λ* = 254 nm), Rt (major) = 27.0 min, Rt (minor) = 32.6 min.
*(Z)-N-(3-(2-iodophenyl)-4-oxo-6-(m-tolyl)-3,4-dihydro-2H-1,3-thiazin-2-ylidene)benzamide* (**3h**), white solid, 53% yield, 27.8 mg, m.p. 206–208 °C; ${\left[\alpha \right]}_{D}^{23}$ = +24.0 (*c* = 1.0 in CHCl_3_); ^1^H NMR (400 MHz, CDCl_3_) δ 8.04 (dd, *J* = 8.0, 1.4 Hz, 1H), 7.82–7.79 (m, 2H), 7.56 (td, *J* = 7.8, 1.4 Hz, 1H), 7.51–7.45 (m, 3H), 7.39–7.31 (m, 5H), 7.21 (td, *J* = 7.8, 1.6 Hz, 1H), 6.92 (s, 1H), 2.44 (s, 3H); ^13^C NMR (101 MHz, CDCl_3_) δ 175.6, 161.8, 160.5, 151.8, 141.5, 139.9, 139.4, 135.0, 134.3, 133.1, 132.6, 130.12, 130.09, 129.7, 129.3, 129.1, 128.3, 127.4, 123.9, 115.6, 98.1, 21.4; HRMS (ESI-TOF) *m*/*z*: [M + Na]^+^ calcd for C_24_H_17_IN_2_O_2_SNa 546.9947, found 546.9944; HPLC analysis: 97:3 er (ODH column, 25 °C, hexane/iPrOH = 80/20, 0.5 mL/min, λ = 254 nm), Rt (major) = 35.4 min, Rt (minor) = 38.5 min.
*(Z)-N-(6-(3-fluorophenyl)-3-(2-iodophenyl)-4-oxo-3,4-dihydro-2H-1,3-thiazin-2-ylidene)benzamide* (**3i**), white solid, 65% yield, 34.4 mg, m.p. 162–164 °C; ${\left[\alpha \right]}_{D}^{24}$ = +10.0 (*c* = 1.0 in CHCl_3_); ^1^H NMR (400 MHz, CDCl_3_) δ 8.04 (dd, *J* = 8.0, 1.4 Hz, 1H), 7.80–7.78 (m, 2H), 7.57 (td, *J* = 7.8, 1.4 Hz, 1H), 7.51–7.46 (m, 3H), 7.43–7.39 (m, 1H), 7.35–7.31 (m, 3H), 7.25–7.20 (m, 2H), 6.92 (s, 1H); ^13^C NMR (101 MHz, CDCl_3_) δ 175.6, 163.0 (d, *J* = 250.7, Hz), 161.5, 160.0, 150.2, 141.3, 139.9, 136.4 (d, *J* = 7.9 Hz), 134.9, 133.2, 131.2 (d, *J* = 4.2 Hz), 130.19, 130.15, 129.7, 129.1, 128.3, 122.6 (d, *J* = 3.0 Hz), 118.8 (d, *J* = 21.1 Hz), 116.5, 114.0 (d, *J* = 23.9 Hz), 98.0; ^19^F NMR (377 MHz, CDCl_3_) δ -110.3; HRMS (ESI-TOF) *m*/*z*: [M + Na]^+^ calcd for C_23_H_14_FIN_2_O_2_SNa 550.9697, found 550.9687; HPLC analysis: 97:3 er (ODH column, 25 °C, hexane/iPrOH = 80/20, 0.5 mL/min, *λ* = 254 nm), Rt (minor) = 45.0 min, Rt (major) = 47.1 min.
*(Z)-N-(3-(2-iodophenyl)-4-oxo-6-(o-tolyl)-3,4-dihydro-2H-1,3-thiazin-2-ylidene)benzamide* (**3j**), white solid, 53% yield, 28.0 mg, m.p. 160–162 °C; ${\left[\alpha \right]}_{D}^{25}$ = +19.3 (*c* = 1.0 in CHCl_3_); ^1^H NMR (400 MHz, CDCl_3_) δ 8.04 (dd, *J* = 8.0, 1.4 Hz, 1H), 7.78–7.76 (m, 2H), 7.57 (td, *J* = 7.4, 1.2 Hz, 1H), 7.49–7.44 (m, 1H), 7.38–7.31 (m, 7H), 7.22 (td, *J* = 7.8, 1.6 Hz, 1H), 6.64 (s, 1H), 2.46 (s, 3H); ^13^C NMR (101 MHz, CDCl_3_) δ 175.6, 161.5, 161.2, 153.0, 141.5, 139.9 (2C), 135.6, 135.0, 134.1, 133.1, 131.1, 130.6, 130.1, 129.7, 129.2, 128.6, 128.3, 126.4, 119.2, 98.0, 19.9; HRMS (ESI-TOF) *m*/*z*: [M + Na]^+^ calcd for C_24_H_17_IN_2_O_2_SNa 546.9948, found 546.9941; UPLC analysis: 97:3 er (OD-3 column, 25 °C, hexane/iPrOH = 80/20, 0.5 mL/min, *λ* = 254 nm), Rt (minor) = 14.7 min, Rt (major) = 17.3 min.
*(Z)-N-(6-(2-fluorophenyl)-3-(2-iodophenyl)-4-oxo-3,4-dihydro-2H-1,3-thiazin-2-ylidene)benzamide* (**3k**), white solid, 48% yield, 25.8 mg, m.p. 114–116 °C; ${\left[\alpha \right]}_{D}^{25}$ = +10.2 (*c* = 1.0 in CHCl_3_); ^1^H NMR (400 MHz, CDCl_3_) δ 8.04 (dd, *J* = 8.0, 1.4 Hz, 1H), 7.79–7.77 (m, 2H), 7.63–7.45 (m, 4H), 7.36–7.29 (m, 4H), 7.25–7.20 (m, 2H), 6.95 (d, *J* = 1.0 Hz, 1H); ^13^C NMR (101 MHz, CDCl_3_) δ 175.6, 161.4, 160.6, 159.4 (d, *J* = 258.5 Hz), 146.0, 141.4, 139.9, 134.9, 133.1, 133.0, 130.1, 129.7, 129.1, 128.3, 125.0 (d, *J* = 3.6 Hz), 124.2, 123.6, 122.4 (d, *J* = 6.55 Hz), 119.8 (d, *J* = 5.6 Hz), 117.0 (d, *J* = 21.9 Hz), 98.0; ^19^F NMR (377 MHz, CDCl_3_) δ -112.4; HRMS (ESI-TOF) *m*/*z*: [M + Na]^+^ calcd for C_23_H_14_FIN_2_O_2_SNa 550.9697, found 550.9682; UPLC analysis: 91:9 er (AD-3 column, 25 °C, hexane/iPrOH = 80/20, 0.5 mL/min, *λ* = 254 nm), Rt (minor) = 35.4 min, Rt (major) = 47.6 min.
*(Z)-N-(6-(2-chlorophenyl)-3-(2-iodophenyl)-4-oxo-3,4-dihydro-2H-1,3-thiazin-2-ylidene)benzamide* (**3l**), yellow solid, 42% yield, 23.0 mg, m.p. 168–170 °C; ${\left[\alpha \right]}_{D}^{24}$ = +9.0 (*c* = 1.0 in CHCl_3_); ^1^H NMR (400 MHz, CDCl_3_) δ 8.05 (dd, *J* = 8.0, 1.4 Hz, 1H), 7.78–7.75 (m, 2H), 7.60–7.30 (m, 9H), 7.23 (td, *J* = 7.8, 1.6 Hz, 1H), 6.77 (s, 1H); ^13^C NMR (101 MHz, CDCl_3_) δ 175.6, 161.4, 161.2, 150.0, 141.4, 139.9, 134.9, 133.3, 133.1, 132.4, 131.8, 130.7, 130.24, 130.16, 130.1, 129.7, 129.1, 128.3, 127.4, 120.5, 98.0; HRMS (ESI-TOF) *m*/*z*: [M + Na]^+^ calcd for C_23_H_14_ClIN_2_O_2_SNa 566.9401, found 566.9395; HPLC analysis: 92:8 er (IB column, 25 °C, hexane/iPrOH = 80/20, 0.5 mL/min, *λ* = 254 nm), Rt (minor) = 18.4 min, Rt (major) = 21.1 min.
*(Z)-N-(3-(2-iodophenyl)-6-(naphthalen-1-yl)-4-oxo-3,4-dihydro-2H-1,3-thiazin-2-ylidene)benzamide* (**3m**), white solid, 57% yield, 32.0 mg, m.p. 185–187 °C; ${\left[\alpha \right]}_{D}^{23}$ = +24.0 (*c* = 1.0 in CHCl_3_); ^1^H NMR (400 MHz, CDCl_3_) δ 8.14–8.12 (m, 1H), 8.07 (dd, *J* = 8.0, 1.4 Hz, 1H), 7.99 (d, *J* = 8.2 Hz, 1H), 7.95–7.93 (m, 1H), 7.78–7.76 (m, 2H), 7.66–7.54 (m, 5H), 7.48–7.41 (m, 2H), 7.32 (t, *J* = 7.8 Hz, 2H), 7.23 (dd, *J* = 7.8, 1.6 Hz, 1H), 6.87 (s, 1H); ^13^C NMR (101 MHz, CDCl_3_) δ 175.6, 161.5, 161.3, 151.8, 141.5, 139.9, 135.0, 133.8, 133.1, 132.0, 131.3, 130.1(2C), 130.0, 129.7, 129.2, 128.8, 128.3, 127.7, 127.0, 126.9, 125.1, 124.5, 120.2, 98.1; HRMS (ESI-TOF) *m*/*z*: [M + Na]^+^ calcd for C_27_H_17_IN_2_O_2_SNa 582.9948, found 582.9943; HPLC analysis: 97:3 er (ODH column, 25 °C, hexane/iPrOH = 80/20, 0.5 mL/min, *λ* = 254 nm), Rt (major) = 35.4 min, Rt (minor) = 38.5 min.
*(Z)-N-(3-(2-iodophenyl)-4-oxo-6-(thiophen-2-yl)-3,4-dihydro-2H-1,3-thiazin-2-ylidene)benzamide* (**3n**), white solid, 80% yield, 41.8 mg, m.p. 144–146 °C; ${\left[\alpha \right]}_{D}^{22}$ = +7.6 (*c* = 1.0 in CHCl_3_); ^1^H NMR (400 MHz, CDCl_3_) δ 8.03 (dd, *J* = 8.0, 1.4 Hz, 1H), 7.81–7.78 (m, 2H), 7.64 (dd, *J* = 3.8, 1.2 Hz, 1H), 7.60–7.53 (m, 2H), 7.50–7.46 (m, 1H), 7.35–7.31 (m, 3H), 7.23–7.18 (m, 2H), 6.93 (s, 1H); ^13^C NMR (101 MHz, CDCl_3_) δ 175.6, 161.6, 160.0, 144.1, 141.5, 139.8, 136.9, 134.9, 133.1, 130.6, 130.13, 130.10, 129.6, 129.1, 128.9, 128.3, 113.2, 98.0; HRMS (ESI-TOF) *m*/*z*: [M + Na]^+^ calcd for C_21_H_13_IN_2_O_2_S_2_Na 538.9355, found 538.9376; HPLC analysis: 99:1 er (ODH column, 25 °C, hexane/iPrOH = 80/20, 0.5 mL/min, *λ* = 254 nm), Rt (minor) = 40.9 min, Rt (major) = 46.7 min.
*(Z)-N-(3-(2-iodophenyl)-4-oxo-6-phenyl-3,4-dihydro-2H-1,3-thiazin-2-ylidene)-4-methylbenzamide* (**4a**), yellow solid, 45% yield, 23.4 mg, m.p. 156–158 °C; ${\left[\alpha \right]}_{D}^{23}$ = +16.1 (*c* = 1.0 in CHCl_3_); ^1^H NMR (400 MHz, CDCl_3_) δ 8.03 (dd, *J* = 8.0, 1.4 Hz, 1H), 7.71–7.68 (m, 4H), 7.57–7.49 (m, 4H), 7.33 (dd, *J* = 8.0, 1.6 Hz, 1H), 7.20 (td, *J* = 7.8, 1.6 Hz, 1H), 7.13 (d, *J* = 8.0 Hz, 2H), 6.92 (s, 1H), 2.36 (s, 3H); ^13^C NMR (101 MHz, CDCl_3_) δ 175.6, 161.9, 159.9, 151.7, 144.0, 141.4, 139.8, 134.4, 132.4, 131.8, 130.2, 130.1, 129.7, 129.4, 129.1, 126.8, 115.7, 98.1, 21.8; HRMS (ESI-TOF) *m*/*z*: [M + Na]^+^ calcd for C_24_H_17_IN_2_O_2_SNa 546.9948, found 546.9949; UPLC analysis: 93:7 er (OD-3 column, 25 °C, hexane/iPrOH = 80/20, 0.5 mL/min, *λ* = 254 nm), Rt (major) = 24.1 min, Rt (minor) = 32.6 min.
*(Z)-N-(3-(2-iodophenyl)-4-oxo-6-phenyl-3,4-dihydro-2H-1,3-thiazin-2-ylidene)-3-methylbenzamide* (**4b**), white solid, 41% yield, 21.7 mg, m.p. 128–130 °C; ${\left[\alpha \right]}_{D}^{22}$ = +17.5 (*c* = 1.0 in CHCl_3_); ^1^H NMR (400 MHz, CDCl_3_) δ 8.04 (dd, *J* = 8.0, 1.4 Hz, 1H), 7.71–7.69 (m, 2H), 7.62–7.49 (m, 6H), 7.35 (dd, *J* = 8.0, 1.4 Hz, 1H), 7.30–7.28 (m, 1H), 7.24–7.19 (m, 2H), 6.93 (s, 1H), 2.30 (s, 3H); ^13^C NMR (101 MHz, CDCl_3_) δ 175.7, 161.8, 159.7, 151.5, 141.5, 139.8, 138.0, 134.9, 134.4, 133.9, 131.8, 130.9, 130.0, 129.7, 129.4, 129.2, 129.1, 128.2, 127.2, 126.8, 115.7, 98.1, 21.3; HRMS (ESI-TOF) *m*/*z*: [M + Na]^+^ calcd for C_24_H_17_IN_2_O_2_SNa 546.9948, found 546.9941; UPLC analysis: 90:10 er (AD-3 column, 25 °C, hexane/iPrOH = 80/20, 0.5 mL/min, *λ* = 254 nm), Rt (major) = 32.5 min, Rt (minor) = 36.2 min.
*(Z)-N-(3-(2-iodophenyl)-4-oxo-6-phenyl-3,4-dihydro-2H-1,3-thiazin-2-ylidene)-3-methoxybenzamide* (**4c**), white solid, 49% yield, 26.6 mg, m.p. 131–133 °C; ${\left[\alpha \right]}_{D}^{23}$ = +14.3 (*c* = 1.0 in CHCl_3_); ^1^H NMR (400 MHz, CDCl_3_) δ 8.03 (dd, *J* = 8.0, 1.4 Hz, 1H), 7.73–7.70 (m, 2H), 7.59–7.50 (m, 4H), 7.45 (dt, *J* = 7.6, 1.2 Hz, 1H), 7.35 (dd, *J* = 8.0, 1.6 Hz, 1H), 7.29–7.28 (m, 1H), 7.24–7.18 (m, 2H), 7.04–7.01 (m, 1H), 6.94 (s, 1H), 3.70 (s, 3H); ^13^C NMR (101 MHz, CDCl_3_) δ 175.4, 161.8, 160.8, 159.4, 151.7, 141.6, 139.8, 136.4, 134.3, 131.9, 130.0, 129.7, 129.5, 129.4, 129.1, 126.8, 122.6, 120.7, 115.8, 113.4, 98.1, 55.3; HRMS (ESI-TOF) *m*/*z*: [M + Na]^+^ calcd for C_24_H_17_IN_2_O_3_SNa 562.9897, found 562.9893; UPLC analysis: 90:10 er (AD-3 column, 25 °C, hexane/iPrOH = 80/20, 0.5 mL/min, *λ* = 254 nm), Rt (minor) = 34.3 min, Rt (major) = 43.5 min.
*(Z)-N-(3-(2-iodophenyl)-4-oxo-6-phenyl-3,4-dihydro-2H-1,3-thiazin-2-ylidene)-2-methylbenzamide* (**4d**), light yellow solid, 42% yield, 22.1 mg, m.p. 139–141 °C; ${\left[\alpha \right]}_{D}^{23}$ = +10.7 (*c* = 1.0 in CHCl_3_); ^1^H NMR (400 MHz, CDCl_3_) δ 8.01 (dd, *J* = 8.0, 1.4 Hz, 1H), 7.72–7.69 (m, 2H), 7.62 (dd, *J* = 8.0, 1.5 Hz, 1H), 7.58–7.49 (m, 4H), 7.34–7.30 (m, 2H), 7.20–7.15 (m, 2H), 7.09 (td, *J* = 7.6, 1.4 Hz, 1H), 6.92 (s, 1H), 2.50 (s, 3H); ^13^C NMR (101 MHz, CDCl_3_) δ 177.2, 161.9, 159.5, 151.7, 141.5, 141.2, 139.9, 134.5, 133.7, 132.3, 132.2, 131.9, 131.8, 130.1, 129.7, 129.4, 129.1, 126.9, 125.6, 115.8, 98.2, 22.3; HRMS (ESI-TOF) *m*/*z*: [M + Na]^+^ calcd for C_24_H_17_IN_2_O_2_SNa 546.9948, found 546.9937; UPLC analysis: 87:13 er (AD-3 column, 25 °C, hexane/iPrOH = 80/20, 0.5 mL/min, *λ* = 254 nm), Rt (minor) = 17.4 min, Rt (major) = 25.5 min.
*(Z)-4-fluoro-N-(3-(2-iodophenyl)-4-oxo-6-phenyl-3,4-dihydro-2H-1,3-thiazin-2-ylidene)benzamide* (**4e**), yellow solid, 49% yield, 26.1 mg, m.p. 147–149 °C; ${\left[\alpha \right]}_{D}^{23}$ = +17.0 (*c* = 1.0 in CHCl_3_); ^1^H NMR (400 MHz, CDCl3) δ 8.04 (dd, *J* = 8.0, 1.4 Hz, 1H), 7.82–7.77 (m, 2H), 7.72–7.69 (m, 2H), 7.59–7.50 (m, 4H), 7.33 (dd, *J* = 8.0, 1.6 Hz, 1H), 7.22 (td, *J* = 7.8, 1.6 Hz, 1H), 7.02–6.96 (m, 2H), 6.94 (s, 1H); ^13^C NMR (101 MHz, CDCl_3_) δ 174.4, 165.9 (d, *J* = 255.4 Hz), 161.7, 161.0, 151.6, 141.4, 139.9, 134.3, 132.7 (d, *J* = 9.5 Hz), 131.9, 131.4 (d, *J* = 2.6 Hz), 130.2, 129.7, 129.5, 129.0, 126.8, 115.8, 115. 4 (d, *J* = 21.9 Hz), 98.0; ^19^F NMR (377 MHz, CDCl_3_) δ -105.5; HRMS (ESI-TOF) *m*/*z*: [M + Na]^+^ calcd for C_23_H_14_FIN_2_O_2_SNa 550.9697, found 550.9694; UPLC analysis: 93:7 er (OD-3 column, 25 °C, hexane/iPrOH = 80/20, 0.5 mL/min, *λ* = 254 nm), Rt (major) = 21.3 min, Rt (minor) = 33.9 min.
*(Z)-4-chloro-N-(3-(2-iodophenyl)-4-oxo-6-phenyl-3,4-dihydro-2H-1,3-thiazin-2-ylidene)benzamide* (**4f**), white solid, 63% yield, 34.3 mg, m.p. 160–163 °C; ${\left[\alpha \right]}_{D}^{23}$ = +17.4 (*c* = 1.0 in CHCl_3_); ^1^H NMR (400 MHz, CDCl_3_) δ 8.03 (dd, *J* = 8.0, 1.4 Hz, 1H), 7.72–7.69 (m, 4H), 7.59–7.50 (m, 5H), 7.34–7.28 (m, 3H), 7.22 (td, *J* = 7.8, 1.6 Hz, 1H), 6.95 (s, 1H); ^13^C NMR (101 MHz, CDCl_3_) δ 174.5, 161.6, 161.2, 151.6, 141.4, 139.9, 139.5, 134.3, 133.6, 131.9, 131.5, 130.6, 129.7, 129.5, 129.1, 128.6, 126.8, 115.9, 97.9; HRMS (ESI-TOF) *m*/*z*: [M + Na]^+^ calcd for C_23_H_14_ClIN_2_O_2_SNa 566.9401, found 566.9392; UPLC analysis: 90:10 er (AD-3 column, 25 °C, hexane/iPrOH = 80/20, 0.5 mL/min, *λ* = 254 nm), Rt (major) = 36.2 min, Rt (minor) = 56.0 min.
*(Z)-4-bromo-N-(3-(2-iodophenyl)-4-oxo-6-phenyl-3,4-dihydro-2H-1,3-thiazin-2-ylidene)benzamide* (**4g**), white solid, 48% yield, 28.3 mg, m.p. 166–168 °C; ${\left[\alpha \right]}_{D}^{24}$ = +20.4 (*c* = 1.0 in CHCl_3_); ^1^H NMR (400 MHz, CDCl_3_) δ 8.03 (dd, *J* = 8.0, 1.4 Hz, 1H), 7.72–7.69 (m, 2H), 7.64–7.61 (m, 2H), 7.59–7.49 (m, 4H), 7.48–7.45 (m, 2H), 7.32 (dd, *J* = 8.0, 1.6 Hz, 1H), 7.21 (td, *J* = 7.8, 1.6 Hz, 1H), 6.94 (s, 1H); ^13^C NMR (101 MHz, CDCl_3_) δ 174.7, 161.6, 161.3, 151.6, 141.4, 139.9, 134.3, 134.0, 131.9, 131.63, 131.59, 130.2, 129.7, 129.5, 129.1, 128.3, 126.8, 115.9, 97.9; HRMS (ESI-TOF) *m*/*z*: [M + Na]^+^ calcd for C_23_H_14_BrIN_2_O_2_SNa 610.8896, found 610.8890; HPLC analysis: 90:10 er (IA column, 25 °C, hexane/iPrOH = 80/20, 0.5 mL/min, *λ* = 254 nm), Rt (major) = 37.0 min, Rt (minor) = 48.5 min.
*(Z)-3-fluoro-N-(3-(2-iodophenyl)-4-oxo-6-phenyl-3,4-dihydro-2H-1,3-thiazin-2-ylidene)benzamide* (**4h**), white solid, 51% yield, 26.7 mg, m.p. 132–134 °C; ${\left[\alpha \right]}_{D}^{25}$ = +23.6 (*c* = 1.0 in CHCl_3_); ^1^H NMR (400 MHz, CDCl_3_) δ 8.05 (dd, *J* = 8.0, 1.4 Hz, 1H), 7.72–7.69 (m, 2H), 7.60–7.50 (m, 5H), 7.43–7.39 (m, 1H), 7.34–7.27 (m, 2H), 7.25–7.14 (m, 2H), 6.95 (s, 1H); ^13^C NMR (101 MHz, CDCl_3_) δ 174.3, 162.7 (d, *J* = 247.9 Hz), 161.6, 161.5, 151.6, 141.3, 139.9, 137.4 (d, *J* = 7.3 Hz), 134.3, 131.9, 130.2, 129.9 (d, *J* = 7.9 Hz), 129.7, 129.5, 129.0, 126.8, 125.7 (d, *J* = 2.9 Hz), 120.0 (d, *J* = 21.8 Hz), 116.8 (d, *J* = 23.1 Hz), 115.9, 97.9; ^19^F NMR (377 MHz, CDCl_3_) δ -112.6; HRMS (ESI-TOF) *m*/*z*: [M + Na]^+^ calcd for C_23_H_14_FIN_2_O_2_SNa 550.9697, found 550.9694; UPLC analysis: 90:10 er (AD-3 column, 25 °C, hexane/iPrOH = 80/20, 0.5 mL/min, *λ* = 254 nm), Rt (minor) = 29.5 min, Rt (major) = 33.9 min.
*(Z)-3-chloro-N-(3-(2-iodophenyl)-4-oxo-6-phenyl-3,4-dihydro-2H-1,3-thiazin-2-ylidene)benzamide* (**4i**), white solid, 70% yield, 38.5 mg, m.p. 124–126 °C; ${\left[\alpha \right]}_{D}^{31}$ = +32.7 (*c* = 1.0 in CHCl_3_); ^1^H NMR (400 MHz, CDCl_3_) δ 8.05 (dd, *J* =7.6, 1.2 Hz, 1H), 7.71–7.66 (m, 4H), 7.60–7.49 (m, 4H), 7.44–7.41 (m, 1H), 7.33 (dd, *J* = 7.6, 1.6 Hz, 1H), 7.27 (d, *J* = 7.8 Hz, 1H), 7.25–7.21 (m, 1H), 6.95 (s, 1H); ^13^C NMR (101 MHz, CDCl_3_) δ 174.1, 161.6, 161.4, 151.5, 141.3, 139.9, 136.9, 134.4, 134.3, 132.9, 131.9, 130.4, 130.2, 129.7, 129.6, 129.5, 129.0, 128.0, 126.8, 115.9, 97.9; HRMS (ESI-TOF) *m*/*z*: [M + Na]^+^ calcd for C_23_H_14_ClIN_2_O_2_SNa 566.9401, found 566.9395; HPLC analysis: 88:12 er (IB column, 25 °C, hexane/iPrOH = 80/20, 0.5 mL/min, *λ* = 254 nm), Rt (major) = 27.6 min, Rt (minor) = 38.8 min.
*(Z)-3-bromo-N-(3-(2-iodophenyl)-4-oxo-6-phenyl-3,4-dihydro-2H-1,3-thiazin-2-ylidene)benzamide* (**4j**), white solid, 48% yield, 28.5 mg, m.p. 126–128 °C; ${\left[\alpha \right]}_{D}^{23}$ = +25.1 (*c* = 1.0 in CHCl_3_); ^1^H NMR (400 MHz, CDCl_3_) δ 8.06 (dd, *J* = 8.0, 1.4 Hz, 1H), 7.85 (t, *J* = 1.8 Hz, 1H), 7.75–7.69 (m, 3H), 7.60–7.50 (m, 5H), 7.33 (dd, *J* = 7.8, 1.4 Hz, 1H), 7.24–7.19 (m, 2H), 6.96 (s, 1H); ^13^C NMR (101 MHz, CDCl_3_) δ 174.0, 161.6, 161.5, 151.5, 141.3, 140.0, 137.0, 135.8, 134.2, 133.4, 132.0, 130.3, 129.9, 129.8, 129.5, 129.0, 128.4, 126.8, 122.5, 116.0, 97.9; HRMS (ESI-TOF) *m*/*z*: [M + Na]^+^ calcd for C_23_H_14_BrIN_2_O_2_SNa 610.8896, found 610.8890; UPLC analysis: 89:11 er (AD-3 column, 25 °C, hexane/iPrOH = 80/20, 0.5 mL/min, *λ* = 254 nm), Rt (minor) = 31.0 min, Rt (major) = 35.4 min.
*(Z)-2-fluoro-N-(3-(2-iodophenyl)-4-oxo-6-phenyl-3,4-dihydro-2H-1,3-thiazin-2-ylidene)benzamide* (**4k**), yellow solid, 56% yield, 29.6 mg, m.p. 131–133 °C; ${\left[\alpha \right]}_{D}^{40}$ = +8.4 (*c* = 1.0 in CHCl_3_); ^1^H NMR (400 MHz, CDCl_3_) δ 8.02 (dd, *J* = 8.0, 1.4 Hz, 1H), 7.72–7.69 (m, 2H), 7.59–7.49 (m, 5H), 7.46–7.40 (m, 1H), 7.32 (dd, *J* = 8.0, 1.4 Hz, 1H), 7.20 (td, *J* = 7.8, 1.6 Hz, 1H), 7.08–7.00 (m, 2H), 6.95 (s, 1H); ^13^C NMR (101 MHz, CDCl_3_) δ 173.2, 162.8 (d, *J* = 263.2 Hz), 161.7, 160.9, 151.7, 141.3, 139.8, 134.7 (d, *J* = 9.4 Hz), 134.3, 133.0, 131.9, 130.1, 129.7, 129.5, 129.1, 126.8, 123.7 (d, *J* = 4.3 Hz), 123.2 (d, *J* = 6.9 Hz), 117.0 (d, *J* = 22.2 Hz), 115.8, 98.0; ^19^F NMR (377 MHz, CDCl_3_) δ -110.2; HRMS (ESI-TOF) *m*/*z*: [M + Na]^+^ calcd for C_23_H_14_FIN_2_O_2_SNa 550.9697, found 550.9692; UPLC analysis: 88:12 er (AD-3 column, 25 °C, hexane/iPrOH = 80/20, 0.5 mL/min, *λ* = 254 nm), Rt (minor) = 28.8 min, Rt (major) = 51.5 min.
*(Z)-2-chloro-N-(3-(2-iodophenyl)-4-oxo-6-phenyl-3,4-dihydro-2H-1,3-thiazin-2-ylidene)benzamide* (**4l**), yellow solid, 40% yield, 21.6 mg, m.p. 141–143 °C; ${\left[\alpha \right]}_{D}^{28}$ = +7.1 (*c* = 1.0 in CHCl_3_); ^1^H NMR (400 MHz, CDCl_3_) δ 8.00 (dd, *J* = 8.0, 1.4 Hz, 1H), 7.73–7.70 (m, 2H), 7.59–7.50 (m, 5H), 7.39–7.29 (m, 3H), 7.20–7.12 (m, 2H), 6.95 (s, 1H); ^13^C NMR (101 MHz, CDCl_3_) δ 174.5, 161.7, 161.2, 151.7, 141.4, 139.8, 134.7, 134.3, 133.4, 133.0, 132.7, 132.0, 131.4, 130.1, 129.6, 129.5, 129.1, 126.9, 126.4, 115.9, 98.0; HRMS (ESI-TOF) *m*/*z*: [M + Na]^+^ calcd for C_23_H_14_ClIN_2_O_2_SNa 566.9401, found 566.9392; HPLC analysis: 84:16 er (IB column, 25 °C, hexane/iPrOH = 80/20, 0.5 mL/min, *λ* = 254 nm), Rt (minor) = 28.2 min, Rt (major) = 31.9 min.
*(Z)-2-bromo-N-(3-(2-iodophenyl)-4-oxo-6-phenyl-3,4-dihydro-2H-1,3-thiazin-2-ylidene)benzamide* (**4m**), yellow solid, 37% yield, 21.7 mg, m.p. 138–140 °C; ${\left[\alpha \right]}_{D}^{23}$ = +10.2 (*c* = 0.8 in CHCl_3_); ^1^H NMR (400 MHz, CDCl_3_) δ 8.00 (dd, *J* = 8.0, 1.4 Hz, 1H), 7.73–7.70 (m, 2H), 7.61–7.50 (m, 6H), 7.31 (dd, *J* = 8.0, 1.6 Hz, 1H), 7.25–7.15 (m, 3H), 6.95 (s, 1H); ^13^C NMR (101 MHz, CDCl_3_) δ 174.9, 161.7, 161.3, 151.7, 141.3, 139.8, 135.1, 134.8, 134.3, 133.1, 132.7, 131.9, 130.1, 129.7, 129.5, 129.2, 127.0, 126.9, 122.7, 115.9, 98.0; HRMS (ESI-TOF) *m*/*z*: [M + Na]^+^ calcd for C_23_H_14_BrIN_2_O_2_SNa 610.8896, found 610.8890; UPLC analysis: 85:15 er (AD-3 column, 25 °C, hexane/iPrOH = 80/20, 0.5 mL/min, *λ* = 254 nm), Rt (minor) = 25.2 min, Rt (major) = 47.3 min.

## 4. Conclusions

In summary, we have developed an NHC-catalyzed atroposelective annulation reaction for facile synthesis of axially chiral thiazine derivatives from readily available starting materials. The reaction conditions are very mild with various well-tolerated functional groups. A series of axially chiral thiazine derivatives can be readily produced using our method. Preliminary bioactive studies revealed that some of our products exhibit promising antibacterial activities against *Xanthomonas oryzae* pv. oryzae (*Xoo*) that causes rice bacterial blight. Further applications of such axially chiral thiazine derivatives in the development of novel green pesticides are in progress in our laboratories.

## Figures and Tables

**Table 1:**
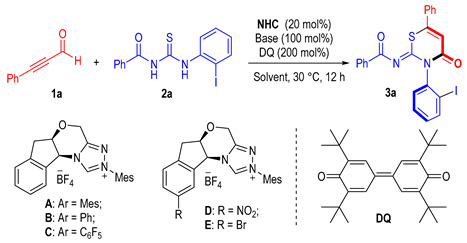
Condition optimization [a].

Entry	NHC	Base	Solvent	Yield [b] [%]	E.r. [c]
1	**A**	DMAP	furan	30	81:19
2	**B**	DMAP	furan	29	56:44
3	**C**	DMAP	furan	<5	-
4	**D**	DMAP	furan	27	86:14
5	**E**	DMAP	furan	27	84:16
6	**D**	Et_3_N	furan	12	88:12
7	**D**	Cs_2_CO_3_	furan	13	86:14
8	**D**	DABCO	THF	<5	-
9	**D**	DMAP	THF	54	91:9
10	**D**	DMAP	EtOAc	34	89:11
11	**D**	DMAP	DCM	22	77:23

[a] Unless otherwise specified, all reactions were carried out at 30 °C using **1a** (0.2 mmol), **2a** (0.1 mmol), pre-NHC catalyst (20 mol%), base (1.0 equiv), **DQ** (0.2 mmol), and 2.0 mL solvent for 12 h. [b] Isolated yield (after SiO_2_ column chromatography purification) based on **2a**. [c] The er values were determined via UPLC on chiral stationary phase.

**Table 2:** Antibacterial activities of the products.

Compounds	*Xanthomonas oryzae* pv. oryzae (*Xoo*) Inhibition Ratio (%) [a]	
	100 μg/mL	50 μg/mL
**3a**	95.4 ± 2.2	54.5 ± 0.8
**3d**	90.1 ± 1.2	50.5 ± 0.8
**3g**	94.9 ± 0.3	89.6 ± 0.5
**3h**	89.6 ± 0.6	48.6 ± 0.9
**3j**	88.3 ± 1.8	23.9 ± 2.3
**4b**	94.7 ± 0.2	87.6 ± 0.2
**4c**	92.2 ± 2.3	88.6 ± 1.3
**4d**	87.7 ± 1.5	35.2 ± 1.1
**4f**	87.2 ± 0.5	37.4 ± 2.6
**4k**	98.3 ± 0.4	46.4 ± 0.1
**4m**	83.7 ± 1.1	73.3 ± 2.1
Thiodiazole copper	78.2 ± 1.7	56.5 ± 1.3
Bismerthiazol	95.4 ± 0.3	72.3 ± 0.9

[a] All data were average data of three replicates.

## Data Availability

The data presented in this study are available in the Supporting information.

## Supplementary Materials

The following supporting information can be downloaded at: https://www.mdpi.com/article/10.3390/molecules28104052/s1. Table S1. The effects of NHCs, bases, solvents and additives.; Figure S1. Proposed Reaction Mechanism. Table S2. Antibacterial activities of the products. Reference [62] is cited in the Supplementary Materials.

## Sample Availability

Samples of the compounds are not available from the authors.
