# Supplementary material for: Carbene-Catalyzed Atroposelective Annulation for Quick Access to Axially Chiral Thiazine Derivatives
Source: Molecules. 2023 May 12;28(10):4052. doi: 10.3390/molecules28104052 (PMC10222838; doi:10.3390/molecules28104052)
Supplement: Supplementary file 1 [file molecules-28-04052-s001.zip › molecules-2385259-supplementary.pdf]

Supporting Information

Carbene-catalyzed atroposelective annulation for quick access to axially chiral thiazine derivatives

Xiaoqun Yang [1], Tingting Li [1], Jinli Chen [1], Yixian Huang [1], Tingwei Shen [1], Shiguang Li [1], Zhichao Jin [1,*] and Shi-Chao Ren [1,*]

[1] *National Key Laboratory of Green Pesticides, Key Laboratory of Green Pesticide and Agricultural Bioengineering, Ministry of Education, Guizhou University, Huaxi District, Guiyang 550025, China*

E-mail: zcjin@gzu.edu.cn; scren@gzu.edu.cn

Content

I. General information **1**

II. Preparation of substrates **2**

III. Conditions optimization for the synthesis of 3a **3**

IV. General procedure for the catalytic reactions **4**

V. Proposed reaction mechanism **5**

VI. *X*-ray crystallography of compound 3h **6**

VII. Anti-*Xoo* activities of the products *in vitro* **7**

VIII. Characterization of substrates and products **9**

IX. ^{1}H NMR, ^{13}C NMR, ^{19}F NMR and HPLC spectra **27**

I. General information

Commercially available materials and dry solvents purchased from Energy Chemical and J&K were used as received. Unless otherwise specified, all reactions were prepared using 4 mL vial. NMR spectra were measured on a Bruker ASCEND (AVANCE III HD 400 MHz) spectrometer. The chemical shift values were corrected to 7.26 ppm (^{1}H NMR) and 77.23 ppm (^{13}C NMR) for $CDCl_3$ or 3.33 ppm (^{1}H NMR) and 39.51 ppm (^{13}C NMR) for DMSO-d_6. ^{1}H NMR splitting patterns were designated as singlet (s), double (d), triplet (t), quartet (q), doublet of doublets (dd), multiplets (m), and etc. All first-order splitting patterns were assigned on the base of the appearance of the multiplet. Splitting patterns that could not be easily interpreted are designated as multiplet (m) or broad (br). High resolution mass spectrometer analysis (HRMS) was performed on Thermo Fisher Q Exactive mass spectrometer (QTOF mass analyzer). HPLC analyses were measured on Shimadzu Model SIL-20AC220V instruments. Chiralcel brand chiral columns from Daicel Chemical Industries were used with models IA, IB, ODH in 4.6 x 250 mm size. UPLC analyses were measured on Waters systems with Empower3 system controller, Waters UPLC H-Class, and Waters ACQUITY UPLC PDA detector. Chiralcel brand chiral columns from Daicel Chemical Industries were used with models AD-3, OD-3 in 3.0 x 100 mm size. The racemic products used to determine the er values were synthesized using racemic catalyst. Optical rotations were measured on an Insmark IP-digi Polarimeter in a 1 dm cuvette. The concentration (*c*) is given in g/100 mL. Melting points were measured on an uncorrected Beijing Tech Instrument X-4 digital display micro melting point apparatus. Single-Crystal *X*-Ray diffraction was recorded at Xcalibur, Eos, Gemini. Analytical thin-layer chromatography (TLC) was carried out on pre-coated silica gel plate (0.2 mm thickness). Visualization was performed using a UV lamp.

II. Preparation of substrates

Preparation of thiourea 2[1]

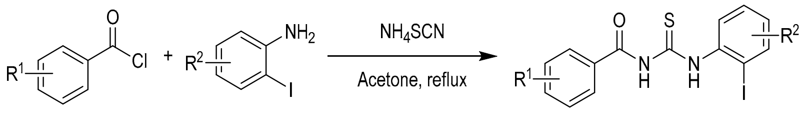

S1 **S2** **2**

To a solution of NH_4SCN (10.04 mmol, 1.1 equiv.) in acetone (20.0 mL) was added benzoyl chloride (9.13 mmol, 1.0 equiv.) over 5 min and the mixture was refluxed for 15 min. Then a solution of aniline (9.13 mmol, 1.0 equiv.) in acetone (10.0 mL) was added and the reaction was stirred at reflux for an additional 30 min. Then the reaction mixture was poured into ice water with vigorous stirring and the afforded solid was washed with water and cold acetone to give the thiourea **2** with sufficient purity, which was directly used without further purification.

References:

1. Rodl, C.B.; Vogt, D.; Kretschmer, S.B.; Ihlefeld, K.; Barzen, S.; Bruggerhoff, A.; Achenbach, J.; Proschak, E.; Steinhilber, D.; Stark, H.; Hofmann, B. Multi-dimensional target profiling of N,4-diaryl-1,3-thiazole-2-amines as potent inhibitors of eicosanoid metabolism. *Eur. J. Med. Chem.* **2014**, *84*, 302–311.

III. Conditions optimization for the synthesis of 3a

Table S1. The effects of NHCs, bases, solvents and additives. [a]

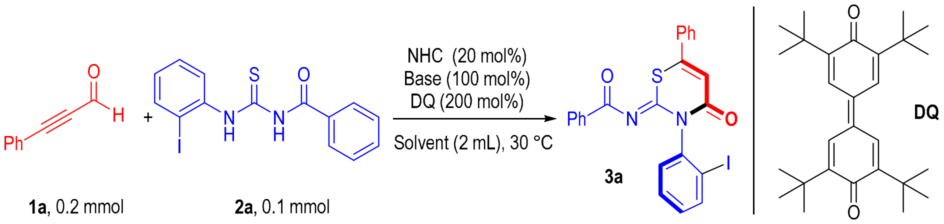

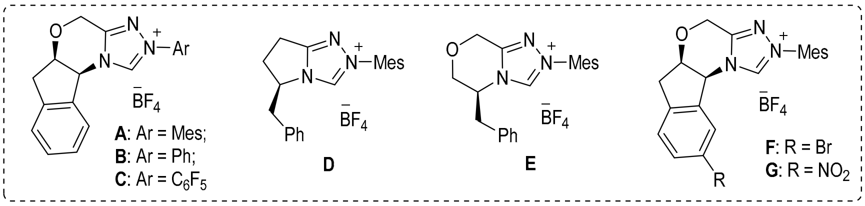

Entry	NHC	base	Solvent	Additive	Yield (%)[b]	e.r. [c]
1	**A**	DMAP	furan	-	30	81:19
2	**B**	DMAP	furan	-	29	56:44
3	**C**	DMAP	furan	-	< 5	-
4	**D**	DMAP	furan	-	28	58:42
5	**E**	DMAP	furan	-	21	71:29
6	**F**	DMAP	furan	-	27	84:16
7	**G**	DMAP	furan	-	27	86:14
8	**G**	Et_3N	furan	-	12	88:12
9	**G**	DBU	furan	-	28	86:14
10	**G**	DABCO	furan	-	< 5	-
11	**G**	Cs_2CO_3	furan	-	13	86:14
12	**G**	K_3PO_4	furan	-	20	86:14
13	**G**	$NaHCO_3$	furan	-	20	88:12
14	**G**	DMAP	THF	-	54	91:9
15	**G**	DMAP	DCM	-	22	77:23
16	**G**	DMAP	EA	-	34	89:11
17	**G**	DMAP	Toluene	-	< 5	-
18	**G**	DMAP	1,4-dioxane	-	< 5	-
19	**G**	DMAP	CH_3CN	-	< 5	-
20 [d]	**G**	DMAP	THF	$Sc(OTf)_3$ / $Cu(OTf)_3$ / $ZnCl_2$ / HOAt / $MgSO_4$ etc.	< 5	-
21 [e]	**G**	DMAP	THF	4Å MS / 5 Å MS	52	91:9

[a] Unless otherwise specified, all reactions were carried out at 30 °C using **1a** (0.2 mmol), **2a** (0.1 mmol), pre-NHC catalyst (20 mol%), base (1.0 equiv.), **DQ** (2.0 equiv.) and 2.0 mL solvent for 12 h. [b] Isolated yield (after SiO_2 column chromatography purification) based on **2a**. [c] The er value were determined by chiral HPLC. [d] Additive (100 mol%) was added. [e] 4Å MS / 4Å MS (150 mg) was added.

IV. General procedure for the catalytic reactions

General procedure for the [3 + 3] annulation Reaction

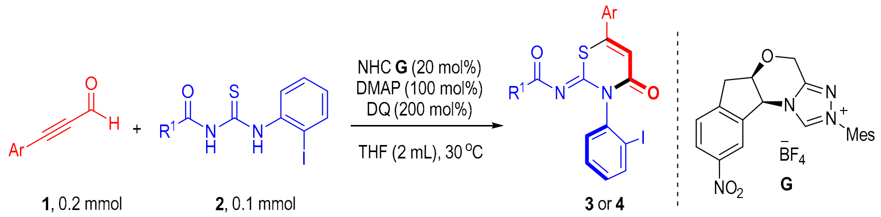

1, 0.2 mmol **2**, 0.1 mmol **3** or **4**

To a 4 mL vial equipped with a magnetic stir bar was added chiral NHC pre-catalyst **G** (0.02 mmol, 9.28 mg), DMAP (0.1 mmol, 12.22 mg), **DQ** (0.2 mmol, 81.73 mg) and substituted thiourea **2** (0.1 mmol). After that, THF (2.0 mL) and ynal **1** (0.2 mmol) was added. Then the reaction mixture was stirred at 30 °C for 12 hours. The solution was then concentrated under reduced pressure, and the residue was subjected to column chromatography (petroleum ether/ethyl acetate = 50/1 to 20/1) directly to obtain the products **3** or **4**.

General procedure for the synthesis of 3a at 2.0 mmol scale

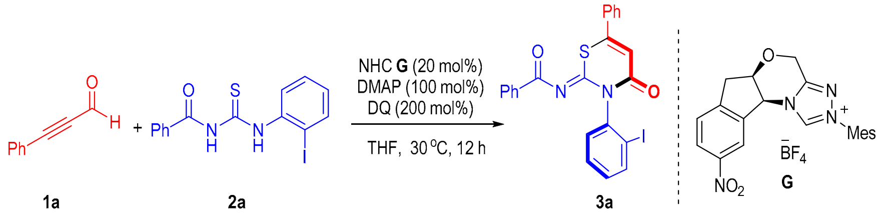

1a **2a** **3a**

To a 100 mL flame-dry flask equipped with a magnetic stir bar was added triazolium salt **G** (0.4 mmol, 185.69 mg), DMAP (2.0 mmol, 244.34 mg), **DQ** (4.0 mmol, 1.63 g) and thiourea **2a** (2.0 mmol, 764.40 mg). After that, THF (40.0 mL) and ynal **1a** (4.0 mmol, 520.51 mg) was added. Then the reaction mixture was stirred at 30 °C for 12 hours. The solution was then concentrated under reduced pressure, and the residue was subjected to column chromatography (petroleum ether/ethyl acetate = 50/1 to 20/1) directly to obtain the products **3a** (white solid, 530.65 mg, 52% yield, 91:9er).

V. Proposed reaction mechanism

A plausible reaction mechanism is illustrated in Figure S1. The deprotonated NHC catalyst **G** can add to the ynal **1a** to give the Breslow intermediate **I**, which then can be oxidized to form the acylazolium intermediate **II**. A thio-Michael addition reaction between the intermediate **II** and the deprotonated nucleophilic substrate **2a'** provides the adduct **III**. A subsequent proton transfer process of the intermediate **III** lead to the new acylazolium intermediate **IV**, which then undergoes an intramolecular lactam formation process to afford the atropisomeric thiazine product **3a**, with the regeneration of the NHC catalyst for additional catalytic cycles.

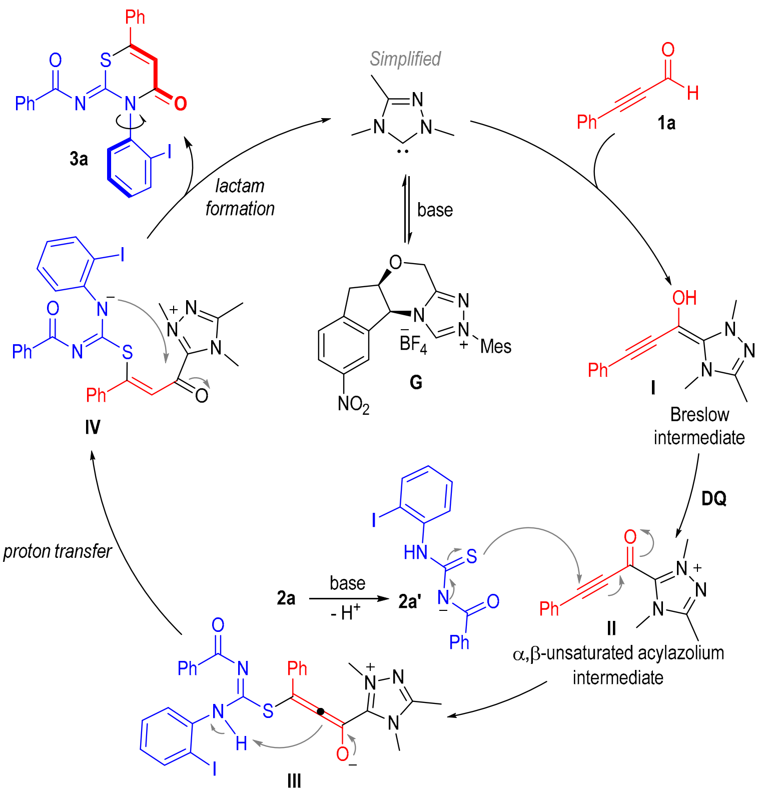

Figure S1. Proposed Reaction Mechanism.

VI. *X*-ray crystallography of compound 3h

Good quality crystal of **3h** (Colorless block crystal) was obtained by vaporization of an ethyl acetate / petroleum ether solution of compound **3h** (~100 mg). CCDC: **2255058** contains the supplementary crystallographic data for this paper. These data can be obtained free of charge from The Cambridge Crystallographic Data Centre via *www.ccdc.cam.ac.uk/data_request/cif*.

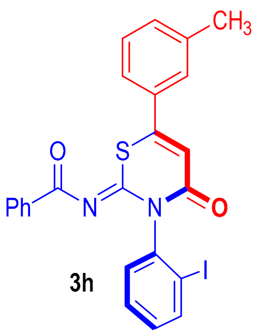

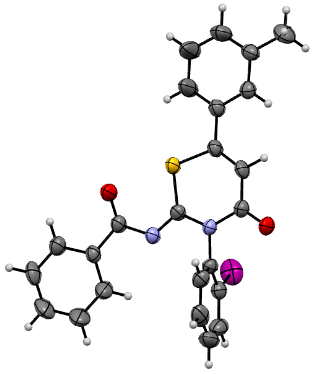

CCDC number: **2255058**

Crystal Data. $C_{24}H_{17}IN_2O_2S$, M_r = 524.36, triclinic, space group P-1 (no. 2), a = 9.0249(8) Å, b = 11.0616(9) Å, c = 11.1788(11) Å, α = 87.187(4)°, β = 80.279(3)°, γ = 75.740(2)°, V = 1066.03(17) Å^3, T = 293(2) K, Z = 2, μ(MoK_α) = 1.623 mm^{-1}, $Dcalc$ = 1.634 g/cm^3, 5168 reflections measured (4.72° ≤ 2Θ ≤ 50.04°), 3697 unique (R_{int} = 0.0253, R_{sigma} = 0.0591) which were used in all calculations. The final R_1 was 0.0516 (>2sigma(I)) and wR_2 was 0.1215 (all data).

VII. Anti-*Xoo* activities of the products *in vitro*

Antibacterial activities of the target compounds **3** or **4** against *Xanthomonas oryzae pv. oryzae* (*Xoo*) *in vitro* were evaluated by using the turbidimeter test, commercial agricultural antibacterial thiodiazole-copper was used as control. The compounds to be measured were dissolved in 150 μL of dimethylformamide and diluted with 0.1% (*V/V*) Tween-20 to prepare two concentrations of 100 μg/mL and 50 μg/mL. 1 mL of the liquid sample was added to the non-toxic nutrient broth (NB: 1.5 g of beef extract, 2.5 g of peptone, 0.5 g of yeast powder, 5.0 g of glucose and 500 mL of distilled water, pH = 7.0 – 7.2) liquid medium in 4 mL tubes. Then, 40 μL of NB containing *Xoo* was added to 5 mL of solvent NB containing the test compounds, thiodiazole-copper. The inoculated test tubes were incubated at (30 ± 1) °C under continuous shaking at 180 rpm for 38 h. The culture growth was monitored spectrophotometric ally by measuring the optical density at 595 nm (OD_{595}) and expressed as corrected turbidity. The relative inhibitory rate (*I* %) compared with a blank assay was calculated as follows:

$$\text{Relative inhibitory rate } I\ (\%) = (C_{\text{tur}} - T_{\text{tur}}) / C_{\text{tur}} \times 100$$

C_{tur}: the corrected turbidity value of bacterial growth on untreated NB;

T_{tur}: the corrected turbidity value of bacterial growth on treated NB;

I: The relative inhibitory rate.

Each experiment was repeated thrice.

Table S2. Antibacterial activities of the products.

Compound	*Xanthomonas oryzae* pv. oryzae inhibition ratio (%)	
	100 μg/mL	50 μg/mL
**3a**	95.4 ± 2.2	54.5 ± 0.8
**3b**	85.9 ± 0.2	56.6 ± 2.1
**3c**	87.6 ± 0.5	35.2 ± 1.6
**3d**	90.1 ± 1.2	50.5 ± 0.8
**3e**	31.0 ± 2.4	23.6 ± 2.9
**3f**	64.4 ± 0.2	17.2 ± 2.1
**3g**	94.9 ± 0.3	89.6 ± 0.5
**3h**	89.6 ± 0.6	48.6 ± 0.9
**3i**	54.8 ±1.1	15.3 ± 1.7
**3j**	88.3 ± 1.8	23.9 ± 2.3
**3k**	87.4 ± 0.4	50.2 ± 0.8
**3l**	85.8 ± 0.8	45.8 ± 0.8
**3m**	61.8 ± 1.7	17.5 ± 2.9
**3n**	75.4 ± 0.9	24.9 ± 1.6
**4a**	66.2 ± 0.1	27.8 ± 3.5
**4b**	94.7 ± 0.2	87.6 ± 0.2
**4c**	92.2 ± 2.3	88.6 ± 1.3
**4d**	87.7 ± 1.5	35.2 ± 1.1
**4e**	87.1 ± 0.6	67.3 ± 1.2
**4f**	87.2 ± 0.5	37.4 ± 2.6
**4g**	61.5 ± 0.1	46.1 ± 1.0
**4h**	83.2 ± 2.2	43.7 ± 0.7
**4i**	26.6 ± 9.2	14.3 ± 0.1
**4j**	62.9 ± 0.7	23.8 ± 3.2
**4k**	98.3 ± 0.4	46.4 ± 0.1
**4l**	82.5 ± 1.3	26.6 ± 2.5
**4m**	83.7 ± 1.1	73.3 ± 2.1
Thiodiazole-copper	78.2 ± 1.7	56.5 ± 1.3
Bismerthiazol	95.4 ± 0.3	72.3 ± 0.9

VIII. Characterization of substrates and products

_N_-((2-iodophenyl)carbamothioyl)benzamide (2a)

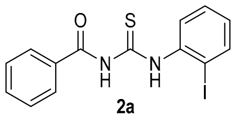

2a

Yellow solid, 82% yield, 4.5 g; m.p. 142 – 144 ºC;

^{1}H NMR (400 MHz, $CDCl_3$) δ 12.35 (s, 1H), 9.31 (s, 1H), 7.91 – 7.87 (m, 4H), 7.62 (t, *J* = 7.2 Hz, 1H), 7.51 (t, *J* = 7.6 Hz, 2H), 7.40 (t, *J* = 7.6 Hz, 1H), 7.02 (t, *J* = 7.6 Hz, 1H).

^{13}C NMR (101 MHz, $CDCl_3$) δ 179.7, 166.8, 139.9, 139.5, 133.9, 131.5, 129.2, 129.0, 128.6, 128.0, 127.8, 95.7.

HRMS (ESI-TOF) m/z: $[M+Na]^+$ Calcd for $C_{14}H_{11}IN_2OSNa$ 404.9528; Found 404.9516.

_N_-((2-iodophenyl)carbamothioyl)-4-methylbenzamide (2b)

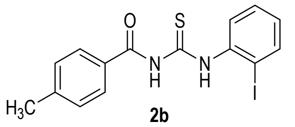

2b

White solid, 90% yield, 3.3 g; m.p. 125 – 127 ºC;

^{1}H NMR (400 MHz, $CDCl_3$) δ 12.39 (s, 1H), 9.29 (s, 1H), 7.88 (d, *J* = 8.0 Hz, 2H), 7.79 (d, *J* = 8.0 Hz, 2H), 7.39 (t, *J* = 7.6 Hz, 1H), 7.29 (d, *J* = 7.6 Hz, 2H), 7.00 (t, *J* = 7.6 Hz, 1H), 2.41 (s, 3H).

^{13}C NMR (101 MHz, $CDCl_3$) δ 179.8, 166.7, 144.9, 139.9, 139.5, 129.9 (2C), 129.0, 128.6, 128.0, 127.8, 95.7, 21.8.

HRMS (ESI-TOF) m/z: $[M+Na]^+$ Calcd for $C_{15}H_{13}IN_2OSNa$ 418.9685; Found 418.9675.

_N_-((2-iodophenyl)carbamothioyl)-3-methylbenzamide (2c)

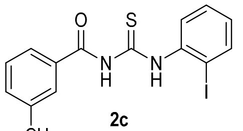

2c

White solid, 93% yield, 3.4 g; m.p. 148 – 150 ºC;

^{1}H NMR (400 MHz, $CDCl_3$) δ 12.37 (s, 1H), 9.20 (s, 1H), 7.92 (dd, *J* = 8.0, 1.6 Hz, 2H), 7.75 – 7.70 (m, 2H), 7.47 – 7.41 (m, 3H), 7.04 (td, *J* = 7.6, 1.6 Hz, 1H), 2.45 (s, 3H).

^{13}C NMR (101 MHz, $CDCl_3$) δ 179.7, 167.0, 139.9, 139.5, 139.3, 134.7, 131.5, 129.1, 129.0, 128.6, 128.3, 127.9, 124.7, 95.5, 21.4.

HRMS (ESI-TOF) m/z: $[M+Na]^+$ Calcd for $C_{15}H_{13}IN_2OSNa$ 418.9685; Found 418.9676.

_N_-((2-iodophenyl)carbamothioyl)-3-methoxybenzamide (2d)

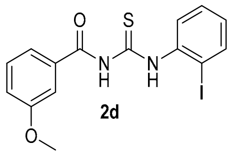

2d

White solid, 47% yield, 1.8 g; m.p. 138 – 140 ºC;

<u>^{1}H NMR</u> (400 MHz, DMSO-*d$_6$*) δ 12.45 (s, 1H), 11.80 (s, 1H), 7.95 (dd, *J* = 7.6, 1.4 Hz, 1H), 7.67 (dd, *J* = 8.0, 1.6 Hz, 1H), 7.62 – 7.59 (m, 2H), 7.46 (t, *J* = 8.0 Hz, 2H), 7.24 (dd, *J* = 8.4, 2.6 Hz, 1H), 7.10 (td, *J* = 7.6, 1.6 Hz, 1H), 3.87 (s, 3H).

<u>^{13}C NMR</u> (101 MHz, DMSO-*d$_6$*) δ 181.1, 168.6, 159.5, 140.8, 139.4, 133.7, 130.2, 129.4, 129.4, 129.1, 121.6, 120.2, 113.7, 98.0, 56.0.

<u>HRMS</u> (ESI-TOF) m/z: [M+Na]$^+$ Calcd for $C_{15}H_{13}IN_2O_2SNa$ 434.9635; Found 434.9628.

_N_-((2-iodophenyl)-2-methylbenzamide (2e)

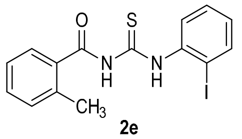

2e

Light white solid, 83% yield, 3.0 g; m.p. 187 – 189 ºC;

<u>^{1}H NMR</u> (400 MHz, DMSO-*d$_6$*) δ 12.35 (s, 1H), 11.92 (s, 1H), 7.95 (d, *J* = 6.4 Hz, 1H), 7.71 (d, *J* = 8.0 Hz, 1H), 7.53 (d, *J* = 7.4 Hz, 1H), 7.49 – 7.43 (m, 2H), 7.32 (t, *J* = 7.4 Hz, 2H), 7.12 – 7.08 (m, 1H), 2.45 (s, 3H).

<u>^{13}C NMR</u> (101 MHz, DMSO-*d$_6$*) δ 181.0, 171.2, 140.7, 139.4, 136.5, 134.4, 131.5, 131.2, 129.4, 129.3, 129.1, 128.5, 126.1, 98.0, 20.0.

<u>HRMS</u> (ESI-TOF) m/z: [M+Na]$^+$ Calcd for $C_{15}H_{13}IN_2OSNa$ 418.9685; Found 418.9679.

4-fluoro-_N_-((2-iodophenyl)carbamothioyl)benzamide (2f)

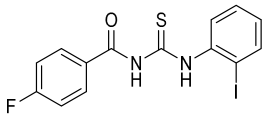

2f

Light brown solid, 81% yield, 3.0 g; m.p. 149 – 151 ºC;

<u>^{1}H NMR</u> (400 MHz, $CDCl_3$) δ 12.29 (s, 1H), 9.17 (s, 1H), 7.98 – 7.87 (m, 4H), 7.43 (td, *J* = 7.6, 1.4 Hz, 1H), 7.26 – 7.20 (m, 2H), 7.05 (td, *J* = 7.6, 1.6 Hz, 1H).

<u>^{13}C NMR</u> (101 MHz, $CDCl_3$) δ 179.6, 166.1 (d, *J* = 257.6 Hz), 165.6, 139.8, 139.5, 130.4 (d, *J* = 9.5 Hz), 129.1, 128.7, 127.9, 127.7 (d, *J* = 3.0 Hz), 116.5 (d, *J* = 22.2 Hz), 95.5.

<u>^{19}F NMR</u> (377 MHz, $CDCl_3$) δ -103.3.

<u>HRMS</u> (ESI-TOF) m/z: [M+Na]$^+$ Calcd for $C_{14}H_{10}FIN_2OSNa$ 422.9435; Found 422.9423.

4-chloro-_N_-((2-iodophenyl)carbamothioyl)benzamide (2g)

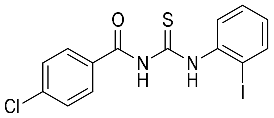

2g

White solid, 78% yield, 3.0 g; m.p. 159 – 161 ºC;

^{1}H NMR (400 MHz, $CDCl_3$) δ 12.27 (s, 1H), 9.15 (s, 1H), 7.93 – 7.87 (m, 4H), 7.55 – 7.51 (m, 2H), 7.43 (td, *J* = 7.6, 1.4 Hz, 1H), 7.05 (td, *J* = 7.6, 1.6 Hz, 1H).

^{13}C NMR (101 MHz, $CDCl_3$) δ 179.5, 165.7, 140.5, 139.7, 139.5 (2C), 129.9, 129.6, 129.1, 128.7, 127.9, 95.5.

HRMS (ESI-TOF) m/z: $[M+Na]^+$ Calcd for $C_{14}H_{10}ClIN_2OSNa$ 438.9139; Found 438.9132.

4-bromo-*N*-((2-iodophenyl)carbamothioyl)benzamide (2h)

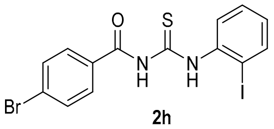

2h

White solid, 92% yield, 3.9 g; m.p. 170 – 172 ºC;

^{1}H NMR (400 MHz, DMSO-*d$_6$*) δ 12.35 (s, 1H), 11.90 (s, 1H), 7.96 – 7.93 (m, 3H), 7.77 – 7.74 (m, 2H), 7.66 (dd, *J* = 8.0, 1.6 Hz, 1H), 7.46 (td, *J* = 7.6, 1.4 Hz, 1H), 7.10 (td, *J* = 7.6, 1.6 Hz, 1H).

^{13}C NMR (101 MHz, DMSO-*d$_6$*) δ 181.0, 168.1, 140.8, 139.4, 132.0, 131.6, 131.4, 129.5, 129.3, 129.1, 127.8, 98.0.

HRMS (ESI-TOF) m/z: $[M+Na]^+$ Calcd for $C_{14}H_{10}BrIN_2OSNa$ 482.8634; Found 482.8625.

3-fluoro-*N*-((2-iodophenyl)carbamothioyl)benzamide (2i)

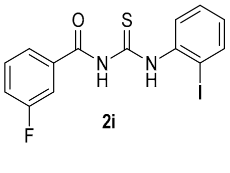

2i

Gray solid, 99% yield, 3.7 g; m.p. 145 – 147 ºC;

^{1}H NMR (400 MHz, $CDCl_3$) δ 12.24 (s, 1H), 9.16 (s, 1H), 7.92 (td, *J* = 9.2, 1.6 Hz, 2H), 7.70 – 7.66 (m, 2H), 7.58 – 7.52 (m, 1H), 7.46 – 7.35 (m, 2H), 7.05 (td, *J* = 7.6, 1.6 Hz, 1H).

^{13}C NMR (101 MHz, $CDCl_3$) δ 179.4, 165.5, 163.0 (d, *J* = 250.9 Hz), 139.7, 139.5, 133.8 (d, *J* = 7.1 Hz), 131.0 (d, *J* = 7.9 Hz), 129.1, 128.7, 127.9, 122.9 (d, *J* = 3.0 Hz), 121.0 (d, *J* = 21.2 Hz), 115.3 (d, *J* = 23.6 Hz), 95.5.

^{19}F NMR (377 MHz, $CDCl_3$) δ -109.9.

HRMS (ESI-TOF) m/z: $[M+Na]^+$ Calcd for $C_{14}H_{10}FIN_2OSNa$ 422.9434; Found 422.9428.

3-chloro-*N*-((2-iodophenyl)carbamothioyl)benzamide (2j)

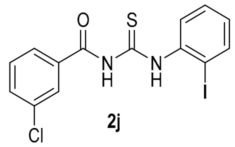

2j

White solid, 93% yield, 3.6 g; m.p. 159 – 161 ºC;

^{1}H NMR (400 MHz, $CDCl_3$) δ 12.23 (s, 1H), 9.16 (s, 1H), 7.96 – 7.90 (m, 3H), 7.80 – 7.78 (m, 1H), 7.65 – 7.62 (m, 1H), 7.52 – 7.42 (m, 2H), 7.05 (td, *J* = 7.6, 1.6 Hz, 1H).

^{13}C NMR (101 MHz, $CDCl_3$) δ 179.4, 165.4, 139.7, 139.5, 135.7, 133.9, 133.3, 130.5, 129.1, 128.7, 128.2, 127.9, 125.5, 95.5.

HRMS (ESI-TOF) m/z: $[M+Na]^+$ Calcd for $C_{14}H_{10}ClIN_2OSNa$ 438.9139; Found 438.9135.

3-bromo-*N*-((2-iodophenyl)carbamothioyl)benzamide (2k)

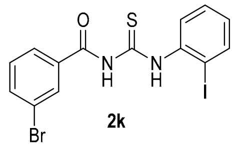

2k

Yellow solid, 76% yield, 3.2 g; m.p. 149 – 151 ºC;

^{1}H NMR (400 MHz, DMSO-*d$_6$*) δ 12.32 (s, 1H), 11.96 (s, 1H), 8.20 (t, *J* = 1.6 Hz, 1H), 8.01 – 7.97 (m, 1H), 7.94 (dd, *J* = 8.0, 1.2 Hz, 1H), 7.88 – 7.85 (m, 1H), 7.67 (dd, *J* = 7.8, 1.4 Hz, 1H), 7.53 – 7.44 (m, 2H), 7.10 (td, *J* = 7.6, 1.6 Hz, 1H).

^{13}C NMR (101 MHz, DMSO-*d$_6$*) δ 180.9, 167.5, 140.8, 139.4, 136.3, 134.6, 131.9, 131.1, 129.5, 129.3, 129.1, 128.4, 122.0, 98.0.

HRMS (ESI-TOF) m/z: $[M+Na]^+$ Calcd for $C_{14}H_{10}BrIN_2OSNa$ 482.8634; Found 482.8628.

2-fluoro-*N*-((2-iodophenyl)carbamothioyl)benzamide (2l)

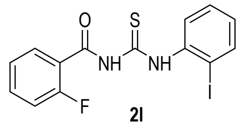

2l

Light brown solid, 92% yield, 3.4 g; m.p. 151 – 153 ºC;

^{1}H NMR (400 MHz, $CDCl_3$) δ 12.29 (s, 1H), 9.77 (d, *J* = 15.6 Hz, 1H), 8.16 (td, *J* = 7.6, 2.0 Hz, 1H), 7.94 – 7.91 (m, 2H), 7.68 – 7.62 (m, 1H), 7.43 (td, *J* = 7.6, 1.4 Hz, 1H), 7.37 (td, *J* = 7.6, 1.2 Hz, 1H), 7.28 – 7.22 (m, 1H), 7.04 (td, *J* = 7.6, 1.6 Hz, 1H).

^{13}C NMR (101 MHz, $CDCl_3$) δ 179.4, 162.9, 160.6 (d, *J* = 251.7 Hz), 139.8, 139.5, 136.0 (d, *J* = 9.6 Hz), 132.4, 129.0, 128.6, 128.0, 125.5 (d, *J* = 3.1 Hz), 118.9 (d, *J* = 9.5 Hz), 116.8 (d, *J* = 24.7 Hz), 95.5.

^{19}F NMR (377 MHz, $CDCl_3$) δ -110.9.

HRMS (ESI-TOF) m/z: $[M+Na]^+$ Calcd for $C_{14}H_{10}FIN_2OSNa$ 422.9435; Found 422.9425.

2-chloro-*N*-((2-iodophenyl)carbamothioyl)benzamide (2m)

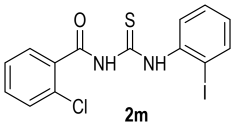

2m

White solid, 85% yield, 3.2 g; m.p. 189 – 191 °C;

<u>1H NMR</u> (400 MHz, DMSO-d_6) δ 12.16 (s, 1H), 12.12 (s, 1H), 7.96 (dd, *J* = 8.0, 1.4 Hz, 1H), 7.69 (dd, *J* = 8.0, 1.6 Hz, 1H), 7.64 (dd, *J* = 7.4, 1.6 Hz, 1H), 7.60 – 7.54 (m, 2H), 7.49 – 7.45 (m, 2H), 7.10 (td, *J* = 7.6, 1.6 Hz, 1H).

<u>13C NMR</u> (101 MHz, DMSO-d_6) δ 180.6, 168.3, 140.7, 139.4, 134.7, 132.7, 130.5, 130.1, 129.8, 129.5, 129.3, 129.1, 127.7, 98.0.

<u>HRMS</u> (ESI-TOF) m/z: $[M+Na]^+$ Calcd for $C_{14}H_{10}ClIN_2OSNa$ 438.9139; Found 438.9132.

2-bromo-*N*-((2-iodophenyl)carbamothioyl)benzamide (2n)

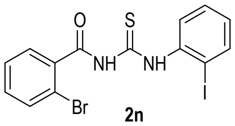

2n

Light pink solid, 82% yield, 3.5 g; m.p. 201 – 203 °C;

<u>1H NMR</u> (400 MHz, DMSO-d_6) δ 12.16 (s, 1H), 12.12 (s, 1H), 7.95 (dd, *J* = 8.0, 1.4 Hz, 1H), 7.73 (dd, *J* = 7.6, 1.4 Hz, 1H), 7.69 (dd, *J* = 8.0, 1.6 Hz, 1H), 7.60 (dd, *J* = 7.4, 2.0 Hz, 1H), 7.54 – 7.44 (m, 3H), 7.10 (td, *J* = 7.6, 1.6 Hz, 1H).

<u>13C NMR</u> (101 MHz, DMSO-d_6) δ 180.6, 169.1, 140.6, 139.4, 136.9, 133.1, 132.6, 129.6, 129.4, 129.1, 129.0, 128.0, 119.4, 97.7.

<u>HRMS</u> (ESI-TOF) m/z: $[M+Na]^+$ Calcd for $C_{14}H_{10}BrIN_2OSNa$ 482.8634; Found 482.8629.

(*Z*)-*N*-(3-(2-iodophenyl)-4-oxo-6-phenyl-3,4-dihydro-2*H*-1,3-thiazin-2-ylidene)benzamide (3a)

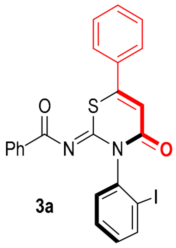

3a

White solid, 54% yield, 27.5 mg; m.p. 134 –136 °C;

<u>$[\alpha]^{23}_D$</u> = +54.3 (*c* = 0.5 in $CHCl_3$);

<u>1H NMR</u> (400 MHz, $CDCl_3$) δ 8.04 (dd, *J* = 8.0, 1.4 Hz, 1H), 7.81 – 7.78 (m, 2H), 7.72 – 7.69 (m, 2H), 7.58 – 7.45 (m, 5H), 7.35 – 7.31 (m, 3H), 7.22 (td, *J* = 7.6, 1.6 Hz, 1H), 6.94 (s, 1H).

<u>13C NMR</u> (101 MHz, $CDCl_3$) δ 175.6, 161.8, 160.4, 151.6, 141.4, 140.0, 135.0, 134.4, 133.1, 131.8, 130.13, 130.11, 129.7, 129.4, 129.1, 128.3, 126.8, 115.8, 98.0.

<u>HRMS</u> (ESI-TOF) m/z: $[M+Na]^+$ Calcd for $C_{23}H_{15}IN_2O_2SNa$ 532.9791; Found 532.9786.

<u>UPLC analysis</u>: 91:9 er (OD-3 column, 25 °C, hexane / ^{i}PrOH = 80 / 20, 0.5 mL / min, λ = 254 nm), Rt (major) = 25.9 min, Rt (minor) = 33.1 min.

(*Z*)-*N*-(3-(2-iodophenyl)-6-(4-methoxyphenyl)-4-oxo-3,4-dihydro-2*H*-1,3-thiazin-2-ylidene)benzamide (3b)

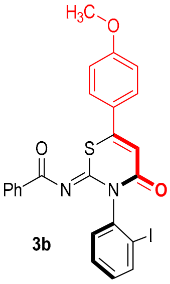

White solid, 78% yield, 42.3 mg; m.p. 118 – 120 ºC;

[α]$^{27}_{D}$ = +52.5 (*c* = 1.0 in $CHCl_3$);

^{1}H NMR (400 MHz, $CDCl_3$) δ 8.03 (dd, *J* = 8.0, 1.4 Hz, 1H), 7.82 – 7.79 (m, 2H), 7.69 – 7.65 (m, 2H), 7.55 (td, *J* = 7.6, 1.4 Hz, 1H), 7.49 – 7.45 (m, 1H), 7.35 – 7.31 (m, 3H), 7.21 (td, *J* = 7.6, 1.6 Hz, 1H), 7.03 – 7.0 (m, 2H), 6.88 (s, 1H), 3.88 (s, 3H).

^{13}C NMR (101 MHz, $CDCl_3$) δ 175.6, 162.6, 162.0, 160.4, 150.9, 141.5, 139.8, 135.1, 133.0, 130.11, 130.05, 129.6, 129.2, 128.4, 128.3, 126.5, 114.8, 113.9, 98.1, 55.6.

HRMS (ESI-TOF) m/z: $[M+Na]^+$ Calcd for $C_{24}H_{17}IN_2O_3SNa$ 562.9897; Found 562.9892.

HPLC analysis: 86:14 er (AD-3 column, 25 °C, hexane / ^{i}PrOH = 80 / 20, 0.5 mL / min, *λ* = 254 nm), Rt (major) = 35.2 min, Rt (minor) = 42.3 min.

(*Z*)-*N*-(3-(2-iodophenyl)-4-oxo-6-(*p*-tolyl)-3,4-dihydro-2*H*-1,3-thiazin-2-ylidene)benzamide (3c)

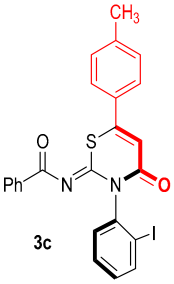

White solid, 76% yield, 40.0 mg; m.p. 156 – 158 ºC;

[α]$^{25}_{D}$ = +17.3 (*c* = 1.0 in $CHCl_3$);

^{1}H NMR (400 MHz, $CDCl_3$) δ 8.04 (dd, *J* = 8.0, 1.4 Hz, 1H), 7.78 – 7.76 (m, 2H), 7.57 (td, *J* = 7.6, 1.4 Hz, 1H), 7.49 – 7.44 (m, 1H), 7.39 – 7.29 (m, 7H), 7.22 (td, *J* = 7.6, 1.6 Hz, 1H), 6.64 (s, 1H), 2.46 (s, 3H).

^{13}C NMR (101 MHz, $CDCl_3$) δ 175.6, 161.5, 153.0, 141.5, 139.9, 135.6, 135.0, 134.1, 133.1, 131.1, 130.6, 130.1, 129.7, 129.2, 128.6, 128.3, 126.4, 119.2, 98.0, 19.9.

HRMS (ESI-TOF) m/z: $[M+Na]^+$ Calcd for $C_{24}H_{17}IN_2O_2SNa$ 546.9948; Found 546.9941.

HPLC analysis: 82:18 er (IB column, 25 °C, hexane / ^{i}PrOH = 80 / 20, 0.5 mL / min, *λ* = 254 nm), Rt (major) = 24.4 min, Rt (minor) = 25.9 min.

(*Z*)-*N*-(6-(4-fluorophenyl)-3-(2-iodophenyl)-4-oxo-3,4-dihydro-2*H*-1,3-thiazin-2-ylidene)benzamide (3d)

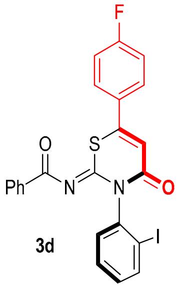

White solid, 61% yield, 32.2 mg; m.p. 216 – 218 °C;

[α]$^{24}_{D}$ = +24.0 (*c* = 1.0 in $CHCl_3$);

^{1}H NMR (400 MHz, $CDCl_3$) δ 8.04 (dd, *J* = 8.0, 1.4 Hz, 1H), 7.81 – 7.78 (m, 2H), 7.73 – 7.68 (m, 2H), 7.56 (td, *J* = 7.6, 1.4 Hz, 1H), 7.50 – 7.46 (m, 1H), 7.35 – 7.31 (m, 3H), 7.24 – 7.19 (m, 3H), 6.89 (s, 1H).

^{13}C NMR (101 MHz, $CDCl_3$) δ 175.6, 164.8 (d, *J* = 255.3 Hz), 161.7, 160.0 150.4, 141.3, 139.9, 134.9, 133.2, 130.58, 130.6 (d, *J* = 3.1 Hz), 130.1, 129.7, 129.1 (d, *J* = 4.2 Hz), 129.0, 128.3, 116.7 (d, *J* = 22.4 Hz), 115.8, 98.0.

^{19}F NMR (377 MHz, $CDCl_3$) δ -107.0.

HRMS (ESI-TOF) m/z: $[M+Na]^+$ Calcd for $C_{23}H_{14}FIN_2O_2SNa$ 550.9697; Found 550.9698.

HPLC analysis: 88:12 er (IA column, 25 °C, hexane / ^{i}PrOH = 80 / 20, 0.5 mL / min, λ = 254 nm), Rt (major) = 45.6 min, Rt (minor) = 48.8 min

(*Z*)-*N*-(6-(4-chlorophenyl)-3-(2-iodophenyl)-4-oxo-3,4-dihydro-2*H*-1,3-thiazin-2-ylidene)benzamide (3e)

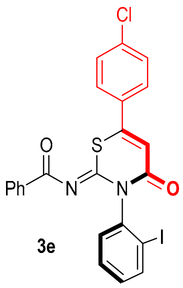

White solid, 57% yield, 31.2 mg; m.p. 180 – 182 °C;

[α]$^{25}_{D}$ = +22.6 (*c* = 1.0 in $CHCl_3$);

^{1}H NMR (400 MHz, $CDCl_3$) δ 8.04 (dd, *J* = 8.0, 1.4 Hz, 1H), 7.80 – 7.78 (m, 2H), 7.66 – 7.63 (m, 2H), 7.56 (td, *J* = 7.8, 1.4 Hz, 1H), 7.51 – 7.46 (m, 3H), 7.35 – 7.31 (m, 3H), 7.22 (td, *J* = 7.8, 1.6 Hz, 1H), 6.91 (s, 1H).

^{13}C NMR (101 MHz, $CDCl_3$) δ 175.6, 161.6, 160.0, 150.3, 141.3, 139.9, 138.2, 134.9, 133.2, 132.8, 130.2, 130.1, 129.8, 129.7, 129.1, 128.3, 128.1, 116.0, 98.0.

HRMS (ESI-TOF) m/z: $[M+Na]^+$ Calcd for $C_{23}H_{14}ClIN_2O_2SNa$ 566.9401; Found 566.9391.

HPLC analysis: 90:10 er (IB column, 25 °C, hexane / ^{i}PrOH = 80 / 20, 0.5 mL / min, λ = 254 nm), Rt (major) = 36.7 min, Rt (minor) = 40.1 min.

(*Z*)-*N*-(6-(4-bromophenyl)-3-(2-iodophenyl)-4-oxo-3,4-dihydro-2*H*-1,3-thiazin-2-ylidene)benzamide (3f)

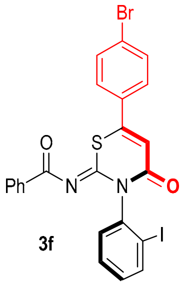

3f

White solid, 48% yield, 28.2 mg; m.p. 94 – 96 °C;

$[\alpha]^{22}_D$ = +10.0 (*c* =1.0 in $CHCl_3$);

^{1}H NMR (400 MHz, $CDCl_3$) δ 8.04 (dd, *J* = 8.0, 1.4 Hz, 1H), 7.80 – 7.78 (m, 2H), 7.67 – 7.64 (m, 2H), 7.59 – 7.54 (m, 3H), 7.50 – 7.46 (m, 1H), 7.35 – 7.31 (m, 3H), 7.22 (td, *J* = 7.6, 1.6 Hz, 1H), 6.91 (s, 1H).

^{13}C NMR (101 MHz, $CDCl_3$) δ 175.6, 161.6, 160.0, 150.4, 141.3, 139.9, 134.9, 133.3, 133.2, 132.7, 130.2, 130.1, 129.7, 129.1, 128.3, 128.2, 126.6, 116.0, 98.0.

HRMS (ESI-TOF) m/z: $[M+Na]^+$ Calcd for $C_{23}H_{14}BrIN_2O_2SNa$ 610.8896; Found 610.8893.

UPLC analysis: 92:8 er (AD-3 column, 25 °C, hexane / ^{i}PrOH = 80 / 20, 0.5 mL / min, *λ* = 254 nm), Rt (major) = 46.2 min, Rt (minor) = 49.9 min.

(*Z*)-*N*-(3-(2-iodophenyl)-6-(3-methoxyphenyl)-4-oxo-3,4-dihydro-2*H*-1,3-thiazin-2-ylidene)benzamide (3g)

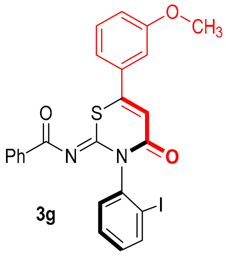

3g

White solid, 66% yield, 35.8 mg; m.p. 118 – 120 °C;

$[\alpha]^{26}_D$ = +15.5 (*c* = 1.0 in $CHCl_3$);

^{1}H NMR (400 MHz, $CDCl_3$) δ 8.04 (dd, *J* = 8.0, 1.4 Hz, 1H), 7.81 – 7.78 (m, 2H), 7.55 (dd, *J* = 7.8, 1.4 Hz, 1H), 7.50 – 7.45 (m, 1H), 7.42 (t, *J* = 8.0 Hz, 1H), 7.37 – 7.27 (m, 4H), 7.24 – 7.18 (m, 2H), 7.10 – 7.07 (m, 1H), 6.93 (s, 1H), 3.88 (s, 3H).

^{13}C NMR (101 MHz, $CDCl_3$) δ 175.5, 161.8, 160.34, 160.25, 151.6, 141.4, 139.9 (2C), 135.7, 135.0, 133.1, 130.5, 130.1, 129.7, 129.1, 128.3, 119.2, 117.7, 115.9, 111.9, 98.0, 55.6.

HRMS (ESI-TOF) m/z: $[M+Na]^+$ Calcd for $C_{24}H_{17}IN_2O_3SNa$ 562.9897; Found 562.9886.

HPLC analysis: 87:13 er (IB column, 25 °C, hexane / ^{i}PrOH = 80 / 20, 0.5 mL / min, *λ* = 254 nm), Rt (major) = 27.0 min, Rt (minor) = 32.6 min.

(*Z*)-*N*-(3-(2-iodophenyl)-4-oxo-6-(*m*-tolyl)-3,4-dihydro-2*H*-1,3-thiazin-2-ylidene)benzamide (3h)

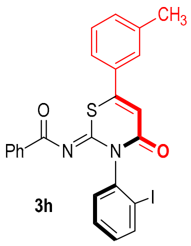

3h

White solid, 53% yield, 27.8 mg; m.p. 206 – 208 °C;

[α]$^{23}_{D}$ = +24.0 (*c* = 1.0 in $CHCl_3$);

^{1}H NMR (400 MHz, $CDCl_3$) δ 8.04 (dd, *J* = 8.0, 1.4 Hz, 1H), 7.82 – 7.79 (m, 2H), 7.56 (td, *J* = 7.8, 1.4 Hz, 1H), 7.51 – 7.45 (m, 3H), 7.39 – 7.31 (m, 5H), 7.21 (td, *J* = 7.8, 1.6 Hz, 1H), 6.92 (s, 1H), 2.44 (s, 3H).

^{13}C NMR (101 MHz, $CDCl_3$) δ 175.6, 161.8, 160.5, 151.8, 141.5, 139.9, 139.4, 135.0, 134.3, 133.1, 132.6, 130.12, 130.09, 129.7, 129.3, 129.1, 128.3, 127.4, 123.9, 115.6, 98.1, 21.4.

HRMS (ESI-TOF) m/z: $[M+Na]^+$ Calcd for $C_{24}H_{17}IN_2O_2SNa$ 546.9947; Found 546.9944.

HPLC analysis: 97:3 er (ODH column, 25 °C, hexane / ^{i}PrOH = 80 / 20, 0.5 mL / min, λ = 254 nm), Rt (major) = 35.4 min, Rt (minor) = 38.5 min.

(*Z*)-*N*-(6-(3-fluorophenyl)-3-(2-iodophenyl)-4-oxo-3,4-dihydro-2*H*-1,3-thiazin-2-ylidene)benzamide (3i)

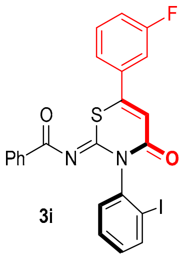

3i

White solid, 65% yield, 34.4 mg; m.p. 162 – 164 °C;

[α]$^{24}_{D}$ = +10.0 (*c* = 1.0 in $CHCl_3$);

^{1}H NMR (400 MHz, $CDCl_3$) δ 8.04 (dd, *J* = 8.0, 1.4 Hz, 1H), 7.80 – 7.78 (m, 2H), 7.57 (td, *J* = 7.8, 1.4 Hz, 1H), 7.51 – 7.46 (m, 3H), 7.43 – 7.39 (m, 1H), 7.35 – 7.31 (m, 3H), 7.25 – 7.20 (m, 2H), 6.92 (s, 1H).

^{13}C NMR (101 MHz, $CDCl_3$) δ 175.6, 163.0 (d, *J* = 250.7, Hz), 161.5, 160.0, 150.2, 141.3, 139.9, 136.4 (d, *J* = 7.9 Hz), 134.9, 133.2, 131.2 (d, *J* = 4.2 Hz), 130.19, 130.15, 129.7, 129.1, 128.3, 122.6 (d, *J* = 3.0 Hz), 118.8 (d, *J* = 21.1 Hz), 116.5, 114.0 (d, *J* = 23.9 Hz), 98.0.

^{19}F NMR (377 MHz, $CDCl_3$) δ -110.3.

HRMS (ESI-TOF) m/z: $[M+Na]^+$ Calcd for $C_{23}H_{14}FIN_2O_2SNa$ 550.9697; Found 550.9687.

HPLC analysis: 97:3 er (ODH column, 25 °C, hexane / ^{i}PrOH = 80 / 20, 0.5 mL / min, λ = 254 nm), Rt (minor) = 45.0 min, Rt (major) = 47.1 min.

(*Z*)-*N*-(3-(2-iodophenyl)-4-oxo-6-(*o*-tolyl)-3,4-dihydro-2*H*-1,3-thiazin-2-ylidene)benzamide (3j)

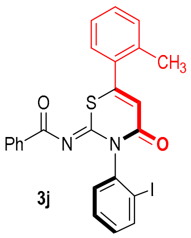

3j

White solid, 53% yield, 28.0 mg; m.p. 160 – 162 °C;

[α]$^{25}_{D}$ = +19.3 (c = 1.0 in $CHCl_3$);

^{1}H NMR (400 MHz, $CDCl_3$) δ 8.04 (dd, J = 8.0, 1.4 Hz, 1H), 7.78 – 7.76 (m, 2H), 7.57 (td, J = 7.4, 1.2 Hz, 1H), 7.49 – 7.44 (m, 1H), 7.38 – 7.31 (m, 7H), 7.22 (td, J = 7.8, 1.6 Hz, 1H), 6.64 (s, 1H), 2.46 (s, 3H).

^{13}C NMR (101 MHz, $CDCl_3$) δ 175.6, 161.5, 161.2, 153.0, 141.5, 139.9 (2C), 135.6, 135.0, 134.1, 133.1, 131.1, 130.6, 130.1, 129.7, 129.2, 128.6, 128.3, 126.4, 119.2, 98.0, 19.9.

HRMS (ESI-TOF) m/z: $[M+Na]^+$ Calcd for $C_{24}H_{17}IN_2O_2SNa$ 546.9948; Found 546.9941.

UPLC analysis: 97:3 er (OD-3 column, 25 °C, hexane / iPrOH = 80 / 20, 0.5 mL / min, λ = 254 nm), Rt (minor) = 14.7 min, Rt (major) = 17.3 min.

(*Z*)-*N*-(6-(2-fluorophenyl)-3-(2-iodophenyl)-4-oxo-3,4-dihydro-2*H*-1,3-thiazin-2-ylidene)benzamide (3k)

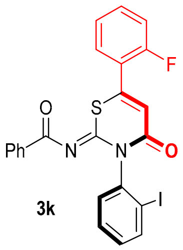

3k

White solid, 48% yield, 25.8 mg; m.p. 114 – 116 °C;

[α]$^{25}_{D}$ = +10.2 (c = 1.0 in $CHCl_3$);

^{1}H NMR (400 MHz, $CDCl_3$) δ 8.04 (dd, J = 8.0, 1.4 Hz, 1H), 7.79 – 7.77 (m, 2H), 7.63 – 7.45 (m, 4H), 7.36 – 7.29 (m, 4H), 7.25 – 7.20 (m, 2H), 6.95 (d, J = 1.0 Hz, 1H).

^{13}C NMR (101 MHz, $CDCl_3$) δ 175.6, 161.4, 160.6, 159.4 (d, J = 258.5 Hz), 146.0, 141.4, 139.9, 134.9, 133.1, 133.0, 130.1, 129.7, 129.1, 128.3, 125.0 (d, J = 3.6 Hz), 124.2, 123.6, 122.4 (d, J = 6.55 Hz), 119.8 (d, J = 5.6 Hz), 117.0 (d, J = 21.9 Hz), 98.0.

^{19}F NMR (377 MHz, $CDCl_3$) δ -112.4.

HRMS (ESI-TOF) m/z: $[M+Na]^+$ Calcd for $C_{23}H_{14}FIN_2O_2SNa$ 550.9697; Found 550.9682.

UPLC analysis: 91:9 er (AD-3 column, 25 °C, hexane / iPrOH = 80 / 20, 0.5 mL / min, λ = 254 nm), Rt (minor) = 35.4 min, Rt (major) = 47.6 min.

(*Z*)-*N*-(6-(2-chlorophenyl)-3-(2-iodophenyl)-4-oxo-3,4-dihydro-2*H*-1,3-thiazin-2-ylidene)benzamide (3l)

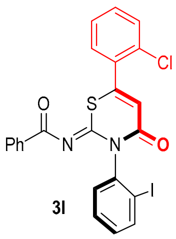

3l

Yellow solid, 42% yield, 23.0 mg; m.p. 168 – 170 °C;

[α]^{24}$_{D}$ = +9.0 (*c* = 1.0 in $CHCl_3$);

^{1}H NMR (400 MHz, $CDCl_3$) δ 8.05 (dd, *J* = 8.0, 1.4 Hz, 1H), 7.78 – 7.75 (m, 2H), 7.60 – 7.30 (m, 9H), 7.23 (td, *J* = 7.8, 1.6 Hz, 1H), 6.77 (s, 1H).

^{13}C NMR (101 MHz, $CDCl_3$) δ 175.6, 161.4, 161.2, 150.0, 141.4, 139.9, 134.9, 133.3, 133.1, 132.4, 131.8, 130.7, 130.24, 130.16, 130.1, 129.7, 129.1, 128.3, 127.4, 120.5, 98.0.

HRMS (ESI-TOF) m/z: $[M+Na]^+$ Calcd for $C_{23}H_{14}ClIN_2O_2SNa$ 566.9401; Found 566.9395.

HPLC analysis: 92:8 er (IB column, 25 °C, hexane / iPrOH = 80 / 20, 0.5 mL / min, *λ* = 254 nm), Rt (minor) = 18.4 min, Rt (major) = 21.1 min.

(*Z*)-*N*-(3-(2-iodophenyl)-6-(naphthalen-1-yl)-4-oxo-3,4-dihydro-2*H*-1,3-thiazin-2-ylidene)benzamide (3m)

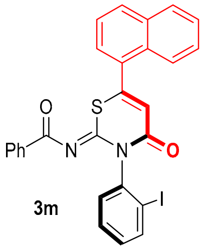

3m

White solid, 57% yield, 32.0 mg; m.p. 185 – 187 °C;

[α]^{23}$_{D}$ = +24.0 (*c* = 1.0 in $CHCl_3$);

^{1}H NMR (400 MHz, $CDCl_3$) δ 8.14 – 8.12 (m, 1H), 8.07 (dd, *J* = 8.0, 1.4 Hz, 1H), 7.99 (d, *J* = 8.2 Hz, 1H), 7.95 – 7.93 (m, 1H), 7.78 – 7.76 (m, 2H), 7.66 – 7.54 (m, 5H), 7.48 – 7.41 (m, 2H), 7.32 (t, *J* = 7.8 Hz, 2H), 7.23 (dd, *J* = 7.8, 1.6 Hz, 1H), 6.87 (s, 1H).

^{13}C NMR (101 MHz, $CDCl_3$) δ 175.6, 161.5, 161.3, 151.8, 141.5, 139.9, 135.0, 133.8, 133.1, 132.0, 131.3, 130.1 (2C), 130.0, 129.7, 129.2, 128.8, 128.3, 127.7, 127.0, 126.9, 125.1, 124.5, 120.2, 98.1.

HRMS (ESI-TOF) m/z: $[M+Na]^+$ Calcd for $C_{27}H_{17}IN_2O_2SNa$ 582.9948; Found 582.9943.

HPLC analysis: 97:3 er (ODH column, 25 °C, hexane / iPrOH = 80 / 20, 0.5 mL / min, *λ* = 254 nm), Rt (major) = 35.4 min, Rt (minor) = 38.5 min.

(*Z*)-*N*-(3-(2-iodophenyl)-4-oxo-6-(thiophen-2-yl)-3,4-dihydro-2*H*-1,3-thiazin-2-ylidene)benzamide (3n)

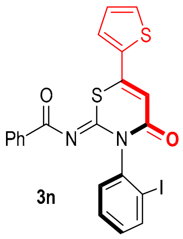

3n

White solid, 80% yield, 41.8 mg; m.p. 144 – 146 °C;

[α]^{22}$_D$ = +7.6 (*c* = 1.0 in $CHCl_3$);

^{1}H NMR (400 MHz, $CDCl_3$) δ 8.03 (dd, *J* = 8.0, 1.4 Hz, 1H), 7.81 – 7.78 (m, 2H), 7.64 (dd, *J* = 3.8, 1.2 Hz, 1H), 7.60 – 7.53 (m, 2H), 7.50 – 7.46 (m, 1H), 7.35 – 7.31 (m, 3H), 7.23 – 7.18 (m, 2H), 6.93 (s, 1H).

^{13}C NMR (101 MHz, $CDCl_3$) δ 175.6, 161.6, 160.0, 144.1, 141.5, 139.8, 136.9, 134.9, 133.1, 130.6, 130.13, 130.10, 129.6, 129.1, 128.9, 128.3, 113.2, 98.0.

HRMS (ESI-TOF) m/z: $[M+Na]^+$ Calcd for $C_{21}H_{13}IN_2O_2S_2Na$ 538.9355; Found 538.9376.

HPLC analysis: 99:1 er (ODH column, 25 °C, hexane / iPrOH = 80 / 20, 0.5 mL / min, λ = 254 nm), Rt (minor) = 40.9 min, Rt (major) = 46.7 min.

(*Z*)-*N*-(3-(2-iodophenyl)-4-oxo-6-phenyl-3,4-dihydro-2*H*-1,3-thiazin-2-ylidene)-4-methylbenzamide (4a)

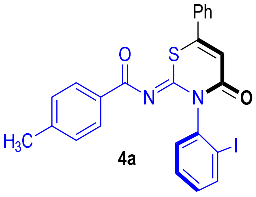

4a

Yellow solid, 45% yield, 23.4 mg; m.p. 156 – 158 °C;

[α]^{23}$_D$ = +16.1 (*c* = 1.0 in $CHCl_3$);

^{1}H NMR (400 MHz, $CDCl_3$) δ 8.03 (dd, *J* = 8.0, 1.4 Hz, 1H), 7.71 – 7.68 (m, 4H), 7.57 – 7.49 (m, 4H), 7.33 (dd, *J* = 8.0, 1.6 Hz, 1H), 7.20 (td, *J* = 7.8, 1.6 Hz, 1H), 7.13 (d, *J* = 8.0 Hz, 2H), 6.92 (s, 1H), 2.36 (s, 3H).

^{13}C NMR (101 MHz, $CDCl_3$) δ 175.6, 161.9, 159.9, 151.7, 144.0, 141.4, 139.8, 134.4, 132.4, 131.8, 130.2, 130.1, 129.7, 129.4, 129.1, 126.8, 115.7, 98.1, 21.8.

HRMS (ESI-TOF) m/z: $[M+Na]^+$ Calcd for $C_{24}H_{17}IN_2O_2SNa$ 546.9948; Found 546.9949.

UPLC analysis: 93:7 er (OD-3 column, 25 °C, hexane / iPrOH = 80 / 20, 0.5 mL / min, λ = 254 nm), Rt (major) = 24.1 min, Rt (minor) = 32.6 min.

(*Z*)-*N*-(3-(2-iodophenyl)-4-oxo-6-phenyl-3,4-dihydro-2*H*-1,3-thiazin-2-ylidene)-3-methylbenzamide (4b)

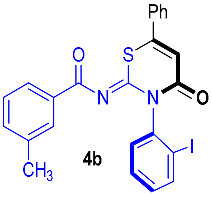

4b

White solid, 41% yield, 21.7 mg; m.p. 128 – 130 °C;

[α]^{22}$_D$ = +17.5 (*c* = 1.0 in $CHCl_3$);

^{1}H NMR (400 MHz, $CDCl_3$) δ 8.04 (dd, *J* = 8.0, 1.4 Hz, 1H), 7.71 – 7.69 (m,

2H), 7.62 – 7.49 (m, 6H), 7.35 (dd, J = 8.0, 1.4 Hz, 1H), 7.30 – 7.28 (m, 1H), 7.24 – 7.19 (m, 2H), 6.93 (s, 1H), 2.30 (s, 3H).

^{13}C NMR (101 MHz, $CDCl_3$) δ 175.7, 161.8, 159.7, 151.5, 141.5, 139.8, 138.0, 134.9, 134.4, 133.9, 131.8, 130.9, 130.0, 129.7, 129.4, 129.2, 129.1, 128.2, 127.2, 126.8, 115.7, 98.1, 21.3.

HRMS (ESI-TOF) m/z: $[M+Na]^+$ Calcd for $C_{24}H_{17}IN_2O_2SNa$ 546.9948; Found 546.9941.

UPLC analysis: 90:10 er (AD-3 column, 25 °C, hexane / iPrOH = 80 / 20, 0.5 mL / min, λ = 254 nm), Rt (major) = 32.5 min, Rt (minor) = 36.2 min.

(*Z*)-*N*-(3-(2-iodophenyl)-4-oxo-6-phenyl-3,4-dihydro-2*H*-1,3-thiazin-2-ylidene)-3-methoxybenzamide (4c)

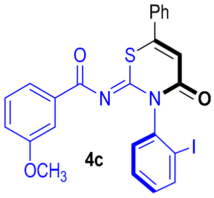

4c

White solid, 49% yield, 26.6 mg; m.p. 131 – 133 °C;

$[\alpha]^{23}_D$ = +14.3 (c = 1.0 in $CHCl_3$);

^{1}H NMR (400 MHz, $CDCl_3$) δ 8.03 (dd, J = 8.0, 1.4 Hz, 1H), 7.73 – 7.70 (m, 2H), 7.59 – 7.50 (m, 4H), 7.45 (dt, J = 7.6, 1.2 Hz, 1H), 7.35 (dd, J = 8.0, 1.6 Hz, 1H), 7.29 – 7.28 (m, 1H), 7.24 – 7.18 (m, 2H), 7.04 – 7.01 (m, 1H), 6.94 (s, 1H), 3.70 (s, 3H).

^{13}C NMR (101 MHz, $CDCl_3$) δ 175.4, 161.8, 160.8, 159.4, 151.7, 141.6, 139.8, 136.4, 134.3, 131.9, 130.0, 129.7, 129.5, 129.4, 129.1, 126.8, 122.6, 120.7, 115.8, 113.4, 98.1, 55.3.

HRMS (ESI-TOF) m/z: $[M+Na]^+$ Calcd for $C_{24}H_{17}IN_2O_3SNa$ 562.9897; Found 562.9893.

UPLC analysis: 90:10 er (AD-3 column, 25 °C, hexane / iPrOH = 80 / 20, 0.5 mL / min, λ = 254 nm), Rt (minor) = 34.3 min, Rt (major) = 43.5 min.

(*Z*)-*N*-(3-(2-iodophenyl)-4-oxo-6-phenyl-3,4-dihydro-2*H*-1,3-thiazin-2-ylidene)-2-methylbenzamide (4d)

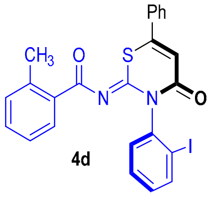

4d

Light yellow solid, 42% yield, 22.1 mg; m.p. 139 – 141 °C;

$[\alpha]^{23}_D$ = +10.7 (c = 1.0 in $CHCl_3$);

^{1}H NMR (400 MHz, $CDCl_3$) δ 8.01 (dd, J = 8.0, 1.4 Hz, 1H), 7.72 – 7.69 (m, 2H), 7.62 (dd, J = 8.0, 1.5 Hz, 1H), 7.58 – 7.49 (m, 4H), 7.34 – 7.30 (m, 2H), 7.20 – 7.15 (m, 2H), 7.09 (td, J = 7.6, 1.4 Hz, 1H), 6.92 (s, 1H), 2.50 (s, 3H).

^{13}C NMR (101 MHz, $CDCl_3$) δ 177.2, 161.9, 159.5, 151.7, 141.5, 141.2, 139.9, 134.5, 133.7, 132.3,

132.2, 131.9, 131.8, 130.1, 129.7, 129.4, 129.1, 126.9, 125.6, 115.8, 98.2, 22.3.

HRMS (ESI-TOF) m/z: $[M+Na]^+$ Calcd for $C_{24}H_{17}IN_2O_2SNa$ 546.9948; Found 546.9937.

UPLC analysis: 87:13 er (AD-3 column, 25 °C, hexane / iPrOH = 80 / 20, 0.5 mL / min, λ = 254 nm), Rt (minor) = 17.4 min, Rt (major) = 25.5 min.

(*Z*)-4-fluoro-*N*-(3-(2-iodophenyl)-4-oxo-6-phenyl-3,4-dihydro-2*H*-1,3-thiazin-2-ylidene)benzamide (4e)

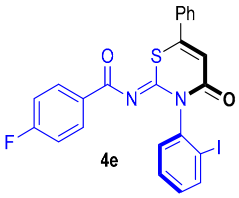

4e

Yellow solid, 49% yield, 26.1 mg; m.p. 147 – 149 °C;

$[\alpha]^{23}_D$ = +17.0 (*c* = 1.0 in $CHCl_3$);

^{1}H NMR (400 MHz, $CDCl_3$) δ 8.04 (dd, *J* = 8.0, 1.4 Hz, 1H), 7.82 – 7.77 (m, 2H), 7.72 – 7.69 (m, 2H), 7.59 – 7.50 (m, 4H), 7.33 (dd, *J* = 8.0, 1.6 Hz, 1H), 7.22 (td, *J* = 7.8, 1.6 Hz, 1H), 7.02 – 6.96 (m, 2H), 6.94 (s, 1H).

^{13}C NMR (101 MHz, $CDCl_3$) δ 174.4, 165.9 (d, *J* = 255.4 Hz), 161.7, 161.0, 151.6, 141.4, 139.9, 134.3, 132.7 (d, *J* = 9.5 Hz), 131.9, 131.4 (d, *J* = 2.6 Hz), 130.2, 129.7, 129.5, 129.0, 126.8, 115.8, 115. 4 (d, *J* = 21.9 Hz), 98.0.

^{19}F NMR (377 MHz, $CDCl_3$) δ -105.5.

HRMS (ESI-TOF) m/z: $[M+Na]^+$ Calcd for $C_{23}H_{14}FIN_2O_2SNa$ 550.9697; Found 550.9694.

UPLC analysis: 93:7 er (OD-3 column, 25 °C, hexane / iPrOH = 80 / 20, 0.5 mL / min, λ = 254 nm), Rt (major) = 21.3 min, Rt (minor) = 33.9 min.

(*Z*)-4-chloro-*N*-(3-(2-iodophenyl)-4-oxo-6-phenyl-3,4-dihydro-2*H*-1,3-thiazin-2-ylidene)benzamide (4f)

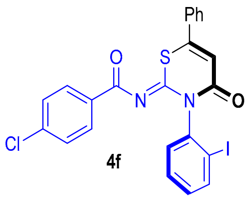

4f

White solid, 63% yield, 34.3 mg; m.p. 160 – 163 °C;

$[\alpha]^{23}_D$ = +17.4 (*c* = 1.0 in $CHCl_3$);

^{1}H NMR (400 MHz, $CDCl_3$) δ 8.03 (dd, *J* = 8.0, 1.4 Hz, 1H), 7.72 – 7.69 (m, 4H), 7.59 – 7.50 (m, 5H), 7.34 – 7.28 (m, 3H), 7.22 (td, *J* = 7.8, 1.6 Hz, 1H), 6.95 (s, 1H).

^{13}C NMR (101 MHz, $CDCl_3$) δ 174.5, 161.6, 161.2, 151.6, 141.4, 139.9, 139.5, 134.3, 133.6, 131.9, 131.5, 130.6, 129.7, 129.5, 129.1, 128.6, 126.8, 115.9, 97.9.

HRMS (ESI-TOF) m/z: $[M+Na]^+$ Calcd for $C_{23}H_{14}ClIN_2O_2SNa$ 566.9401; Found 566.9392.

UPLC analysis: 90:10 er (AD-3 column, 25 °C, hexane / iPrOH = 80 / 20, 0.5 mL / min, λ = 254 nm), Rt (major) = 36.2 min, Rt (minor) = 56.0 min.

(*Z*)-4-bromo-*N*-(3-(2-iodophenyl)-4-oxo-6-phenyl-3,4-dihydro-2*H*-1,3-thiazin-2-ylidene)benzamide (4g)

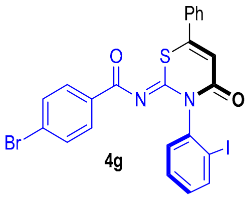

White solid, 48% yield, 28.3 mg; m.p. 166 – 168 °C;

$[\alpha]^{24}_D$ = +20.4 (c = 1.0 in $CHCl_3$);

^{1}H NMR (400 MHz, $CDCl_3$) δ 8.03 (dd, J = 8.0, 1.4 Hz, 1H), 7.72 – 7.69 (m, 2H), 7.64 – 7.61 (m, 2H), 7.59 – 7.49 (m, 4H), 7.48 – 7.45 (m, 2H), 7.32 (dd, J = 8.0, 1.6 Hz, 1H), 7.21 (td, J = 7.8, 1.6 Hz, 1H), 6.94 (s, 1H).

^{13}C NMR (101 MHz, $CDCl_3$) δ 174.7, 161.6, 161.3, 151.6, 141.4, 139.9, 134.3, 134.0, 131.9, 131.63, 131.59, 130.2, 129.7, 129.5, 129.1, 128.3, 126.8, 115.9, 97.9.

HRMS (ESI-TOF) m/z: $[M+Na]^+$ Calcd for $C_{23}H_{14}BrIN_2O_2SNa$ 610.8896; Found 610.8890.

HPLC analysis: 90:10 er (IA column, 25 °C, hexane / iPrOH = 80 / 20, 0.5 mL / min, λ = 254 nm), Rt (major) = 37.0 min, Rt (minor) = 48.5 min.

(*Z*)-3-fluoro-*N*-(3-(2-iodophenyl)-4-oxo-6-phenyl-3,4-dihydro-2*H*-1,3-thiazin-2-ylidene)benzamide (4h)

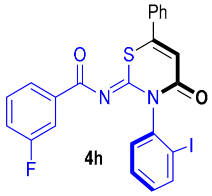

White solid, 51% yield, 26.7 mg; m.p. 132 – 134 °C;

$[\alpha]^{25}_D$ = +23.6 (c = 1.0 in $CHCl_3$);

^{1}H NMR (400 MHz, $CDCl_3$) δ 8.05 (dd, J = 8.0, 1.4 Hz, 1H), 7.72 – 7.69 (m, 2H), 7.60 – 7.50 (m, 5H), 7.43 – 7.39 (m, 1H), 7.34 – 7.27 (m, 2H), 7.25 – 7.14 (m, 2H), 6.95 (s, 1H).

^{13}C NMR (101 MHz, $CDCl_3$) δ 174.3, 162.7 (d, J = 247.9 Hz), 161.6, 161.5, 151.6, 141.3, 139.9, 137.4 (d, J = 7.3 Hz), 134.3, 131.9, 130.2, 129.9 (d, J = 7.9 Hz), 129.7, 129.5, 129.0, 126.8, 125.7 (d, J = 2.9 Hz), 120.0 (d, J = 21.8 Hz), 116.8 (d, J = 23.1 Hz), 115.9, 97.9.

^{19}F NMR (377 MHz, $CDCl_3$) δ -112.6.

HRMS (ESI-TOF) m/z: $[M+Na]^+$ Calcd for $C_{23}H_{14}FIN_2O_2SNa$ 550.9697; Found 550.9694.

UPLC analysis: 90:10 er (AD-3 column, 25 °C, hexane / iPrOH = 80 / 20, 0.5 mL / min, λ = 254 nm), Rt (minor) = 29.5 min, Rt (major) = 33.9 min.

(*Z*)-3-chloro-*N*-(3-(2-iodophenyl)-4-oxo-6-phenyl-3,4-dihydro-2*H*-1,3-thiazin-2-ylidene)benzamide (4i)

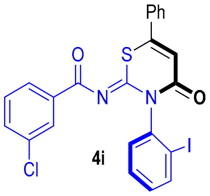

4i

White solid, 70% yield, 38.5 mg; m.p. 124 – 126 °C;

$[\alpha]^{31}_{D}$ = +32.7 (c = 1.0 in $CHCl_3$);

^{1}H NMR (400 MHz, $CDCl_3$) δ 8.05 (dd, J =7.6, 1.2 Hz, 1H), 7.71 – 7.66 (m, 4H), 7.60 – 7.49 (m, 4H), 7.44 – 7.41 (m, 1H), 7.33 (dd, J = 7.6, 1.6 Hz, 1H), 7.27 (d, J = 7.8 Hz, 1H), 7.25 – 7.21 (m, 1H), 6.95 (s, 1H).

^{13}C NMR (101 MHz, $CDCl_3$) δ 174.1, 161.6, 161.4, 151.5, 141.3, 139.9, 136.9, 134.4, 134.3, 132.9, 131.9, 130.4, 130.2, 129.7, 129.6, 129.5, 129.0, 128.0, 126.8, 115.9, 97.9.

HRMS (ESI-TOF) m/z: $[M+Na]^+$ Calcd for $C_{23}H_{14}ClIN_2O_2SNa$ 566.9401; Found 566.9395.

HPLC analysis: 88:12 er (IB column, 25 °C, hexane / iPrOH = 80 / 20, 0.5 mL / min, λ = 254 nm), Rt (major) = 27.6 min, Rt (minor) = 38.8 min.

(*Z*)-3-bromo-*N*-(3-(2-iodophenyl)-4-oxo-6-phenyl-3,4-dihydro-2*H*-1,3-thiazin-2-ylidene)benzamide (4j)

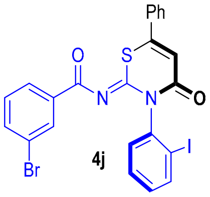

4j

White solid, 48% yield, 28.5 mg; m.p. 126 – 128 °C;

$[\alpha]^{23}_{D}$ = +25.1 (c = 1.0 in $CHCl_3$);

^{1}H NMR (400 MHz, $CDCl_3$) δ 8.06 (dd, J = 8.0, 1.4 Hz, 1H), 7.85 (t, J = 1.8 Hz, 1H), 7.75 – 7.69 (m, 3H), 7.60 – 7.50 (m, 5H), 7.33 (dd, J = 7.8, 1.4 Hz, 1H), 7.24 – 7.19 (m, 2H), 6.96 (s, 1H).

^{13}C NMR (101 MHz, $CDCl_3$) δ 174.0, 161.6, 161.5, 151.5, 141.3, 140.0, 137.0, 135.8, 134.2, 133.4, 132.0, 130.3, 129.9, 129.8, 129.5, 129.0, 128.4, 126.8, 122.5, 116.0, 97.9.

HRMS (ESI-TOF) m/z: $[M+Na]^+$ Calcd for $C_{23}H_{14}BrIN_2O_2SNa$ 610.8896; Found 610.8890.

UPLC analysis: 89:11 er (AD-3 column, 25 °C, hexane / iPrOH = 80 / 20, 0.5 mL / min, λ = 254 nm), Rt (minor) = 31.0 min, Rt (major) = 35.4 min.

(*Z*)-2-fluoro-*N*-(3-(2-iodophenyl)-4-oxo-6-phenyl-3,4-dihydro-2*H*-1,3-thiazin-2-ylidene)benzamide (4k)

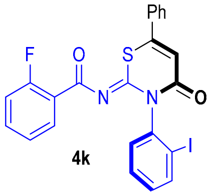

Yellow solid, 56% yield, 29.6 mg; m.p. 131 – 133 °C;

[α]$^{40}_{D}$ = +8.4 (*c* = 1.0 in $CHCl_3$);

^{1}H NMR (400 MHz, $CDCl_3$) δ 8.02 (dd, *J* = 8.0, 1.4 Hz, 1H), 7.72 – 7.69 (m, 2H), 7.59 – 7.49 (m, 5H), 7.46 – 7.40 (m, 1H), 7.32 (dd, *J* = 8.0, 1.4 Hz, 1H), 7.20 (td, *J* = 7.8, 1.6 Hz, 1H), 7.08 – 7.00 (m, 2H), 6.95 (s, 1H).

^{13}C NMR (101 MHz, $CDCl_3$) δ 173.2, 162.8 (d, *J* = 263.2 Hz), 161.7, 160.9, 151.7, 141.3, 139.8, 134.7 (d, *J* = 9.4 Hz), 134.3, 133.0, 131.9, 130.1, 129.7, 129.5, 129.1, 126.8, 123.7 (d, *J* = 4.3 Hz), 123.2 (d, *J* = 6.9 Hz), 117.0 (d, *J* = 22.2 Hz), 115.8, 98.0.

^{19}F NMR (377 MHz, $CDCl_3$) δ -110.2.

HRMS (ESI-TOF) m/z: $[M+Na]^+$ Calcd for $C_{23}H_{14}FIN_2O_2SNa$ 550.9697; Found 550.9692.

UPLC analysis: 88:12 er (AD-3 column, 25 °C, hexane / iPrOH = 80 / 20, 0.5 mL / min, λ = 254 nm), Rt (minor) = 28.8 min, Rt (major) = 51.5 min.

(*Z*)-2-chloro-*N*-(3-(2-iodophenyl)-4-oxo-6-phenyl-3,4-dihydro-2*H*-1,3-thiazin-2-ylidene)benzamide (4l)

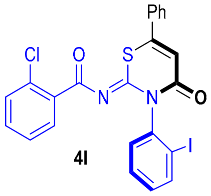

Yellow solid, 40% yield, 21.6 mg; m.p. 141 – 143 °C;

[α]$^{28}_{D}$ = +7.1 (*c* = 1.0 in $CHCl_3$);

^{1}H NMR (400 MHz, $CDCl_3$) δ 8.00 (dd, *J* = 8.0, 1.4 Hz, 1H), 7.73 – 7.70 (m, 2H), 7.59 – 7.50 (m, 5H), 7.39 – 7.29 (m, 3H), 7.20 – 7.12 (m, 2H), 6.95 (s, 1H).

^{13}C NMR (101 MHz, $CDCl_3$) δ 174.5, 161.7, 161.2, 151.7, 141.4, 139.8, 134.7, 134.3, 133.4, 133.0, 132.7, 132.0, 131.4, 130.1, 129.6, 129.5, 129.1, 126.9, 126.4, 115.9, 98.0.

HRMS (ESI-TOF) m/z: $[M+Na]^+$ Calcd for $C_{23}H_{14}ClIN_2O_2SNa$ 566.9401; Found 566.9392.

HPLC analysis: 84:16 er (IB column, 25 °C, hexane / iPrOH = 80 / 20, 0.5 mL / min, λ = 254 nm), Rt (minor) = 28.2 min, Rt (major) = 31.9 min.

(*Z*)-2-bromo-*N*-(3-(2-iodophenyl)-4-oxo-6-phenyl-3,4-dihydro-2*H*-1,3-thiazin-2-

ylidene)benzamide (4m)

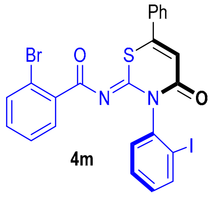

4m

Yellow solid, 37% yield, 21.7 mg; m.p. 138 – 140 °C;

[α]^{23}$_D$ = +10.2 (*c* = 1.0 in $CHCl_3$);

^{1}H NMR (400 MHz, $CDCl_3$) δ 8.00 (dd, *J* = 8.0, 1.4 Hz, 1H), 7.73 – 7.70 (m, 2H), 7.61 – 7.50 (m, 6H), 7.31 (dd, *J* = 8.0, 1.6 Hz, 1H), 7.25 – 7.15 (m, 3H), 6.95 (s, 1H).

^{13}C NMR (101 MHz, $CDCl_3$) δ 174.9, 161.7, 161.3, 151.7, 141.3, 139.8, 135.1, 134.8, 134.3, 133.1, 132.7, 131.9, 130.1, 129.7, 129.5, 129.2, 127.0, 126.9, 122.7, 115.9, 98.0.

HRMS (ESI-TOF) m/z: $[M+Na]^+$ Calcd for $C_{23}H_{14}BrIN_2O_2SNa$ 610.8896; Found 610.8890.

UPLC analysis: 85:15 er (AD-3 column, 25 °C, hexane / iPrOH = 80 / 20, 0.5 mL / min, λ = 254 nm), Rt (minor) = 25.2 min, Rt (major) = 47.3 min.

IX. ^{1}H NMR, ^{13}C NMR, ^{19}F NMR and HPLC spectra

N-((2-iodophenyl)carbamothioyl)benzamide (2a)

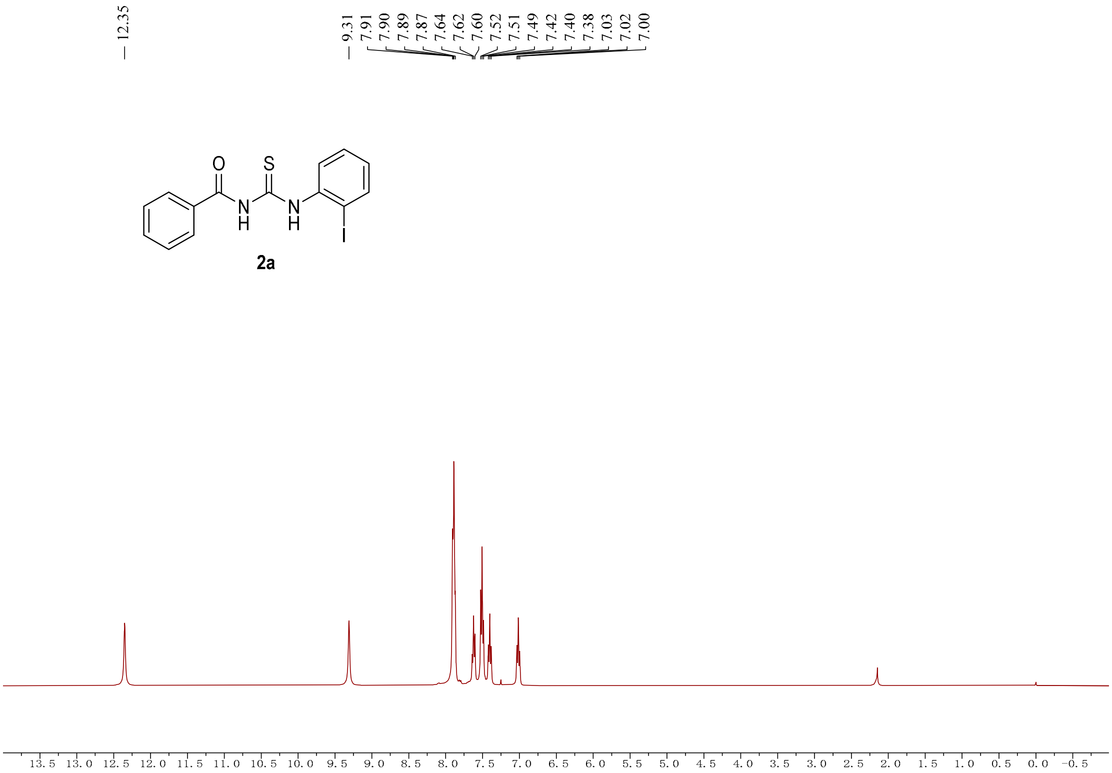

2a

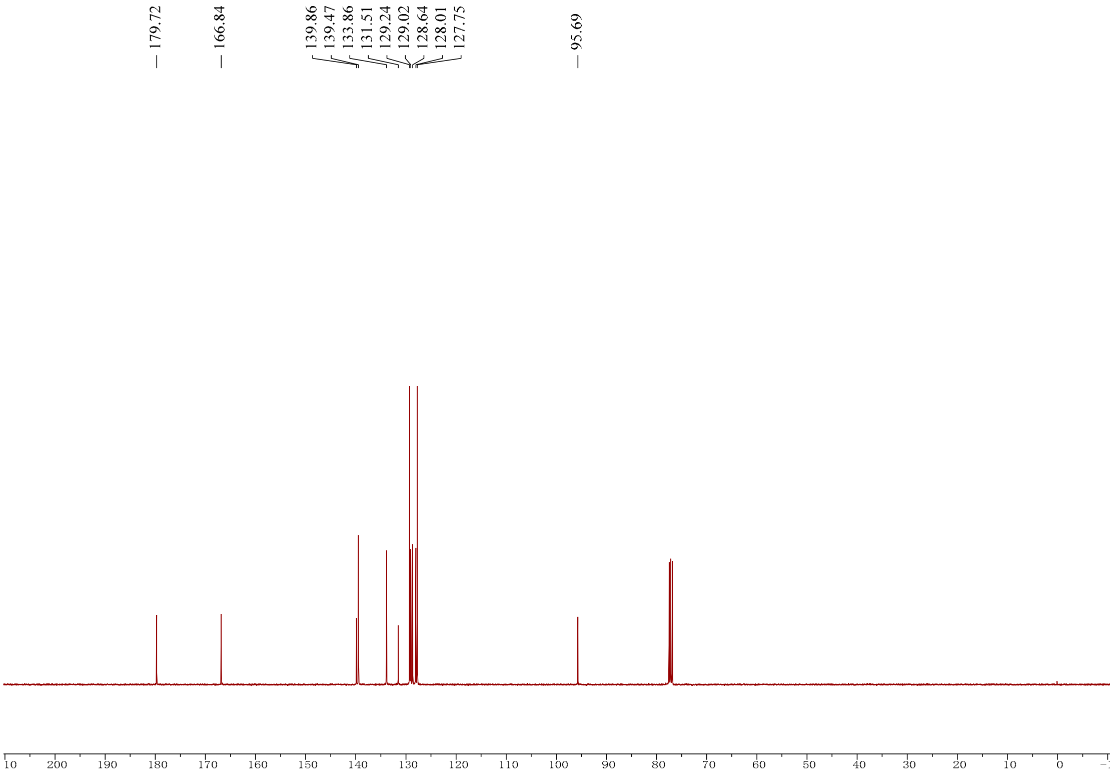

N-((2-iodophenyl)carbamothioyl)-4-methylbenzamide (2b)

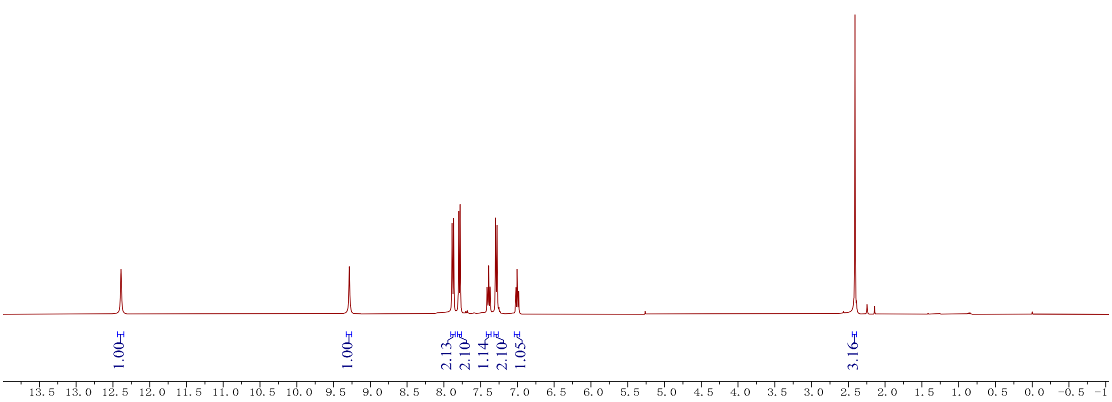

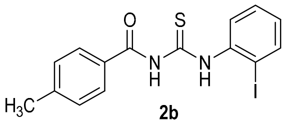

2b

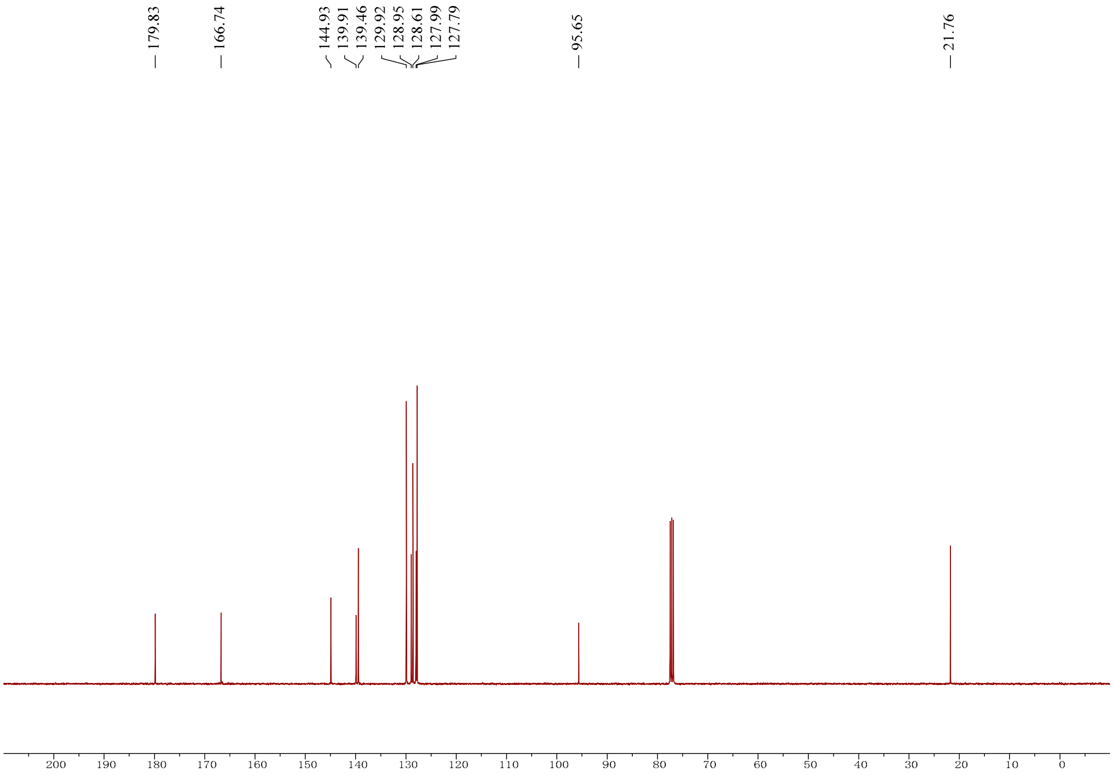

N-((2-iodophenyl)carbamothioyl)-3-methylbenzamide (2c)

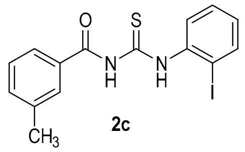

2c

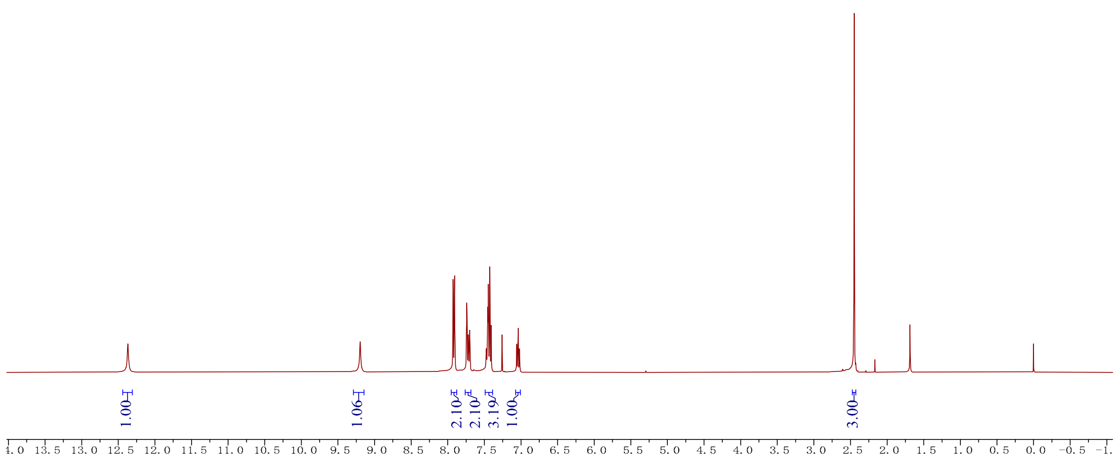

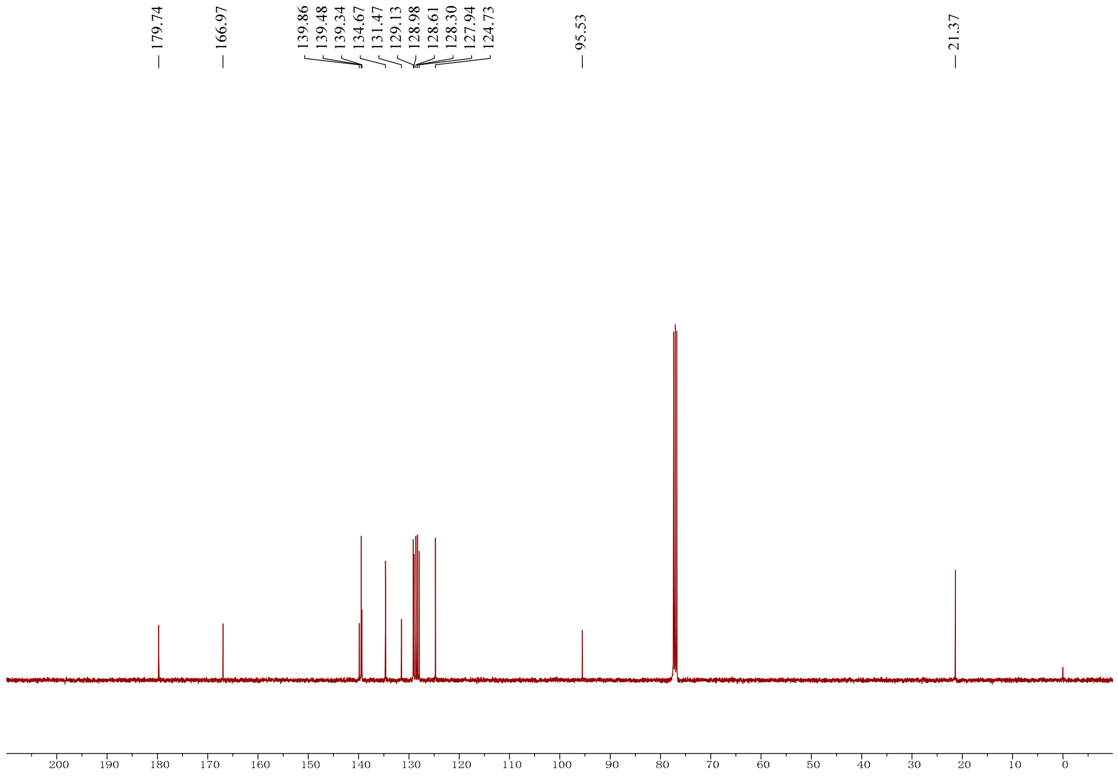

N-((2-iodophenyl)carbamothioyl)-3-methoxybenzamide (2d)

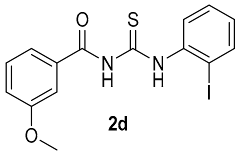

2d

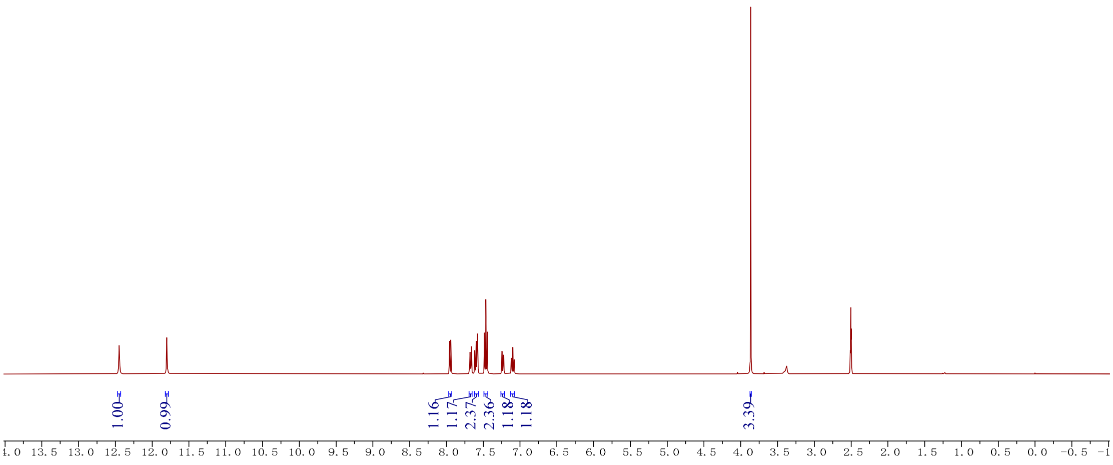

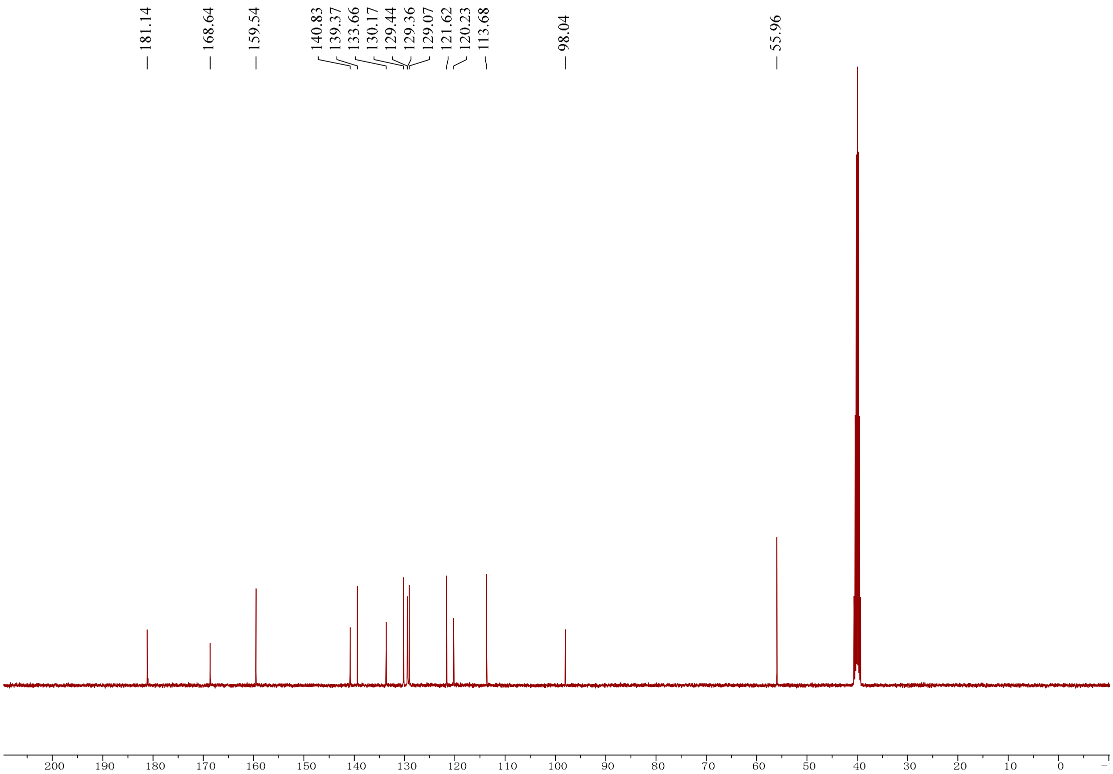

N-((2-iodophenyl)carbamothioyl)-2-methylbenzamide (2e)

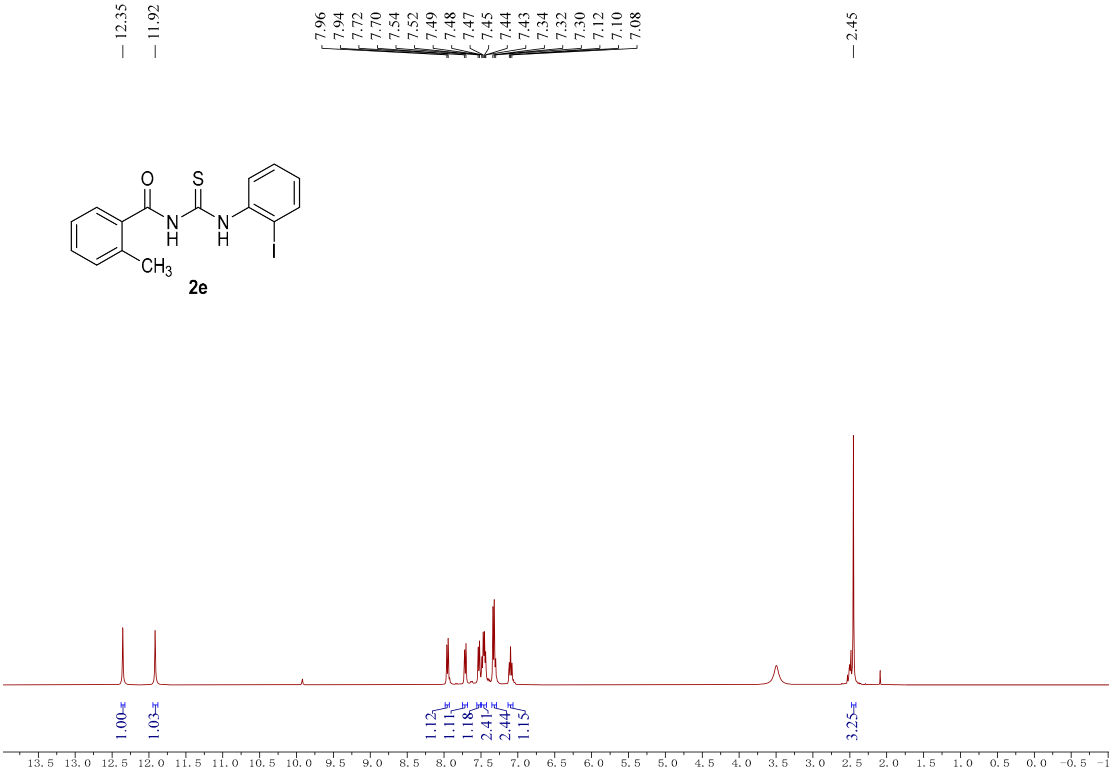

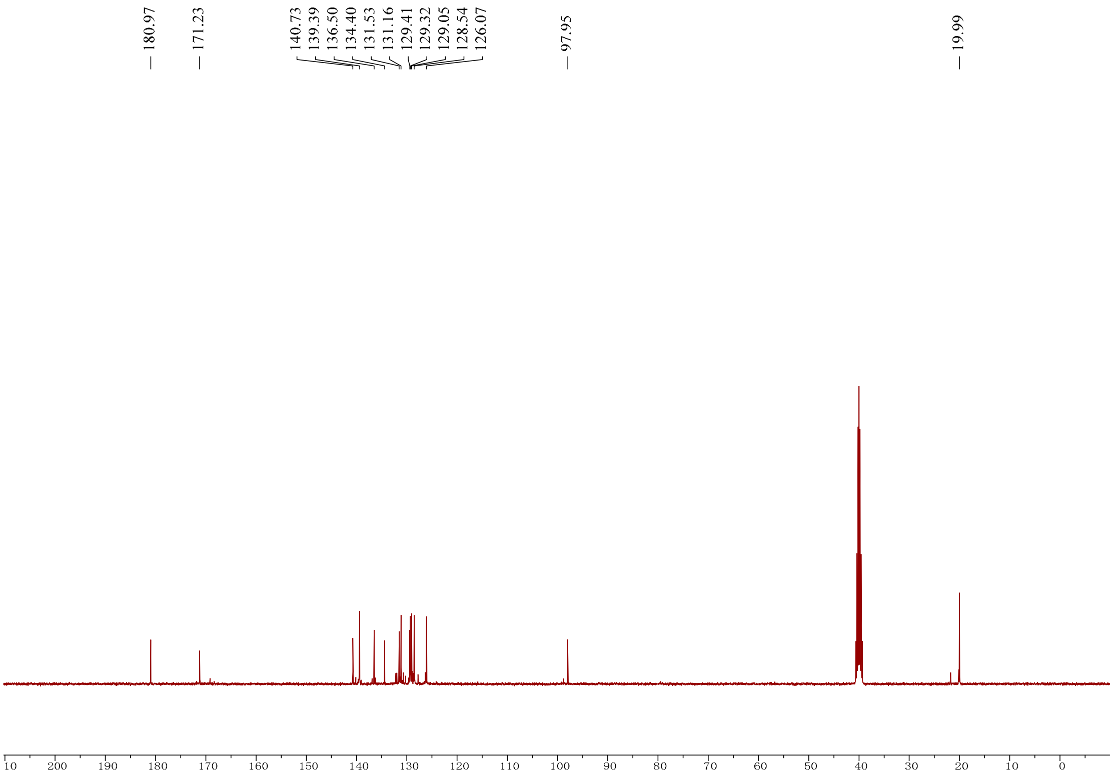

4-fluoro-*N*-((2-iodophenyl)carbamothioyl)benzamide (2f)

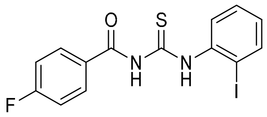

2f

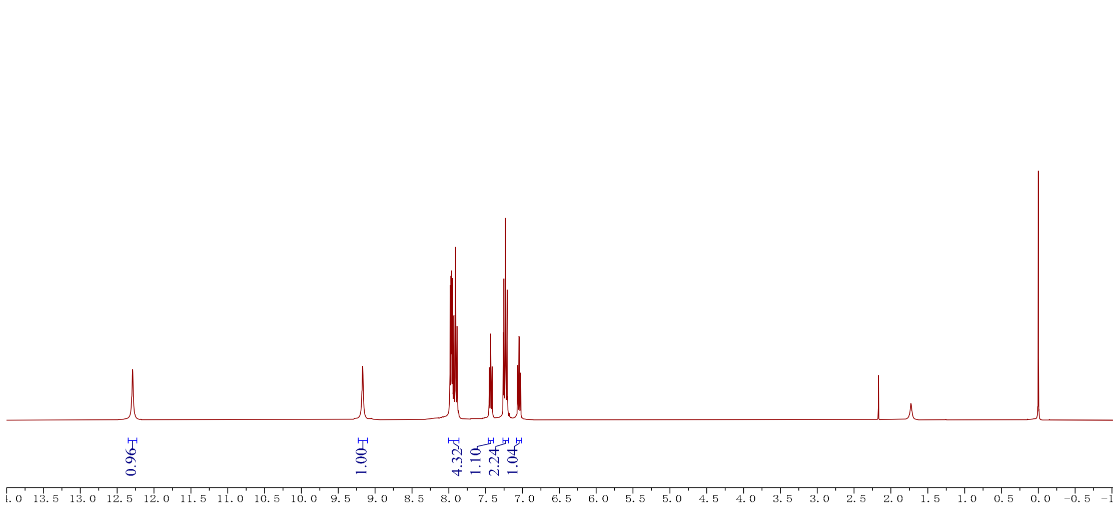

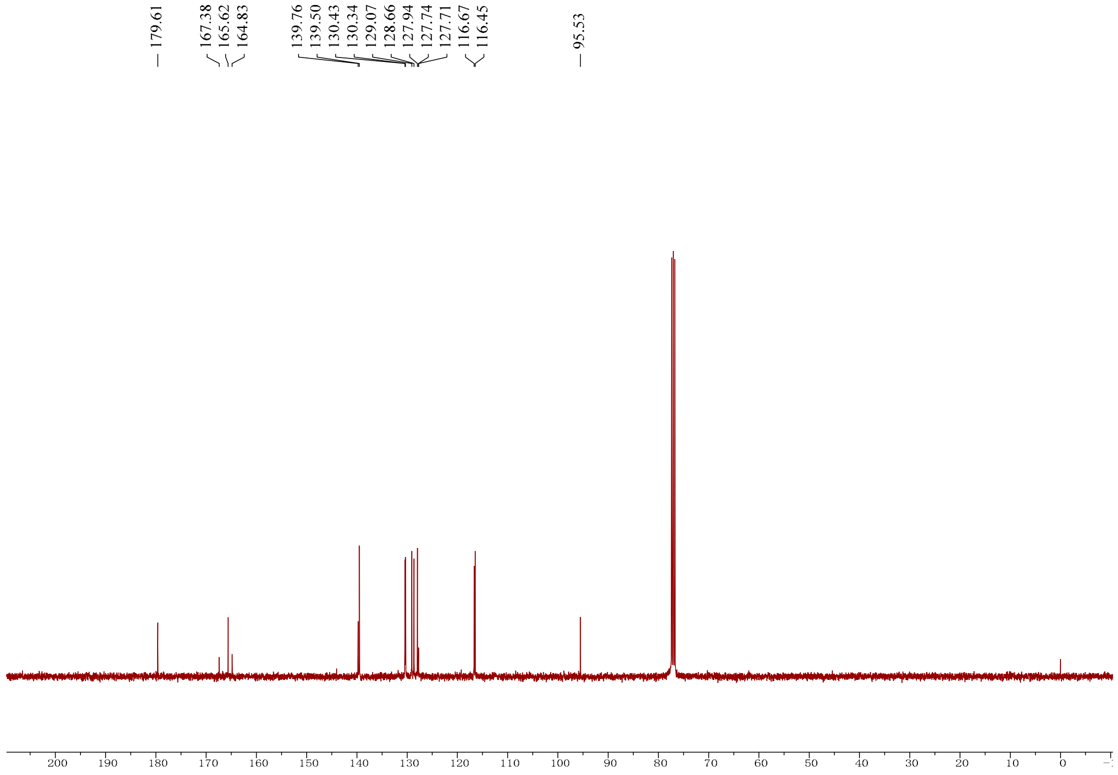

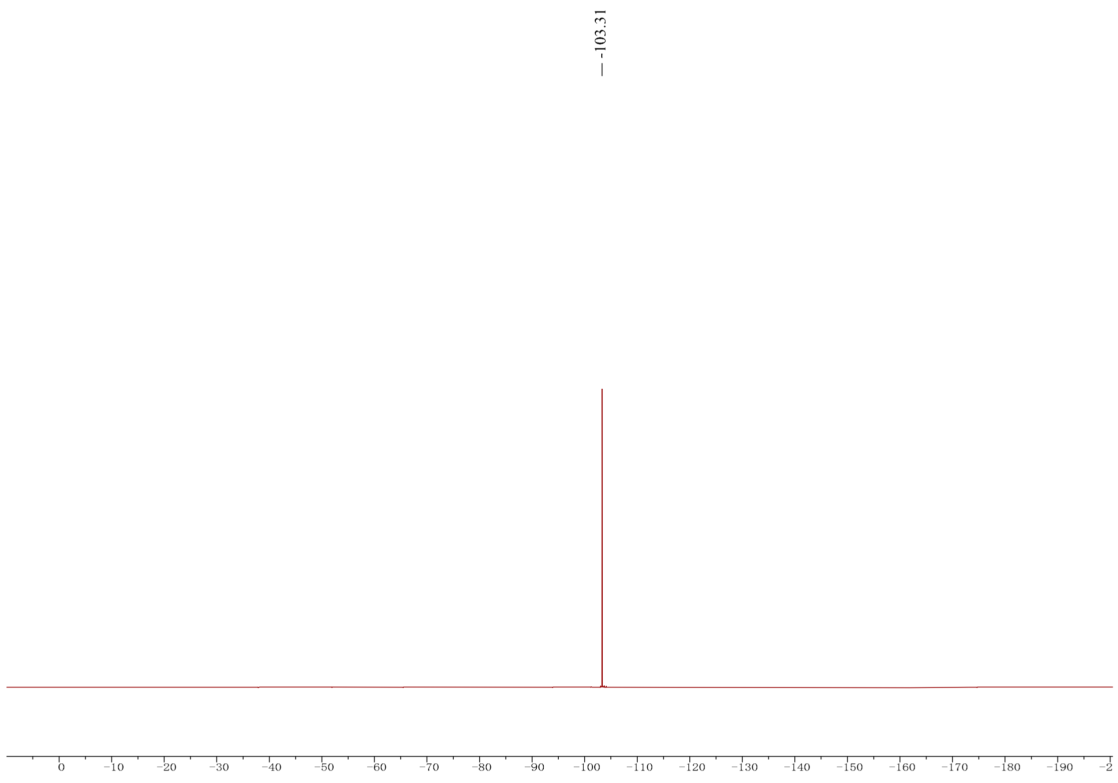
-103.31
0
-10
-20
-30
-40
-50
-60
-70
-80
-90
-100
-110
-120
-130
-140
-150
-160
-170
-180
-190
-2

4-chloro-*N*-((2-iodophenyl)carbamothioyl)benzamide (2g)

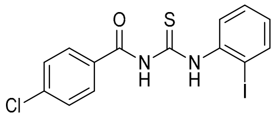

2g

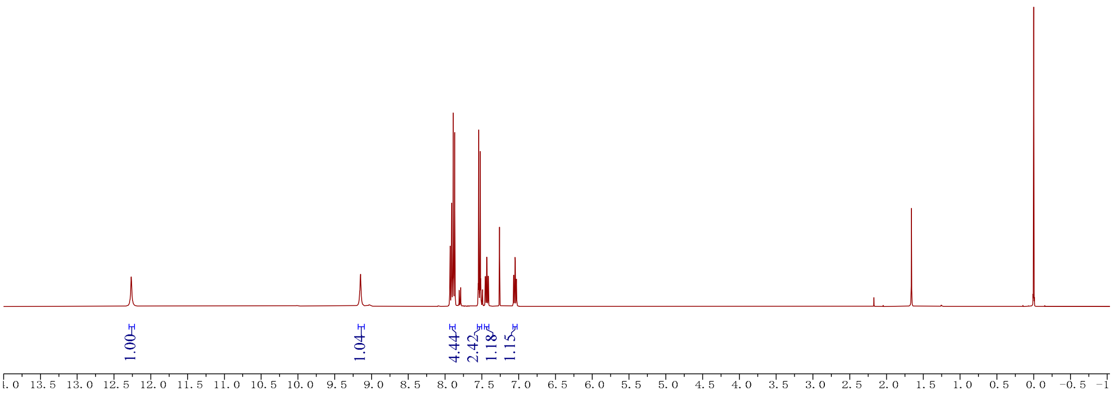

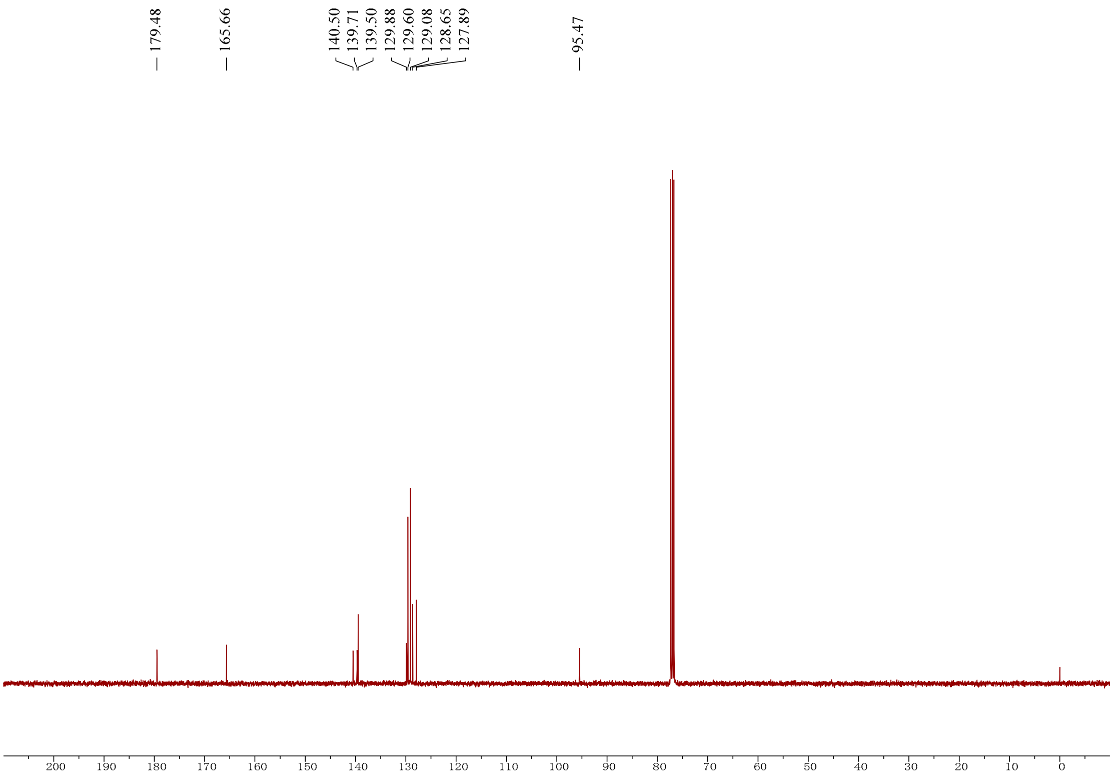

4-bromo-*N*-((2-iodophenyl)carbamothioyl)benzamide (2h)

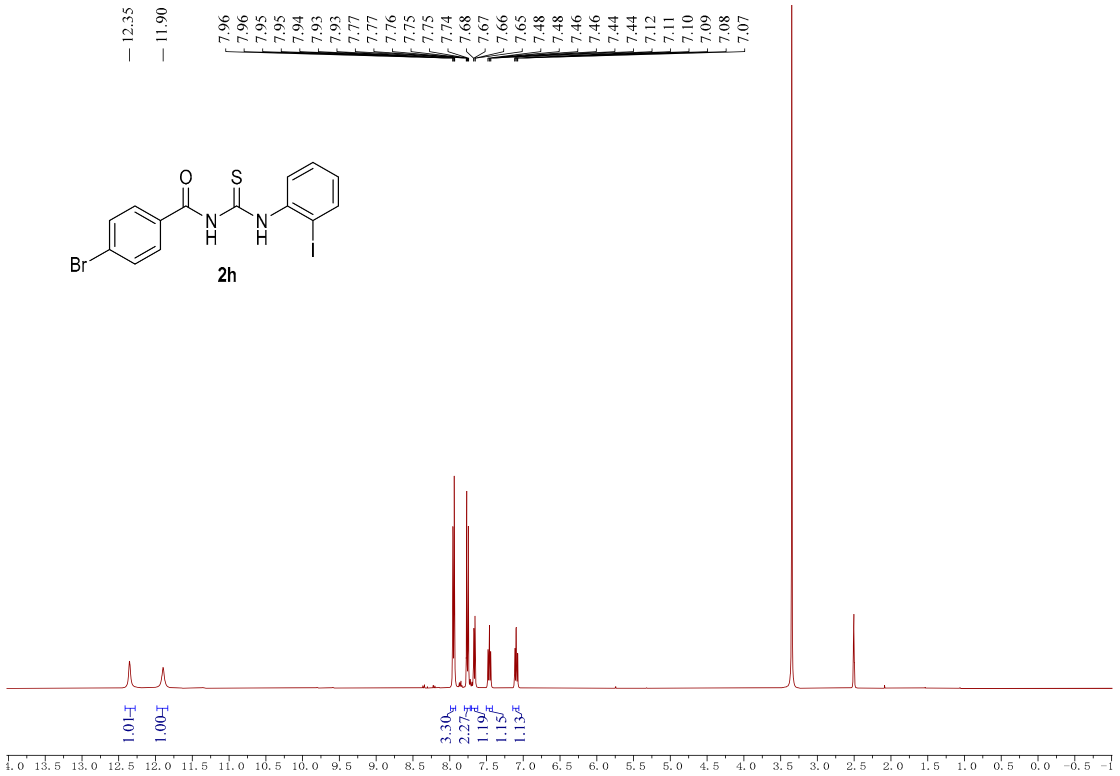

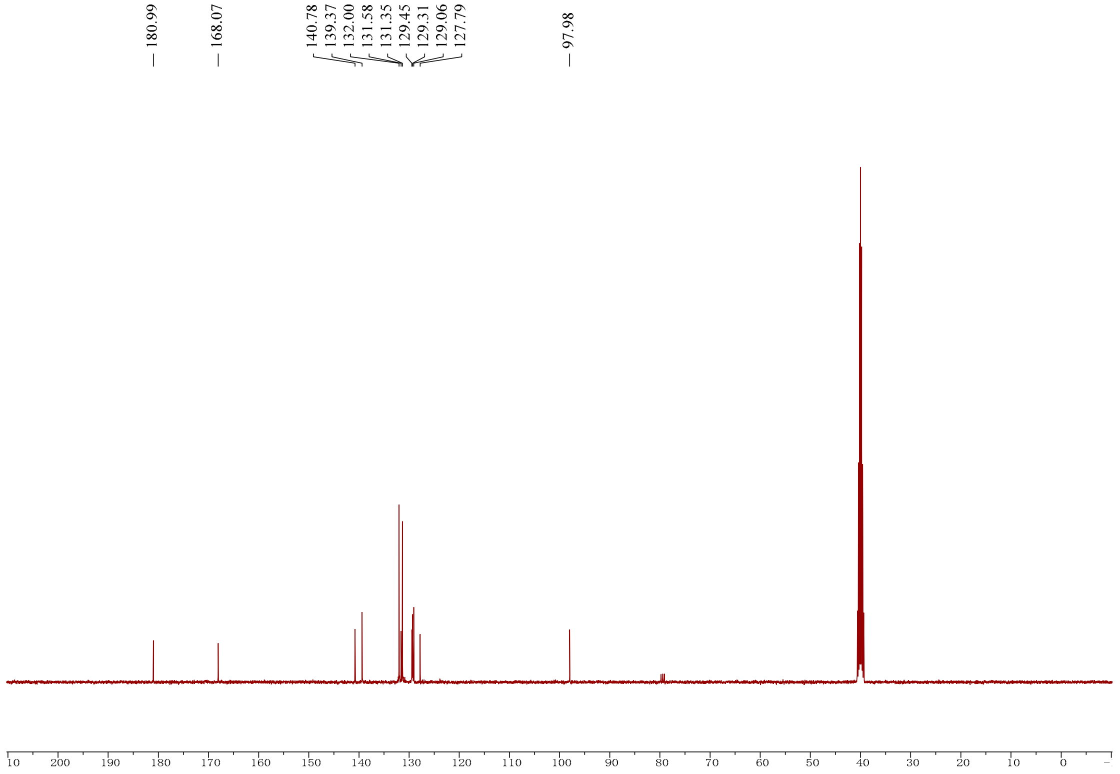

3-fluoro-*N*-((2-iodophenyl)carbamothioyl)benzamide (2i)

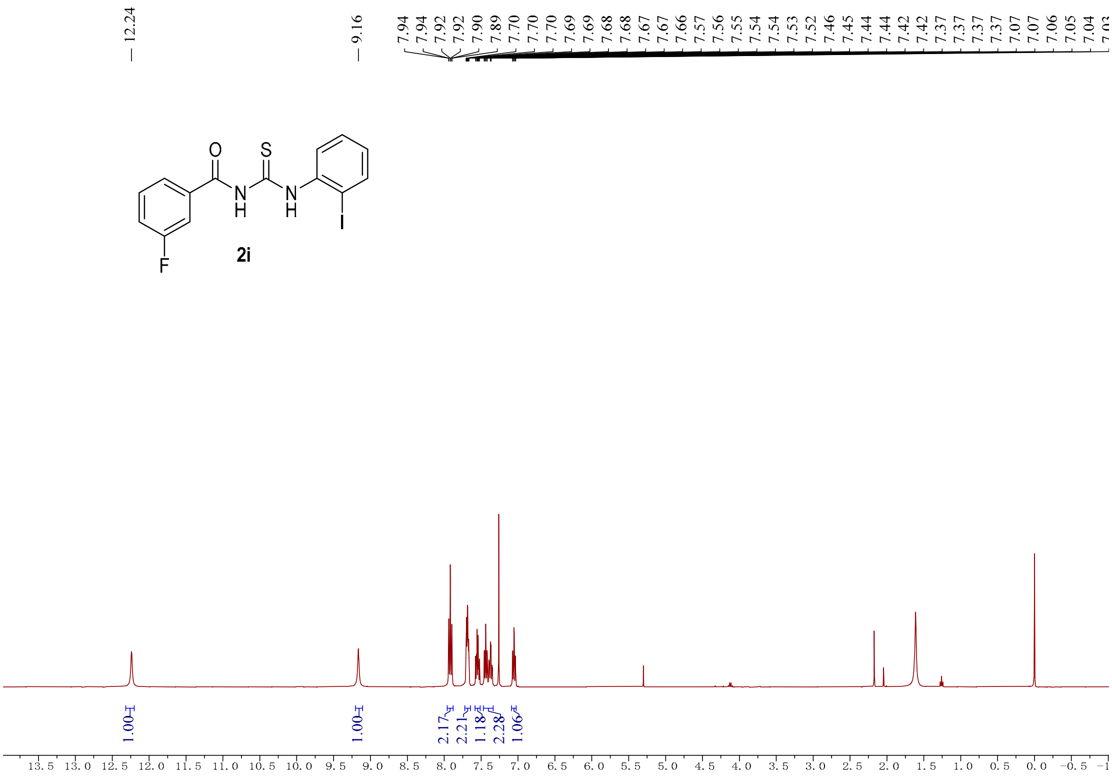

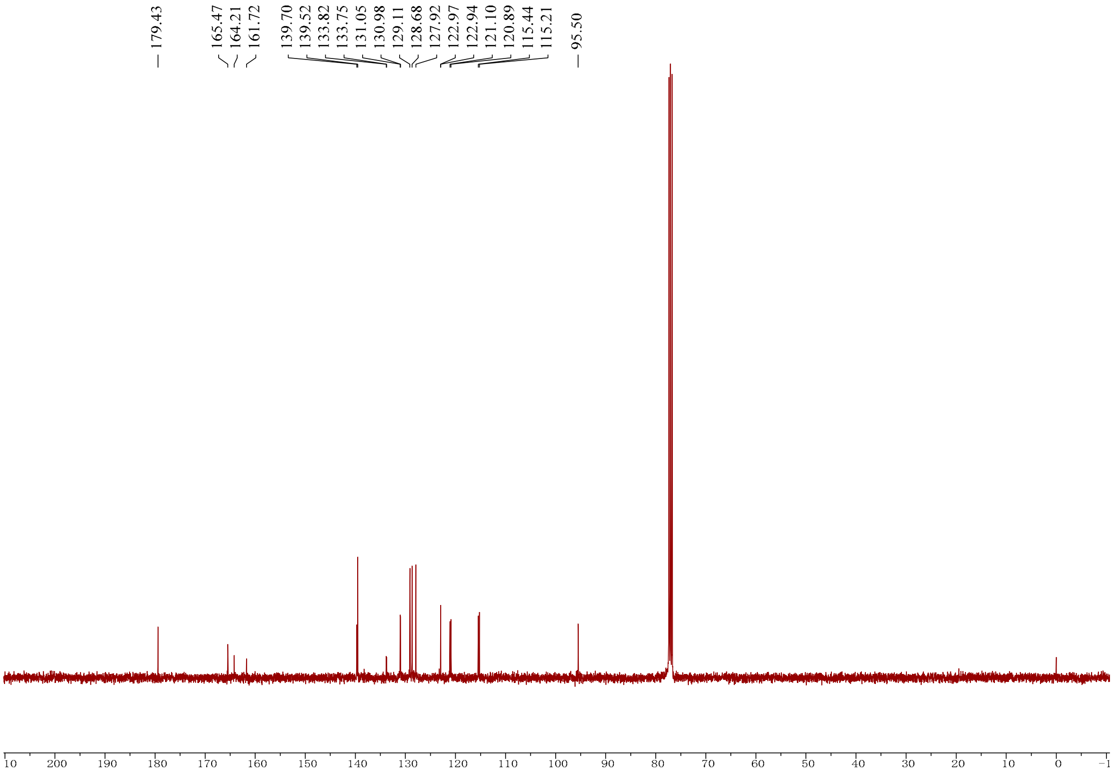

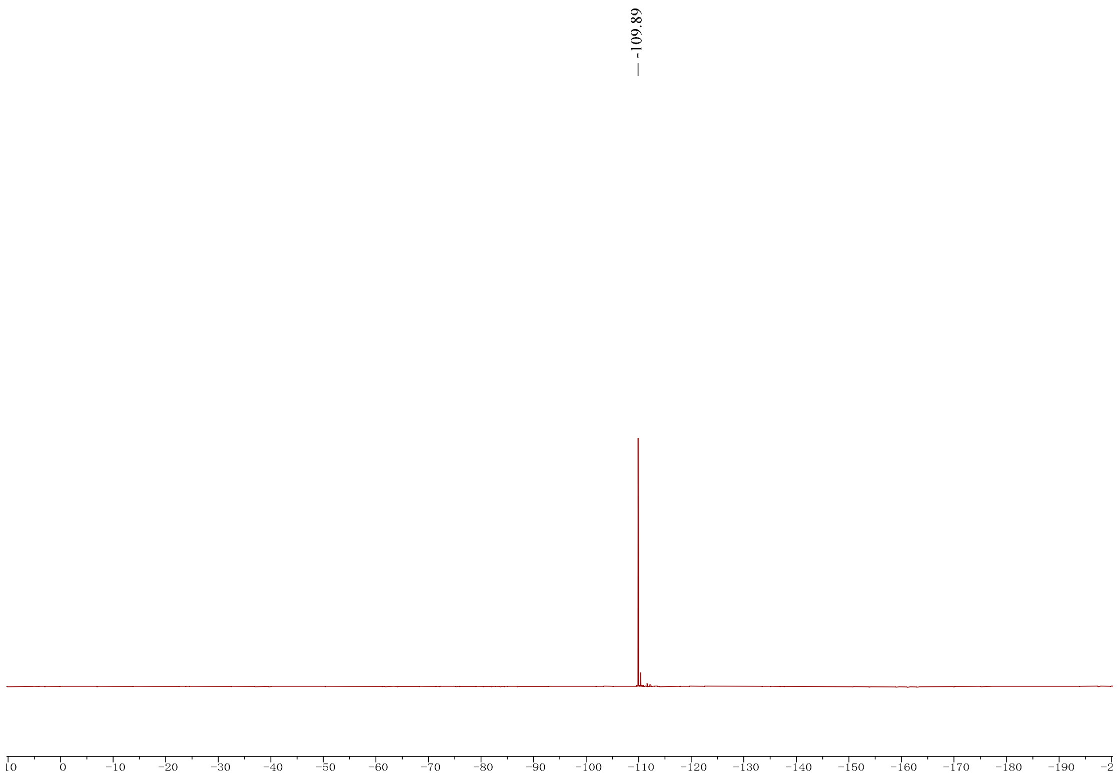
-109.89
10 0 -10 -20 -30 -40 -50 -60 -70 -80 -90 -100 -110 -120 -130 -140 -150 -160 -170 -180 -190 -2

3-chloro-*N*-((2-iodophenyl)carbamothioyl)benzamide (2j)

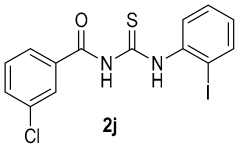

2j

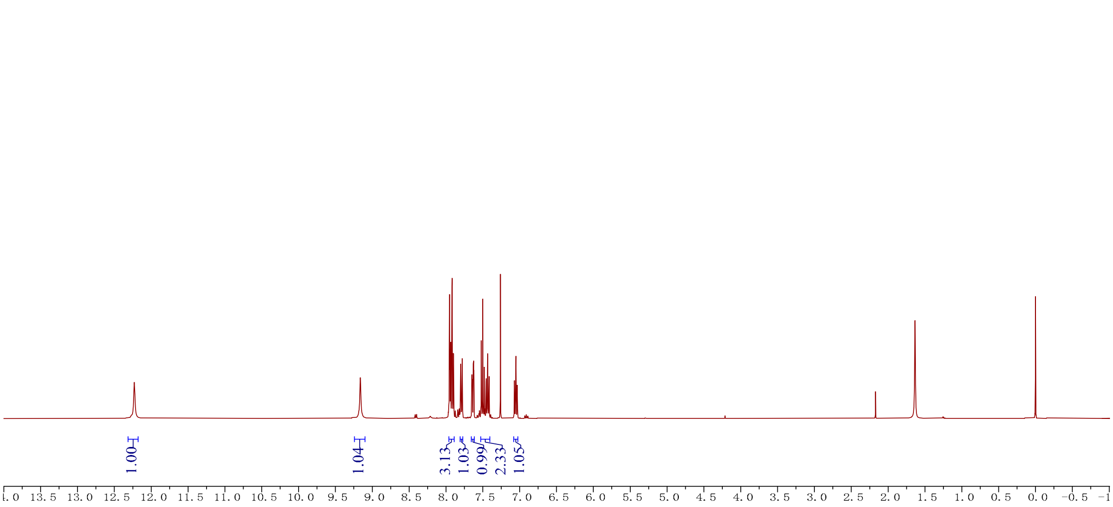

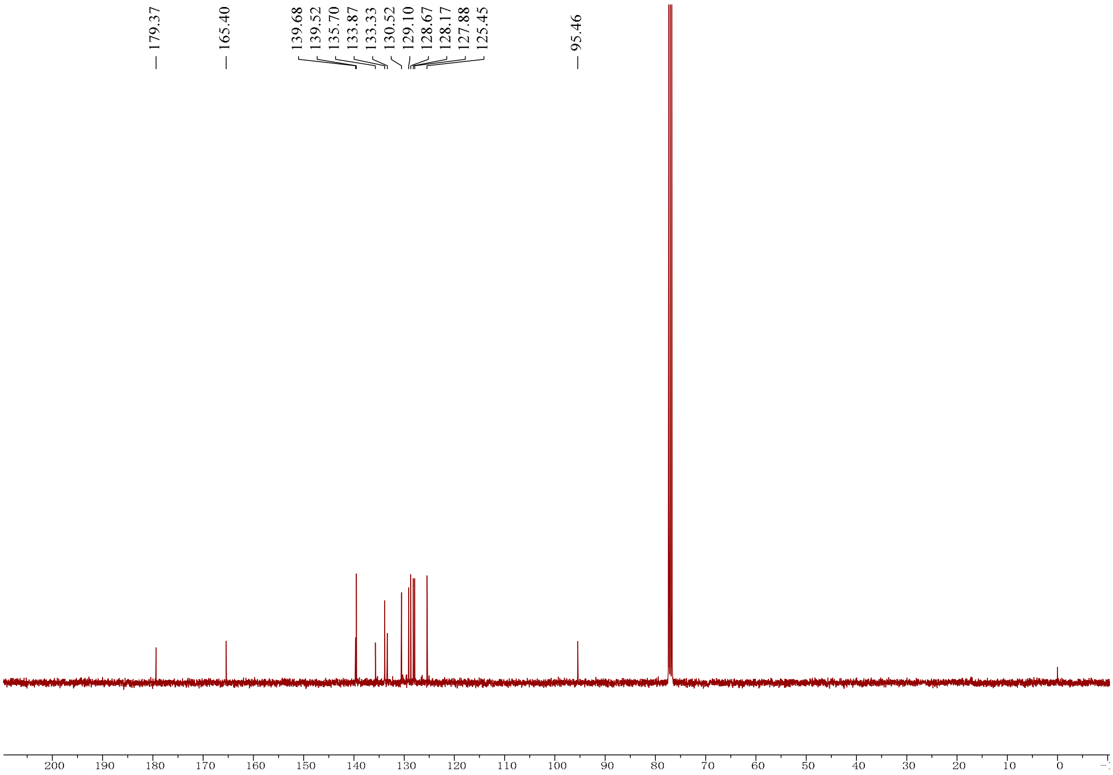

3-bromo-*N*-((2-iodophenyl)carbamothioyl)benzamide (2k)

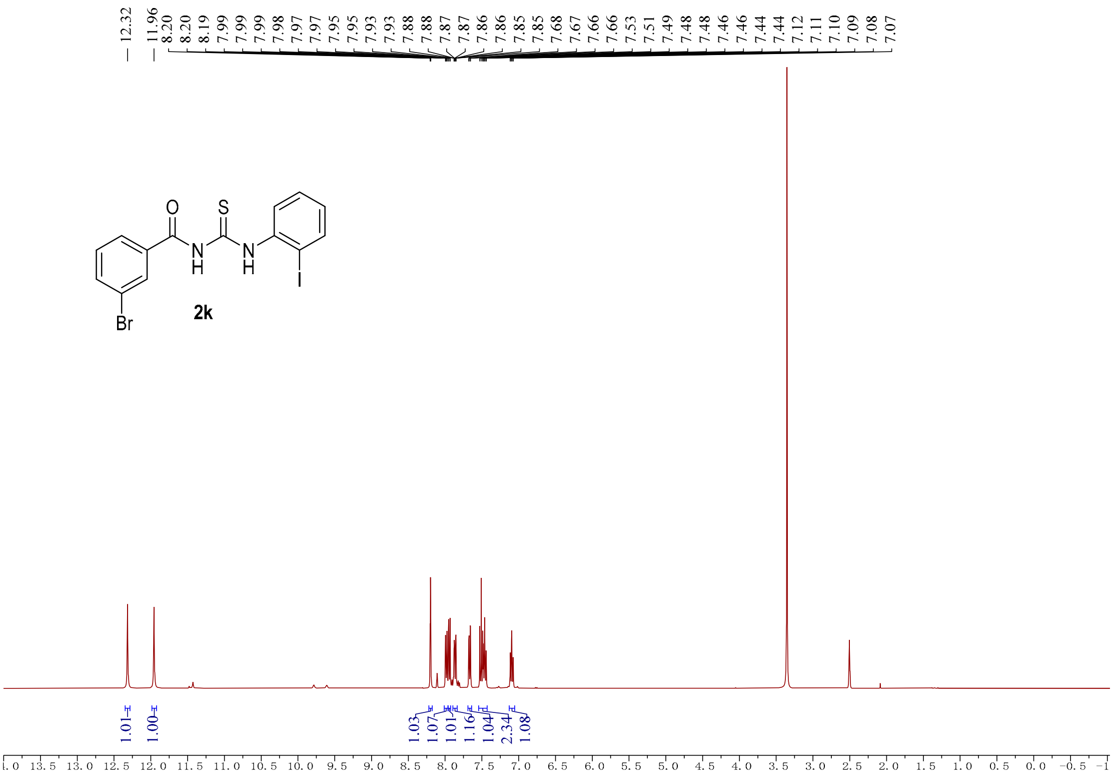

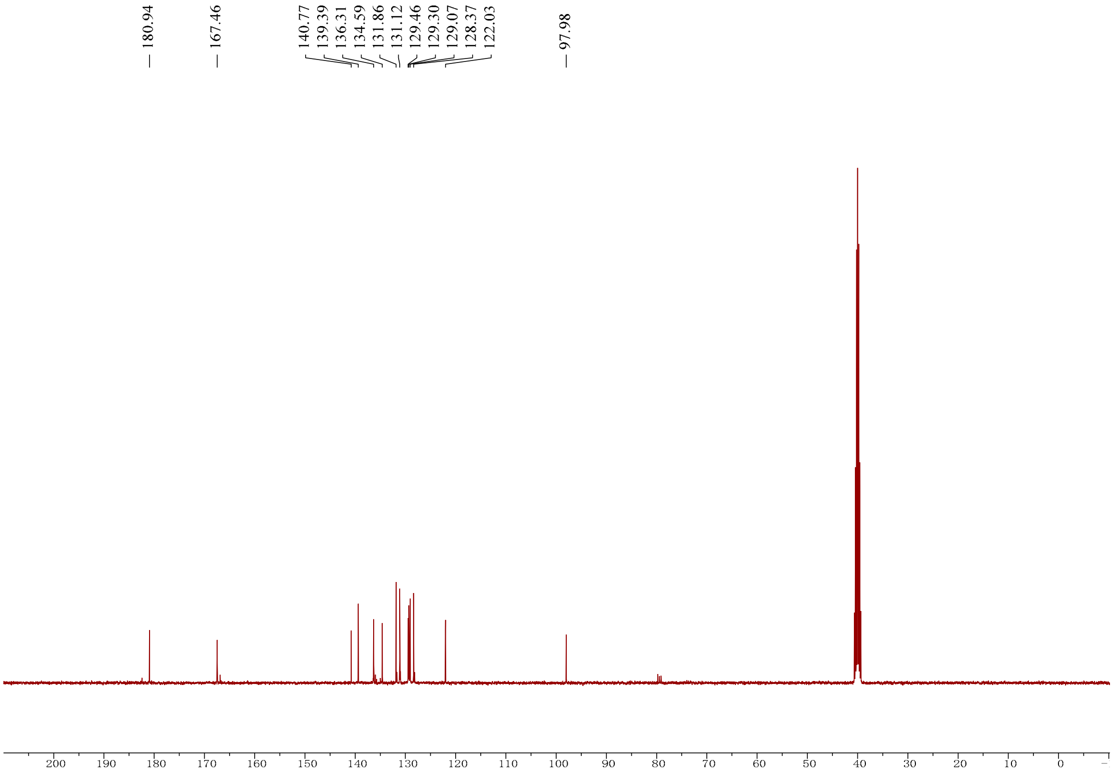

2-fluoro-*N*-((2-iodophenyl)carbamothioyl)benzamide (2l)

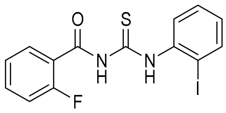

2l

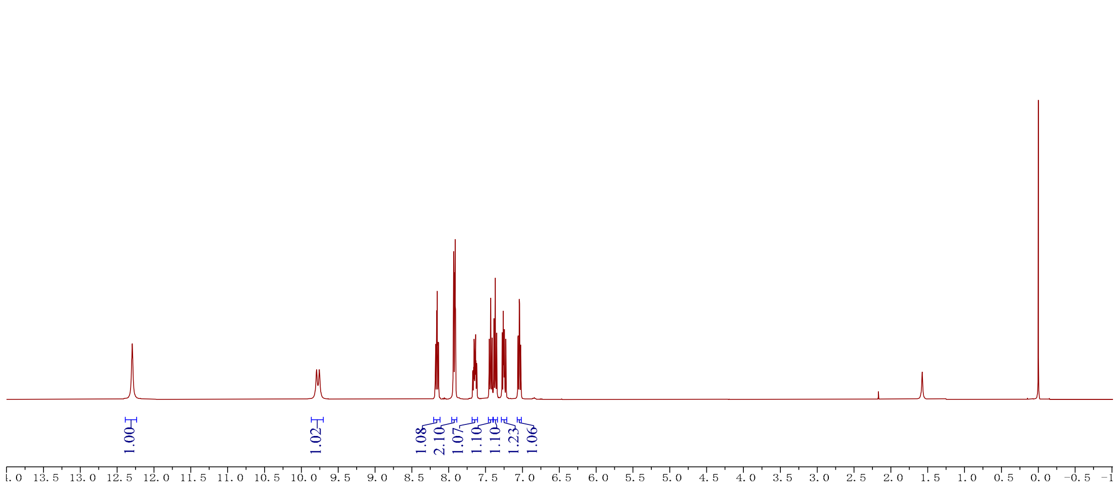

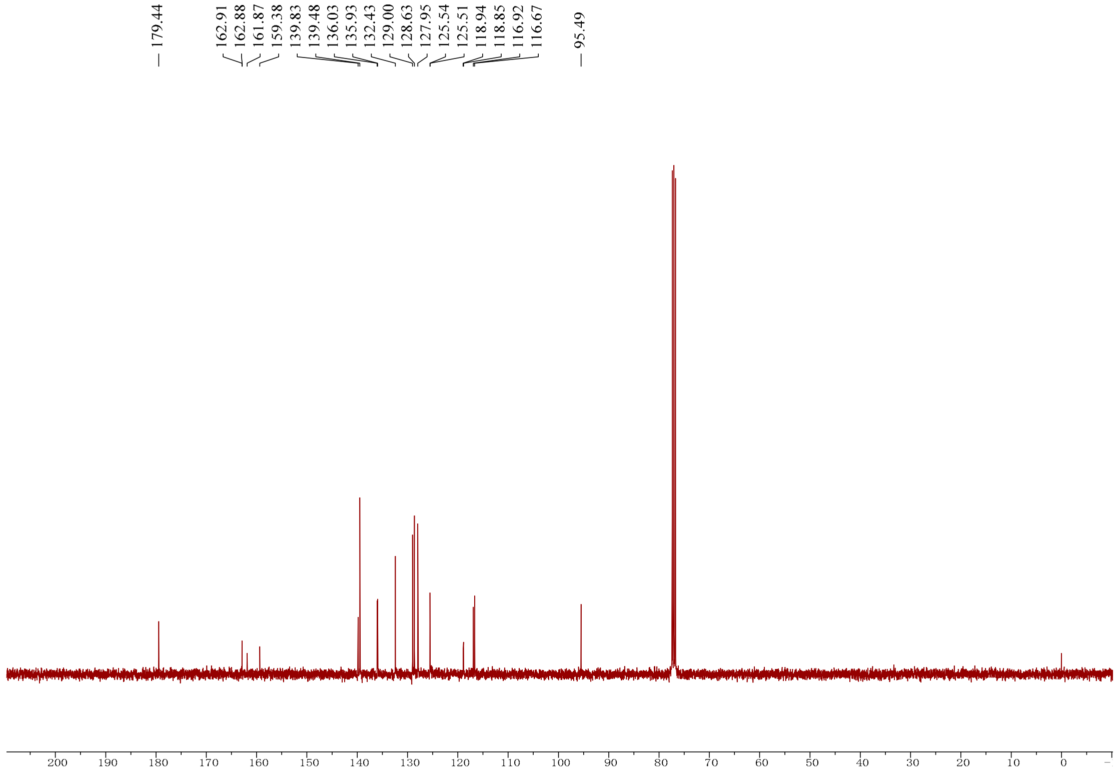

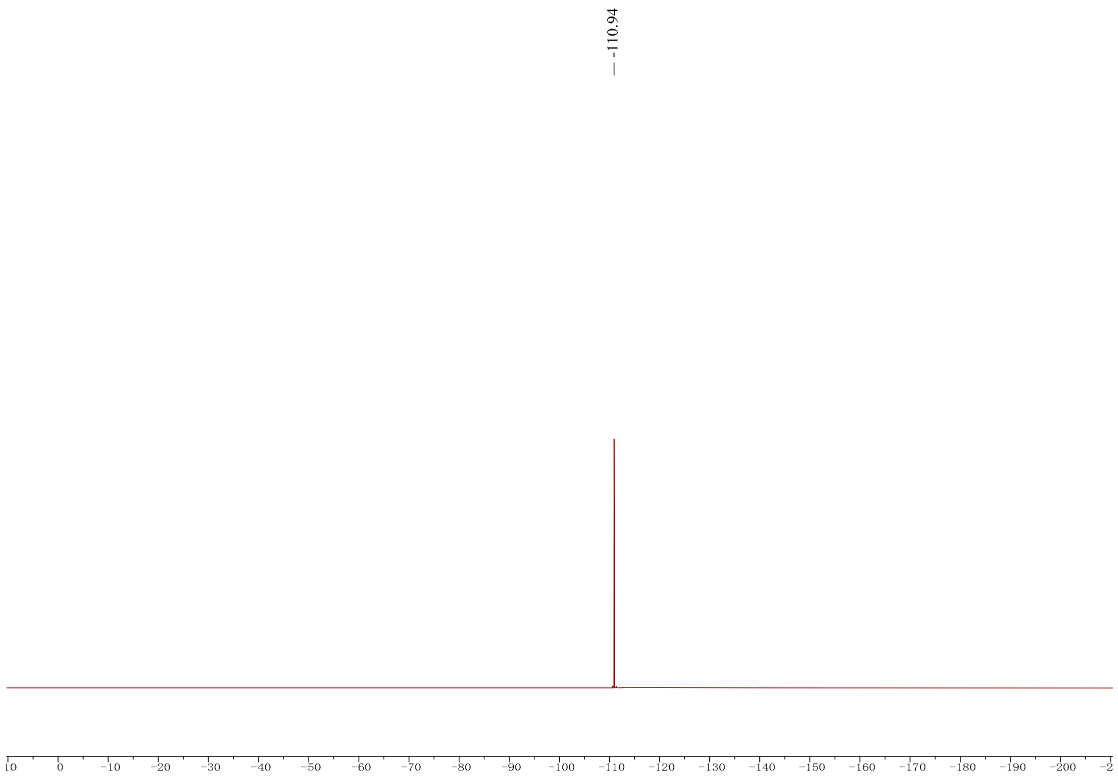

-110.94
10
0
-10
-20
-30
-40
-50
-60
-70
-80
-90
-100
-110
-120
-130
-140
-150
-160
-170
-180
-190
-200
-2

2-chloro-*N*-((2-iodophenyl)carbamothioyl)benzamide (2m)

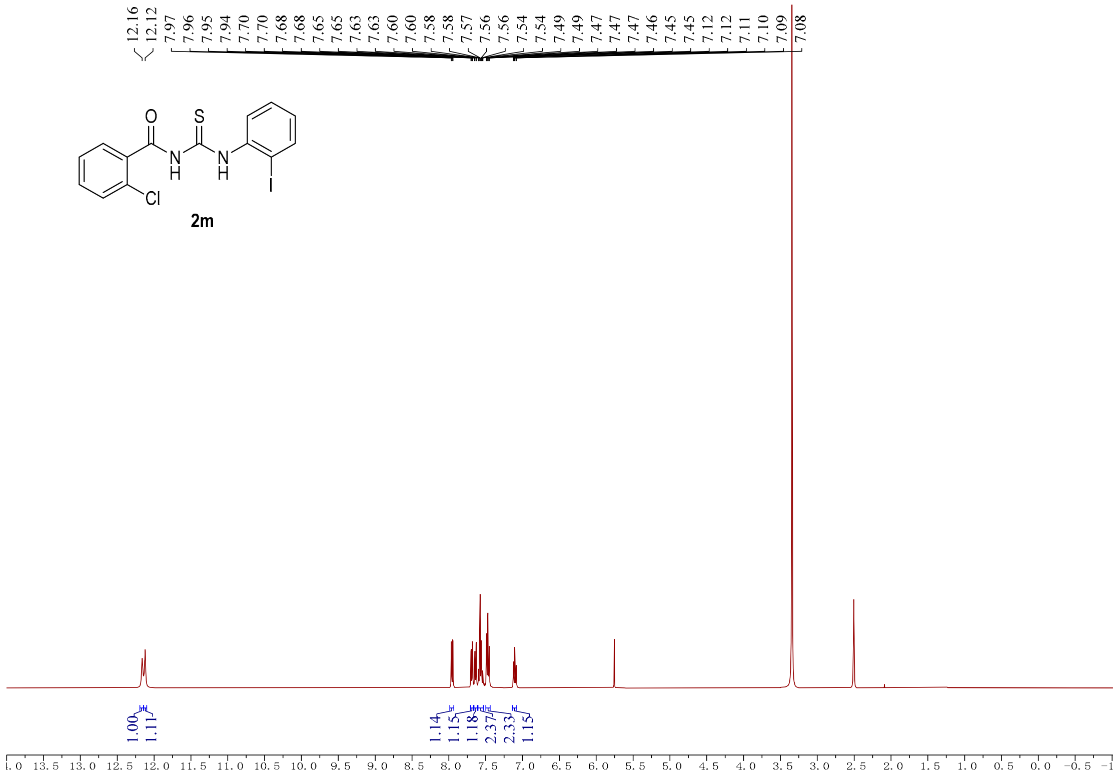

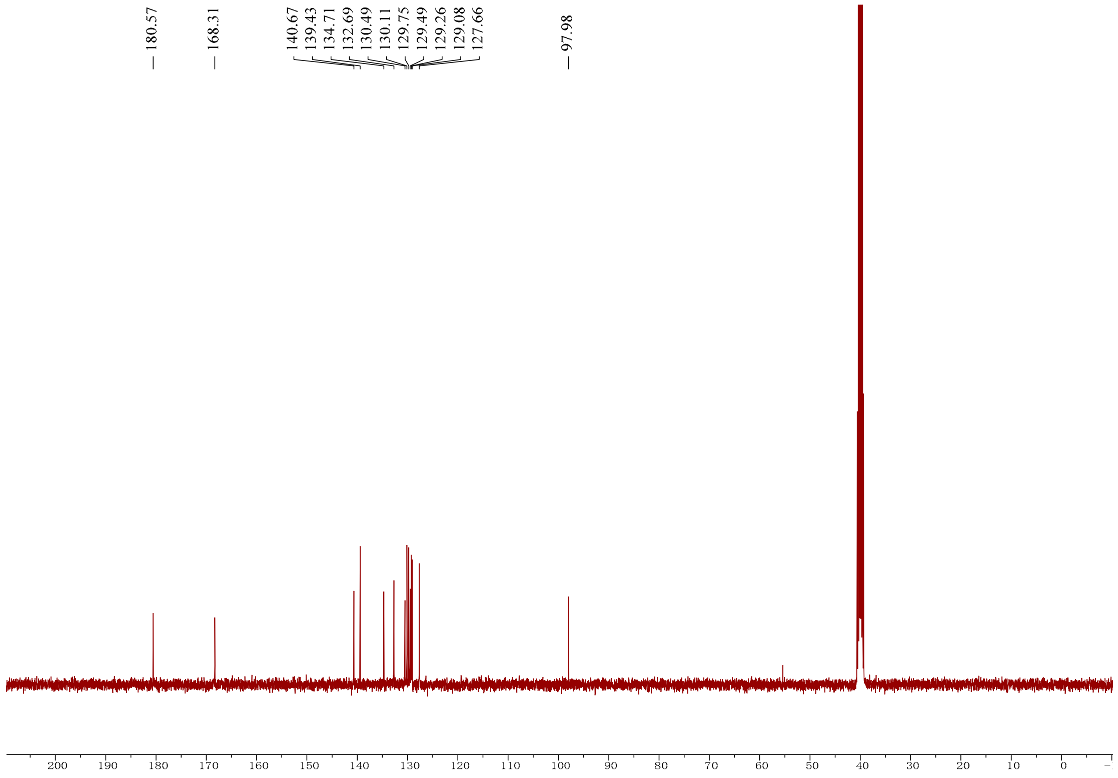

2-bromo-*N*-((2-iodophenyl)carbamothioyl)benzamide (2n)

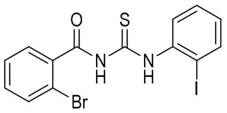

2n

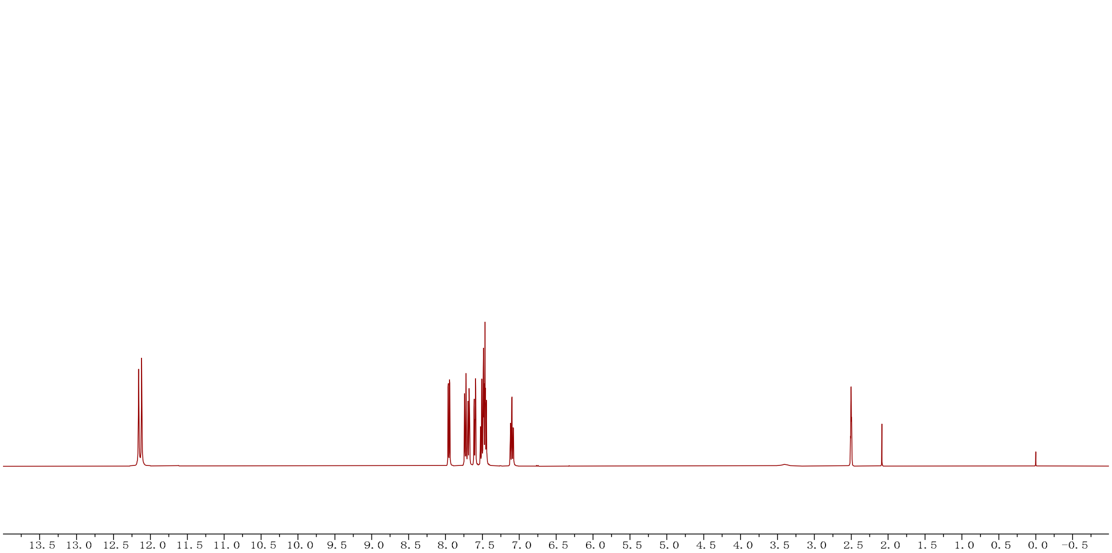

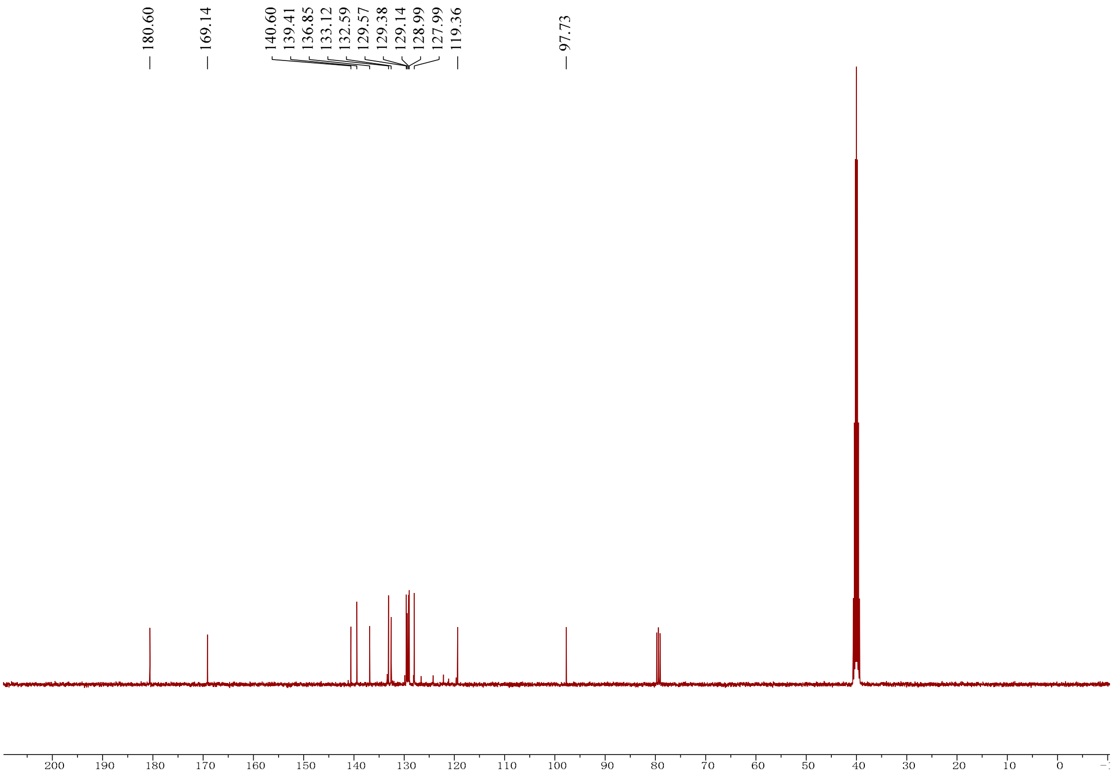

(*Z*)-*N*-(3-(2-iodophenyl)-4-oxo-6-phenyl-3,4-dihydro-2*H*-1,3-thiazin-2-ylidene)benzamide (3a)

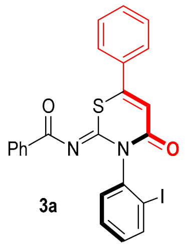

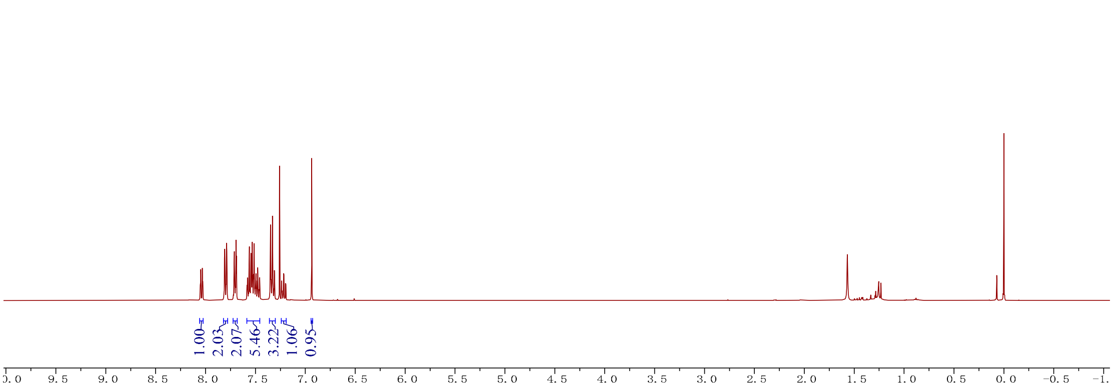

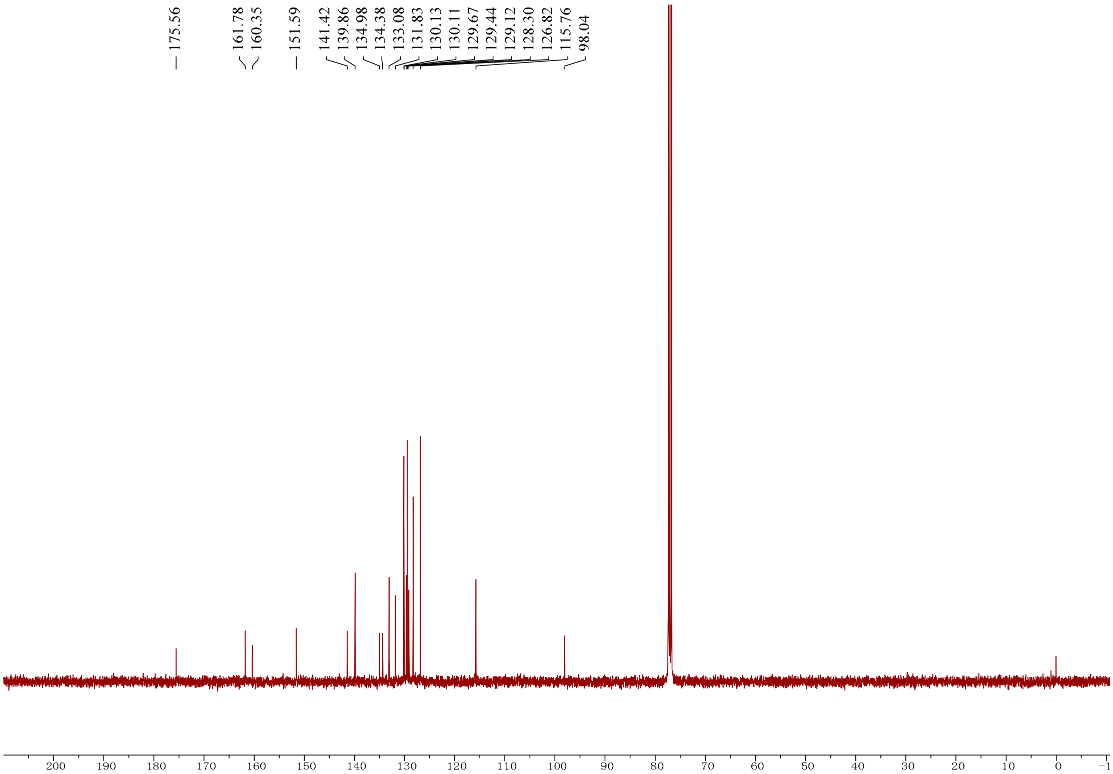

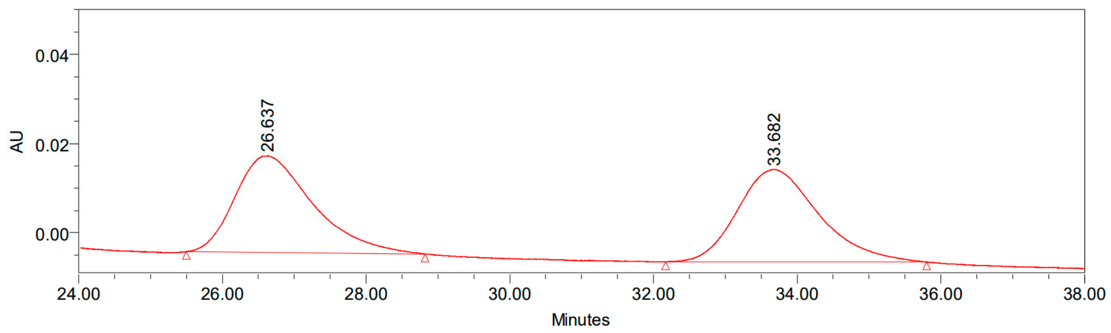

	RT	Area	% Area	Height
1	26.637	1640855	50.16	21730
2	33.682	1630575	49.84	20736

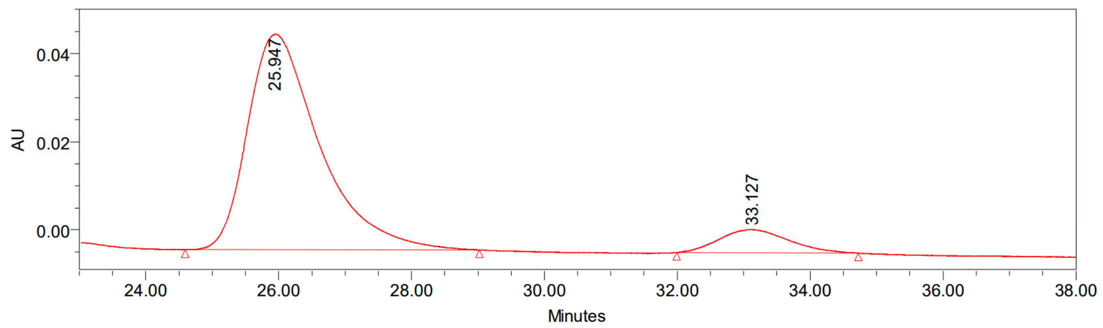

	RT	Area	% Area	Height
1	25.947	3689754	90.69	48948
2	33.127	378983	9.31	5240

(*Z*)-*N*-(3-(2-iodophenyl)-6-(4-methoxyphenyl)-4-oxo-3,4-dihydro-2*H*-1,3-thiazin-2-ylidene)benzamide (3b)

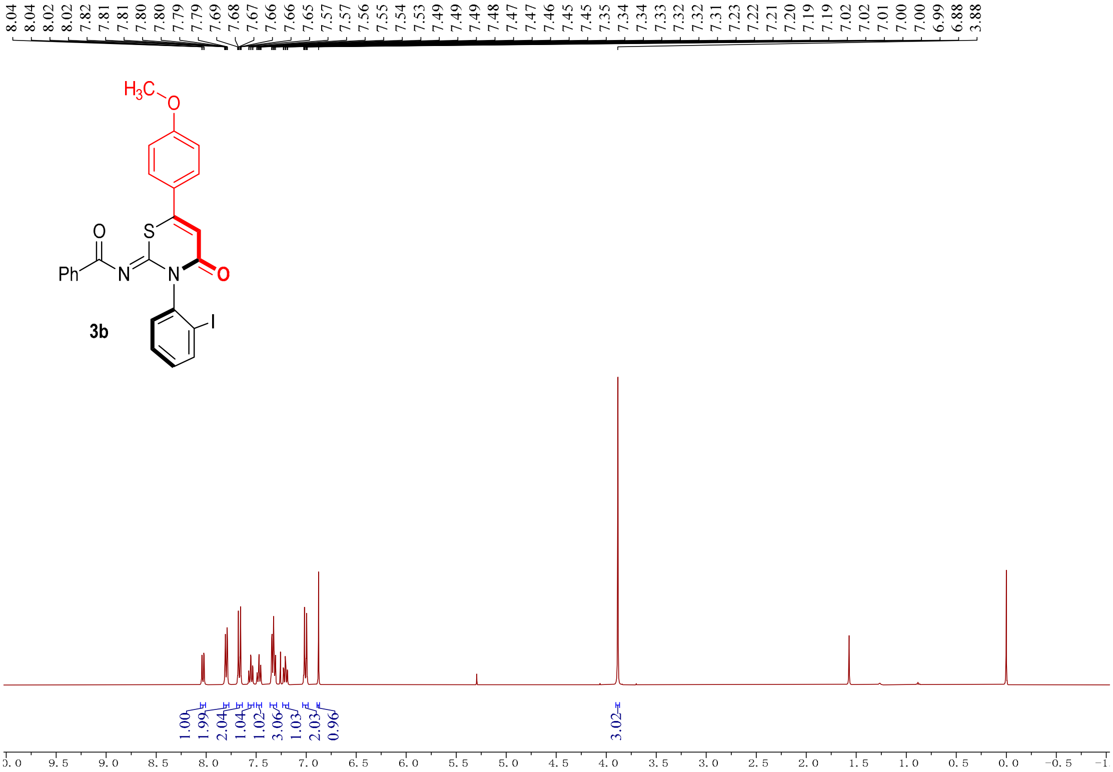

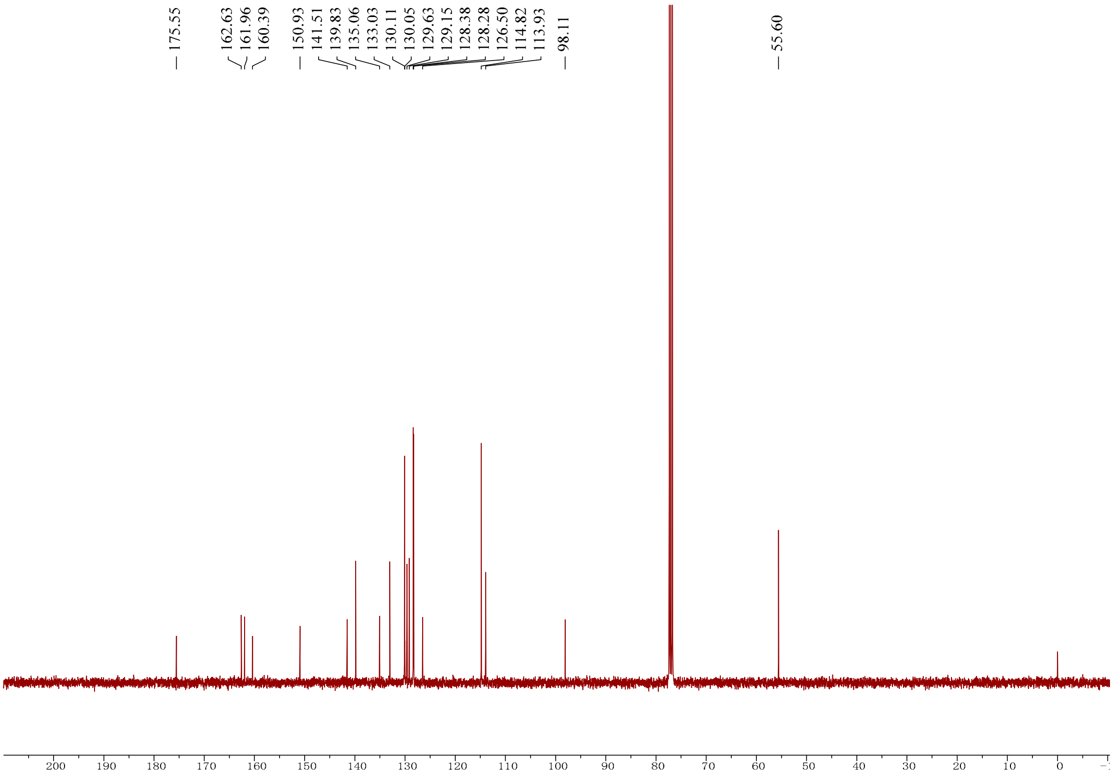

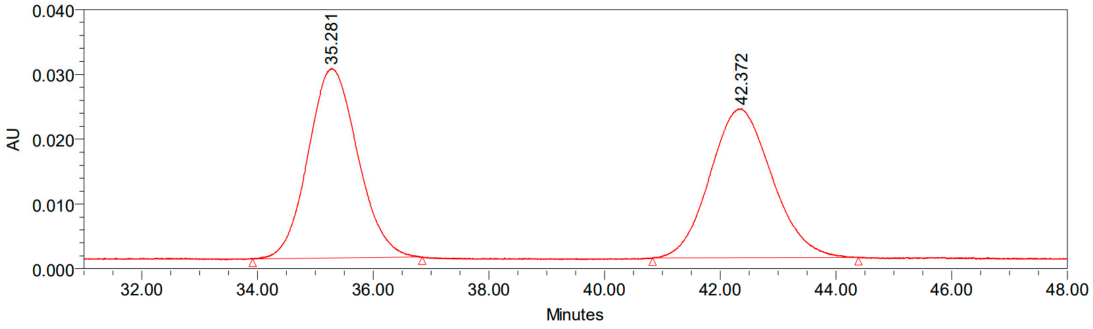

	RT	Area	% Area	Height
1	35.281	1740090	50.39	29236
2	42.372	1712849	49.61	22988

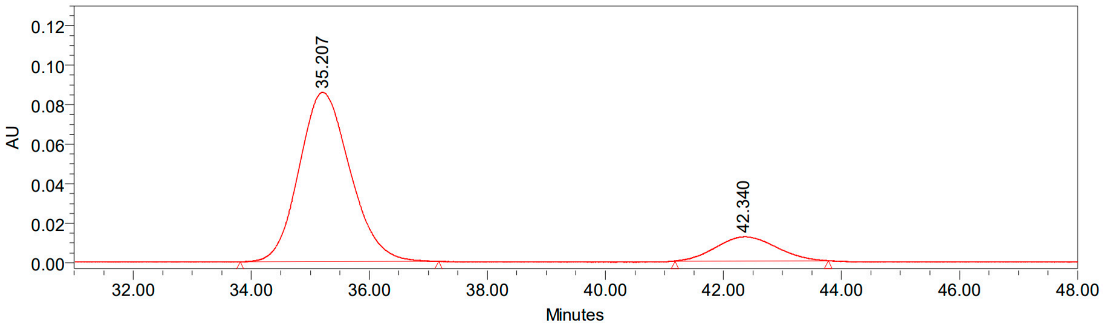

	RT	Area	% Area	Height
1	35.207	5138184	85.62	85662
2	42.340	863117	14.38	12157

(*Z*)-*N*-(3-(2-iodophenyl)-4-oxo-6-(*p*-tolyl)-3,4-dihydro-2*H*-1,3-thiazin-2-ylidene)benzamide (3c)

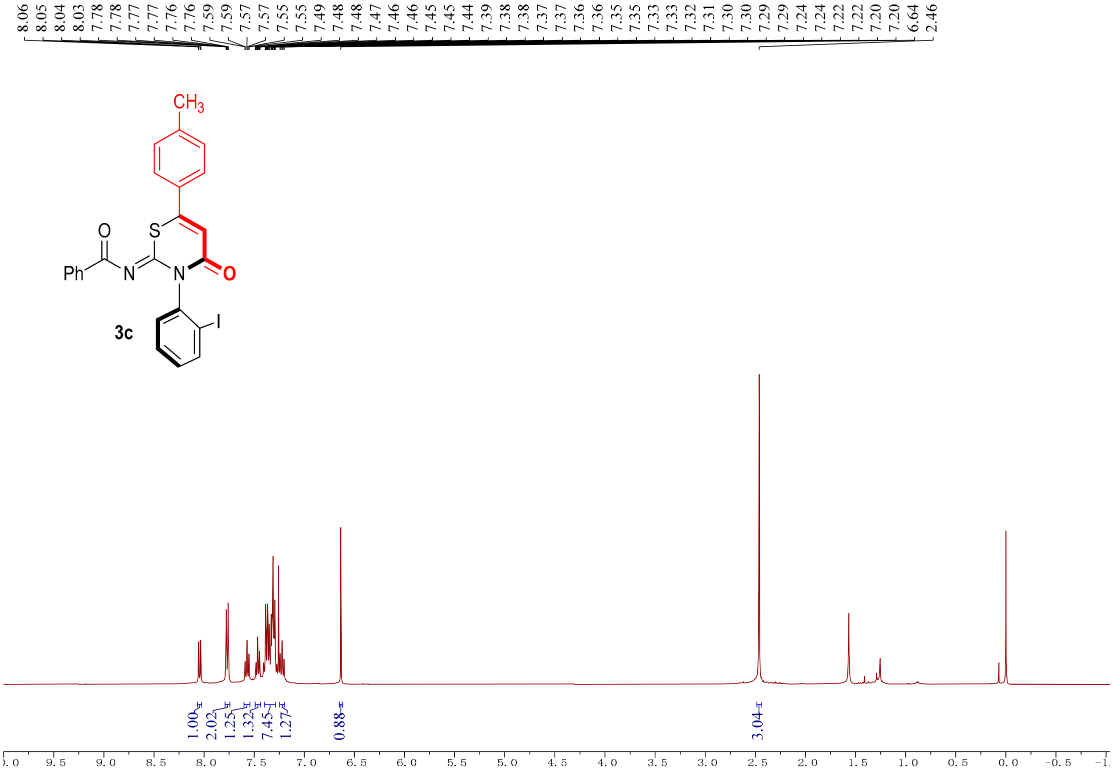

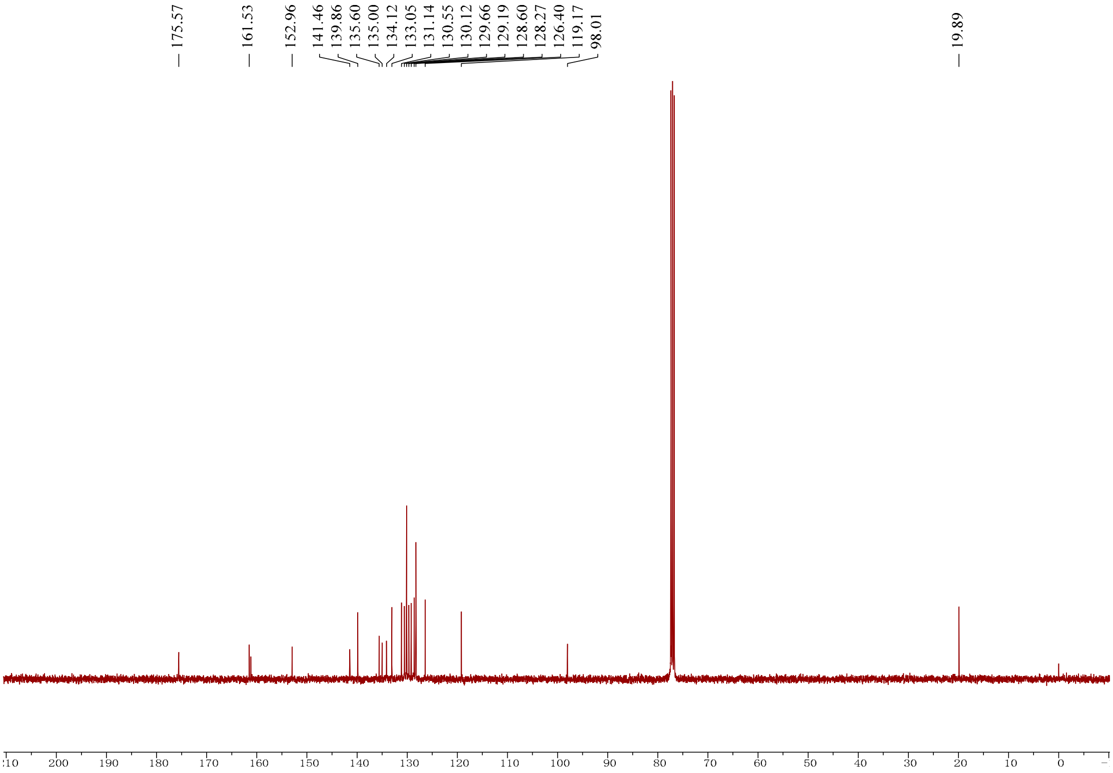

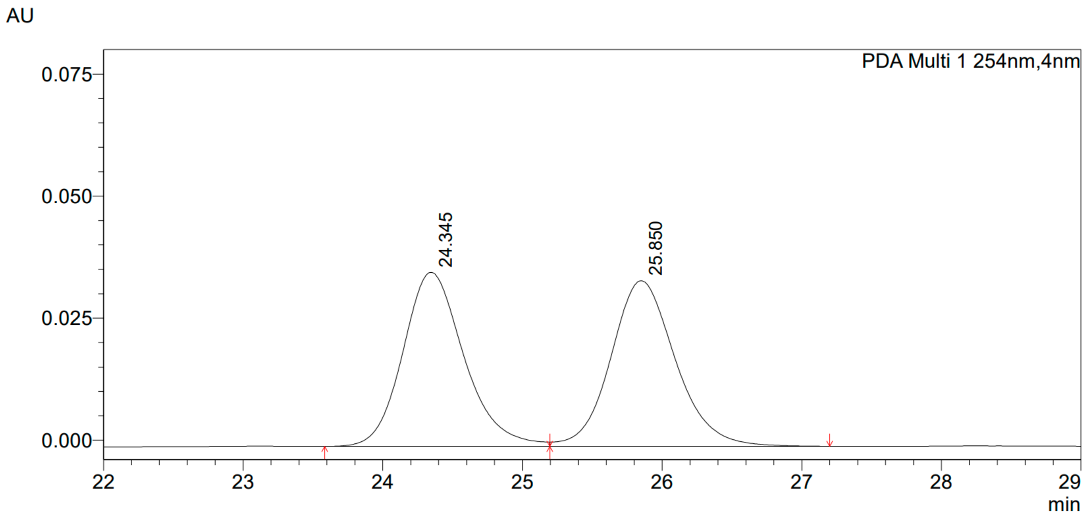

PDA Ch1 254nm

Peak#	Ret. Time	Area	Height	Area%
1	24.345	1088350	35655	49.869
2	25.850	1094083	33935	50.131
Total		2182434	69590	100.000

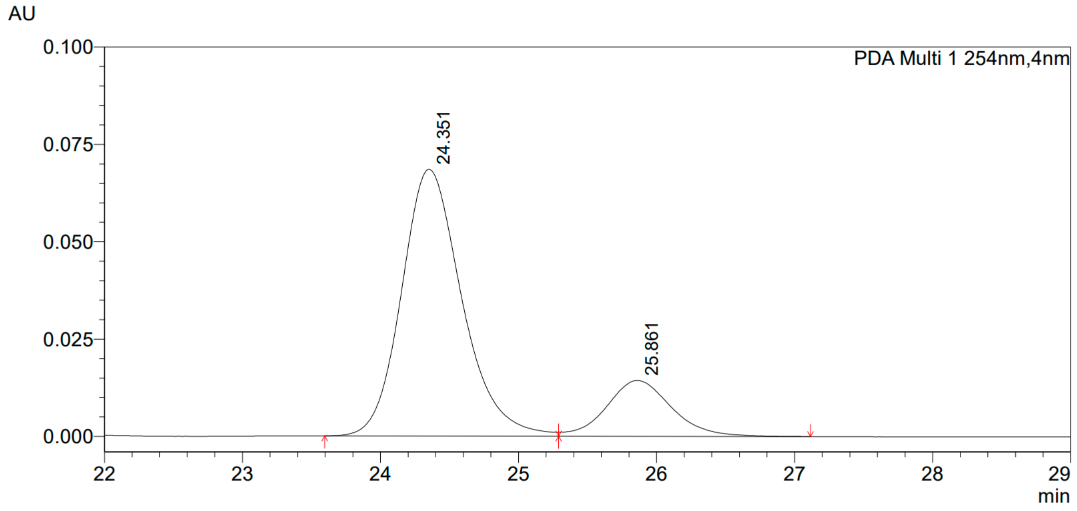

PDA Ch1 254nm

Peak#	Ret. Time	Area	Height	Area%
1	24.351	2083723	68505	81.617
2	25.861	469324	14336	18.383
Total		2553047	82841	100.000

(*Z*)-*N*-(6-(4-fluorophenyl)-3-(2-iodophenyl)-4-oxo-3,4-dihydro-2*H*-1,3-thiazin-2-ylidene)benzamide (3d)

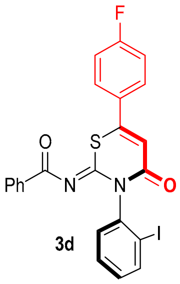

3d

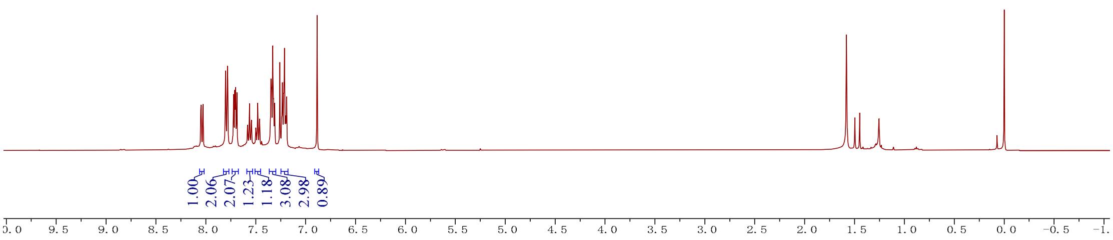

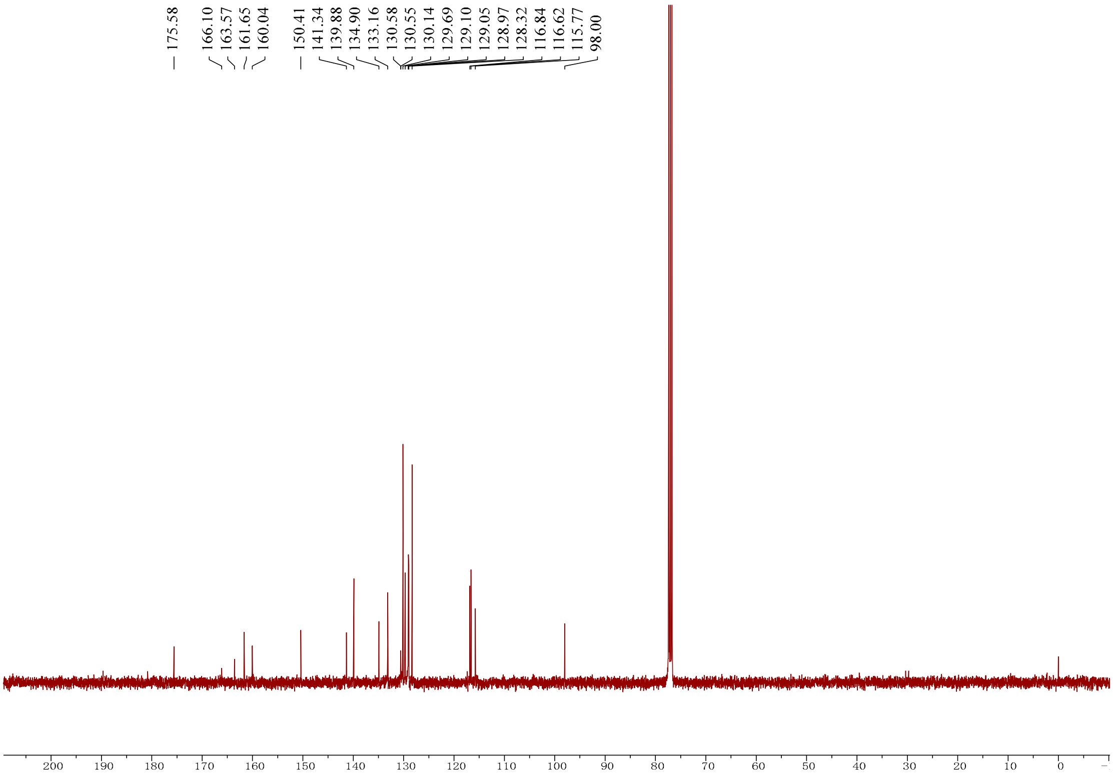

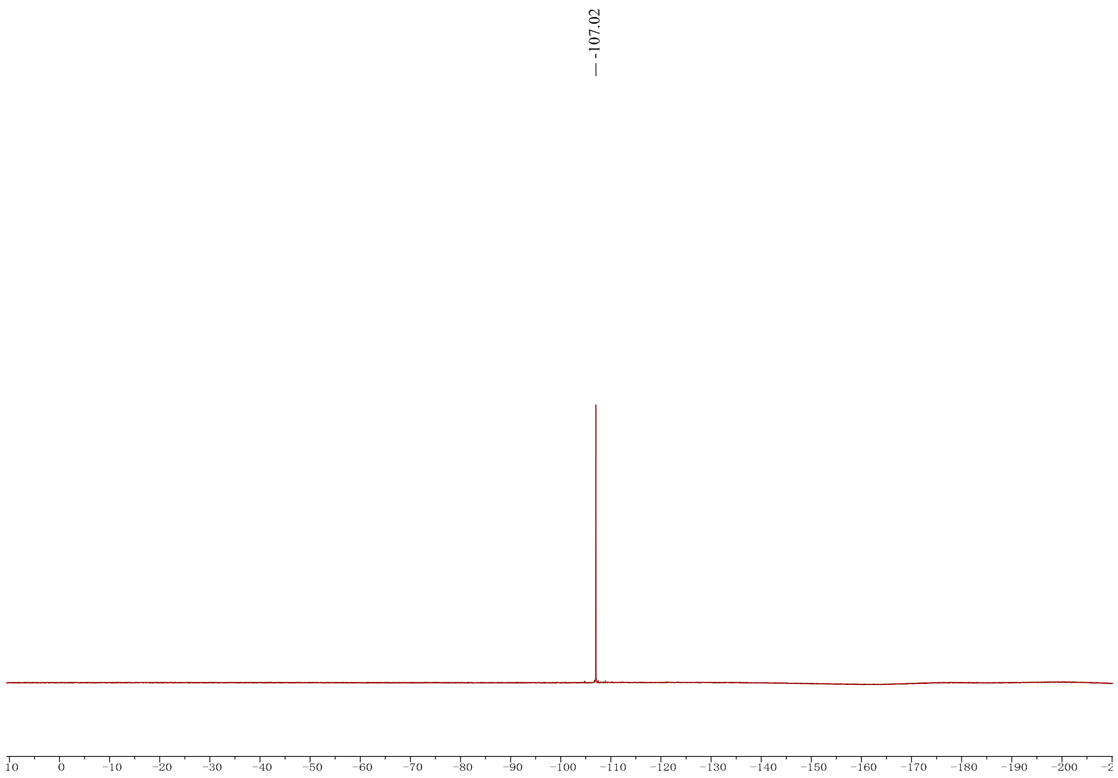
-107.02
10
0
-10
-20
-30
-40
-50
-60
-70
-80
-90
-100
-110
-120
-130
-140
-150
-160
-170
-180
-190
-200

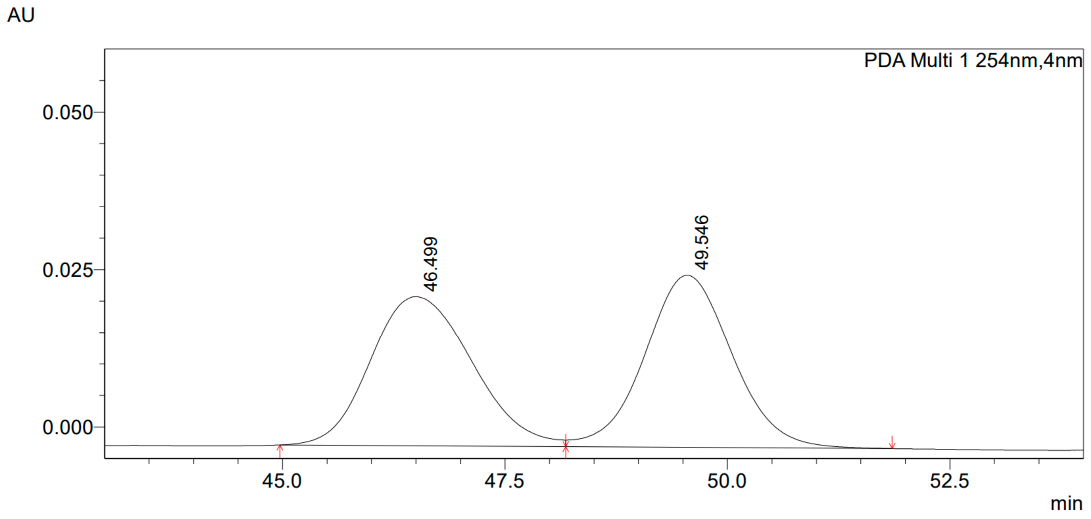

PDA Ch1 254nm

Peak#	Ret. Time	Area	Height	Area%
1	46.499	1882968	23687	50.127
2	49.546	1873439	27352	49.873
Total		3756407	51039	100.000

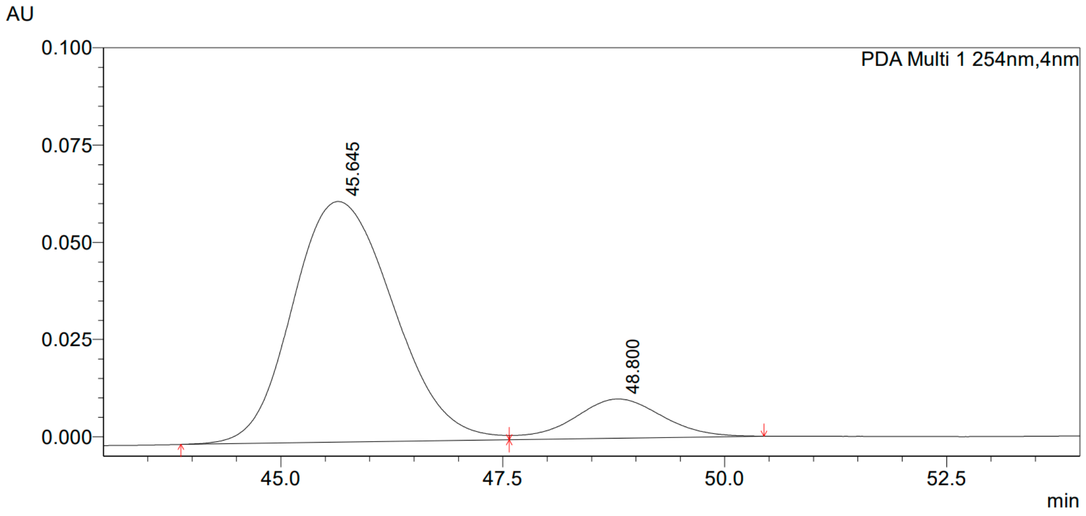

PDA Ch1 254nm

Peak#	Ret. Time	Area	Height	Area%
1	45.645	4909289	61931	87.462
2	48.800	703779	10080	12.538
Total		5613068	72011	100.000

(*Z*)-*N*-(6-(4-chlorophenyl)-3-(2-iodophenyl)-4-oxo-3,4-dihydro-2*H*-1,3-thiazin-2-ylidene)benzamide (3e)

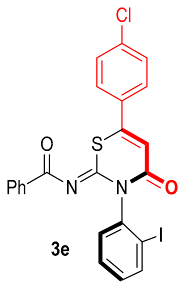

3e

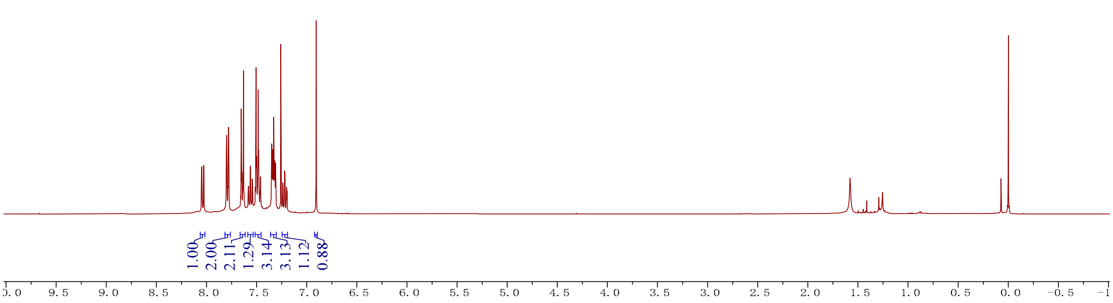

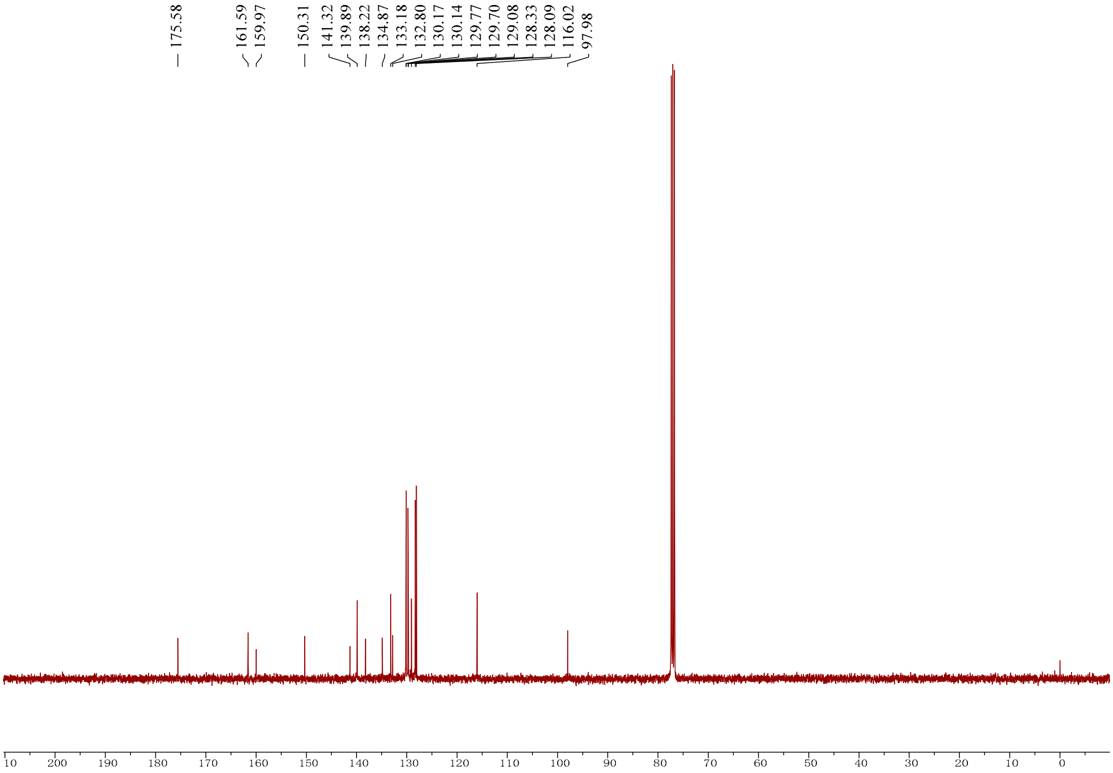

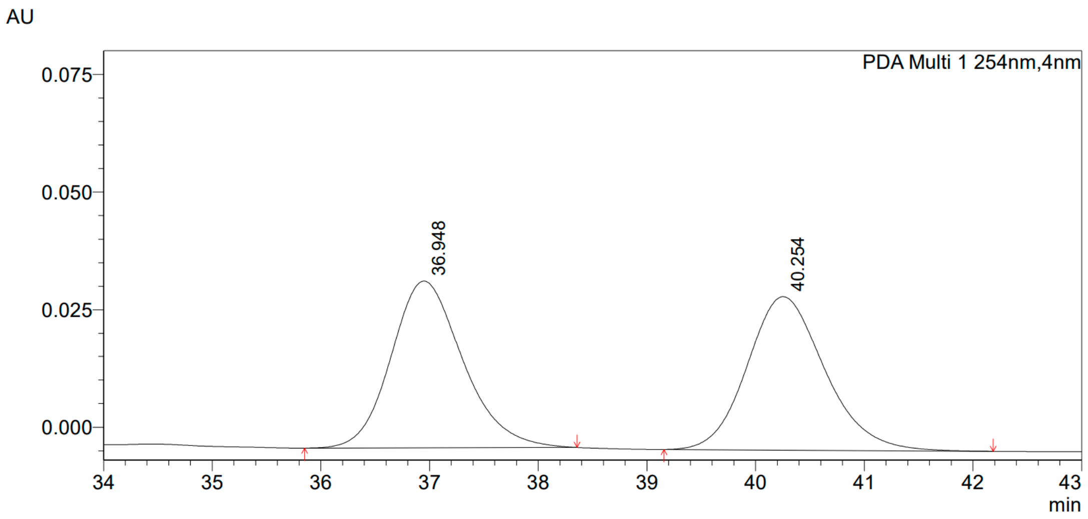

PDA Ch1 254nm

Peak#	Ret. Time	Area	Height	Area%
1	36.948	1631308	35489	50.121
2	40.254	1623460	32653	49.879
Total		3254768	68142	100.000

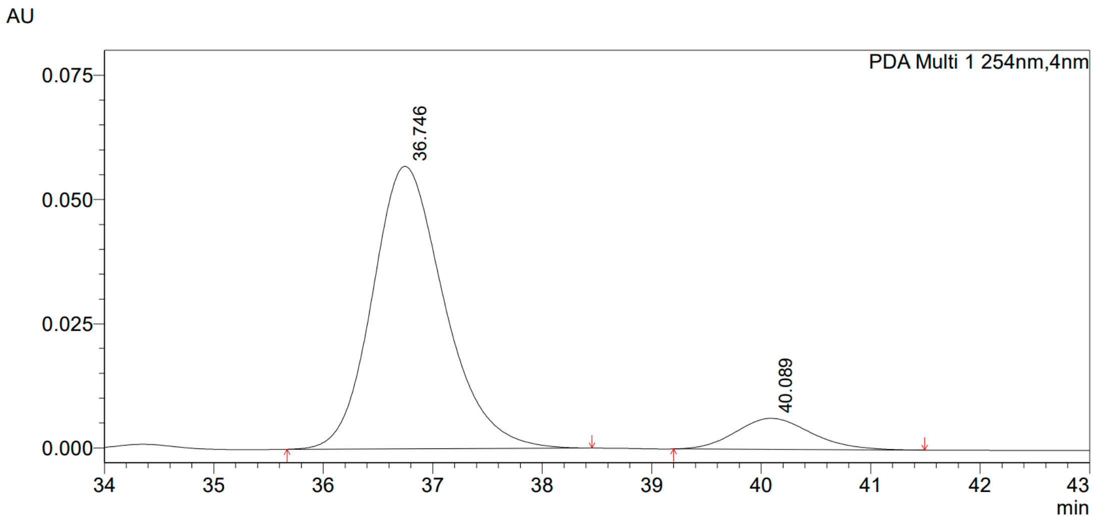

PDA Ch1 254nm

Peak#	Ret. Time	Area	Height	Area%
1	36.746	2597171	56881	89.680
2	40.089	298860	6231	10.320
Total		2896031	63112	100.000

(*Z*)-*N*-(6-(4-bromophenyl)-3-(2-iodophenyl)-4-oxo-3,4-dihydro-2*H*-1,3-thiazin-2-ylidene)benzamide (3f)

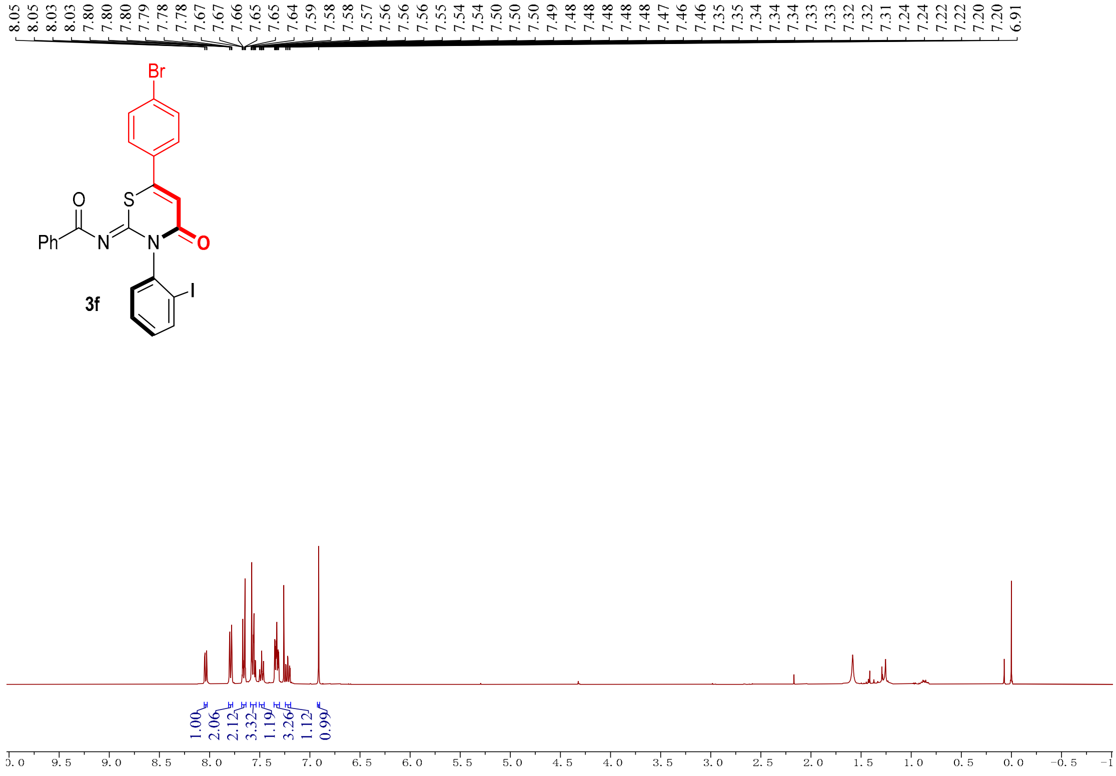

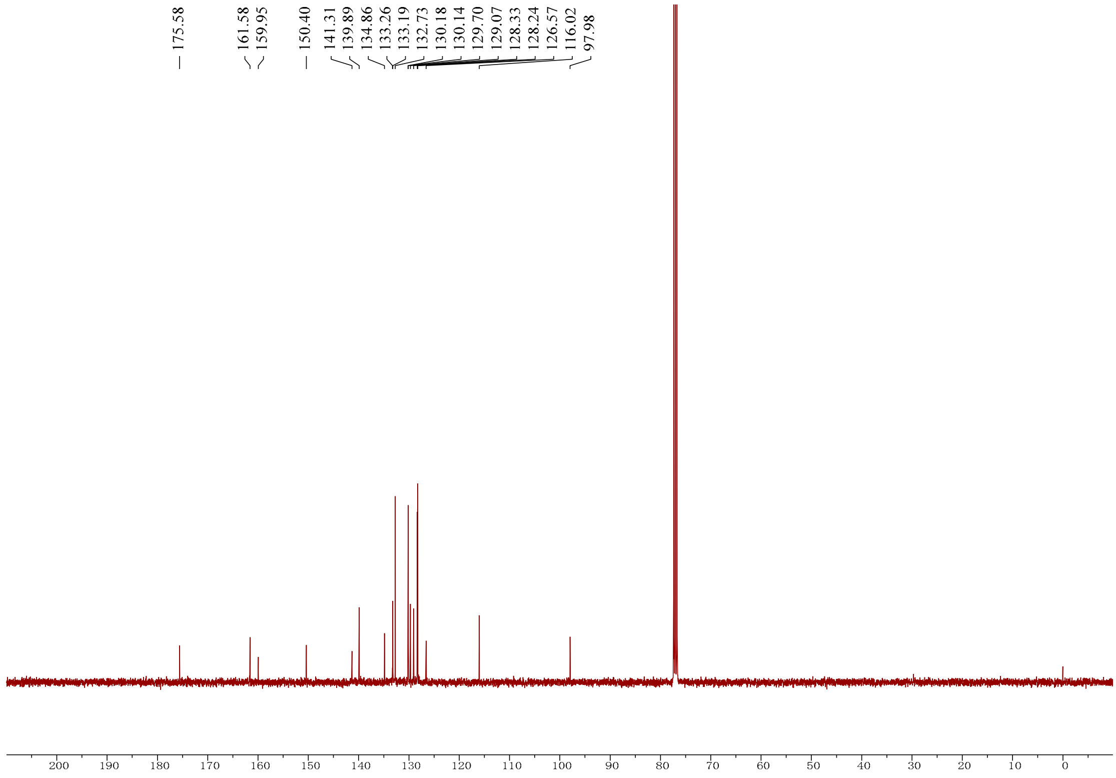

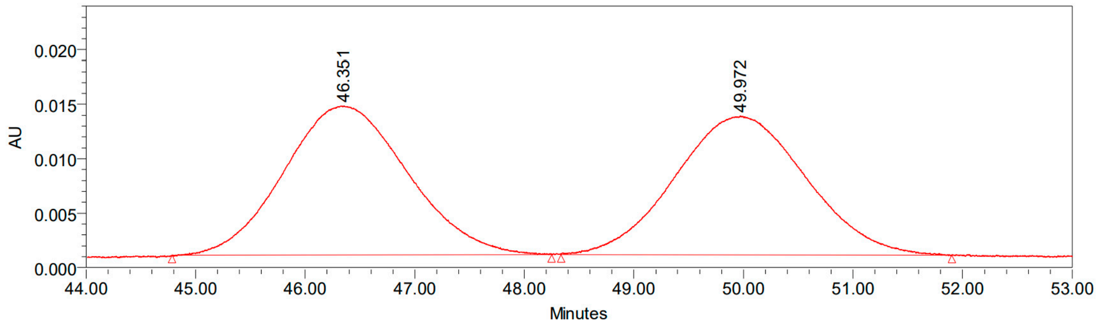

	RT	Area	% Area	Height
1	46.351	1075745	50.34	13697
2	49.972	1061017	49.66	12753

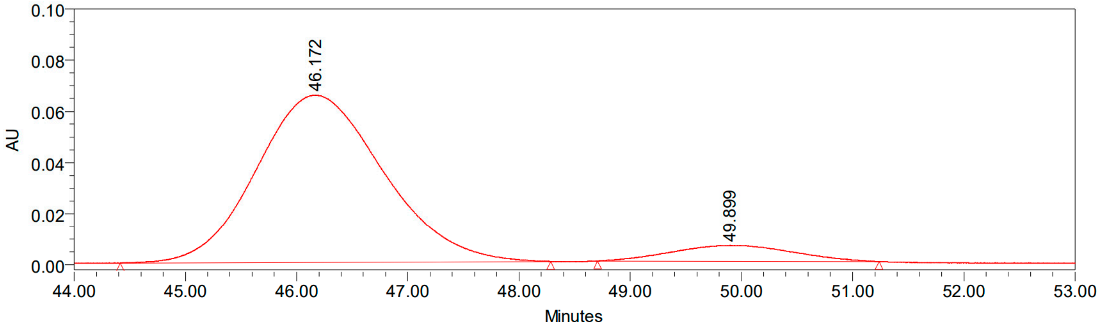

	RT	Area	% Area	Height
1	46.172	5179775	91.79	65276
2	49.899	463034	8.21	6142

(*Z*)-*N*-(3-(2-iodophenyl)-6-(3-methoxyphenyl)-4-oxo-3,4-dihydro-2*H*-1,3-thiazin-2-ylidene)benzamide (3g)

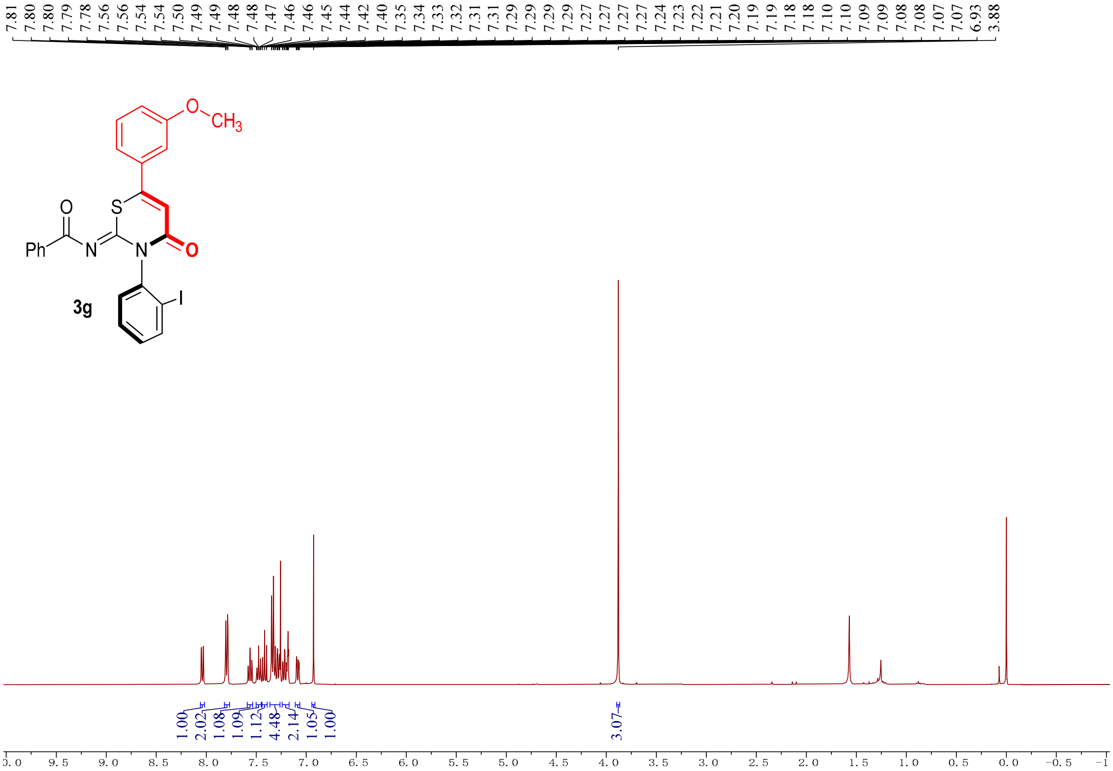

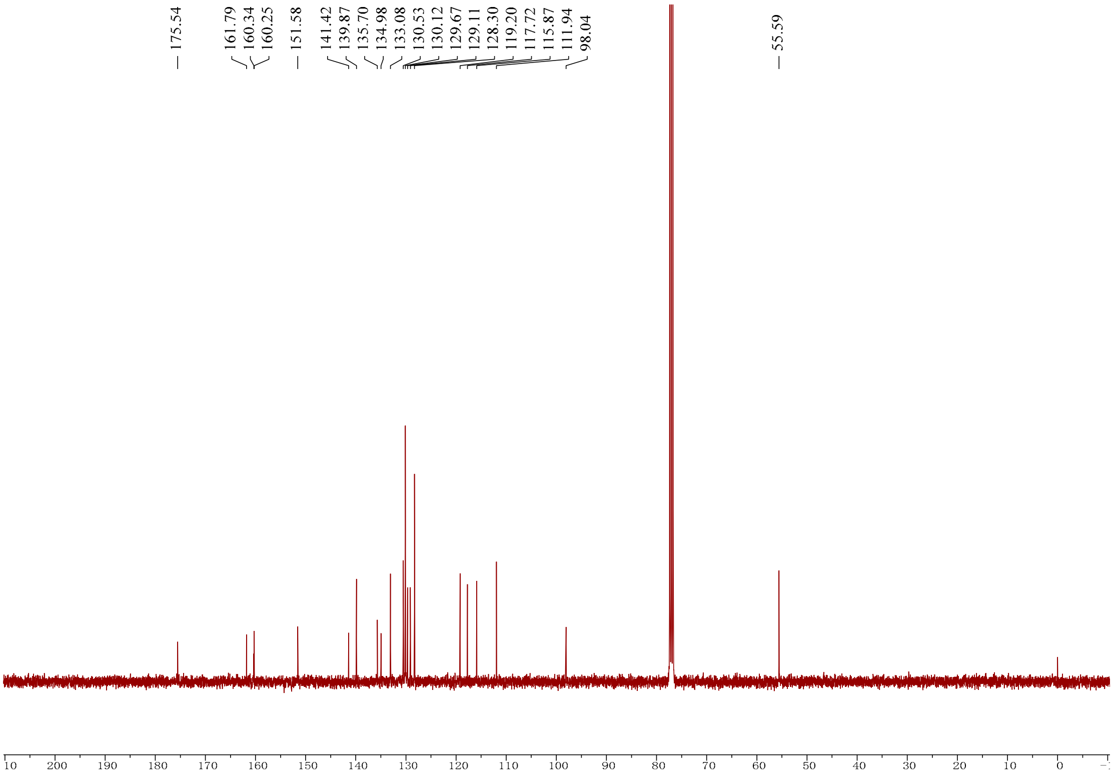

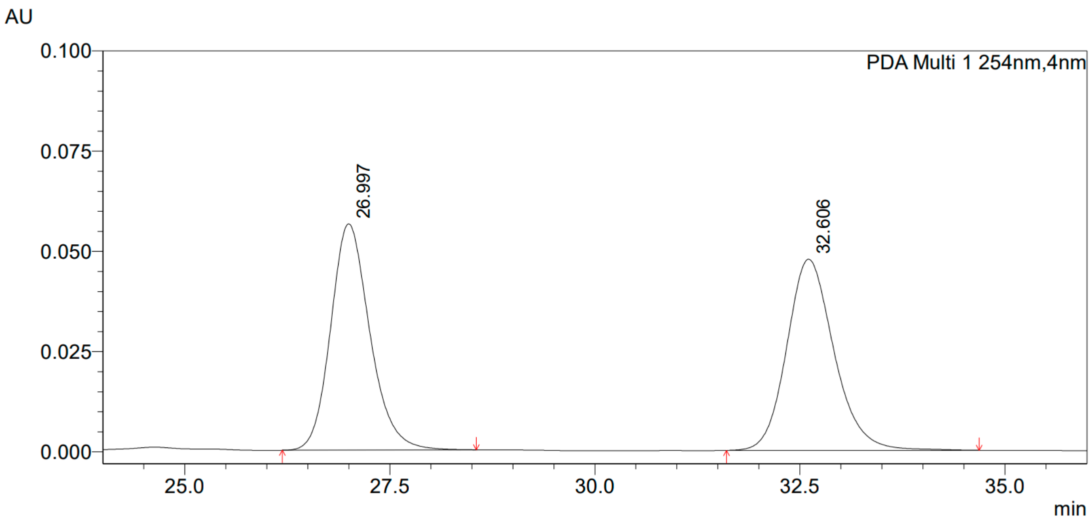

PDA Ch1 254nm

Peak#	Ret. Time	Area	Height	Area%
1	26.997	1880331	56480	49.536
2	32.606	1915548	47695	50.464
Total		3795879	104175	100.000

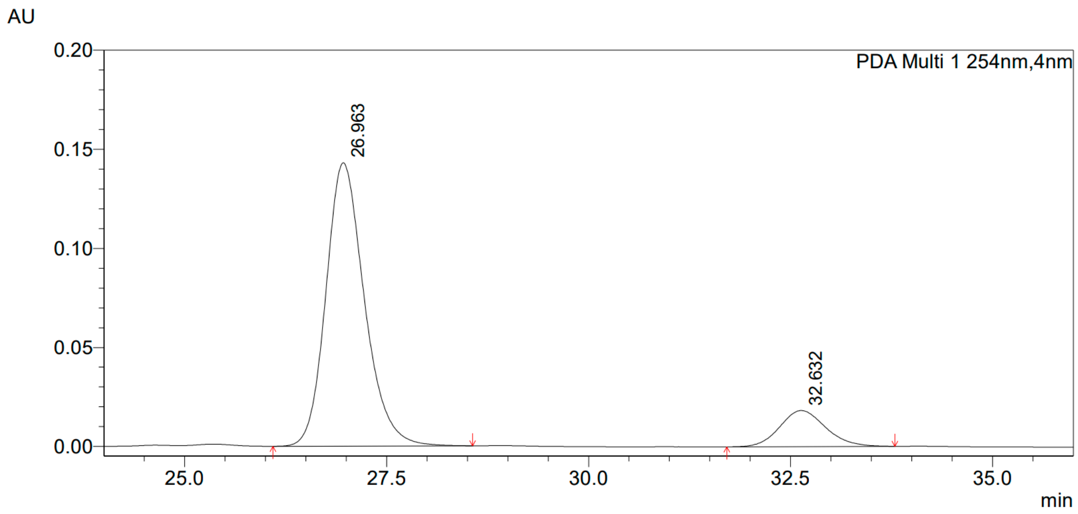

PDA Ch1 254nm

Peak#	Ret. Time	Area	Height	Area%
1	26.963	4743177	143156	86.833
2	32.632	719218	18206	13.167
Total		5462395	161362	100.000

(*Z*)-*N*-(3-(2-iodophenyl)-4-oxo-6-(*m*-tolyl)-3,4-dihydro-2*H*-1,3-thiazin-2-ylidene)benzamide (3h)

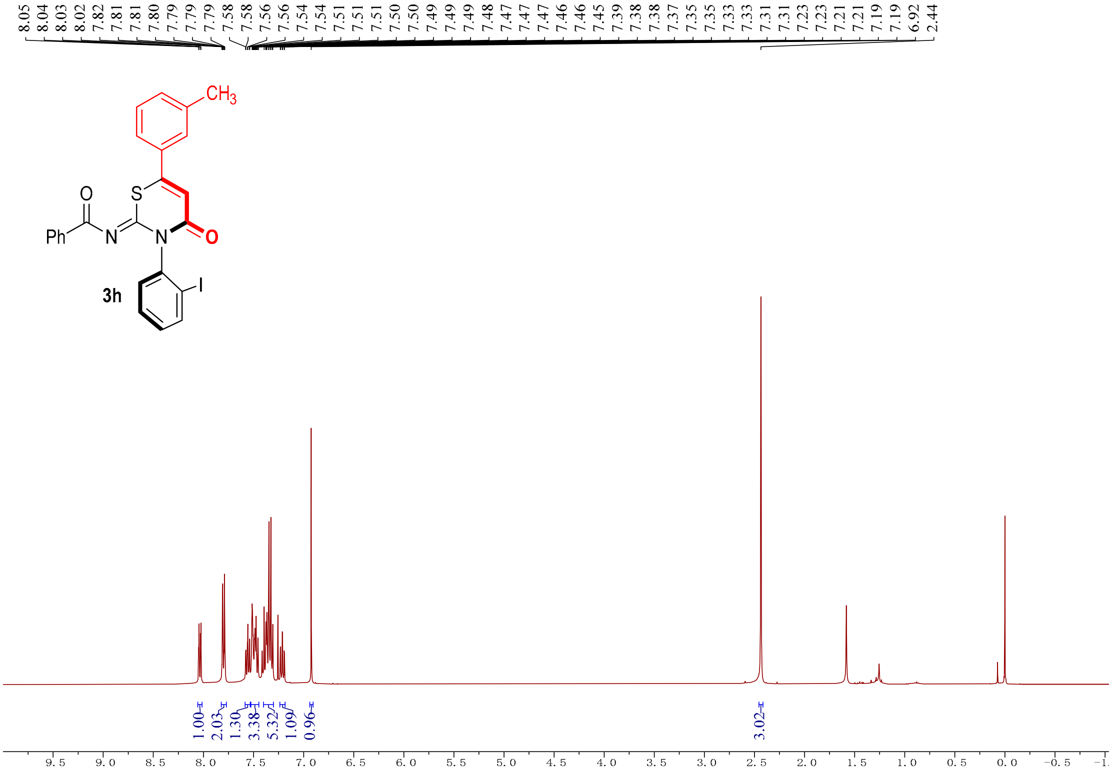

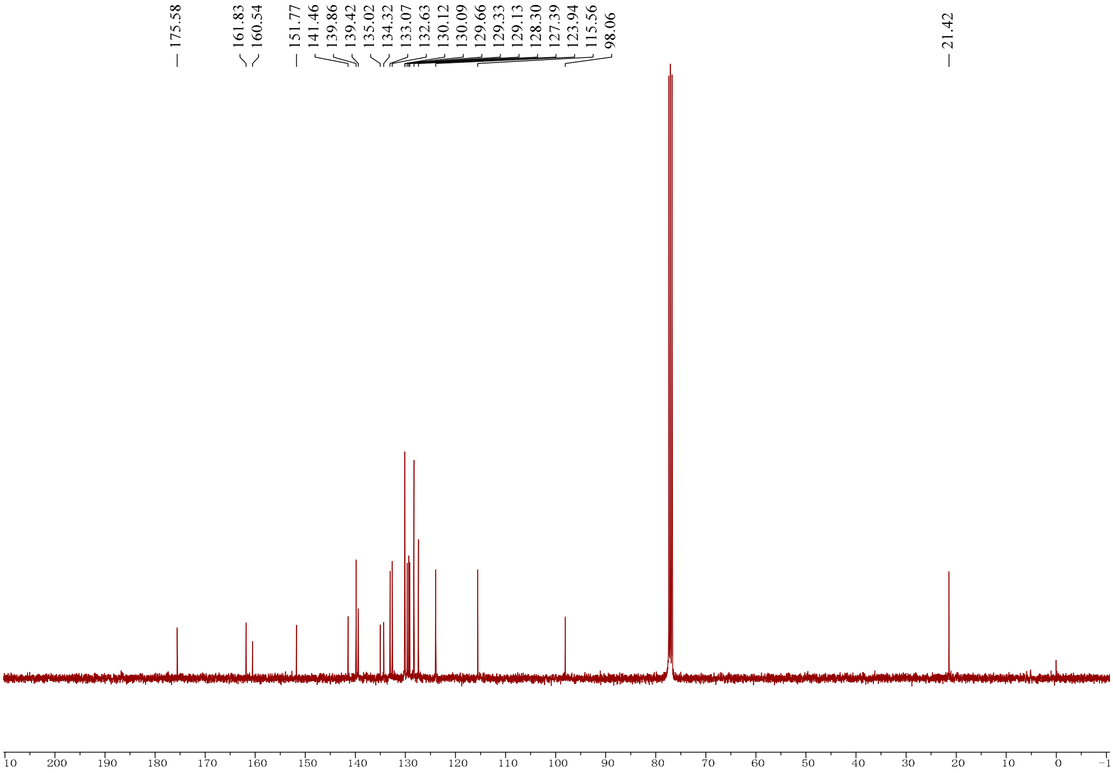

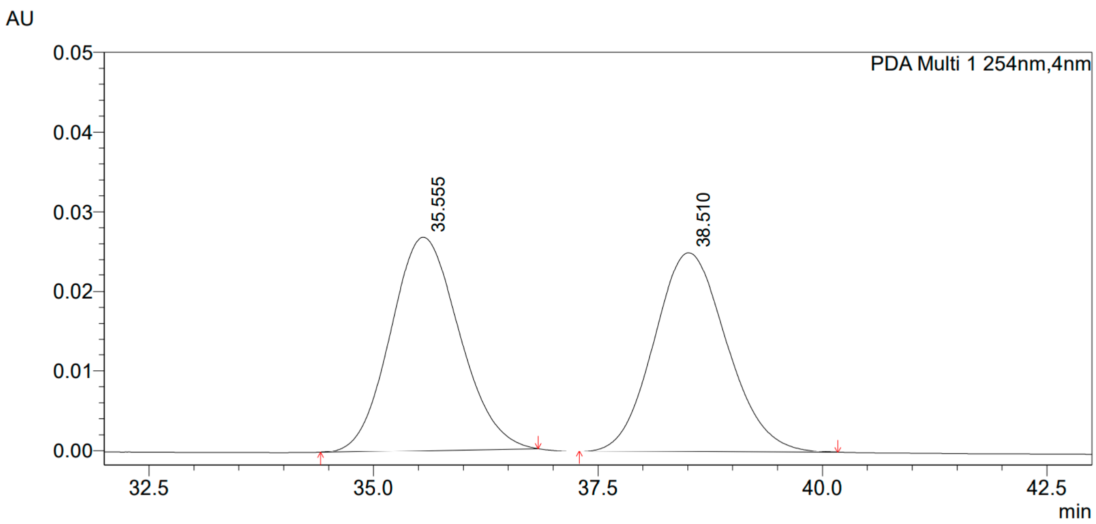

PDA Ch1 254nm

Peak#	Ret. Time	Area	Height	Area%
1	35.555	1433013	26833	50.256
2	38.510	1418423	25042	49.744
Total		2851436	51875	100.000

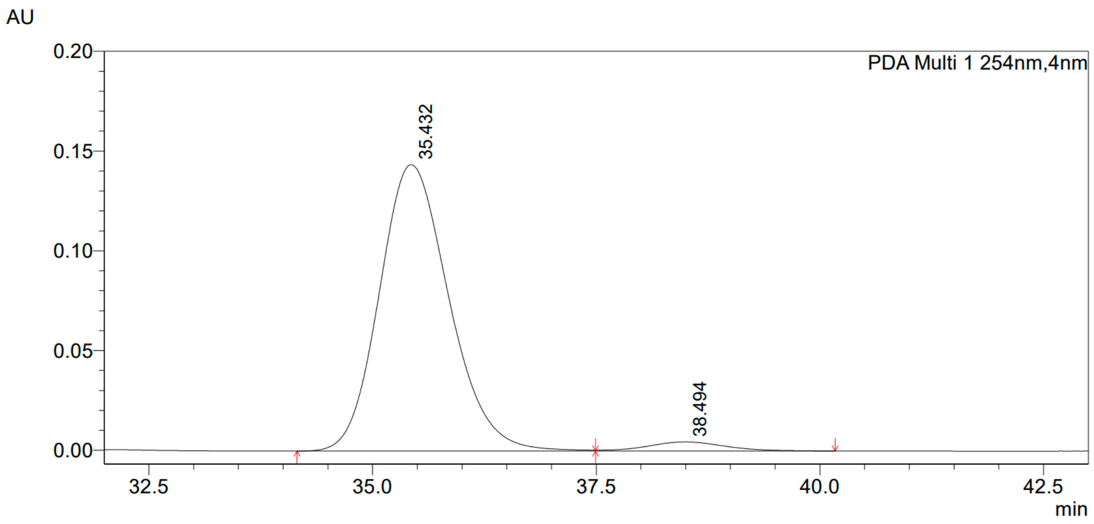

PDA Ch1 254nm

Peak#	Ret. Time	Area	Height	Area%
1	35.432	7777142	143595	96.529
2	38.494	279612	4602	3.471
Total		8056754	148197	100.000

(*Z*)-*N*-(6-(3-fluorophenyl)-3-(2-iodophenyl)-4-oxo-3,4-dihydro-2*H*-1,3-thiazin-2-ylidene)benzamide (3i)

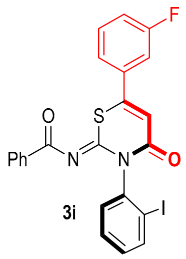

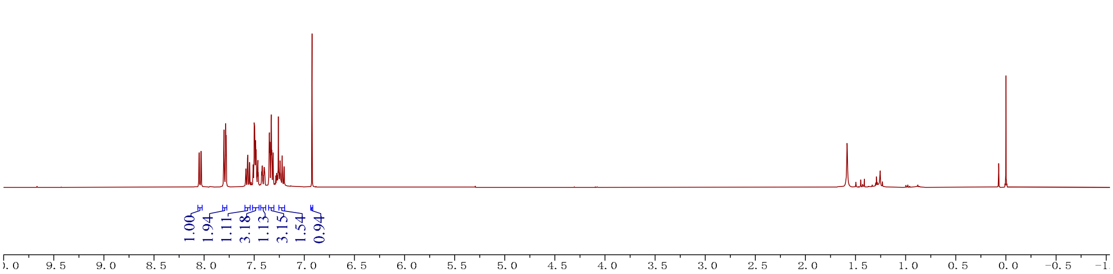

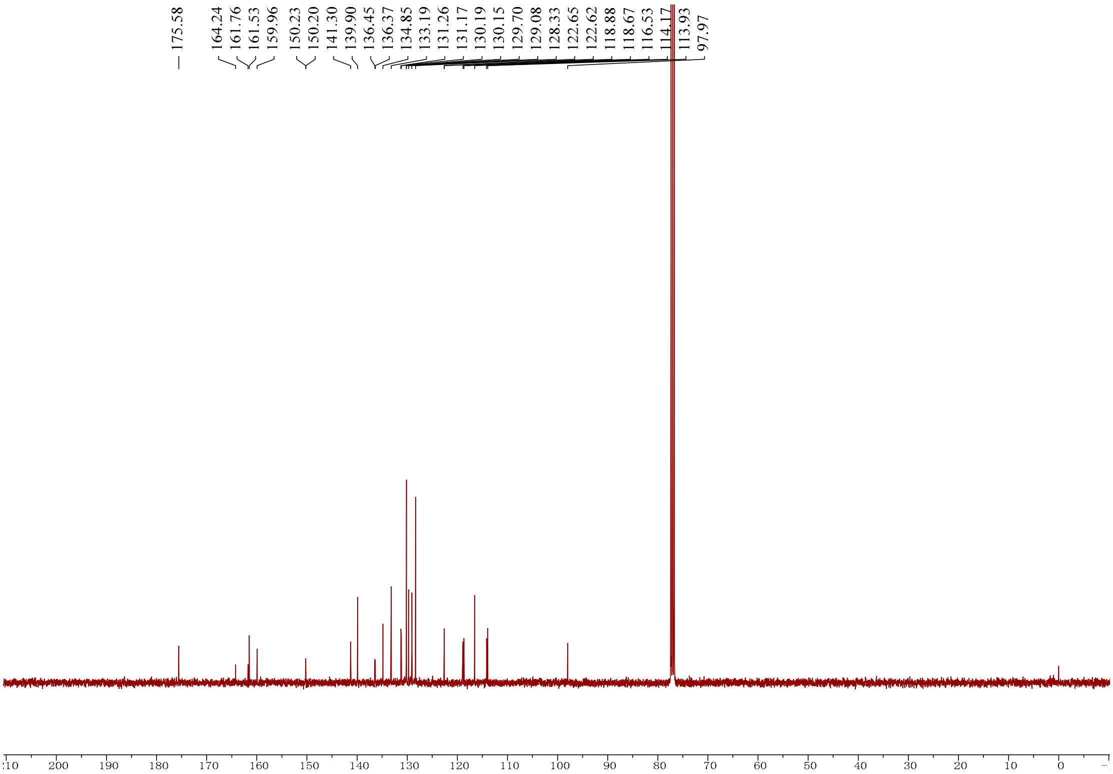

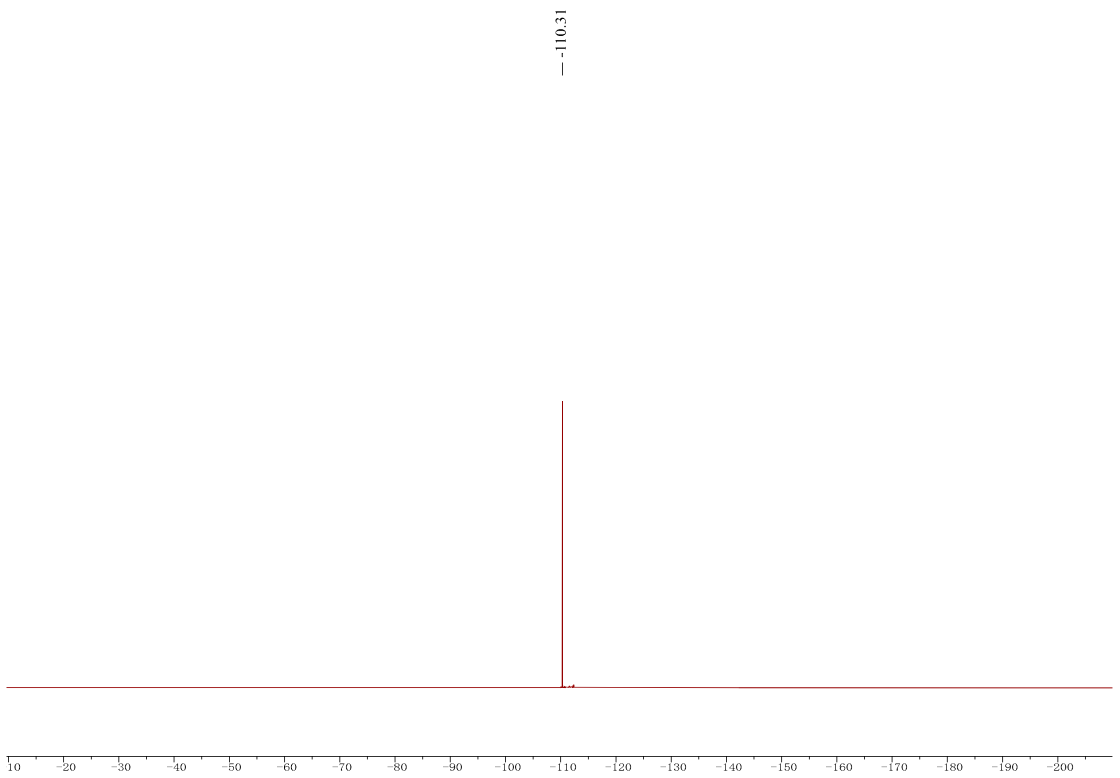
-110.31
10
-20
-30
-40
-50
-60
-70
-80
-90
-100
-110
-120
-130
-140
-150
-160
-170
-180
-190
-200

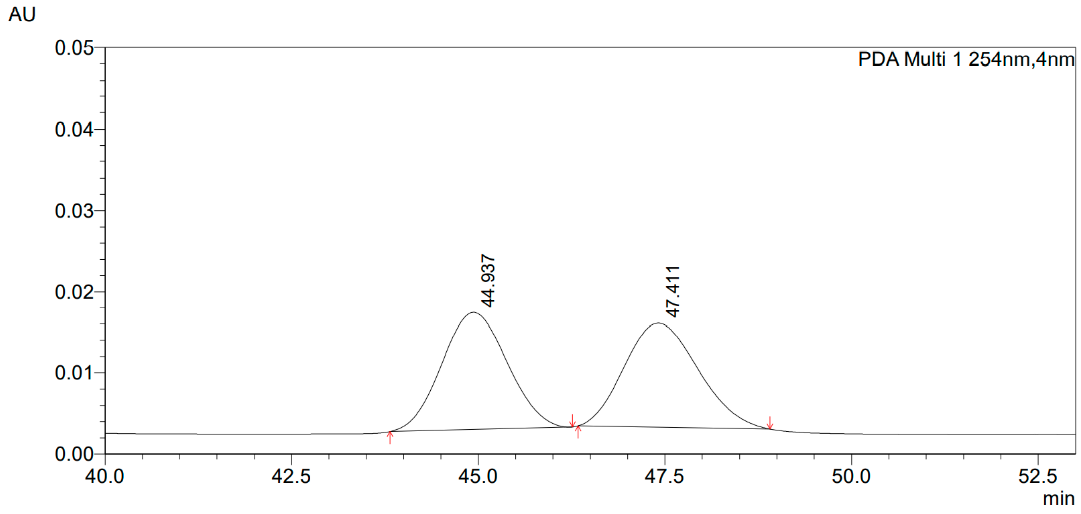

PDA Ch1 254nm

Peak#	Ret. Time	Area	Height	Area%
1	44.937	875684	14385	49.877
2	47.411	880004	12797	50.123
Total		1755689	27183	100.000

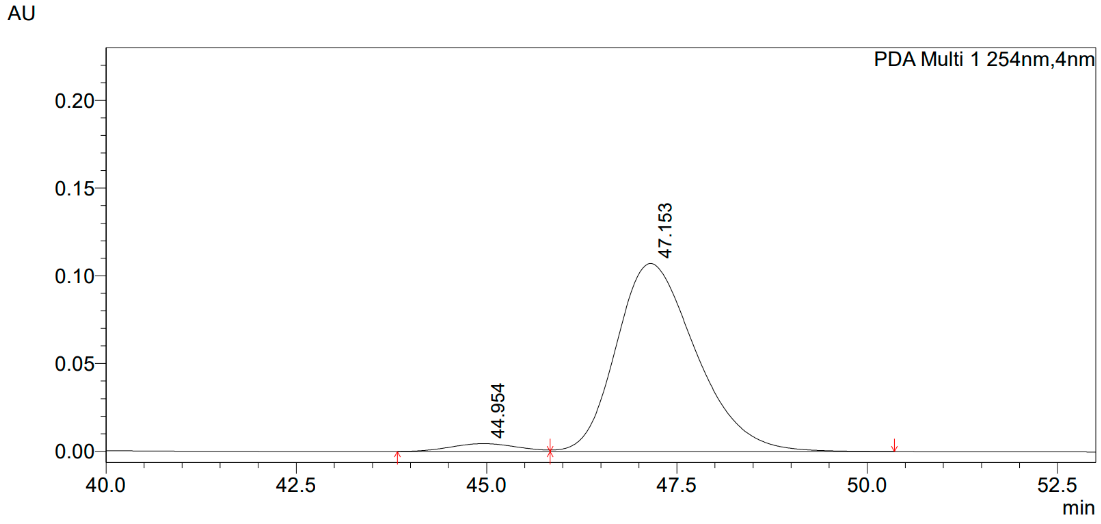

PDA Ch1 254nm

Peak#	Ret. Time	Area	Height	Area%
1	44.954	273102	4498	3.373
2	47.153	7823978	107175	96.627
Total		8097080	111673	100.000

(*Z*)-*N*-(3-(2-iodophenyl)-4-oxo-6-(*o*-tolyl)-3,4-dihydro-2*H*-1,3-thiazin-2-ylidene)benzamide (3j)

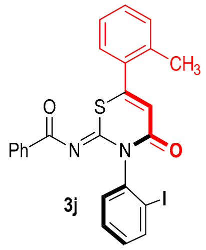

3j

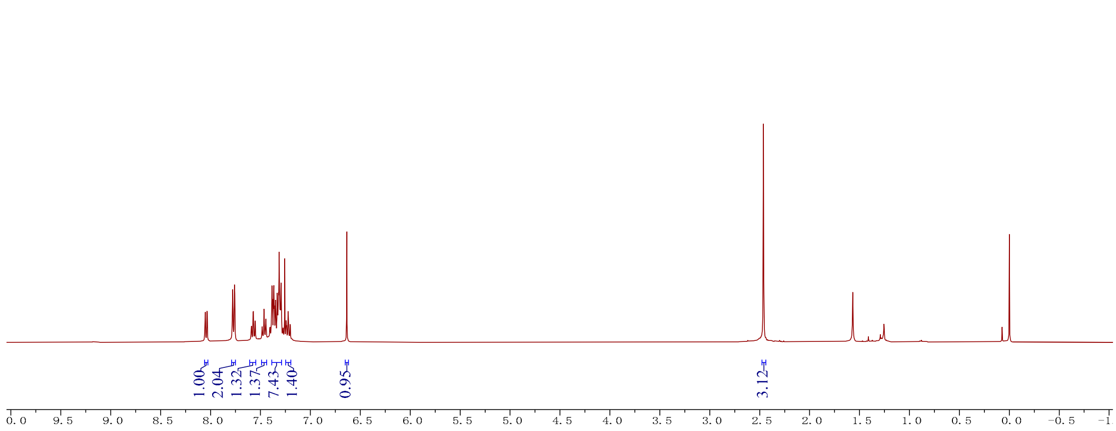

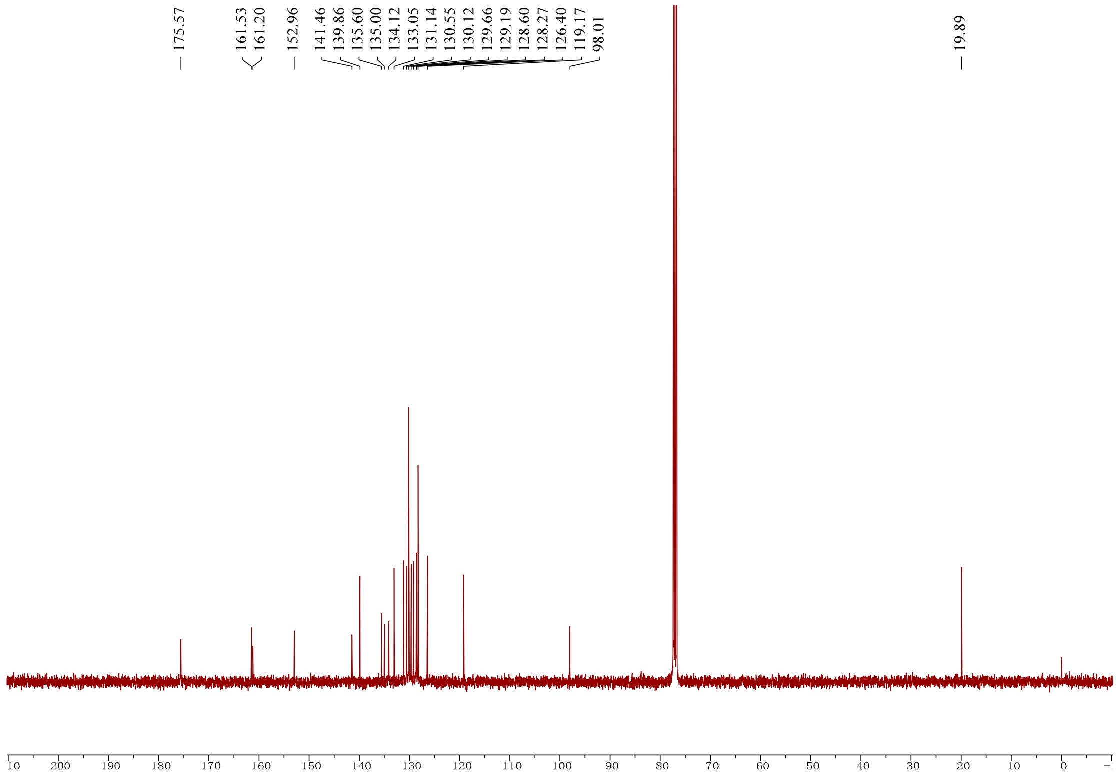

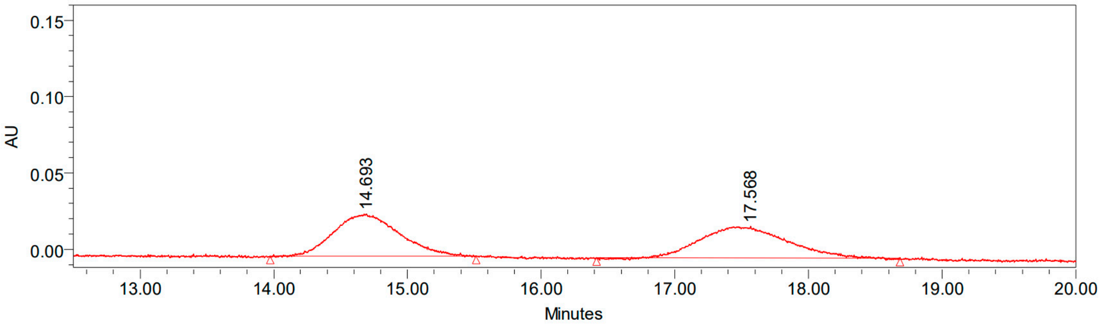

	RT	Area	% Area	Height
1	14.693	943802	50.22	27800
2	17.568	935373	49.78	20405

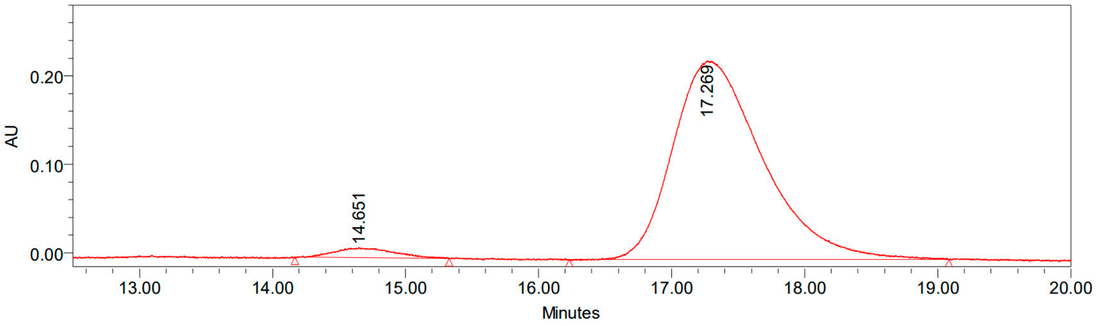

	RT	Area	% Area	Height
1	14.651	331344	3.05	11039
2	17.269	10543287	96.95	224576

(*Z*)-*N*-(6-(2-fluorophenyl)-3-(2-iodophenyl)-4-oxo-3,4-dihydro-2*H*-1,3-thiazin-2-ylidene)benzamide (3k)

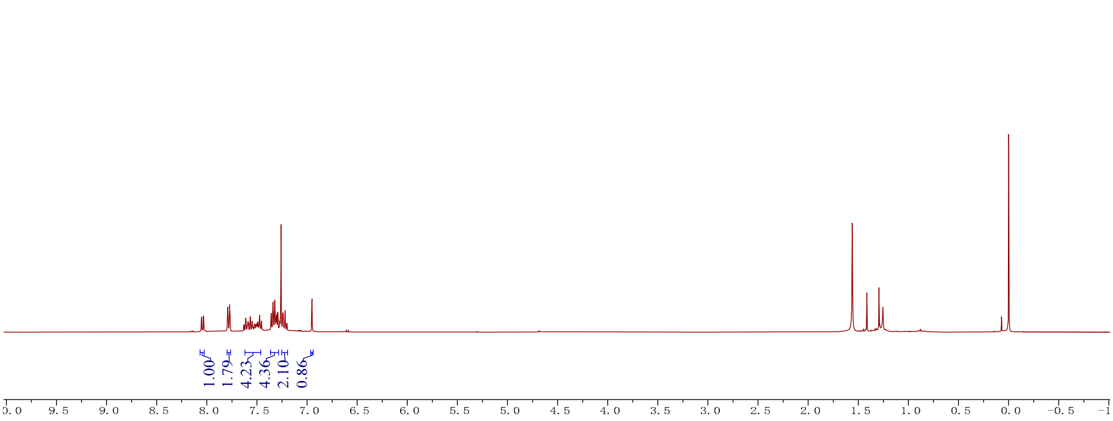

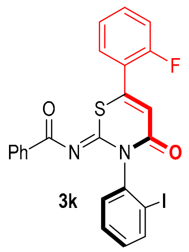

3k

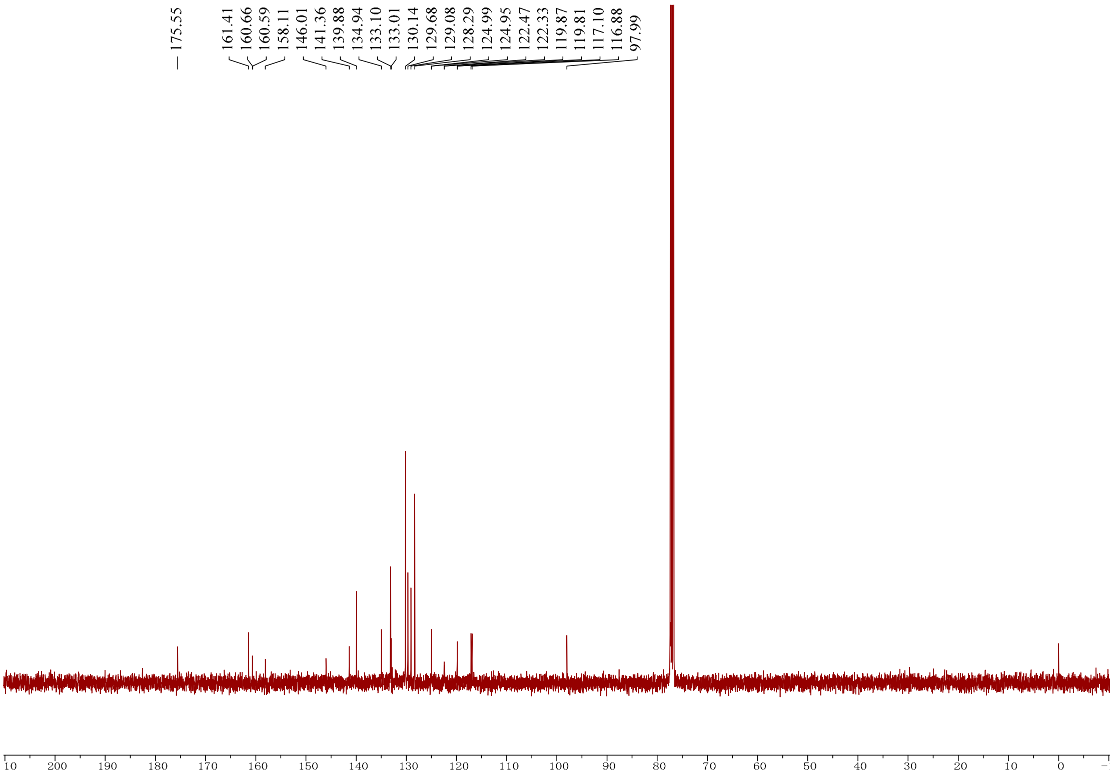

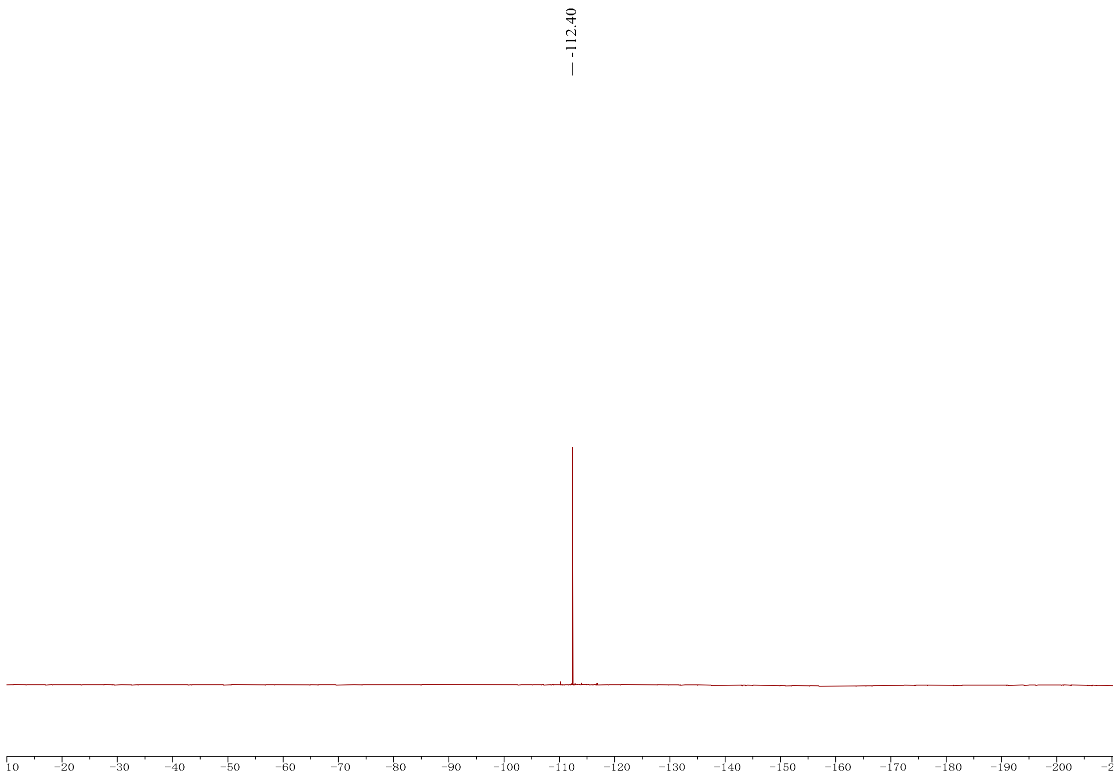
-112.40
10
-20
-30
-40
-50
-60
-70
-80
-90
-100
-110
-120
-130
-140
-150
-160
-170
-180
-190
-200

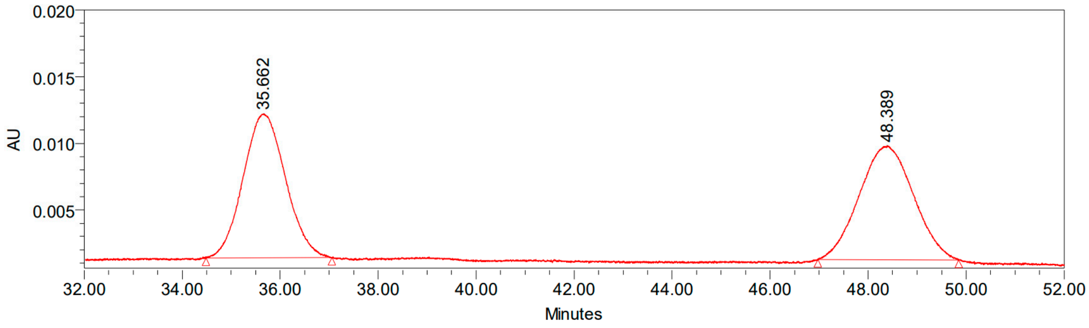

	RT	Area	% Area	Height
1	35.662	649872	49.65	10812
2	48.389	659095	50.35	8572

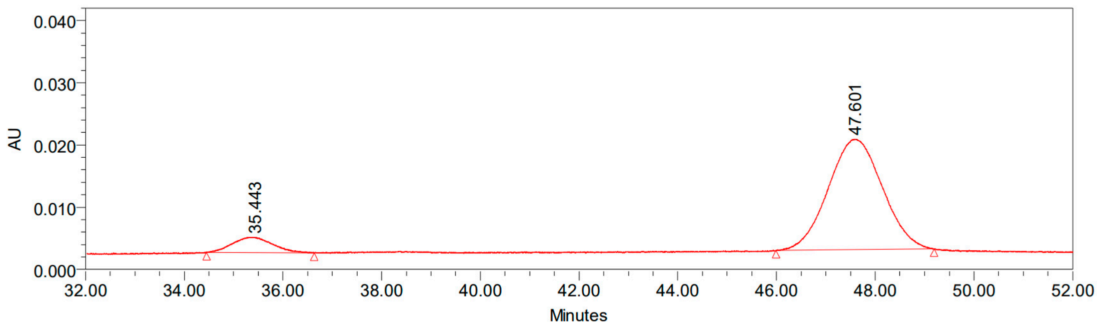

	RT	Area	% Area	Height
1	35.443	135765	9.11	2437
2	47.601	1354225	90.89	17727

(*Z*)-*N*-(6-(2-chlorophenyl)-3-(2-iodophenyl)-4-oxo-3,4-dihydro-2*H*-1,3-thiazin-2-ylidene)benzamide (3l)

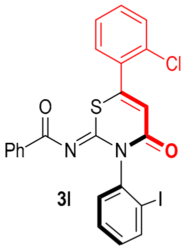

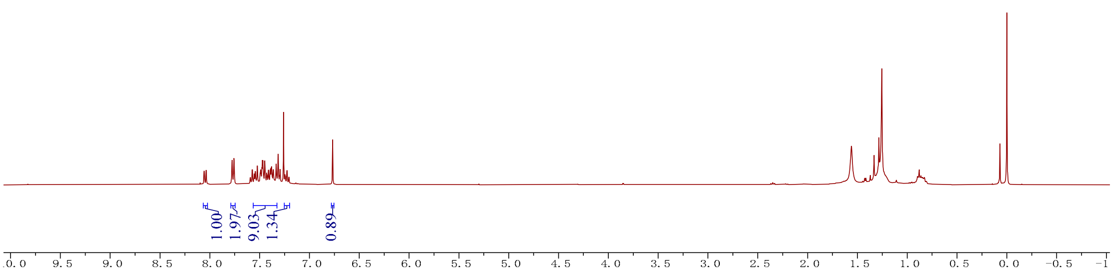

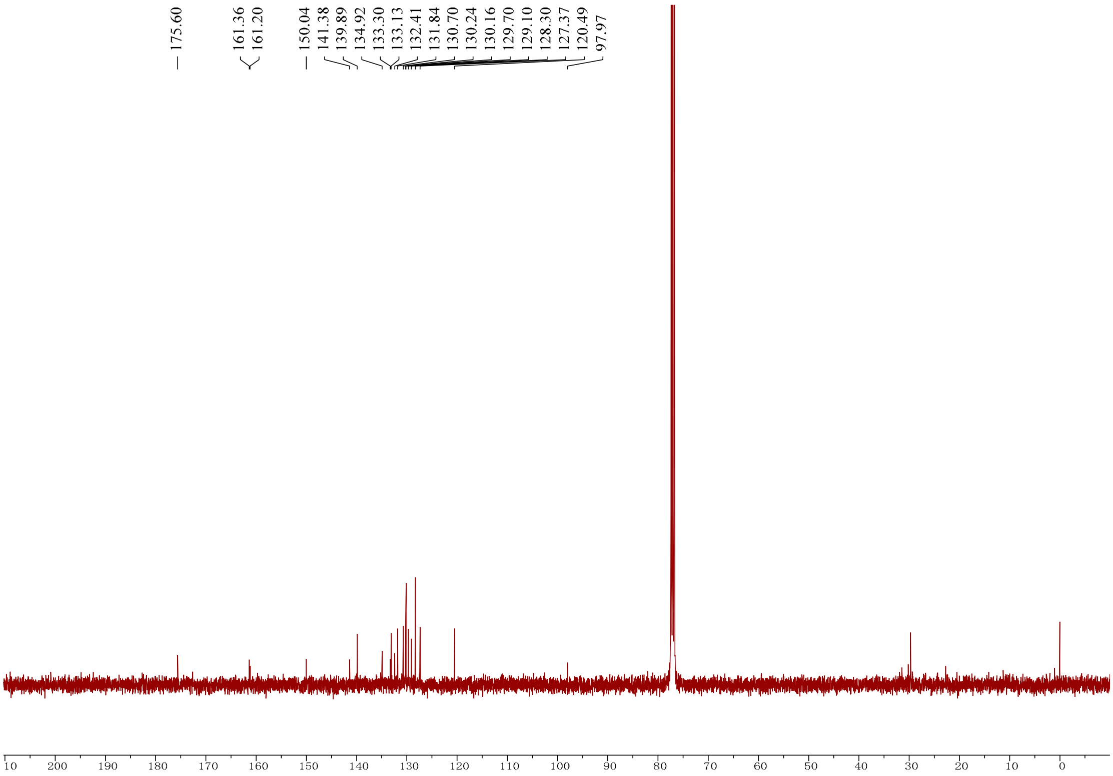

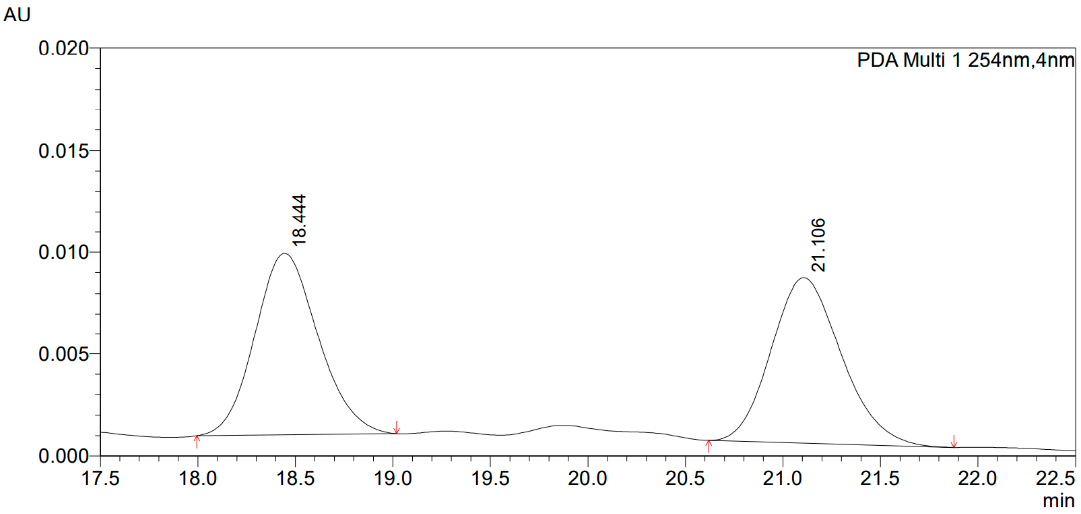

PDA Ch1 254nm

Peak#	Ret. Time	Area	Height	Area%
1	18.444	201532	8909	49.712
2	21.106	203863	8123	50.288
Total		405395	17031	100.000

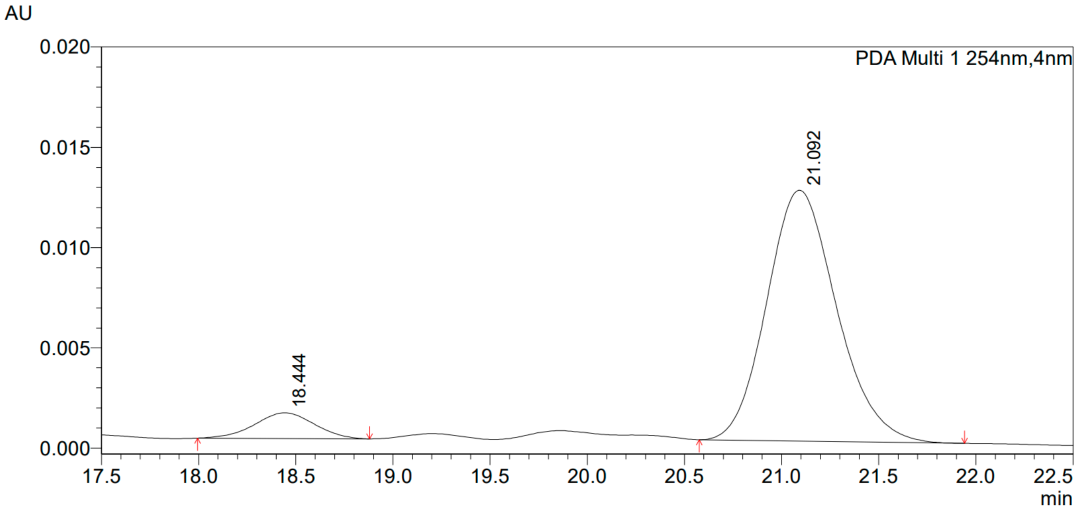

PDA Ch1 254nm

Peak#	Ret. Time	Area	Height	Area%
1	18.444	27290	1277	8.012
2	21.092	313350	12517	91.988
Total		340640	13794	100.000

(*Z*)-*N*-(3-(2-iodophenyl)-6-(naphthalen-1-yl)-4-oxo-3,4-dihydro-2*H*-1,3-thiazin-2-ylidene)benzamide (3m)

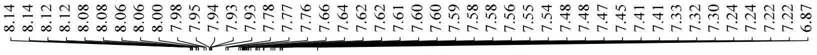

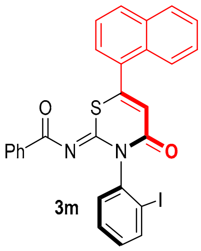

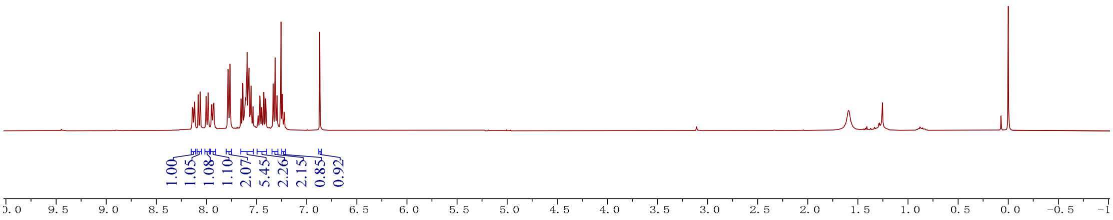

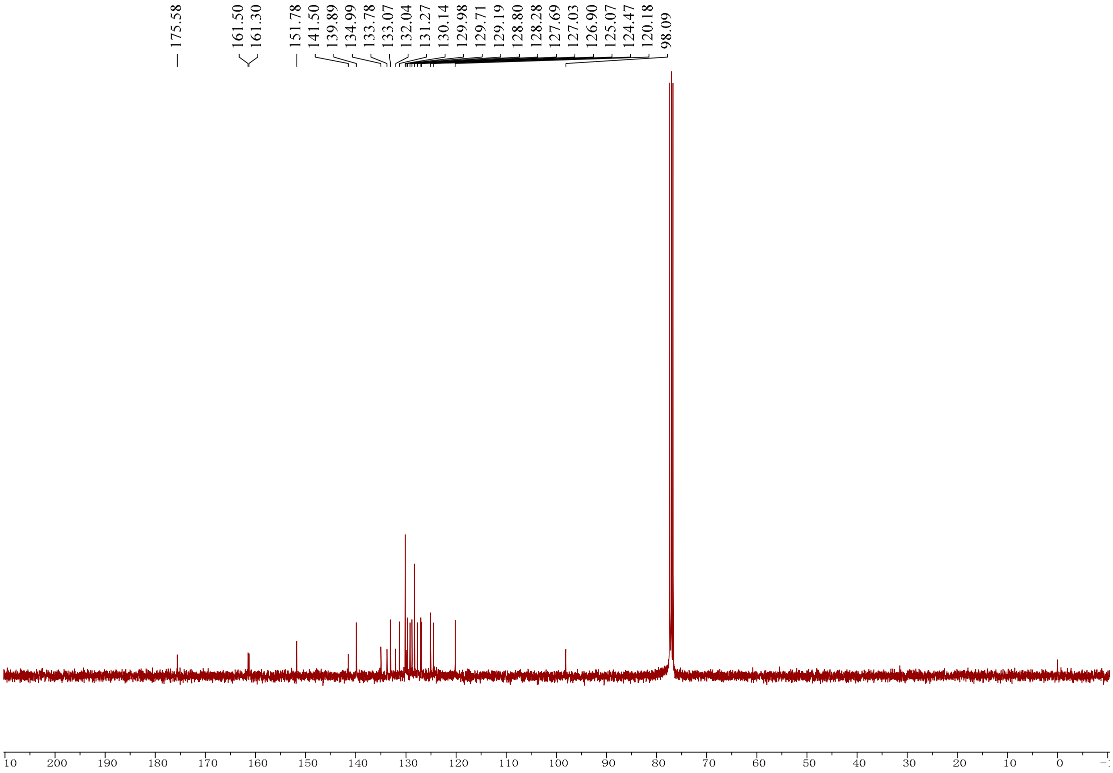

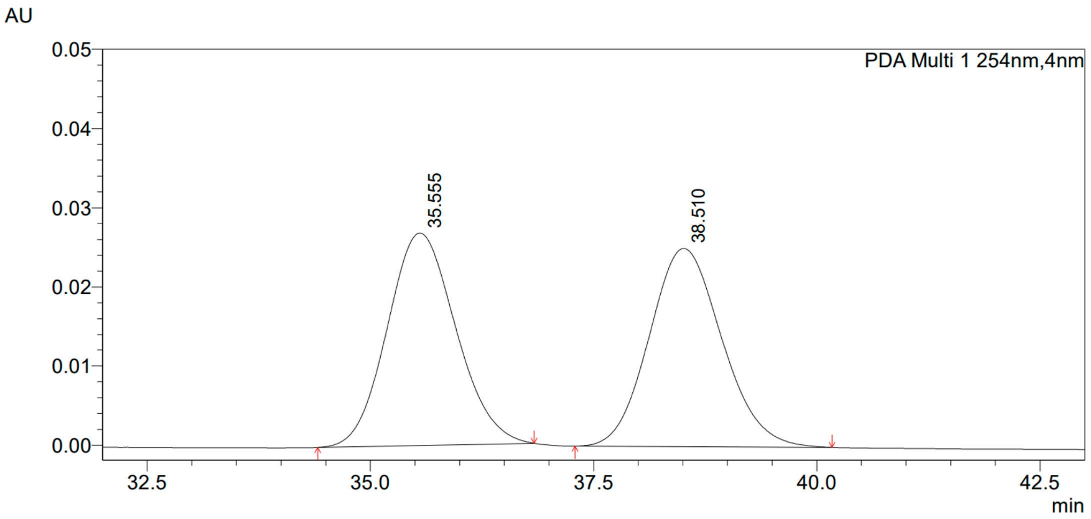

PDA Ch1 254nm

Peak#	Ret. Time	Area	Height	Area%
1	35.555	1433013	26833	50.256
2	38.510	1418423	25042	49.744
Total		2851436	51875	100.000

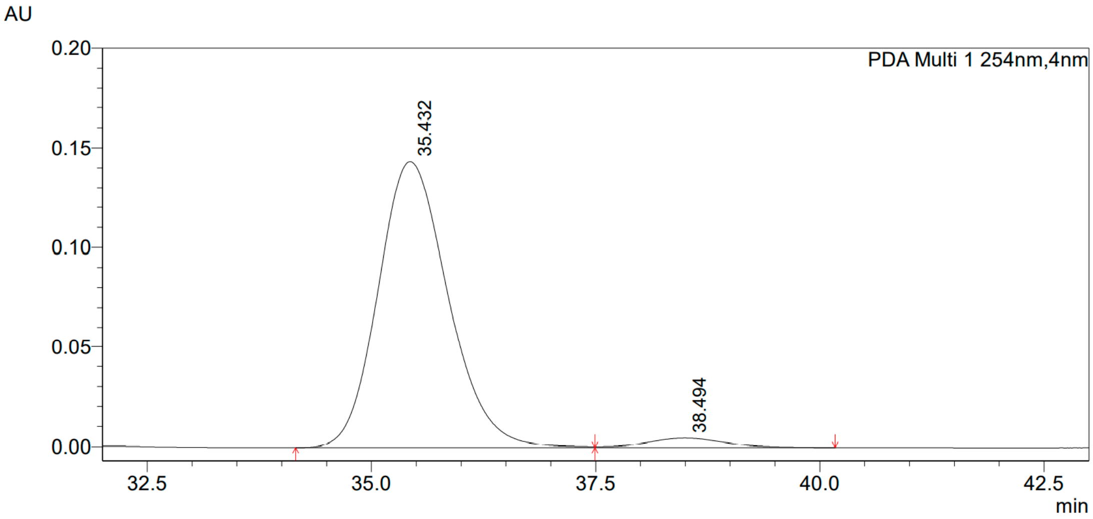

PDA Ch1 254nm

Peak#	Ret. Time	Area	Height	Area%
1	35.432	7777142	143595	96.529
2	38.494	279612	4602	3.471
Total		8056754	148197	100.000

(*Z*)-*N*-(3-(2-iodophenyl)-4-oxo-6-(thiophen-2-yl)-3,4-dihydro-2*H*-1,3-thiazin-2-ylidene)benzamide (3n)

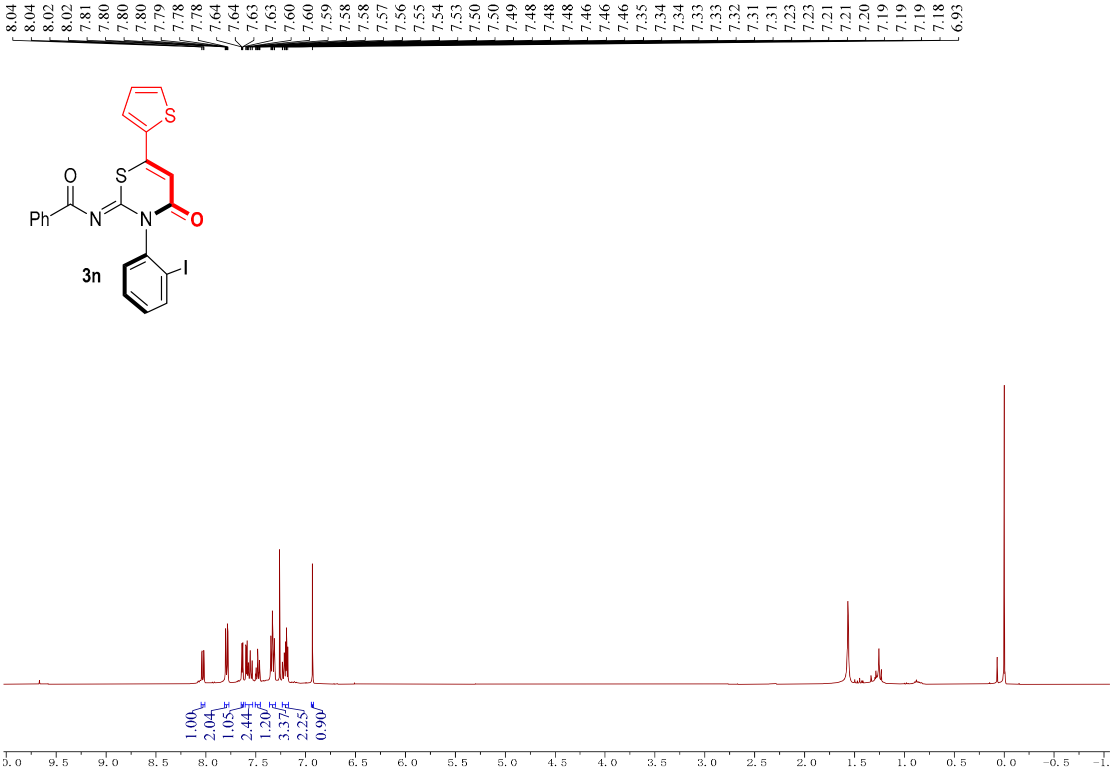

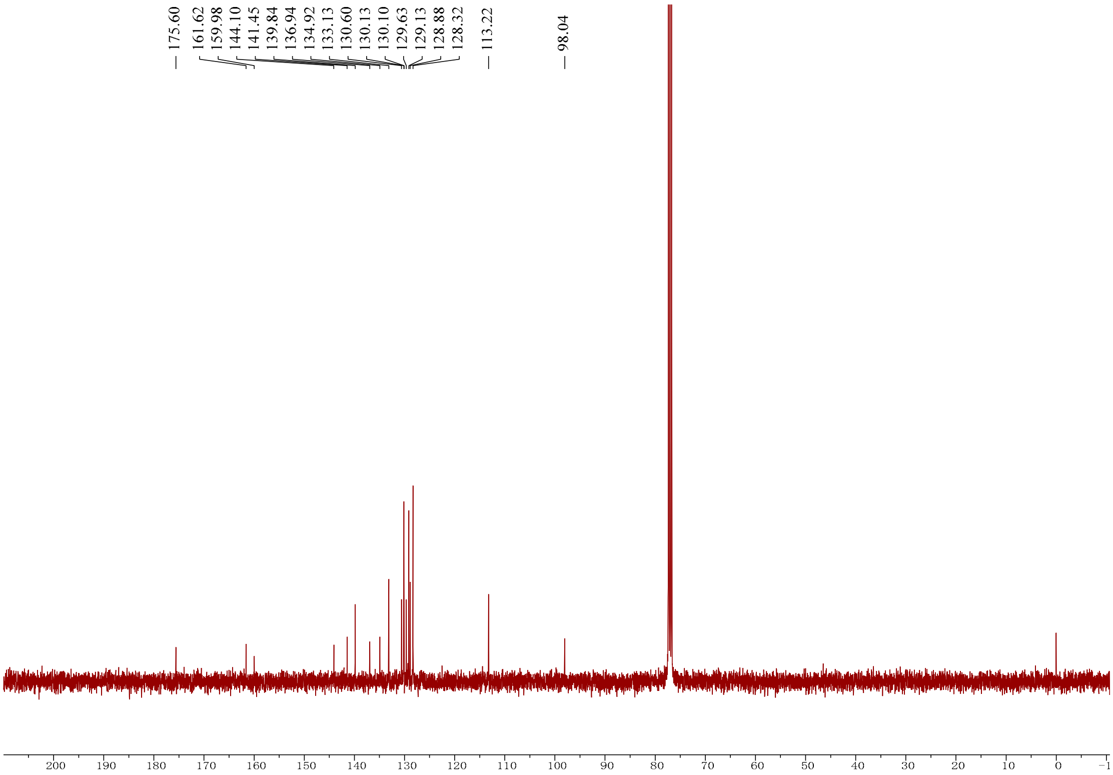

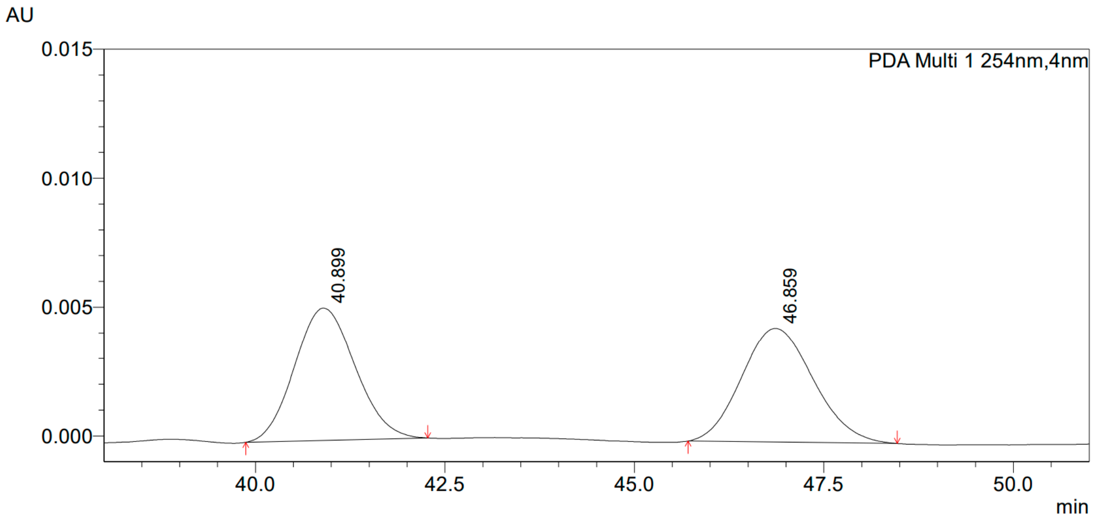

PDA Ch1 254nm

Peak#	Ret. Time	Area	Height	Area%
1	40.899	285959	5142	49.578
2	46.859	290829	4414	50.422
Total		576788	9556	100.000

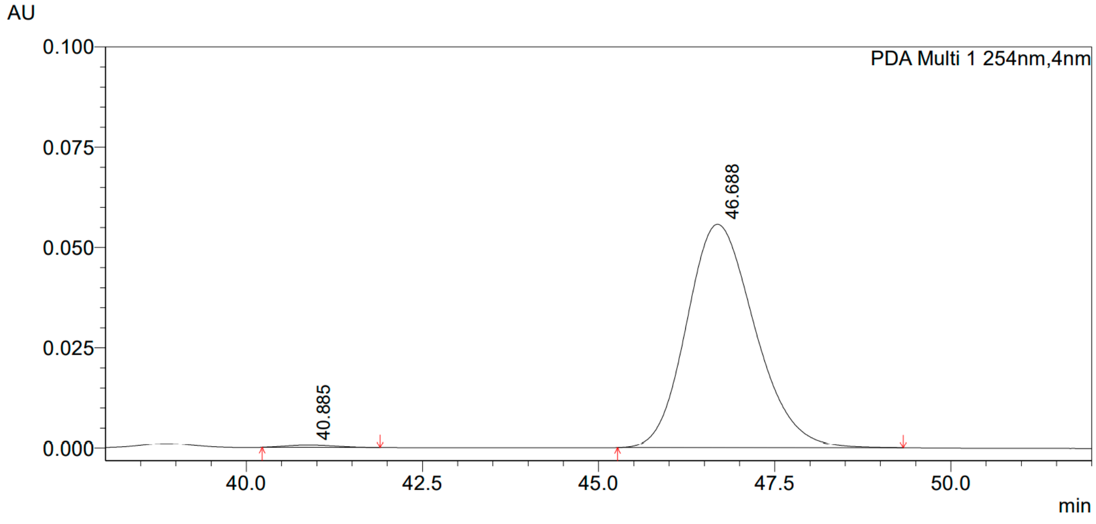

PDA Ch1 254nm

Peak#	Ret. Time	Area	Height	Area%
1	40.885	29445	603	0.779
2	46.688	3752783	55674	99.221
Total		3782228	56277	100.000

(*Z*)-*N*-(3-(2-iodophenyl)-4-oxo-6-phenyl-3,4-dihydro-2*H*-1,3-thiazin-2-ylidene)-4-methylbenzamide (4a)

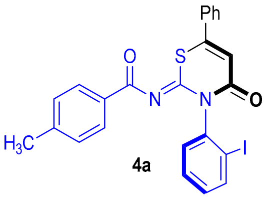

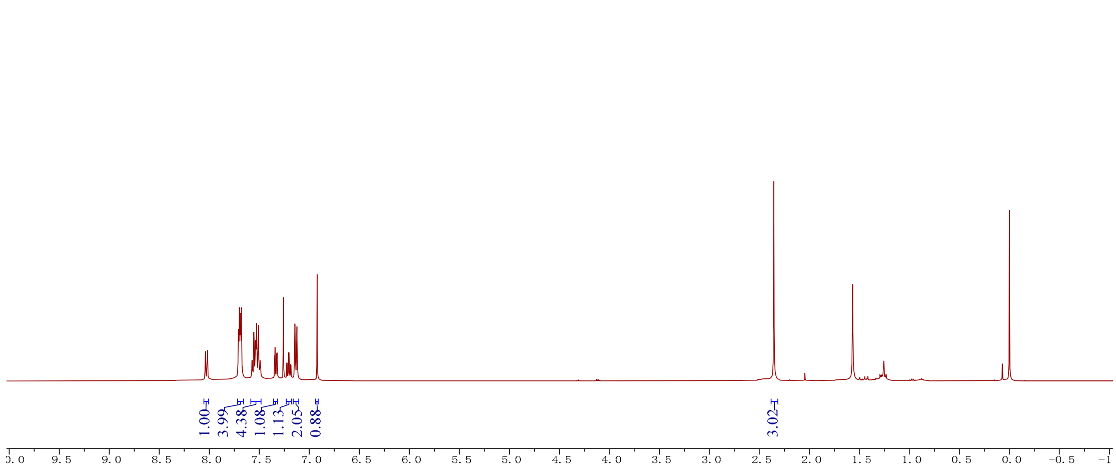

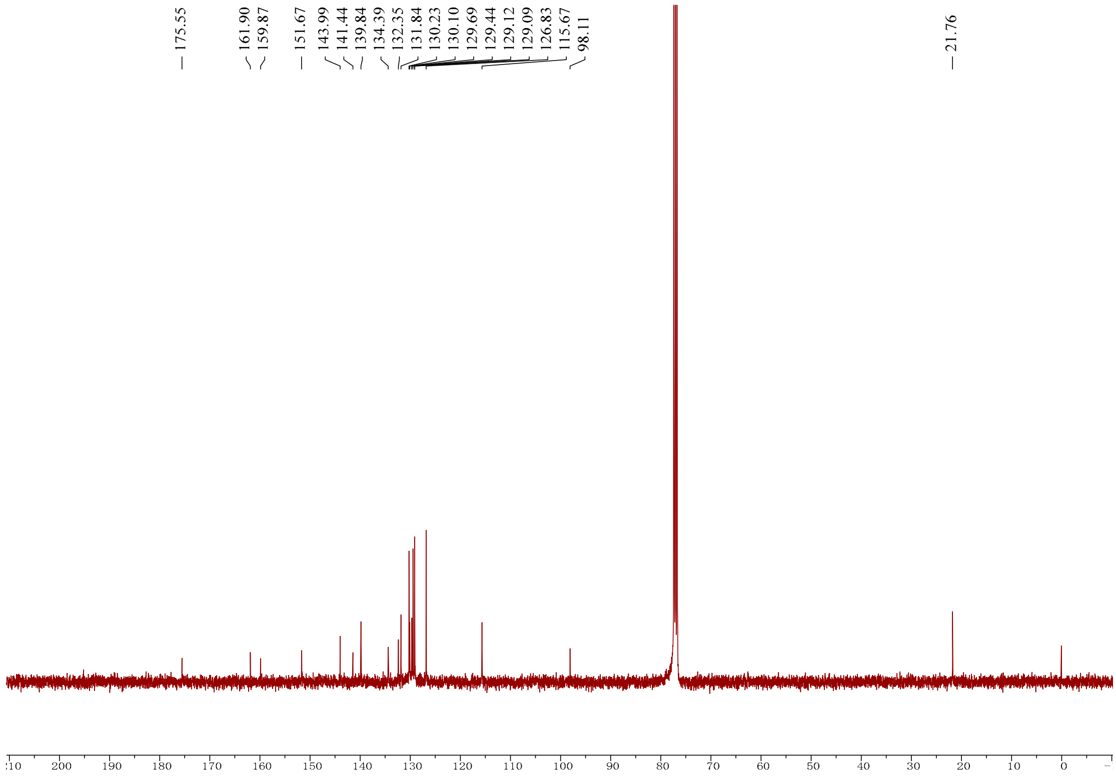

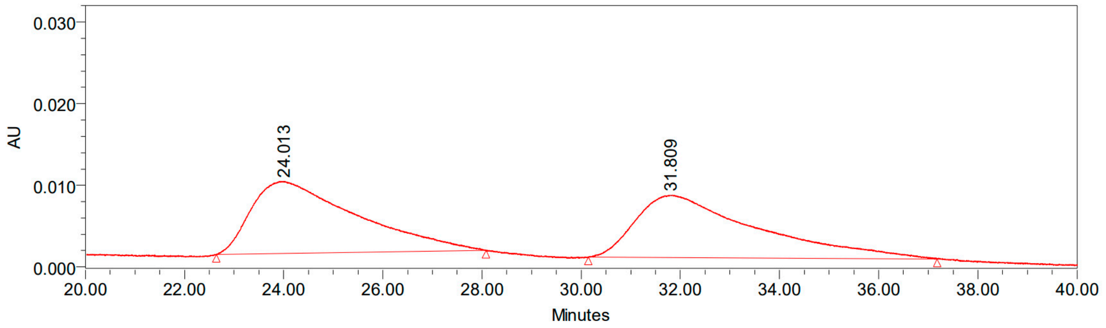

	RT	Area	% Area	Height
1	24.013	1301574	50.19	8822
2	31.809	1291545	49.81	7629

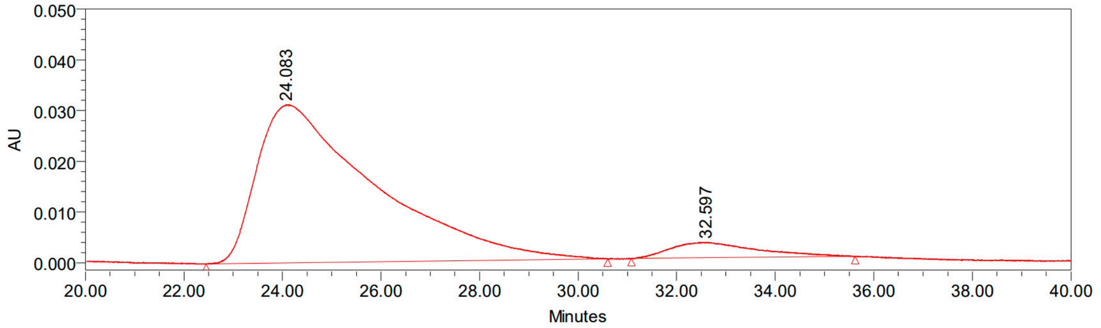

	RT	Area	% Area	Height
1	24.083	5177727	93.38	31130
2	32.597	366936	6.62	3016

(*Z*)-*N*-(3-(2-iodophenyl)-4-oxo-6-phenyl-3,4-dihydro-2*H*-1,3-thiazin-2-ylidene)-3-methylbenzamide (4b)

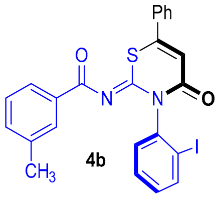

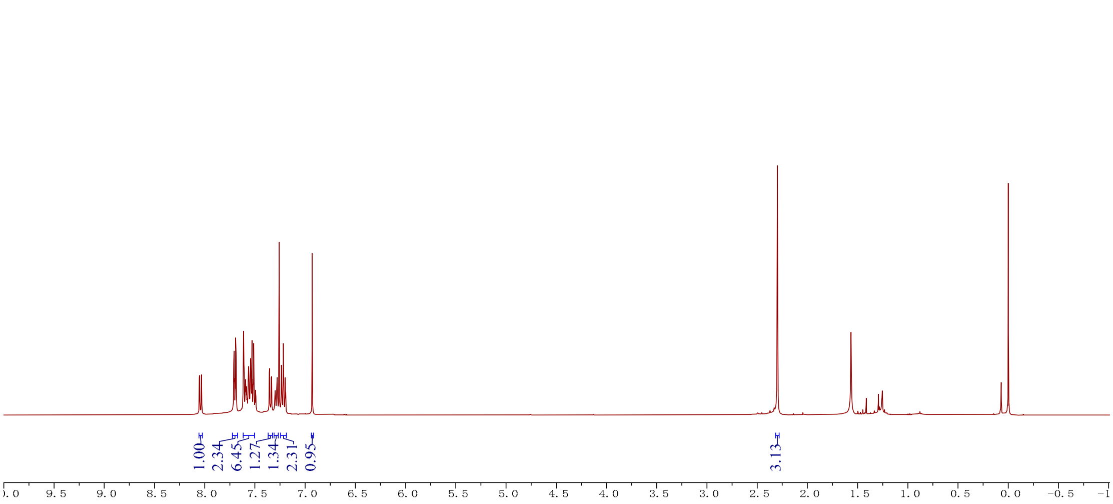

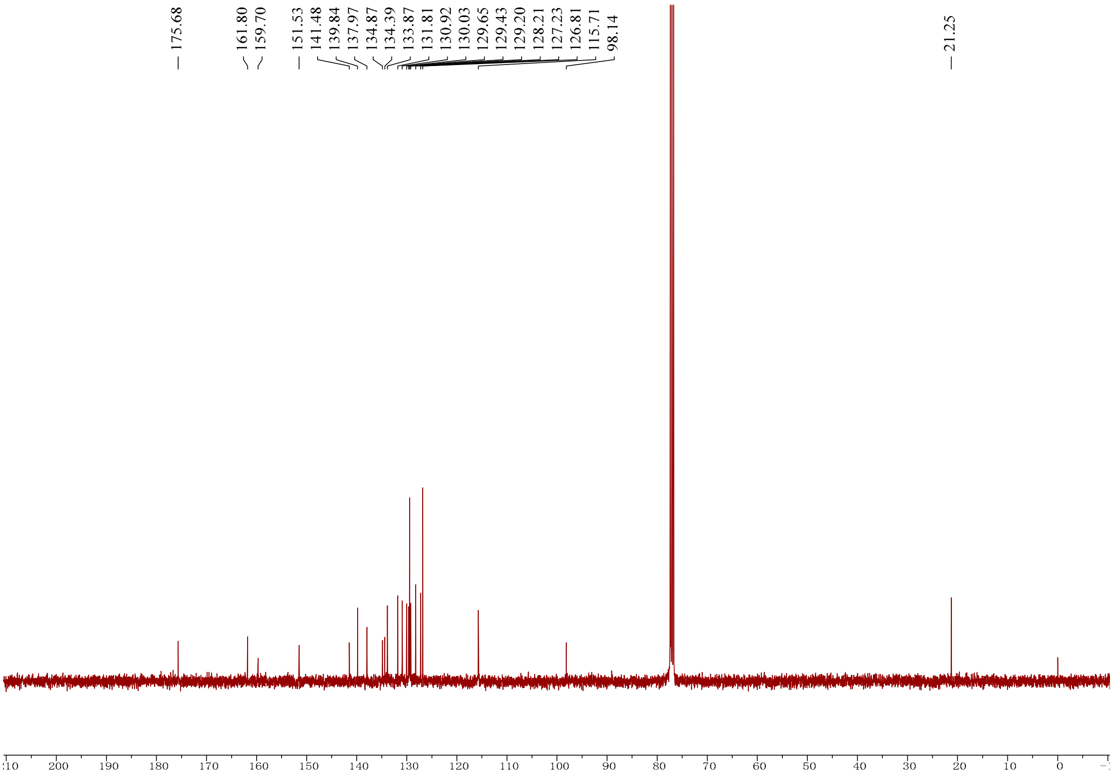

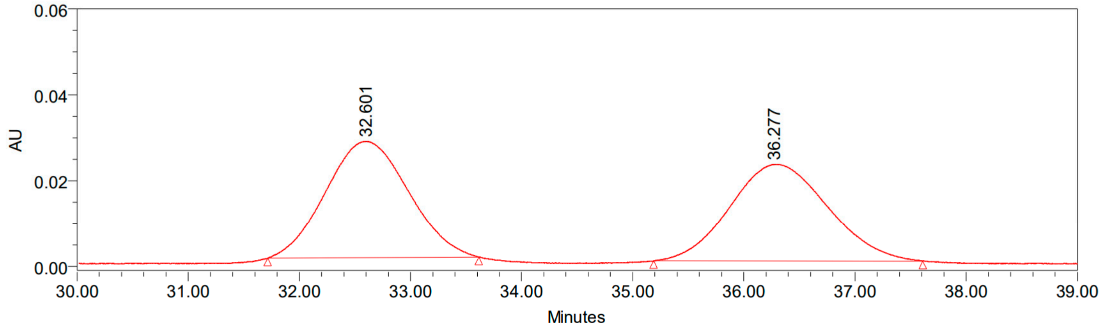

	RT	Area	% Area	Height
1	32.601	1409449	50.46	27184
2	36.277	1383613	49.54	22640

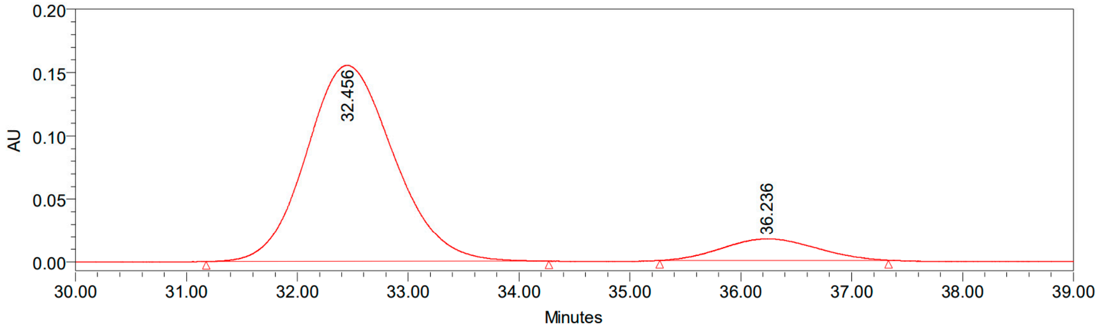

	RT	Area	% Area	Height
1	32.456	8534103	89.63	155161
2	36.236	987617	10.37	16955

(*Z*)-*N*-(3-(2-iodophenyl)-4-oxo-6-phenyl-3,4-dihydro-2*H*-1,3-thiazin-2-ylidene)-3-methoxybenzamide (4c)

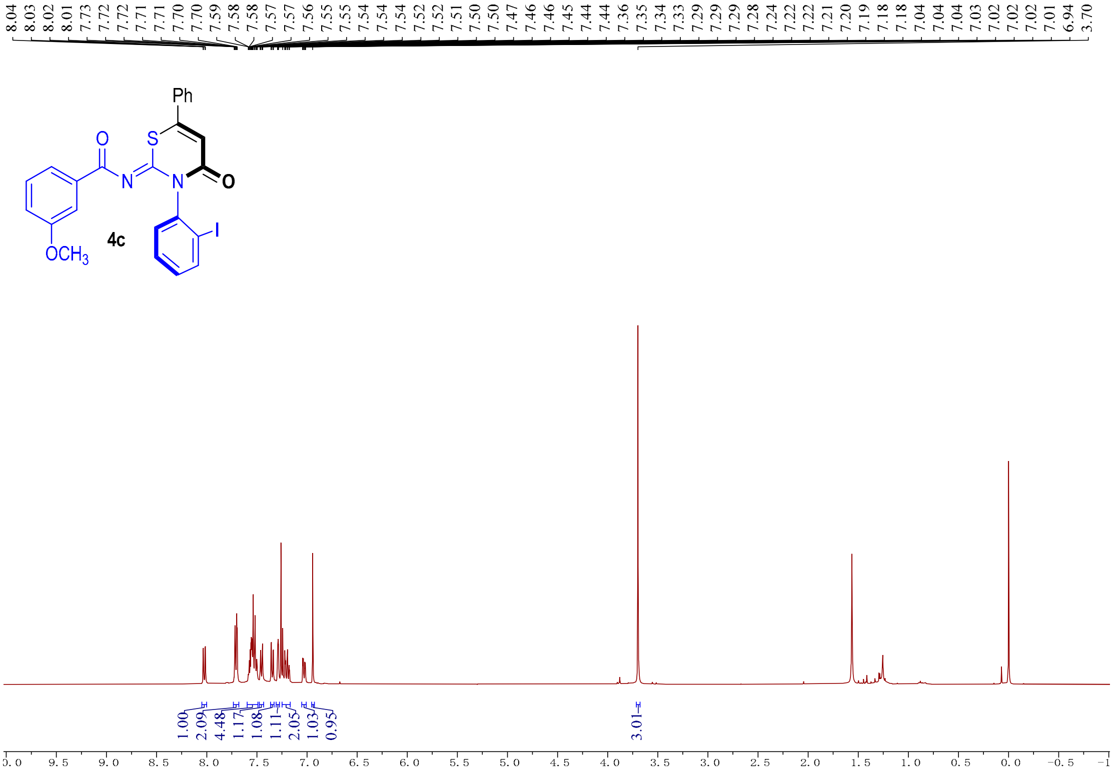

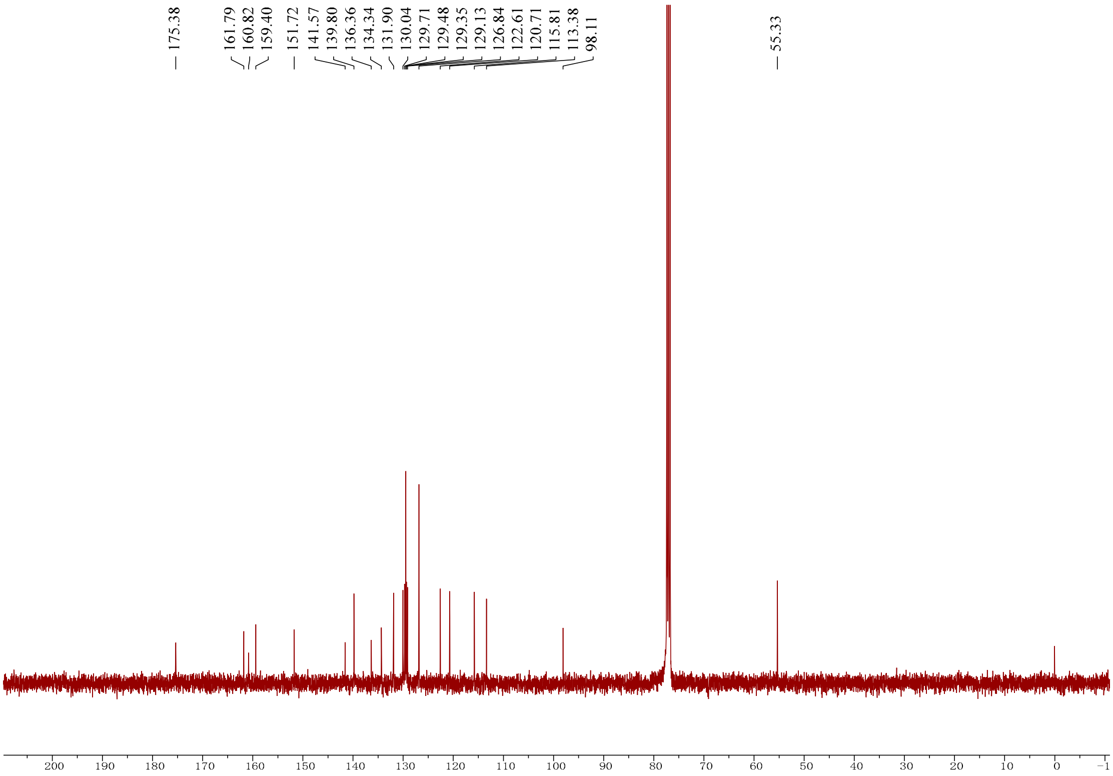

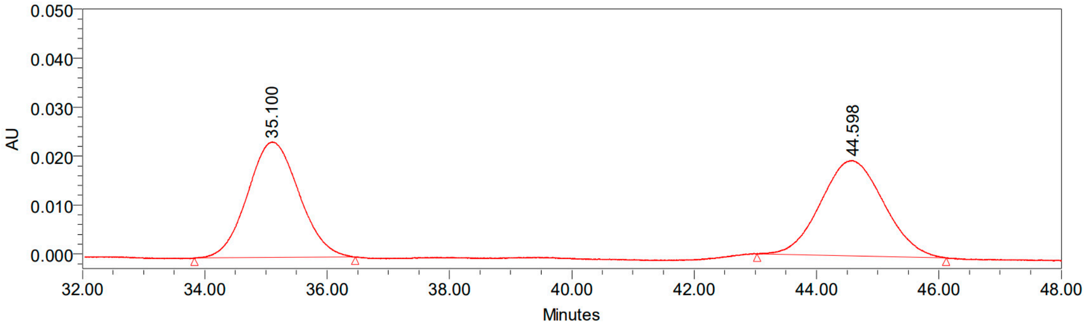

	RT	Area	% Area	Height
1	35.100	1377033	49.72	23569
2	44.598	1392423	50.28	19453

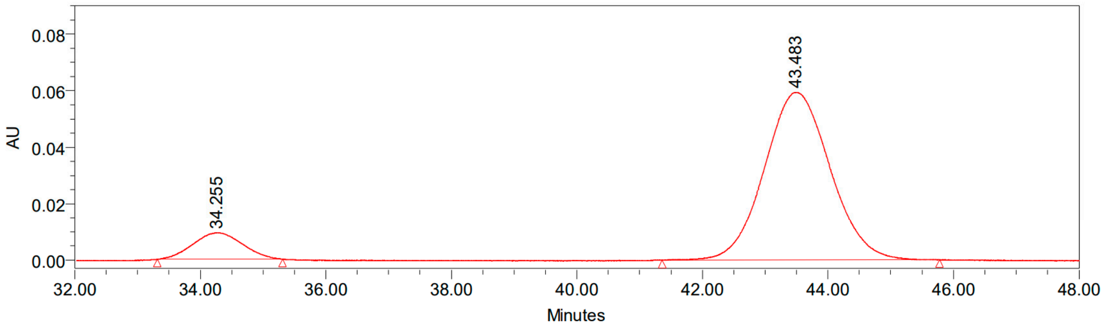

	RT	Area	% Area	Height
1	34.255	507302	10.47	9325
2	43.483	4339773	89.53	59447

(*Z*)-*N*-(3-(2-iodophenyl)-4-oxo-6-phenyl-3,4-dihydro-2*H*-1,3-thiazin-2-ylidene)-2-methylbenzamide (4d)

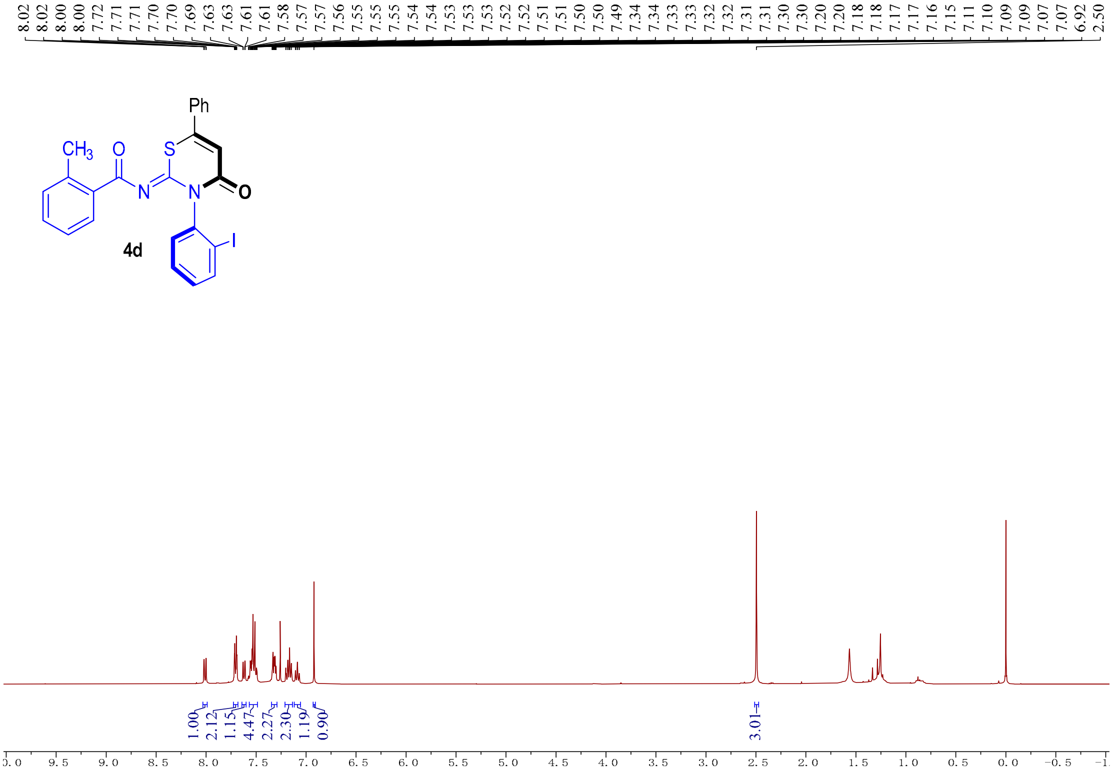

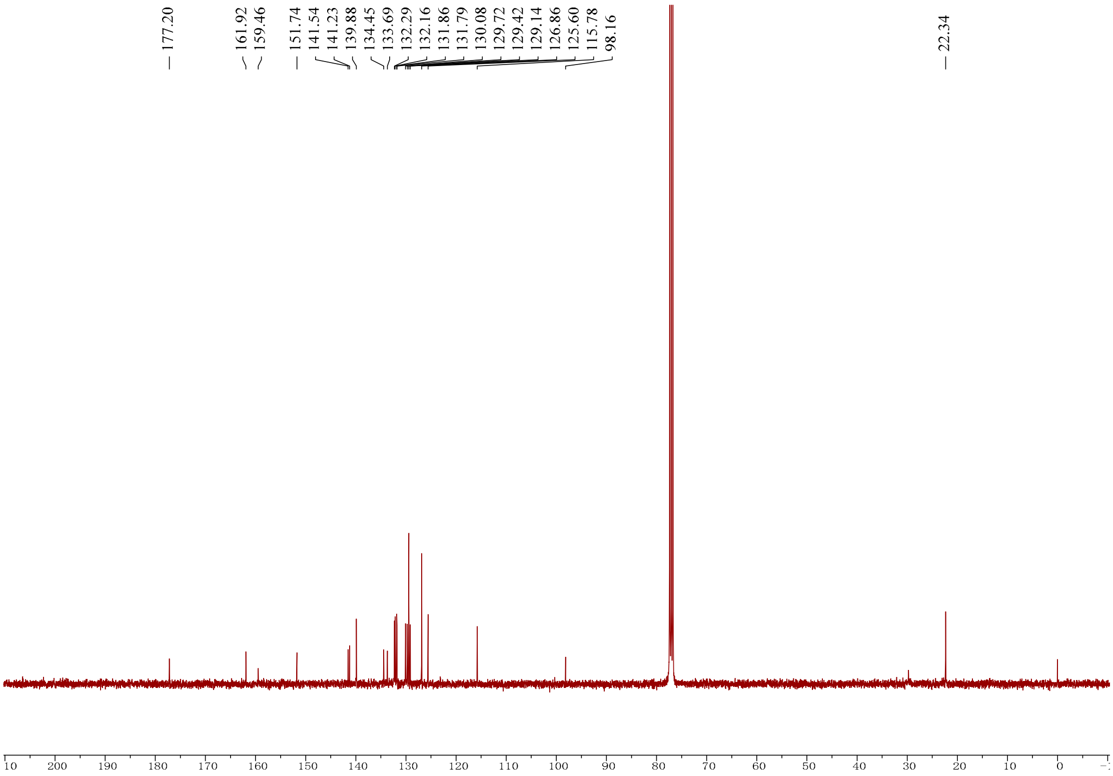

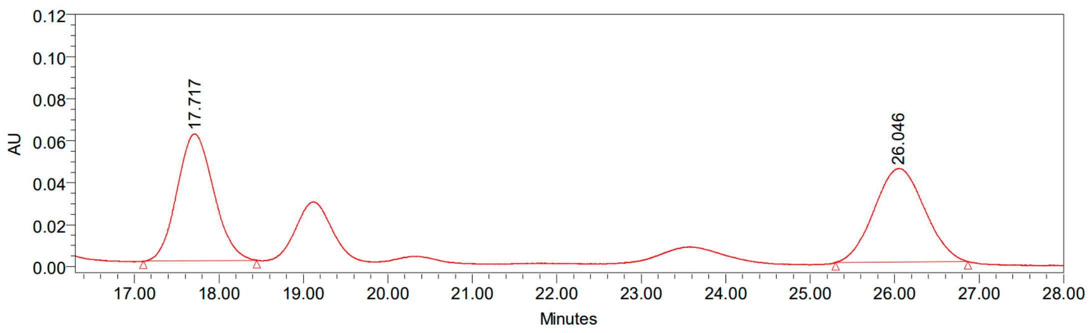

	RT	Area	% Area	Height
1	17.717	1822561	49.64	60446
2	26.046	1848840	50.36	44635

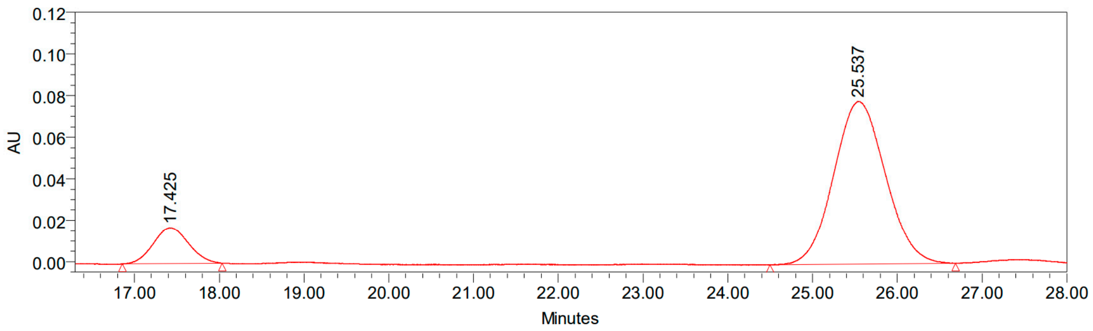

	RT	Area	% Area	Height
1	17.425	498881	13.07	17238
2	25.537	3317518	86.93	78217

(*Z*)-4-fluoro-*N*-(3-(2-iodophenyl)-4-oxo-6-phenyl-3,4-dihydro-2*H*-1,3-thiazin-2-ylidene)benzamide (4e)

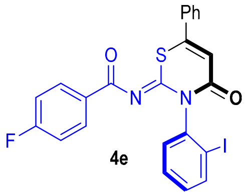

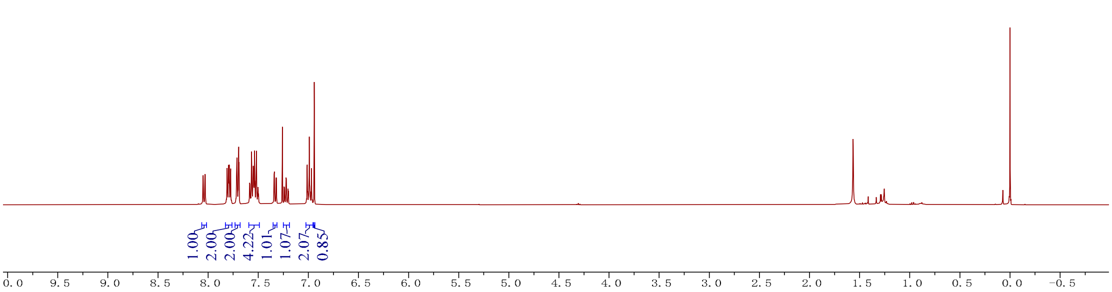

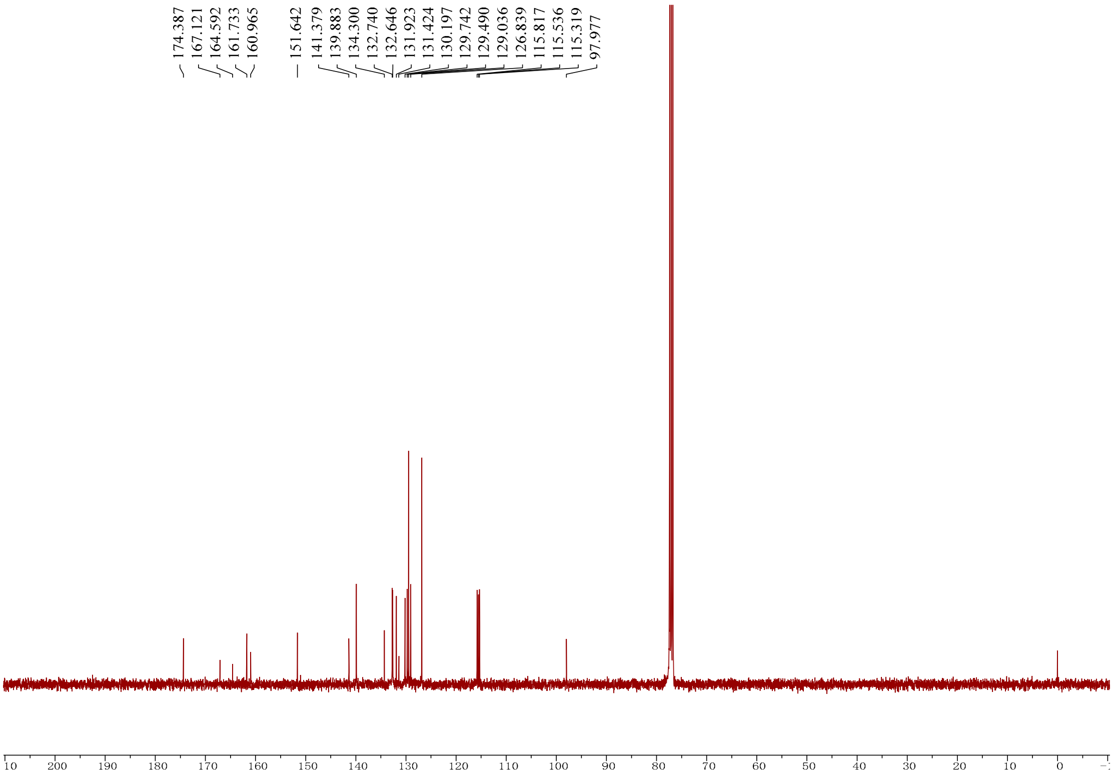

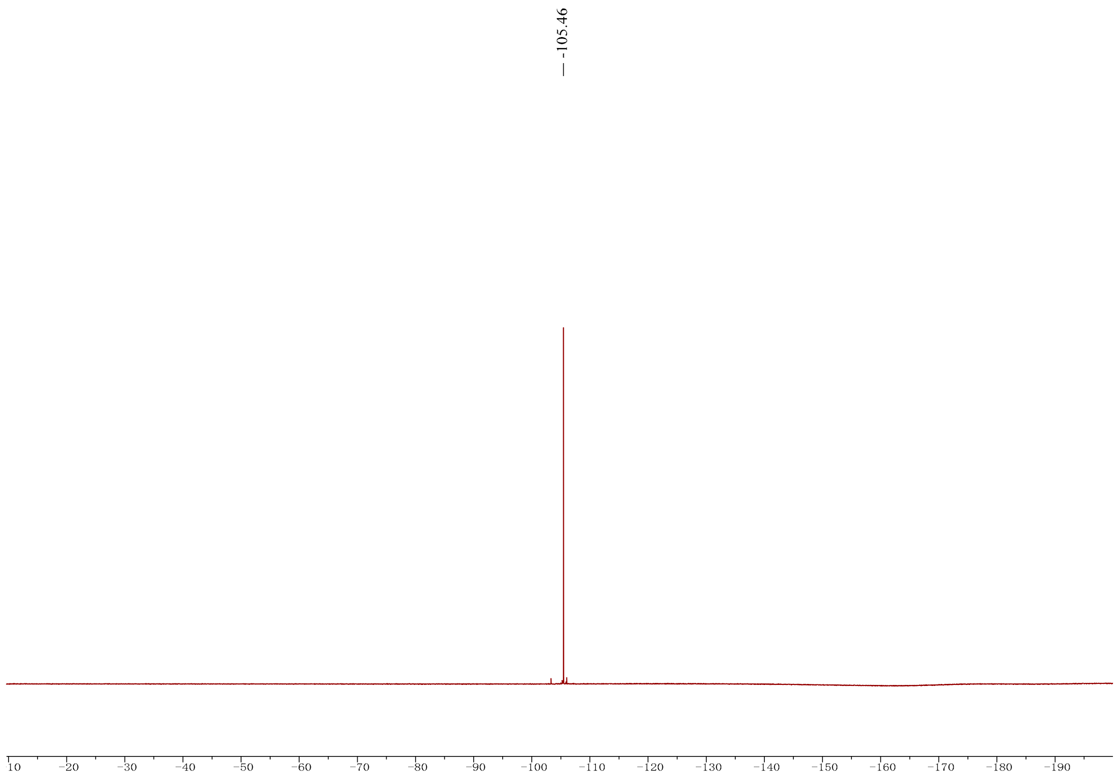
-105.46
10 −20 −30 −40 −50 −60 −70 −80 −90 −100 −110 −120 −130 −140 −150 −160 −170 −180 −190

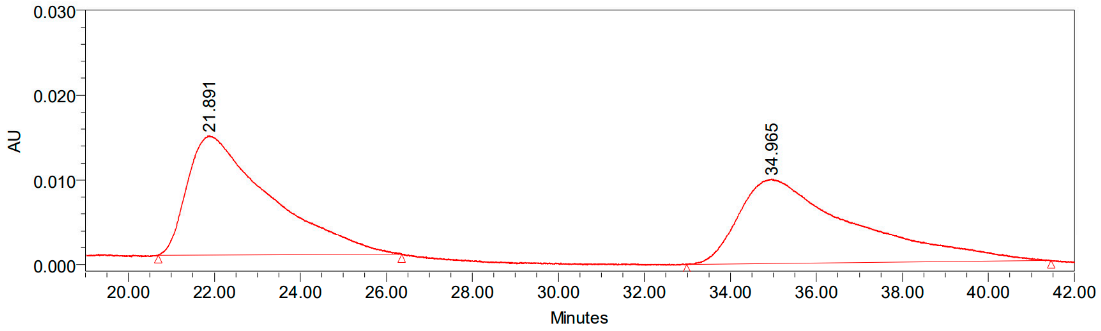

	RT	Area	% Area	Height
1	21.891	1886104	50.47	14098
2	34.965	1851204	49.53	9986

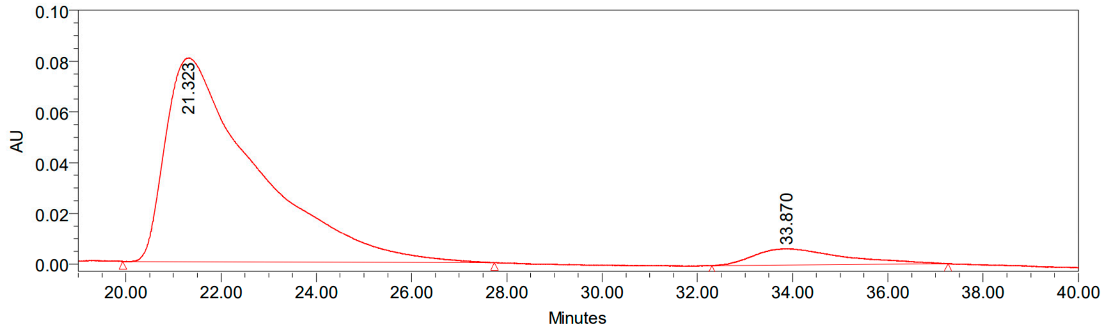

	RT	Area	% Area	Height
1	21.323	10667492	92.77	80433
2	33.870	830960	7.23	6390

(*Z*)-4-chloro-*N*-(3-(2-iodophenyl)-4-oxo-6-phenyl-3,4-dihydro-2*H*-1,3-thiazin-2-ylidene)benzamide (4f)

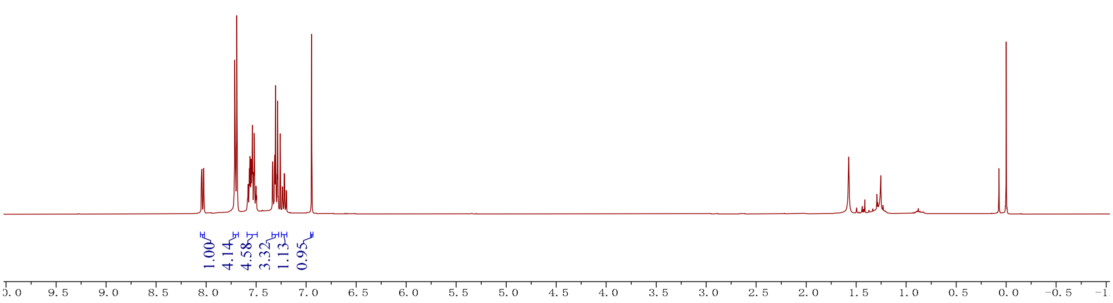

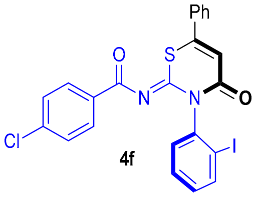

4f

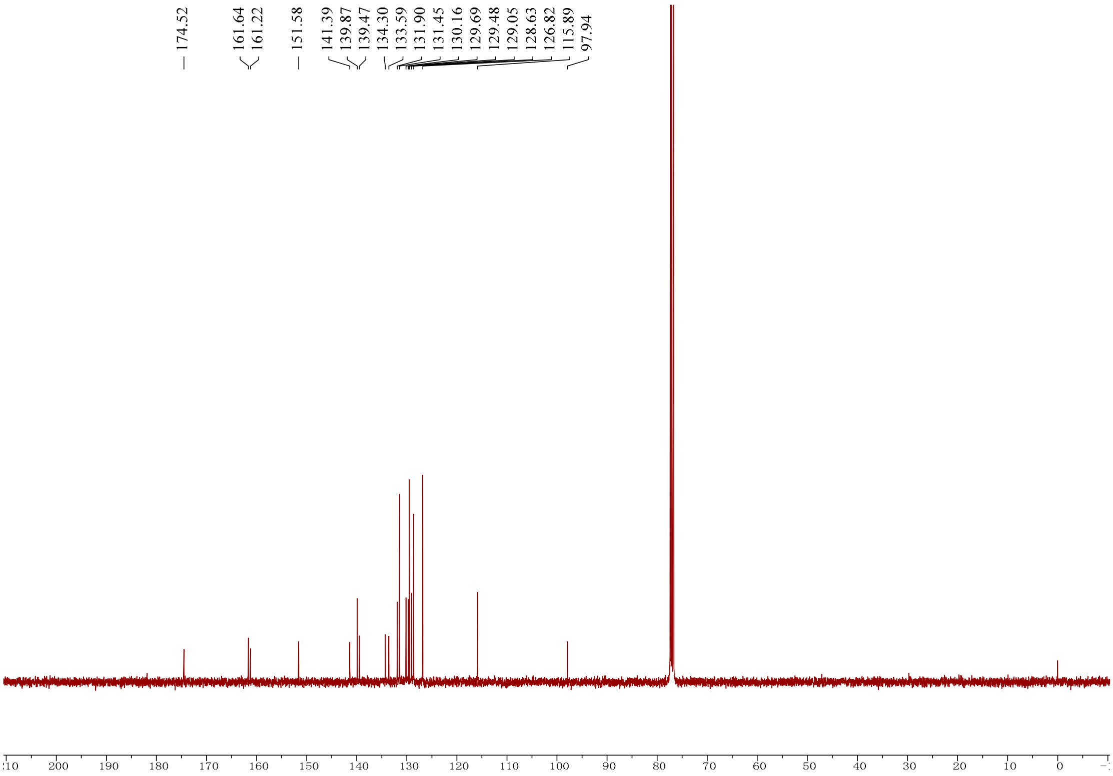

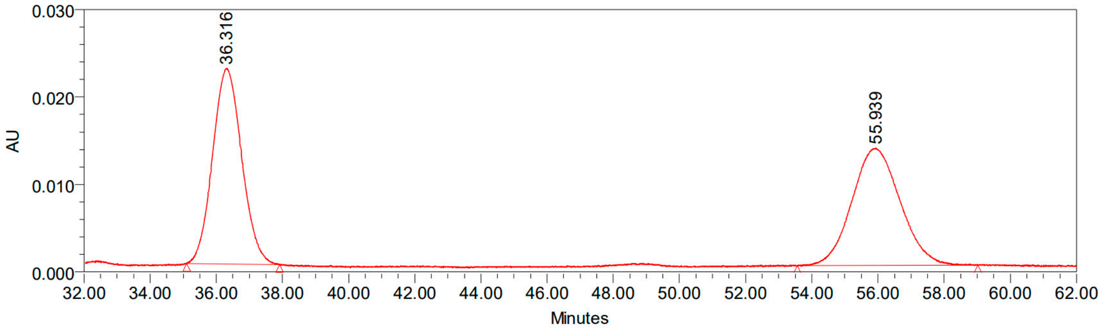

	RT	Area	% Area	Height
1	36.316	1367728	50.26	22338
2	55.939	1353416	49.74	13387

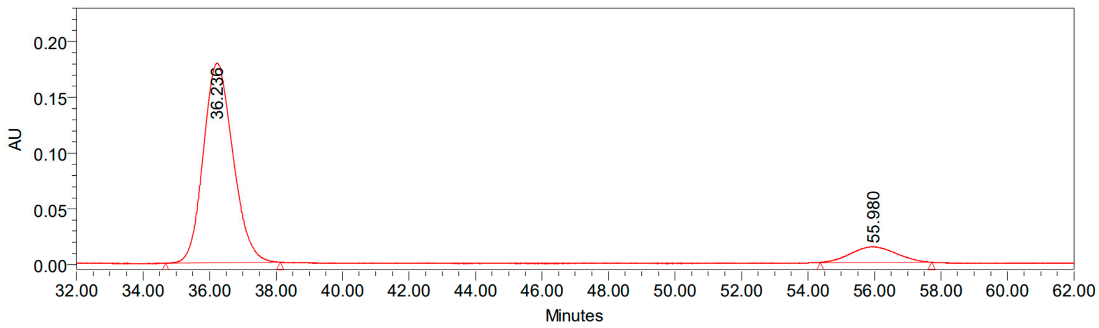

	RT	Area	% Area	Height
1	36.236	11102354	89.55	179081
2	55.980	1295399	10.45	13764

(*Z*)-4-bromo-*N*-(3-(2-iodophenyl)-4-oxo-6-phenyl-3,4-dihydro-2*H*-1,3-thiazin-2-ylidene)benzamide (4g)

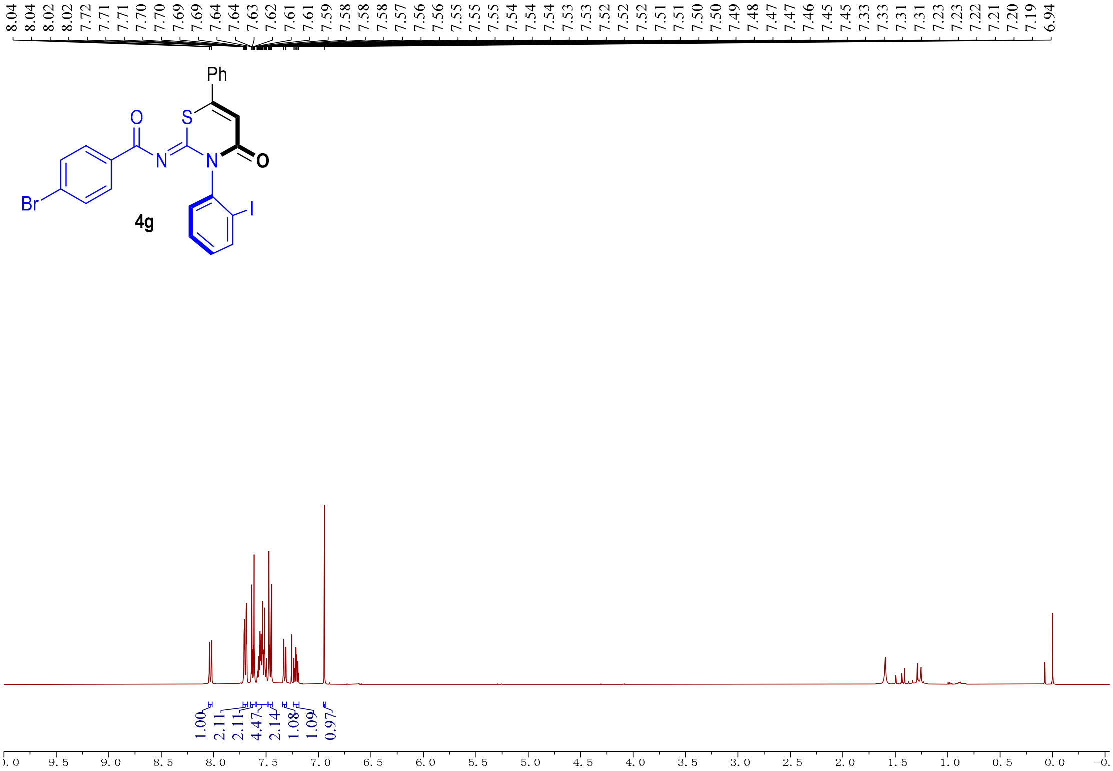

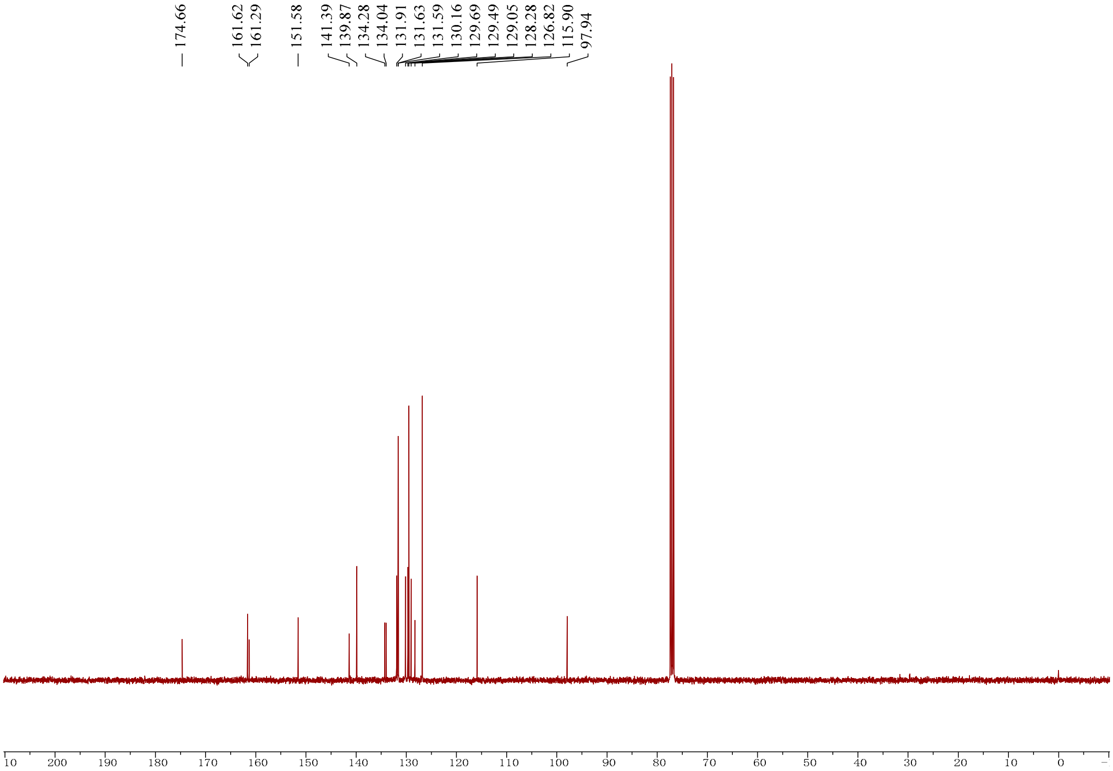

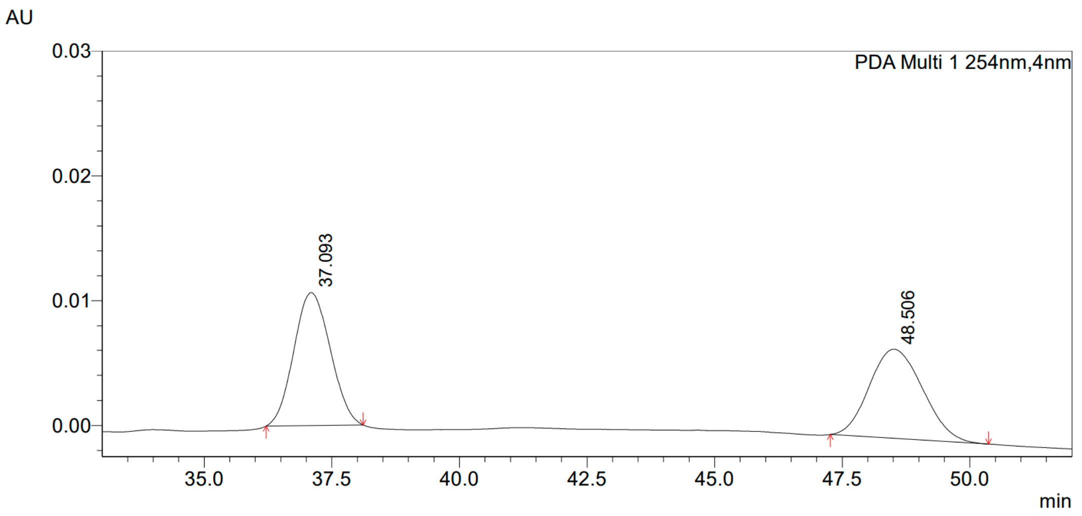

PDA Ch1 254nm

Peak#	Ret. Time	Area	Height	Area%
1	37.093	529667	10658	50.587
2	48.506	517367	7113	49.413
Total		1047034	17770	100.000

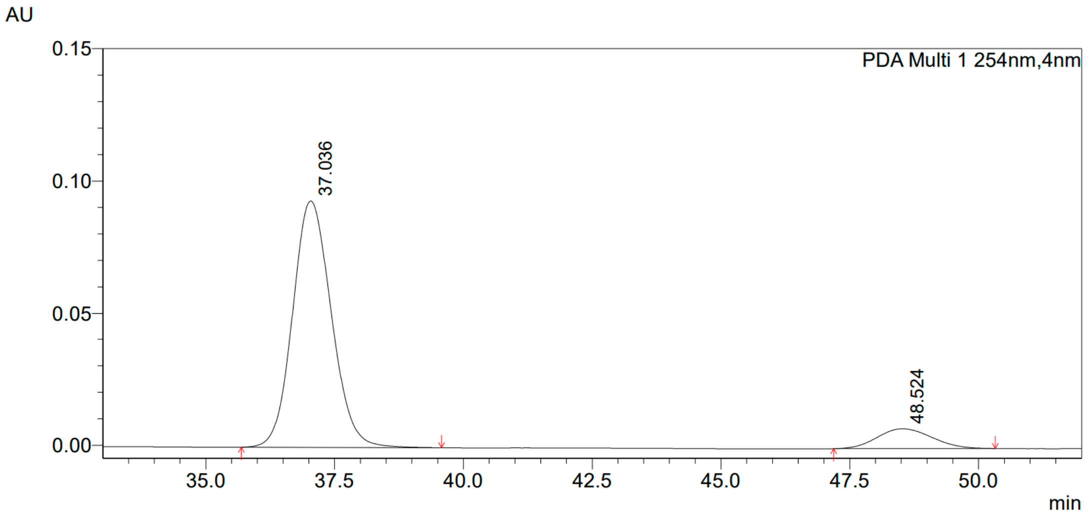

PDA Ch1 254nm

Peak#	Ret. Time	Area	Height	Area%
1	37.036	4880056	93292	89.822
2	48.524	552978	7489	10.178
Total		5433034	100781	100.000

(*Z*)-3-fluoro-*N*-(3-(2-iodophenyl)-4-oxo-6-phenyl-3,4-dihydro-2*H*-1,3-thiazin-2-ylidene)benzamide (4h)

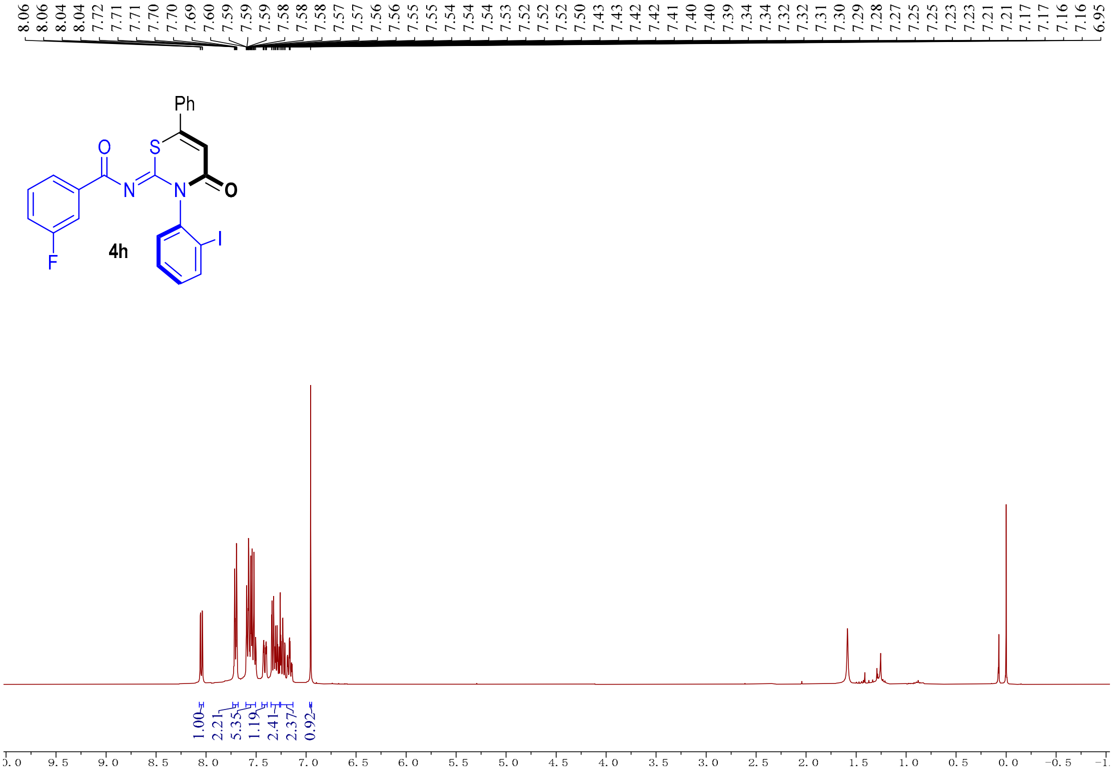

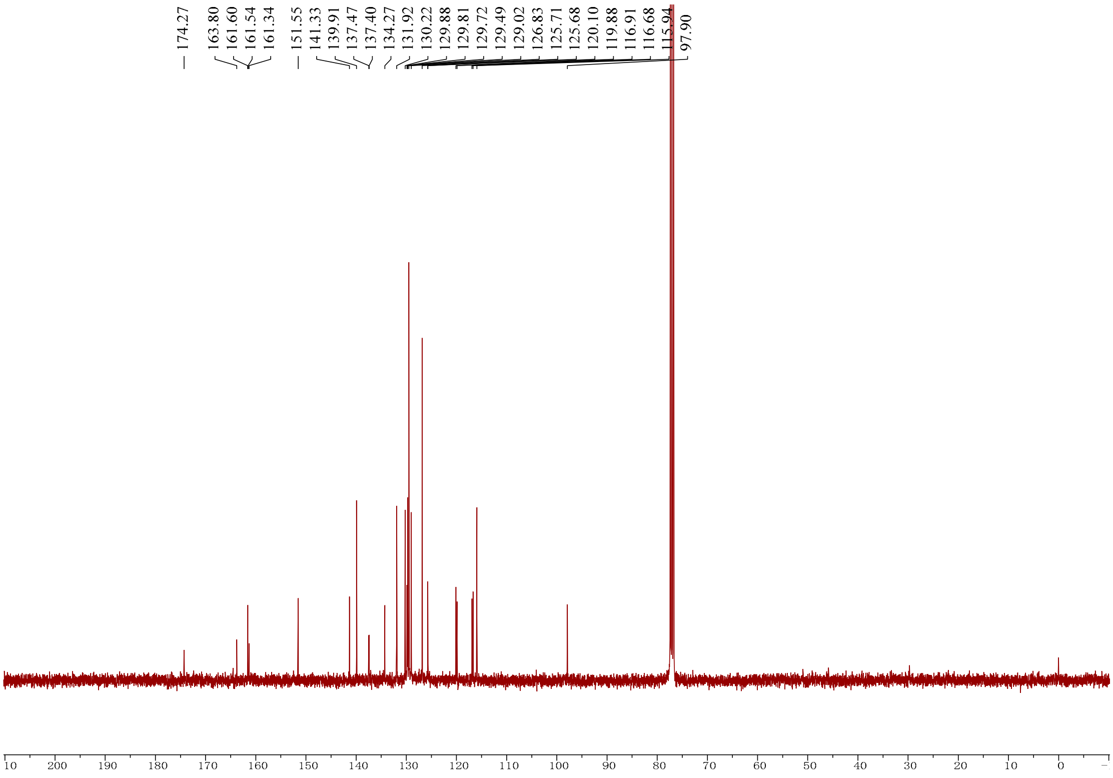

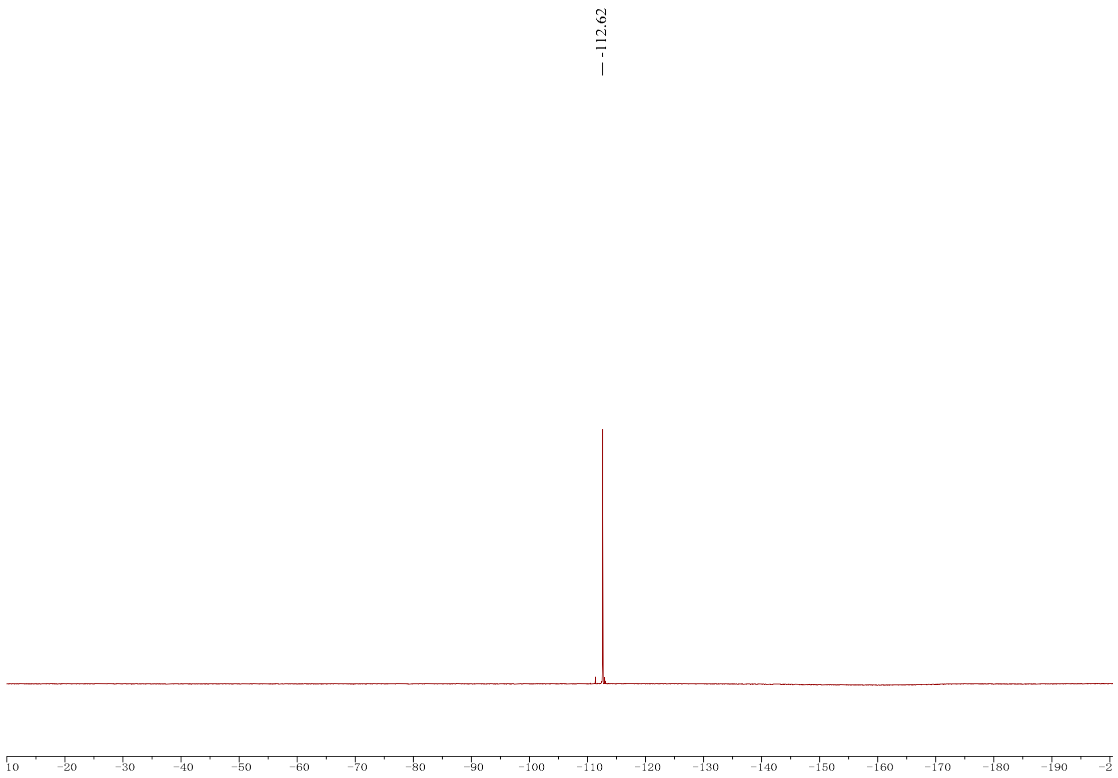
-112.62
10
-20
-30
-40
-50
-60
-70
-80
-90
-100
-110
-120
-130
-140
-150
-160
-170
-180
-190
-2

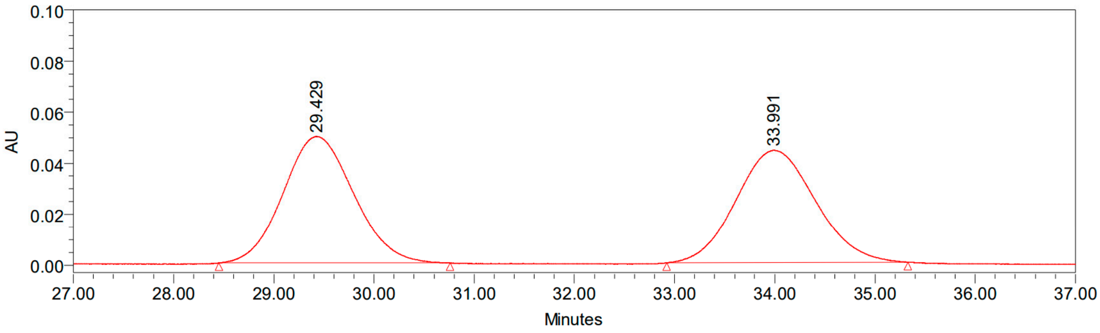

	RT	Area	% Area	Height
1	29.429	2437075	50.02	49656
2	33.991	2434905	49.98	44073

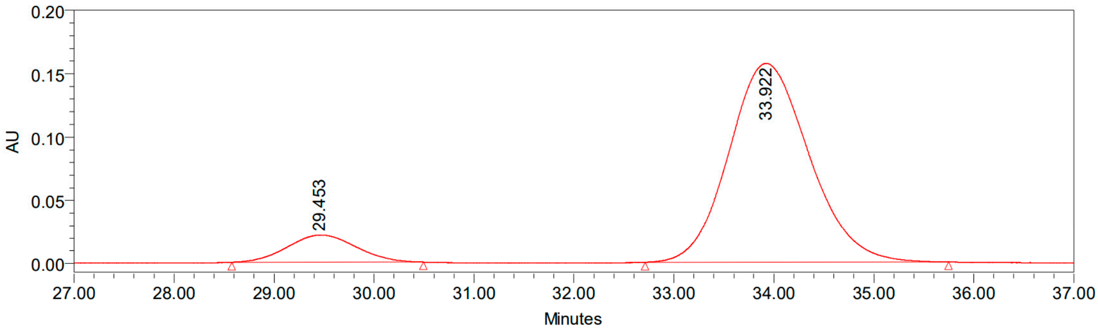

	RT	Area	% Area	Height
1	29.453	1026068	10.45	21446
2	33.922	8790682	89.55	157140

(*Z*)-3-chloro-*N*-(3-(2-iodophenyl)-4-oxo-6-phenyl-3,4-dihydro-2*H*-1,3-thiazin-2-ylidene)benzamide (4i)

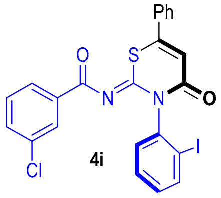

4i

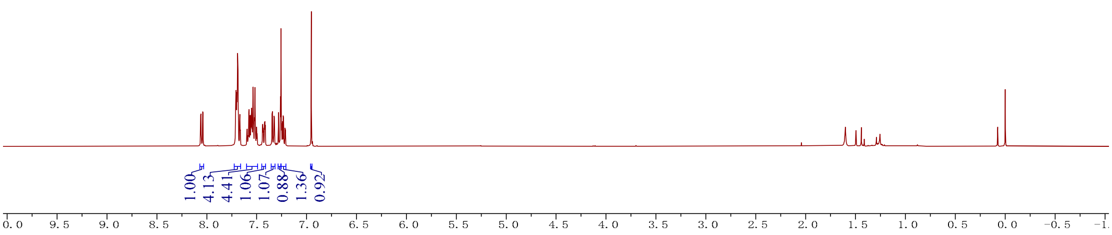

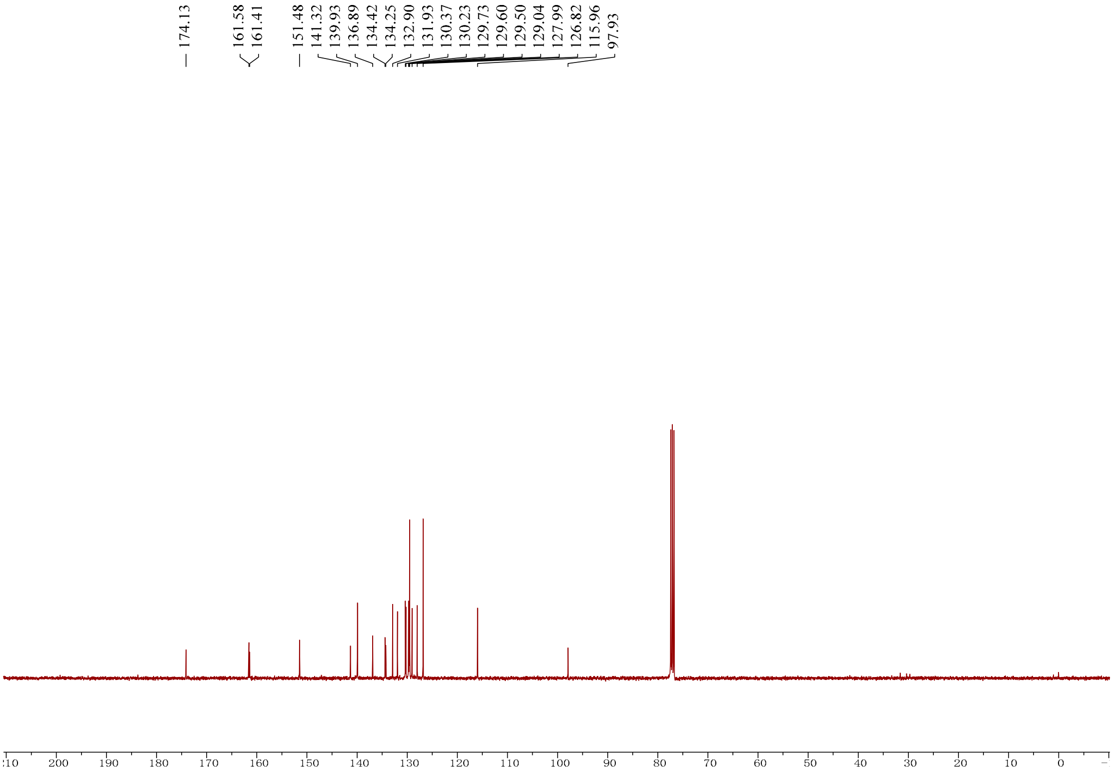

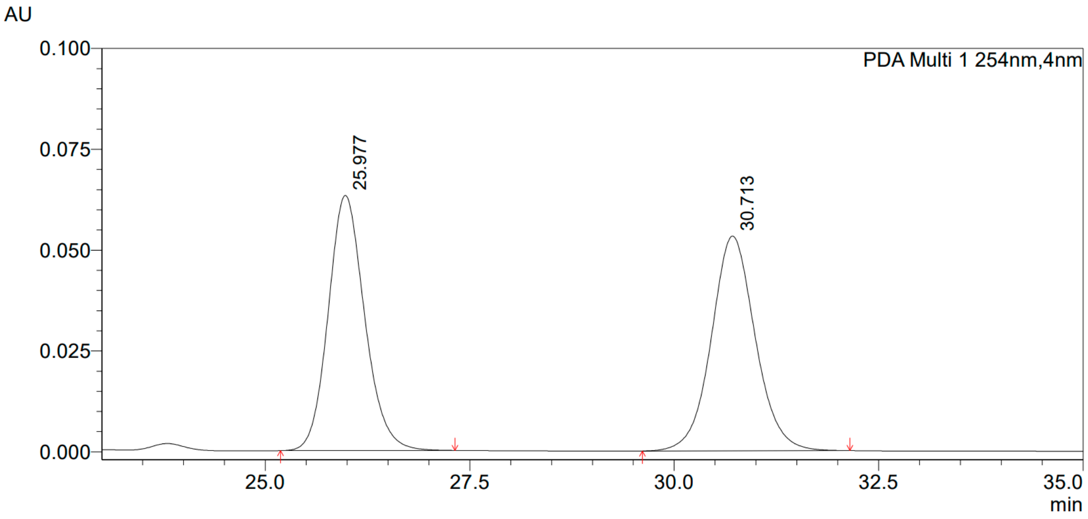

PDA Ch1 254nm

Peak#	Ret. Time	Area	Height	Area%
1	25.977	1965216	63276	49.645
2	30.713	1993339	53242	50.355
Total		3958556	116518	100.000

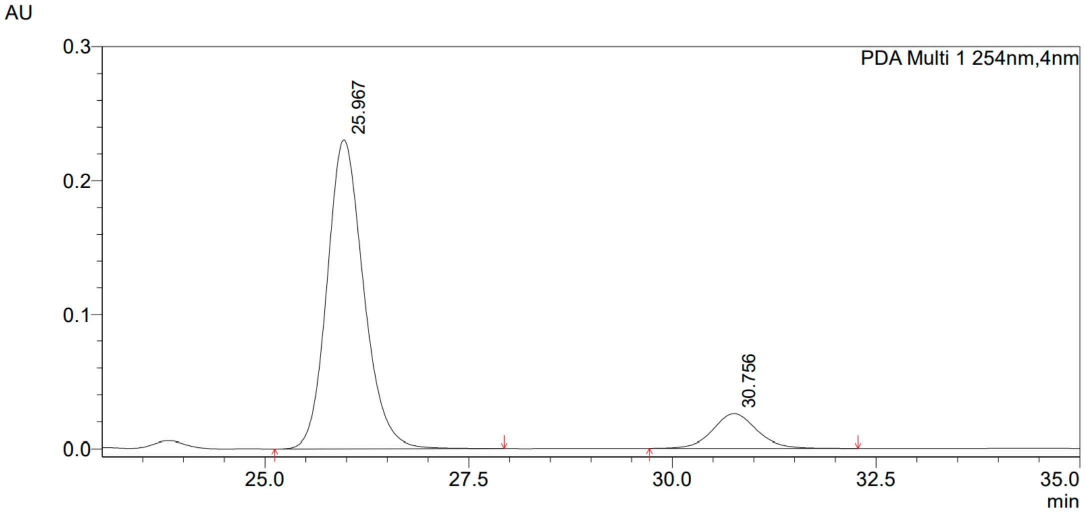

PDA Ch1 254nm

Peak#	Ret. Time	Area	Height	Area%
1	25.967	7170221	230509	88.231
2	30.756	956423	25506	11.769
Total		8126644	256015	100.000

(*Z*)-3-bromo-*N*-(3-(2-iodophenyl)-4-oxo-6-phenyl-3,4-dihydro-2*H*-1,3-thiazin-2-ylidene)benzamide (4j)

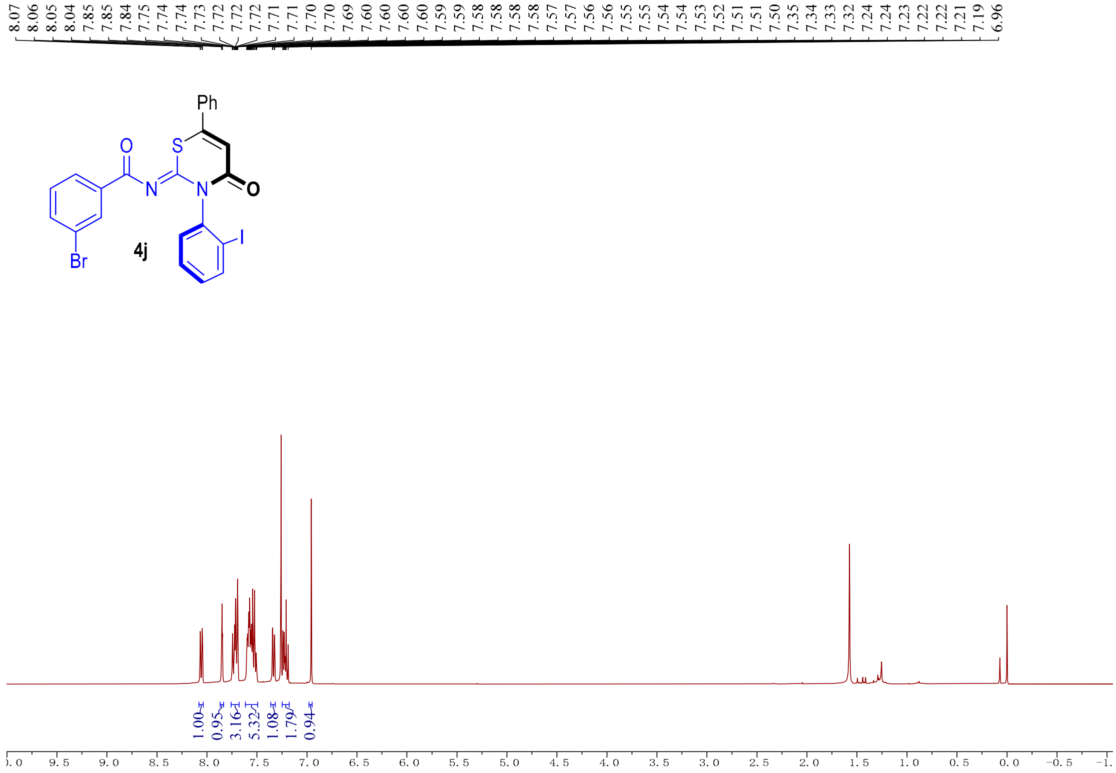

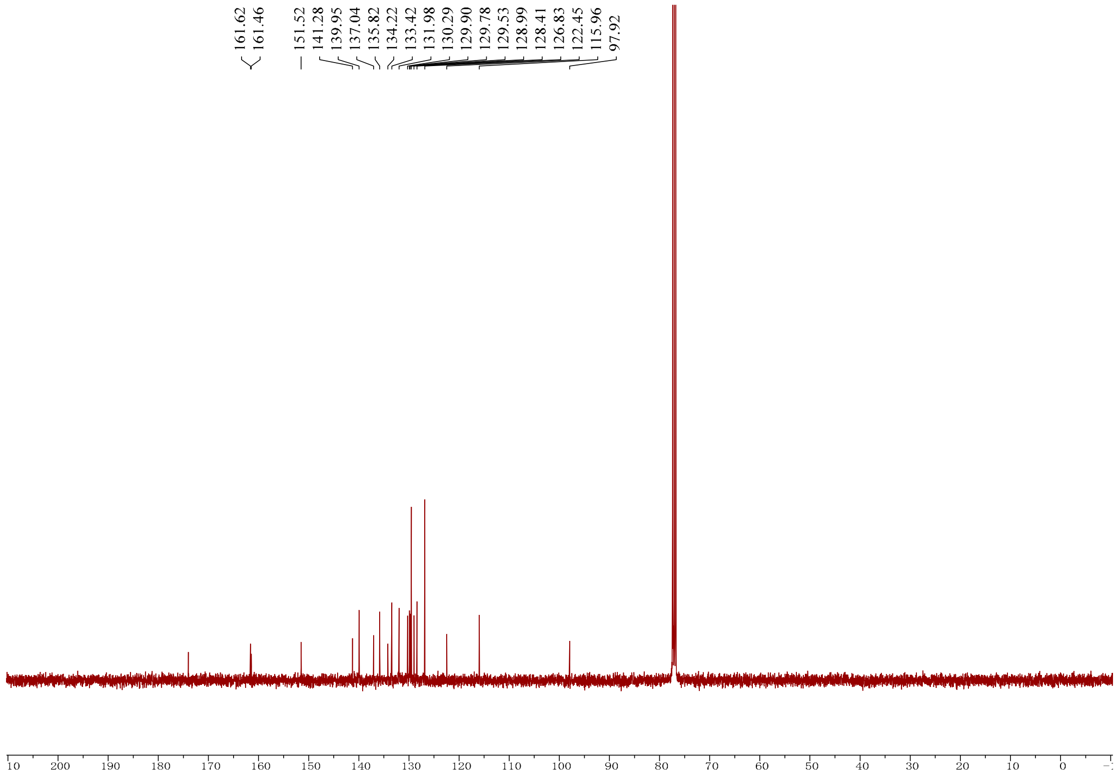

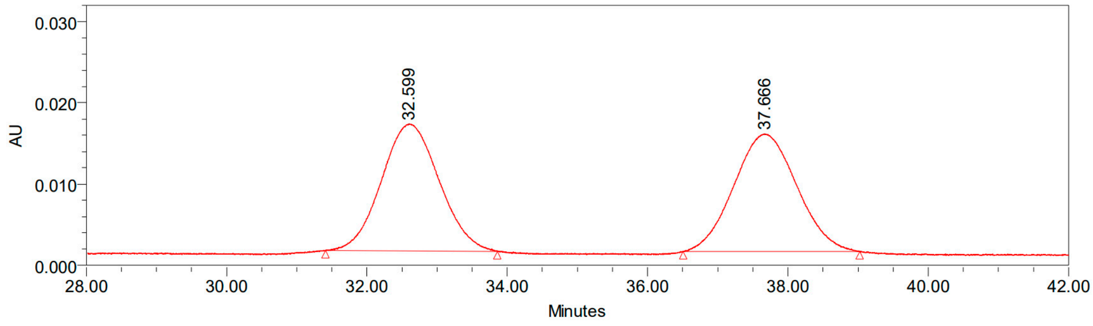

	RT	Area	% Area	Height
1	32.599	890809	49.71	15596
2	37.666	901377	50.29	14472

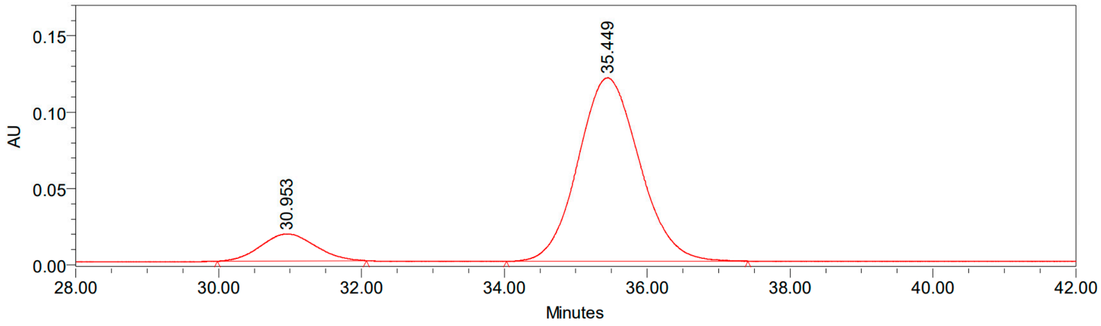

	RT	Area	% Area	Height
1	30.953	927673	11.45	17650
2	35.449	7170749	88.55	120316

(*Z*)-2-fluoro-*N*-(3-(2-iodophenyl)-4-oxo-6-phenyl-3,4-dihydro-2*H*-1,3-thiazin-2-ylidene)benzamide (4k)

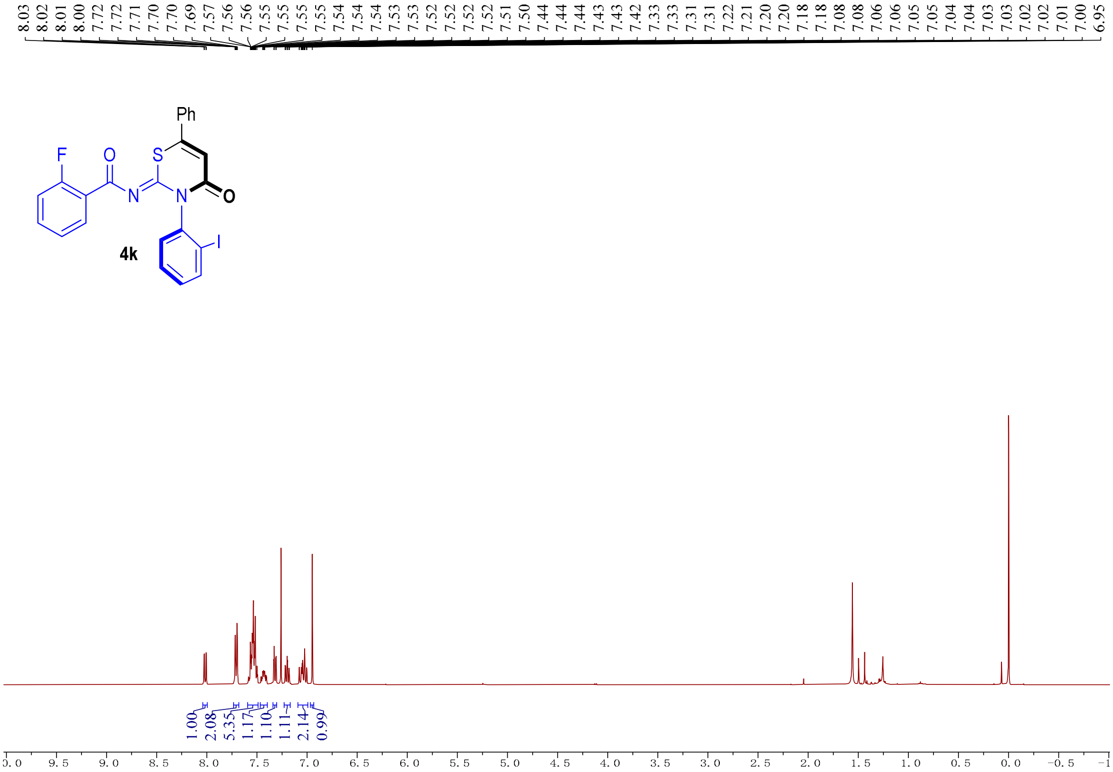

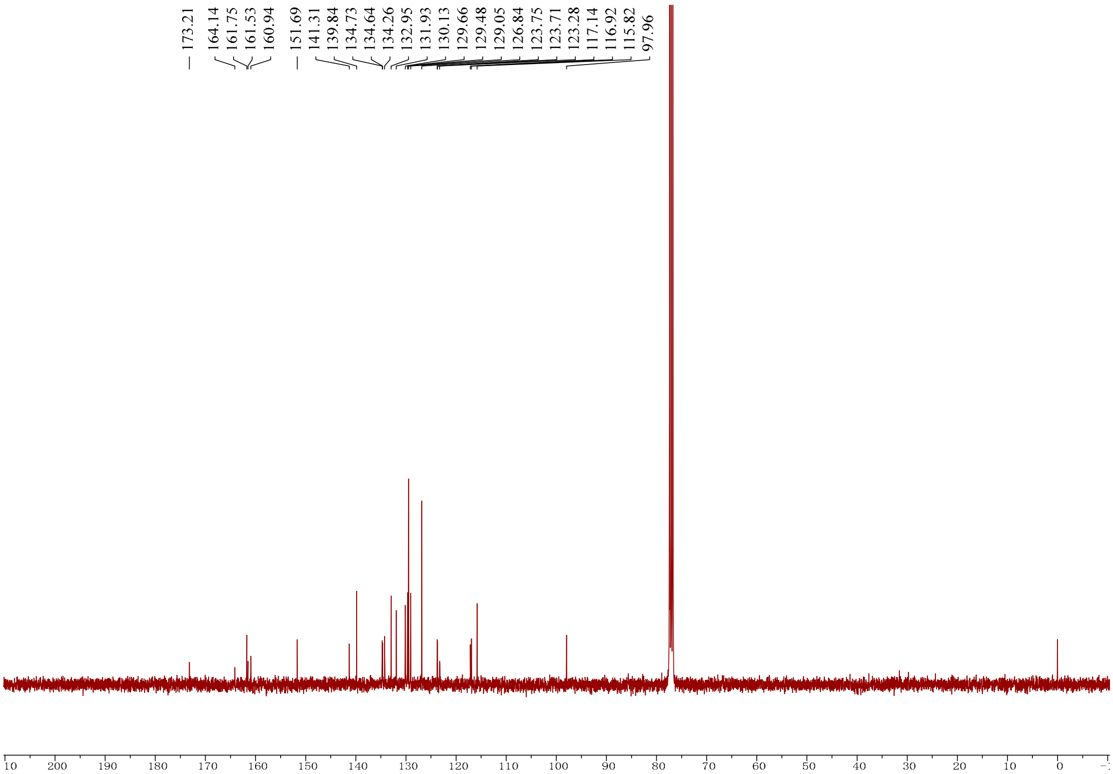

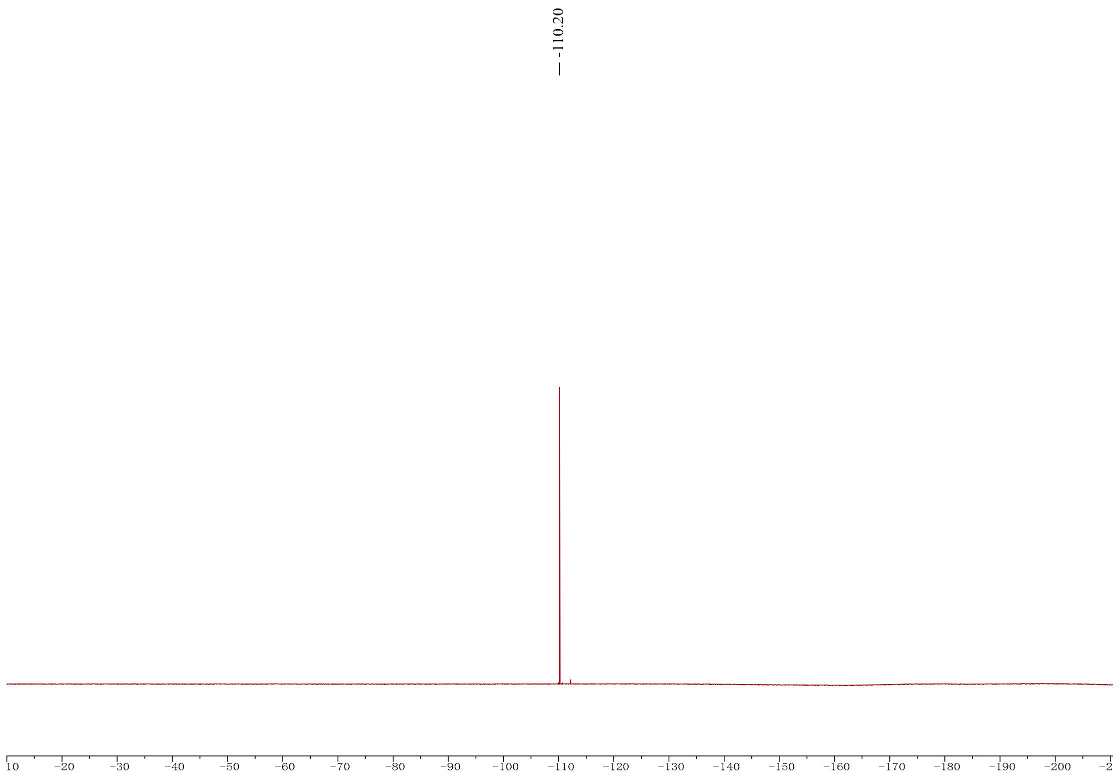
-110.20
10 -20 -30 -40 -50 -60 -70 -80 -90 -100 -110 -120 -130 -140 -150 -160 -170 -180 -190 -200 -2

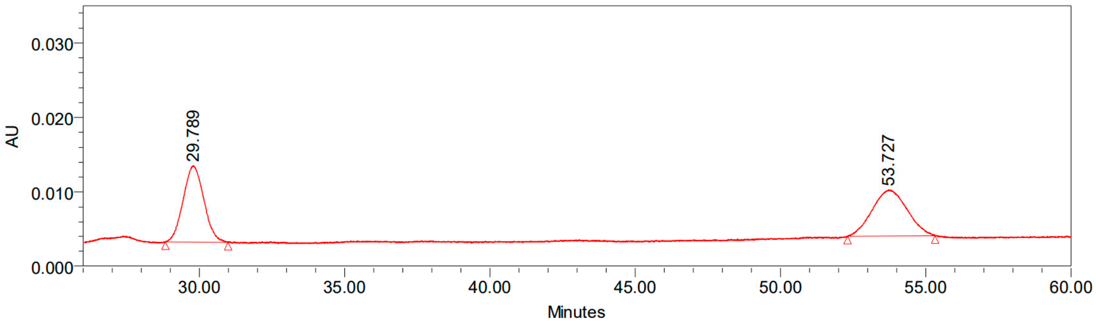

	RT	Area	% Area	Height
1	29.789	509287	49.53	10299
2	53.727	518996	50.47	6198

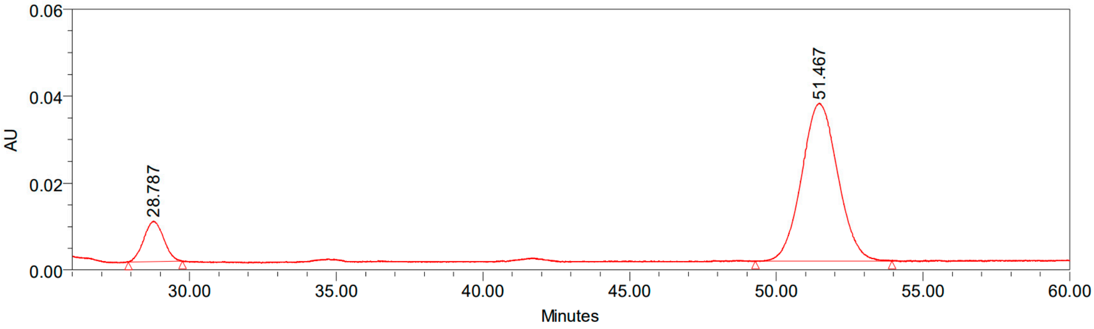

	RT	Area	% Area	Height
1	28.787	437775	12.30	9393
2	51.467	3122026	87.70	36314

(*Z*)-2-chloro-*N*-(3-(2-iodophenyl)-4-oxo-6-phenyl-3,4-dihydro-2*H*-1,3-thiazin-2-ylidene)benzamide (4l)

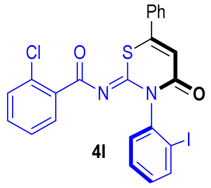

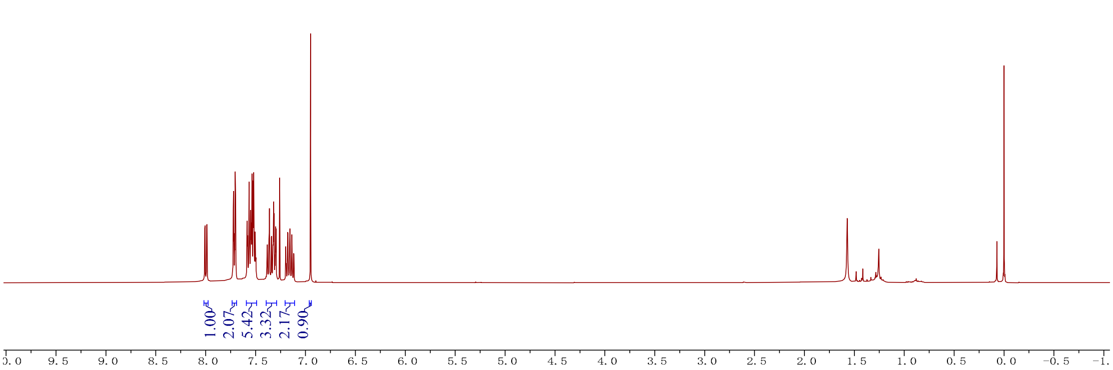

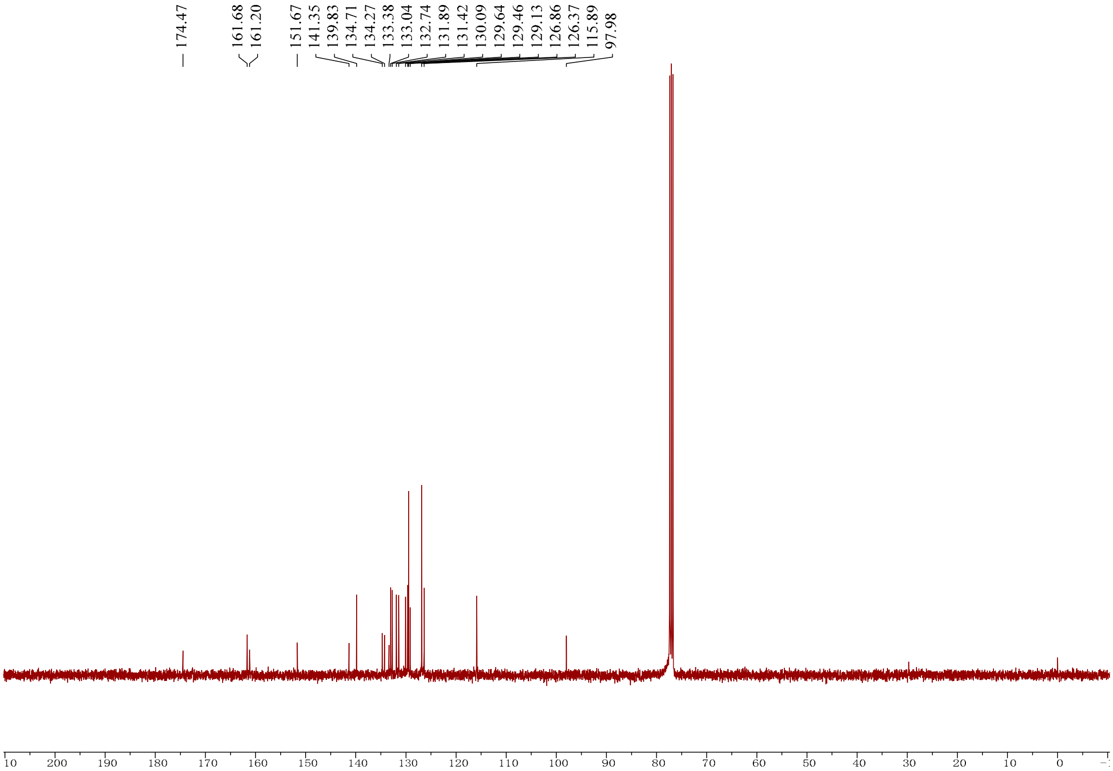

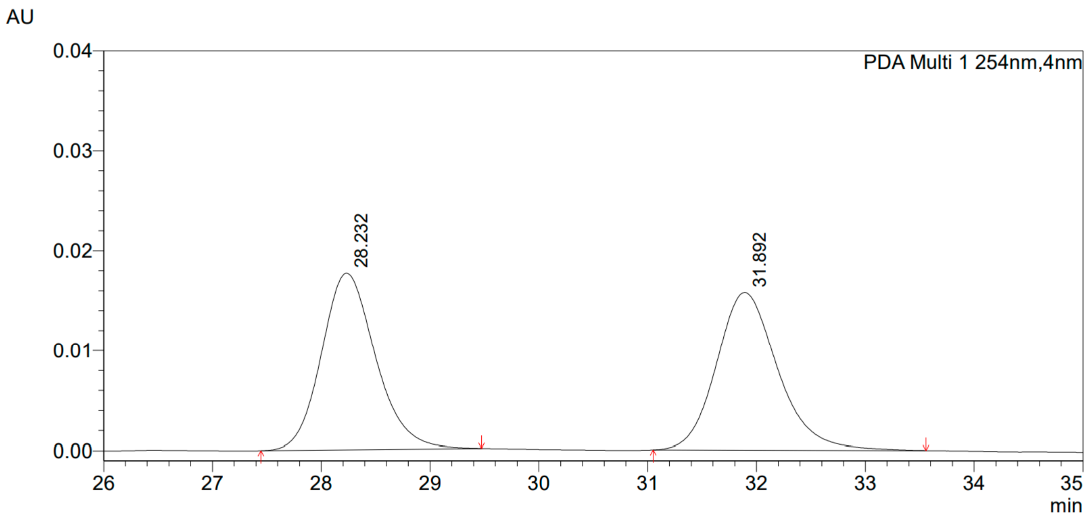

PDA Ch1 254nm

Peak#	Ret. Time	Area	Height	Area%
1	28.232	610289	17668	49.723
2	31.892	617086	15756	50.277
Total		1227374	33423	100.000

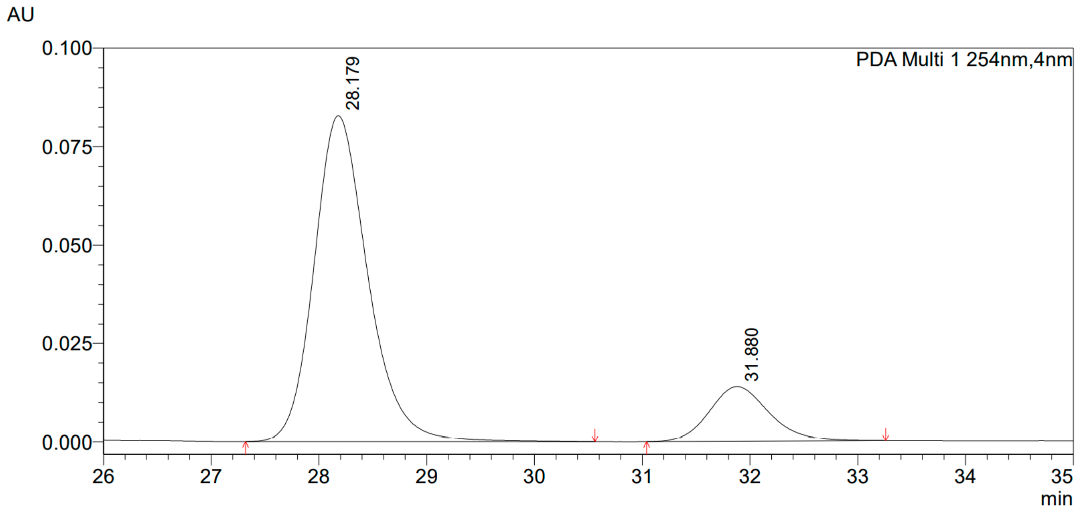

PDA Ch1 254nm

Peak#	Ret. Time	Area	Height	Area%
1	28.179	2876424	82776	84.349
2	31.880	533741	13817	15.651
Total		3410165	96593	100.000

(*Z*)-2-bromo-*N*-(3-(2-iodophenyl)-4-oxo-6-phenyl-3,4-dihydro-2*H*-1,3-thiazin-2-ylidene)benzamide (4m)

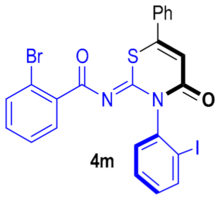

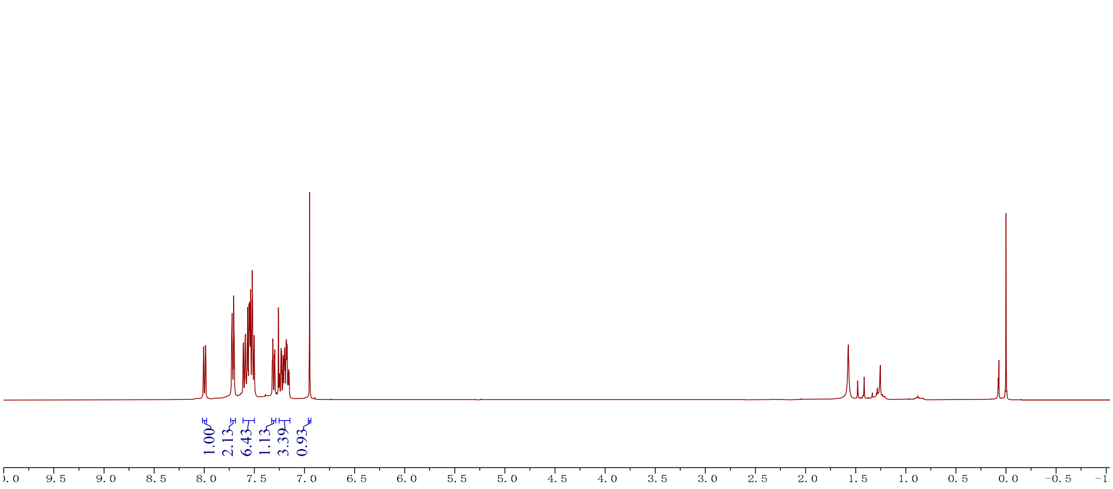

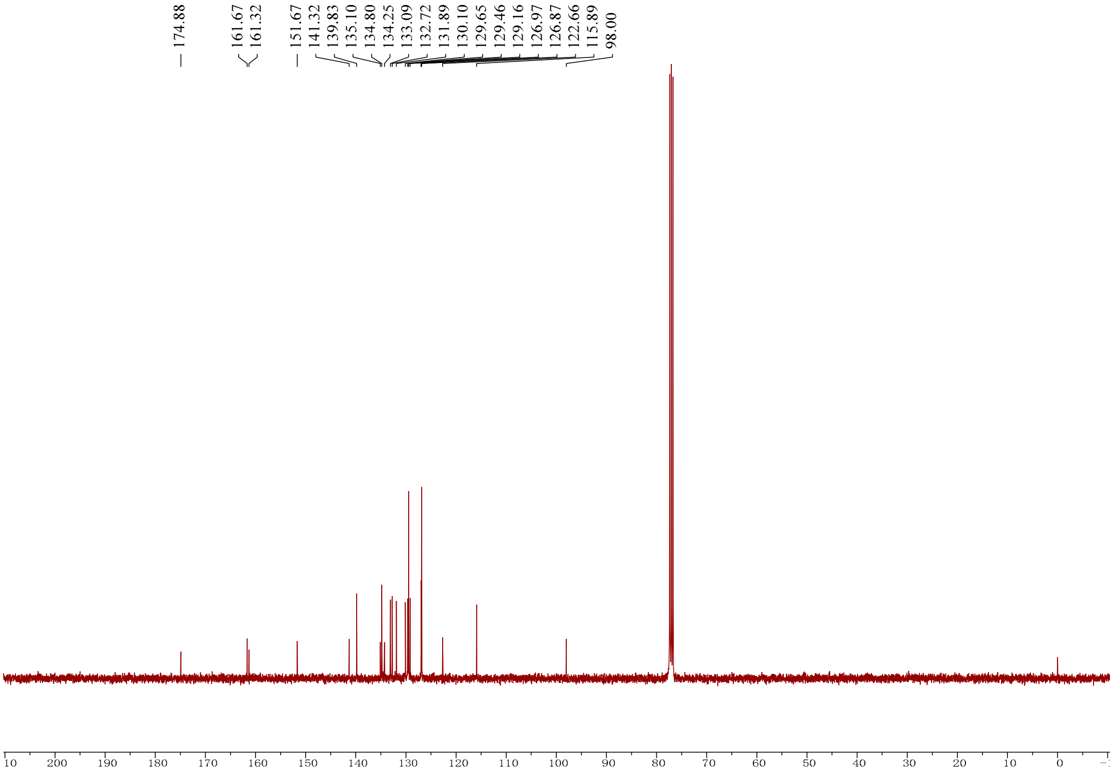

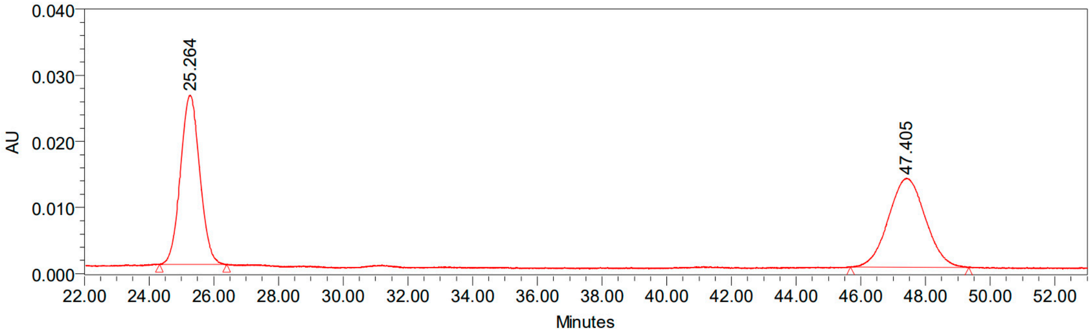

	RT	Area	% Area	Height
1	25.264	1061341	49.96	25580
2	47.405	1063158	50.04	13430

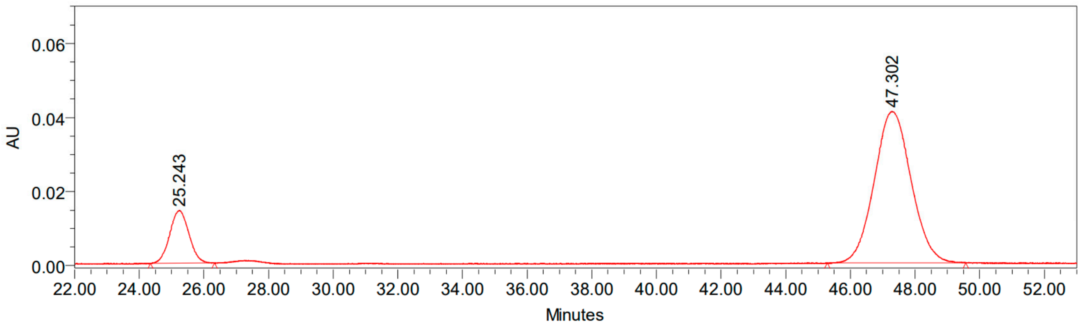

	RT	Area	% Area	Height
1	25.243	583030	15.25	14281
2	47.302	3241262	84.75	40945
